# Photochemical Generation of Allenylidenes from Cyclopropanated Phenanthrenes: An Experimental and Computational Study

**DOI:** 10.1021/acs.joc.4c00147

**Published:** 2024-05-29

**Authors:** Alexander D. Roth, David R. Ramgren, Yuewei Wen, Megan S. Michie, Dasan M. Thamattoor

**Affiliations:** Department of Chemistry, Colby College, Waterville, Maine 04901, United States

## Abstract

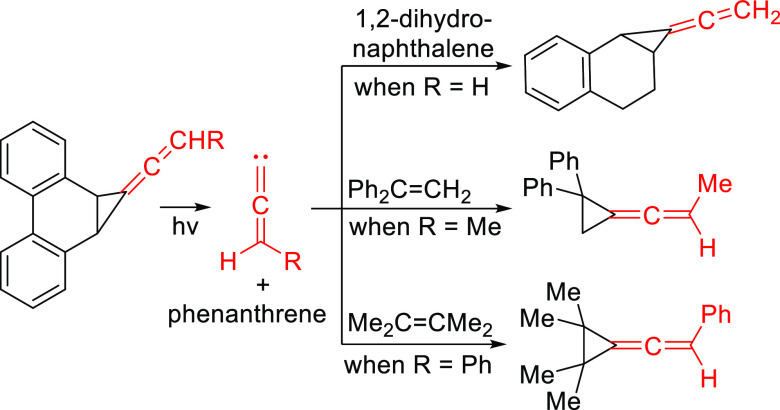

To address the scarcity of generally applicable photochemical routes to allenylidenes in solution, phenanthrene-based sources have been investigated. Specifically, the syntheses of 1-vinylidene-1a,9b-dihydro-1*H*-cyclopropa[*l*]phenanthrene, 1-(2-phenylvinylidene)-1a,9b-dihydro-1*H*-cyclopropa[*l*]phenanthrene, and 1-(2-methylvinylidene)-1a,9b-dihydro-1*H*-cyclopropa[*l*]phenanthrene, photochemical precursors to propadienylidene, 3-phenylpropadienylidene, and 3-methylpropadienylidene have been carried out. Photolysis of these new precursors in olefin traps and benzene afforded the expected cyclopropane adducts of the corresponding allenylidenes. Quantum chemical calculations show that the ground state of all three carbenes is a singlet with a singlet–triplet gap of ∼29, 30, and 33 kcal/mol for propadienylidene, 3-phenylpropadienylidene, and 3-methylpropadienylidene, respectively.

## 1 Introduction

**Figure 1:**
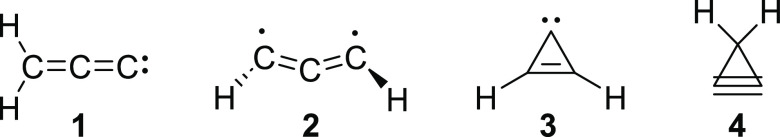
Isomers of the formula C_3_H_2_.

Allenylidenes are carbenes featuring a divalent carbon at the terminus of an allenic linkage. The parent member of this class of carbenes, the simplest cumulenyl carbene propadienylidene (**1**), belongs to the group of C_3_H_2_ isomers which also includes the simplest alkynyl carbene, propynylidene (**2**), that is better described as a triplet diradical; the smallest aromatic carbene, cyclopropenylidene (**3**); and the simplest carbocyclic alkyne, cyclopropyne (**4**) (Figure 1).^1^ In addition to being highly reactive species that challenge our understanding of structure and bonding, C_3_H_2_ isomers are of interest due to their apparent ubiquity throughout the galaxy, with **3** and its lowest energy isomer **1** residing in interstellar clouds (especially TMC-1) and planetary atmospheres.^2−8^ Isomers of C_3_H_2_ have also been identified in combustion flames, where they are implicated in the formation of polycyclic aromatic hydrocarbons and soot.^9−11^

**Scheme 1:**
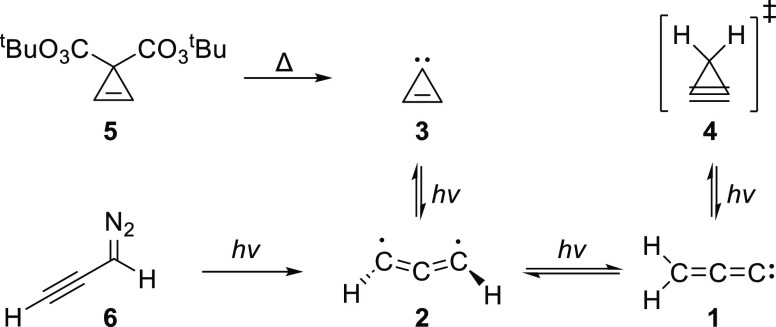
Several Means of Generating **1** and Other Interconvertible Species on the C_3_H_2_ Potential Energy Surface

Various experimental routes to **1** and its isomers have been described in the literature whereby light induces transitions between the readily interconvertible intermediates on the C_3_H_2_ potential energy surface (Scheme 1). The first reported generation and observation of **1** was by IR spectroscopy in a cryogenic argon matrix, where the perester **5** was used as a progenitor of **3**, which rearranged first into **2** and then into **1** upon further irradiation.^12^ A few years later, the first electronic spectrum of the parent allenylidene **1** was reported in a matrix isolation study that used diazo precursor **6** to generate triplet propynylidene **2**, which subsequently isomerized to **1** upon further photolysis (Scheme 1).^13^ Two critical studies using ^13^C-labeled **6** demonstrated that not only does **2** interconvert into **1** and **3,** but **1** also automerizes (as evident by scrambling of ^13^C labels), presumably via cyclopropyne **4** which was predicted to be a transition state rather than a discrete intermediate.^1,14^

The rotational spectrum of **1** was first reported by Vrtilek et al., who produced the intermediate by a direct current discharge in a gaseous mixture of acetylene, CO, and He.^7^ Chen and co-workers have also reported the photoionization mass spectrum and photoelectron spectrum of **1**, generated by pyrolysis of the dibromo alkyne **7**, and estimated that its singlet state lies about 40 kcal/mol below the triplet.^15^ A subsequent photodetachment study revised that gap to 29.7 kcal/mol.^16^
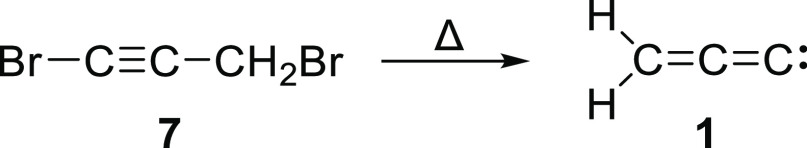


**Scheme 2:**
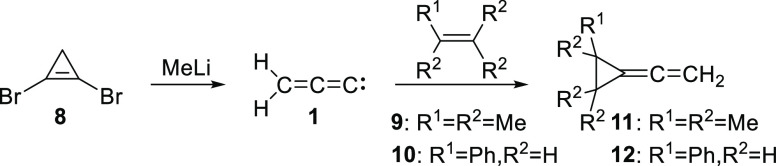
Generation of **1**, or a Carbenoid Equivalent, and its Trapping by Alkenes in Solution

The first report on the generation and trapping of **1** in solution described the addition of methyllithium to dibromocyclopropene (**8**) in the presence of tetramethylethylene (TME, **9**) or styrene (**10**), as shown in Scheme 2.^17^ The formation of cyclopropanes **11** and **12** was attributed to the interception of initially formed **1** by the alkene traps. It is not clear, however, if the cyclopropanes were formed by “free” **1** or a metal-coordinated carbenoid species serving as a surrogate for **1**.

**Scheme 3:**
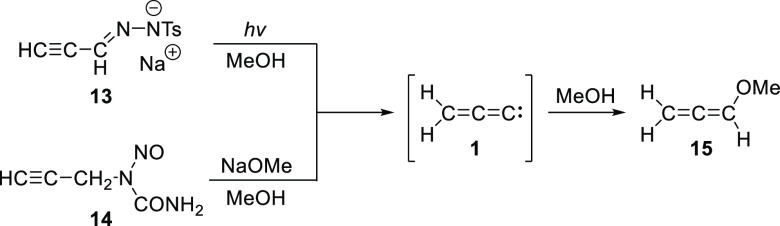
Speculated Generation of **1** from Nitrogenous Precursors and its Trapping by Methanol

It has been speculated that the pyrolysis of *N*-propynylaniline (PhNHCH_2_C≡CH) could produce **1**.^18^ The generation of **1** as a putative intermediate, either during photolysis of the tosylhydrazone salt **13** in methanol or treatment of the *N*-nitrosourea **14** with NaOMe/MeOH, has also been invoked, based on the formation of ether **15** (Scheme 3).^19^

**Scheme 4:**
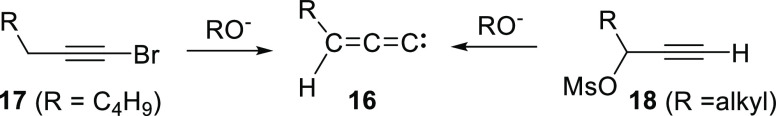
Generation of Monosubstituted Allenylidenes from Alkynyl Precursors Under Basic Conditions

**Scheme 5:**
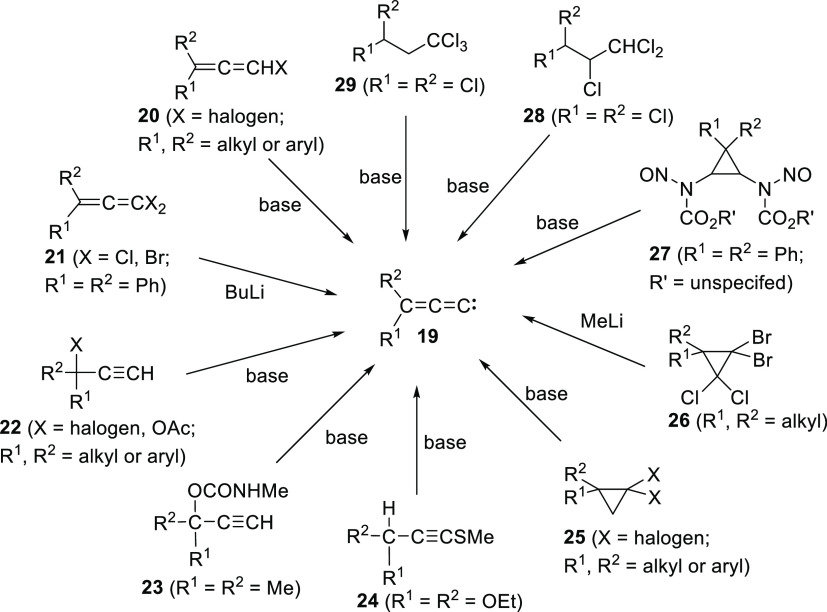
Means of Generating Disubstituted Allenylidenes Under Basic Conditions

Monosubstituted allenylidenes (**16**) have been generated by the action of alkoxide bases on 1-bromo-1-heptyne **17**(20) or propargyl mesylates **18**(21) (Scheme 4). Approaches to disubstituted allenylidenes (**19**) have involved the reaction of allenyl (**20** and **21**), alkynyl (**22**, **23**, and **24**), cyclopropyl (**25**, **26**, and **27**), and polychloroalkane derivatives (**28** and **29**) with strong bases (Scheme 5).^22−24^ The bases typically used are oxyanions, except in the case of **21** and **26**, which required alkyllithium reagents. Under these conditions, it is not always clear if **16** and **19** are produced as free carbenes or metal-coordinated carbenoids.

**Scheme 6:**
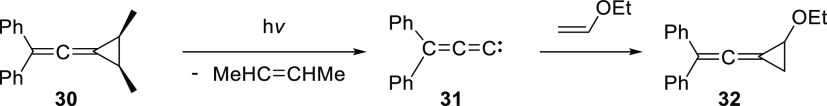
Photochemical Generation and Trapping of Diphenylvinylidenecarbene

Conspicuously absent from the literature is a general and broadly applicable photochemical route for the generation of **1** and other allenylidenes in solution. To the best of our knowledge, there is only one report which describes the generation of a substituted allenylidene, the diphenyl variant **31**, by photolysis of the cyclopropane derivative **30** in solvents (Scheme 6).^25^ When the photolysis is carried out in ethyl vinyl ether, the adduct **32** is formed in 25% yield. Carbene **31** can also be intercepted by cyclohexene albeit in even lower yield.

**Scheme 7:**
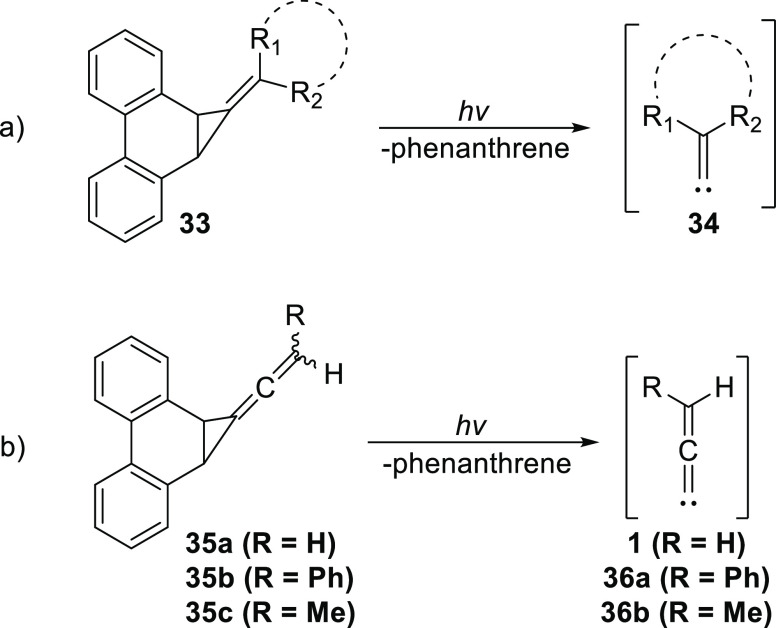
(a) Photochemical Generation of Exocyclic and Acyclic Alkylidene Carbenes **34** from Phenanthrene-Based Precursor **33**; (b) Photochemical Generation of Allenylidene Carbenes **1**, **36a**, and **36b** from their Respective Precursors

In an effort to develop alternative photochemical precursors to allenylidenes with a potential for general applicability, we modified our cyclopropanated phenanthrene system (**33**), from which our laboratory has previously generated several cyclic and acyclic alkylidenecarbenes (**34**),^26^ to incorporate an allene unit at the corner of the cyclopropane ring (**35a**, **b**, and **c**), as shown in Scheme 7. Herein, we describe our synthetic approach to **35a**, **b**, and **c**, their subsequent photolysis leading to the extrusion of **1**, **36a**, and **36b** respectively, and trapping studies. We also present the results of our computational work on the structures and energies of **1**, **36a**, and **36b** using modern methods and basis sets.

## 2 Results and Discussion

### 2.1 Synthesis of Phenanthrene-Based Precursors (**35a**, **b**, and **c**) to Carbenes

**Scheme 8:**
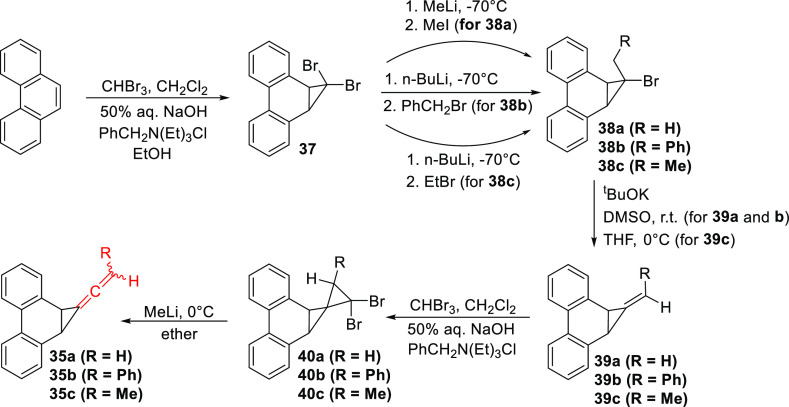
Synthesis of **35a**, **b**, and **c**, Phenanthrene-Based Precursors to **1**,**36a**, and **36b** Respectively

**Figure 2:**
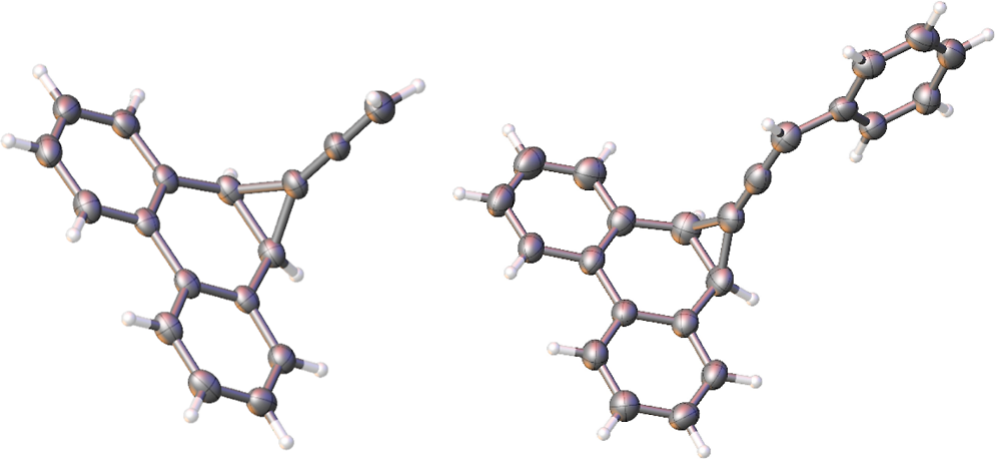
Single crystal X-ray structures of **35a** and *exo***-35b**. Thermal ellipsoids are shown at the 50% probability level.

The syntheses of 1-vinylidene-1a,9b-dihydro-1*H*-cyclopropa[*l*]phenanthrene (**35a**), 1-(2-phenylvinylidene)-1a,9b-dihydro-1*H*-cyclopropa[*l*]phenanthrene (**35b**), and 1-(prop-1-en-1-ylidene)-1a,9b-dihydro-1*H*-cyclopropa[*l*]phenanthrene (**35c**), photochemical precursors to **1**, **36a**, and **36b,** respectively, were accomplished as shown in Scheme 8. First, dibromocarbene was added to phenanthrene under phase-transfer catalysis conditions to afford the dibromocyclopropane **37**, using an improved procedure previously developed in our laboratory.^27^ Reaction of **37** with alkyllithiums at low temperature, followed by addition of the appropriate alkyl halide, gave **38a**, **b**, and **c**.^28^ Elimination of HBr from **38a** and **b** using KO^t^Bu in DMSO afforded the alkenes **39a** and **b,** respectively, while KO^t^Bu at 0 °C in THF was used for the conversion of **38c** to **39c**. Another addition of dibromocarbene to **39a**, **b**, and **c**(28) gave the corresponding dibromospiropentanes **40a**, **b**, and **c**. Careful addition of MeLi to **40a**, **b**, and **c** in ether at 0 °C yielded the desired precursors, **35a**, **b**, and **c** respectively. The allene **35b** was obtained as a mixture of *exo* and *endo* isomers in a 1:0.78 ratio, while the same isomers of **35c** were obtained in a 0.65:1 mixture. The single-crystal X-ray structures of **35a** and *exo*-**35b** are shown below in Figure 2, and their salient features are presented in the Supporting Information. The X-ray structures of **38a**, **39a**, **40a**, and **40b** are also provided in the Supporting Information.

### 2.2 Photolysis Experiments

**Scheme 9:**
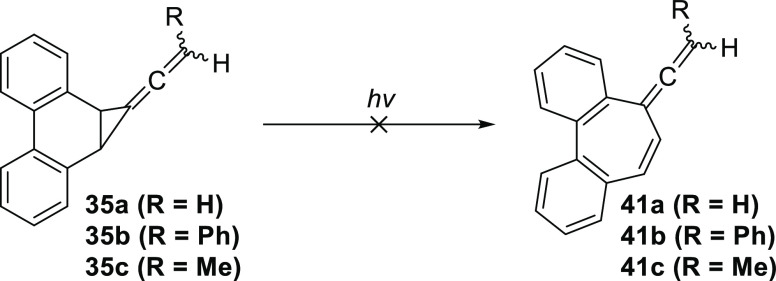
Rearrangement of **35a**, **b**, and **c** into the Respective Seven-Membered Systems **41a**, **b**, and **c** was Not Observed

Photolyses of **35a** and **b** were performed in olefin traps TME and 1,2-dihydronaphthalene (DHN). While we observed a product attributable to the adduct of **1** with TME, its low boiling point made isolation and characterization difficult; likewise, while **36a** did trap in DHN, it gave two isomers which proved difficult to separate and characterize. Therefore, herein we report in detail only the trapping of **1** in DHN and **36a** in TME. Photolysis of **35c** was performed only in 1,1-diphenylethylene (DPE). In stark contrast to the previous observations with related methylenecyclopropanes,^29−31^ none of the three precursors showed signs of rearrangement to seven-membered ring systems **41a**, **b**, and **c** (Scheme 9).

#### 2.2.1 Photolysis of **35a** in DHN and *m*-Xylene

**Scheme 10:**
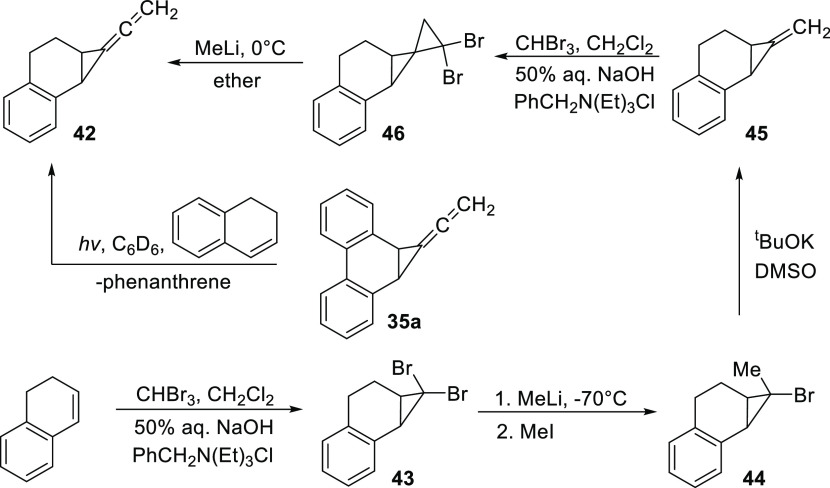
Generation of **42** from Precursor **35a** and its Independent Synthesis

Photolysis of **35a** in DHN, *m*-xylene (internal standard), and benzene-*d6* afforded the allene adduct **42** in 35% yield as determined by ^1^H NMR spectroscopy. The identity of **42** was confirmed by the independent synthesis of an authentic sample, as shown in Scheme 10. The first step involved the phase-transfer catalyzed addition of dibromocarbene to DHN, giving dibromocyclopropane **43**, a known compound.^32^ Addition of MeLi to **43** and subsequent reaction with iodomethane gave alkyl halide **44**. Dehydrobromination of **44** with KO^t^Bu in DMSO gave alkene **45**, to which dibromocarbene was added, leading to the spiropentane **46**. Finally, the addition of MeLi to ethereal **46** at ice temperature afforded the desired allene **42**. GC–MS and NMR analysis of the authentic sample of **42** indicated that it was identical to the product of photolysis, and IR showed the characteristic allene stretch.

#### 2.2.2 Photolysis of **35b** in TME

**Scheme 11:**
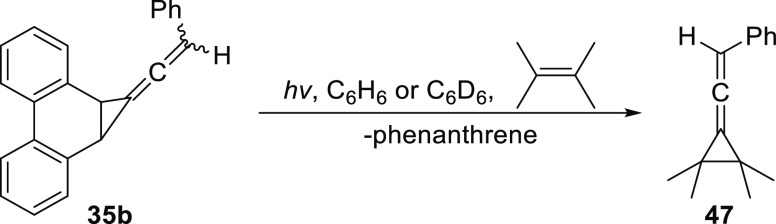
Cyclopropane Adduct **47**

Photolysis of **35b** in benzene with a significant excess of TME afforded the allenylcyclopropane adduct **47** (Scheme 11). Product **47** was isolated by column chromatography in 43% yield. The characterization of **47** was performed using the sample isolated from this photolysis, which showed the expected peaks in the ^1^H and ^13^C NMR and the characteristic allene stretch in the IR.

#### 2.2.3 Photolysis of **35b** in TME and Benzodioxole

Photolysis of **35b** in TME, benzodioxole (internal standard), and benzene-*d6* gave allene **47** in 33% yield, as determined by ^1^H NMR spectroscopy. The identity of **47** was confirmed by comparison to the product obtained from the photolysis (Section 2.2.2) above.

#### 2.2.4 Photolysis of **35c** in DPE and *m*-Xylene

**Scheme 12:**
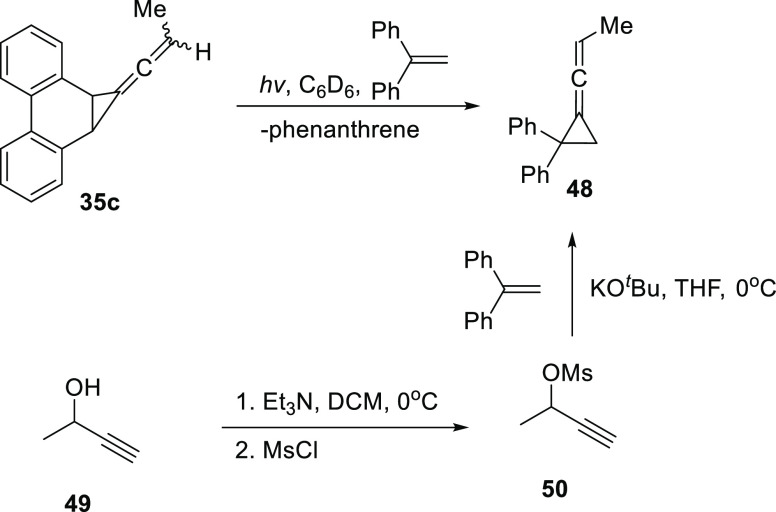
Photolysis of **35c** in the Presence of DPE Afforded the Cyclopropane Adduct **48**, which was Confirmed via Preparation of an Authentic Sample

Photolysis of **35c** in DPE, *m*-xylene (internal standard), and benzene-*d6* afforded the allene **48** in 66% yield, as determined by NMR. The identity of **48** was confirmed by synthesis of an authentic sample, following a known procedure,^33^ as shown in Scheme 12. First, the propargyl alcohol **49** was converted into the mesylate **50**, which was then reacted with KO^t^Bu in the presence of DPE to afford the desired adduct **48**. Spectroscopic analysis of **48** showed that it was identical to the product of photolysis.

### 2.3 Computational Results

We conducted quantum chemical calculations investigating the singlet–triplet gap for both allenylidenes. Single-point DLPNO–CCSD(T)^34,35^ calculations were performed on geometries optimized using four different methods—double hybrid density functional theory (B2PLYP),^36^ hybrid density functional theory (both PBE0^37−39^ and B3LYP^40,41^), and range-separated hybrid density functional theory (ωB97x-D3BJ), using the def2-TZVP basis set in all cases.^42^ Zero-point vibrational energy (ZPVE) corrections were applied to all energies.

The computations unanimously determined that **1** is more stable in its singlet ground state by ∼29 kcal/mol, with **36a** and **b** likewise having singlet states ∼30 and ∼33 kcal/mol more stable than their triplets (Table 1). Evidently, the substitutions on **36a** and **b** did little to affect the character of the carbenes, with all species having remarkably similar singlet–triplet gaps. Perhaps this similarity may be attributed to the orthogonal relationship between the two π systems in the allene moiety, which prevents substituents at one terminus from exerting a major influence on the carbene center at the other terminus. An overwhelming favorability for the singlet ground state is noted in the literature on both alkylidene carbenes and allenylidene carbenes,^24^ so the calculated singlet favorability of the present species is expected. Furthermore, experimental^16^ and computational^43^ singlet–triplet gaps for **1** (29.7 and 30 kcal/mol respectively) are in very good agreement with our computations. The singlet–triplet gaps for **36a** and **36b** have not been previously reported to our knowledge.

**Table 1:** Relative Single Point Energies (kcal/mol), Including ZPVE Corrections, for Singlet and Triplet **1**, **36a**, and **36b**^a^

computational level	**1** (singlet)	**1** (triplet)	**36a** (singlet)	**36a** (triplet)	36b (singlet)	**36b** (triplet)
DLPNO–CCSD(T)/def2-TZVP//B2PLYP/def2-TZVP	0 (0.02)	29.19 (0.03)	0 (0.01)	30.33 (0.02)	0 (0.02)	33.14 (0.03)
DLPNO–CCSD(T)/def2-TZVP//B3LYP/def2-TZVP	0 (0.02)	29.13 (0.03)	0 (0.01)	30.24 (0.02)	0 (0.02)	33.09 (0.03)
DLPNO–CCSD(T)/def2-TZVP//PBE0/def2-TZVP	0 (0.02)	28.99 (0.03)	0 (0.01)	29.83 (0.02)	0 (0.02)	33.00 (0.03)
DLPNO–CCSD(T)/def2-TZVP//ωB97x-D3BJ/def2-TZVP	0 (0.02)	28.81 (0.03)	0 (0.01)	29.66 (0.02)	0 (0.02)	32.82 (0.03)

a T_1_ diagnostics are presented in parentheses.

Singlet and triplet geometries of all intermediates are similar, with the major difference being in the length of the allenic bonds. Specifically, the β,γ-double bond in the singlet allenylidenes measured 1.28 Å, while the α,β-double bond was slightly longer at 1.33 Å. The same bonds in the triplet allenylidenes differed slightly, at 1.23 and 1.38 Å. The angles at the central carbon in the allene moiety were all nearly 180°, apart from triplets **36a** and **b**, which had an angle of ∼178° and thus adopted a slight bend.

## 3 Conclusions

Herein, we report what is, to our knowledge, the first photochemical generation of the parent allenylidene (**1**) in solution. The possibility of this approach to other substituted allenylidenes is supported by the generation of the phenyl and methyl analogues (**36a** and **b**). A straightforward synthetic sequence provided access to the photochemical precursors 1-vinylidene-1a,9b-dihydro-1*H*-cyclopropa[*l*]phenanthrene (**35a**), 1-(2-phenylvinylidene)-1a,9b-dihydro-1*H*-cyclopropa[*l*]phenanthrene (**35b**), and 1-(prop-1-en-1-ylidene)-1a,9b-dihydro-1*H*-cyclopropa[*l*]phenanthrene (**35c**). Photolysis of **35a**, **b**, and **c** in olefin traps with benzene as the solvent afforded the expected cyclopropane adducts. Quantum chemical calculations with geometries optimized by four different methods unanimously placed singlets **1**, **36a**, and **36b** ∼29, ∼30, and ∼33 kcal/mol lower in energy than their triplets, respectively. Given the success in generating these two allenylidenes photochemically in solution, we plan to use precursors **35a**, **b**, and **c** in time-resolved laser flash photolysis experiments in the future, which would be the first time that allenylidenes have been studied directly in this fashion.

## 4 Experimental Methods

### 4.1 General Experimental Procedures

Tetrahydrofuran and diethyl ether were degassed by purging with nitrogen and dried by passage through two activated alumina columns (2 ft × 4 in). Other solvents and reagents were used as obtained from commercial sources. Caution should be exercised while working with alkyllithium reagents. Previously, we have reported procedures for preparing 1,1-dibromo-1a,9b-dihydro-1*H*-cyclopropa[*l*]phenanthrene (**37**)^27^ and 1-benzylidene-1a,9b-dihydro-1*H*-cyclopropa[*l*]phenanthrene (**39b**).^28^ Medium pressure flash chromatography was performed on an automated system using prepacked silica gel columns (70–230 mesh) with the indicated eluents. Proton (^1^H) and proton-decoupled carbon (^13^C {^1^H}) NMR spectra were recorded in CDCl_3_ or C_6_D_6_ at 500 and 126 MHz, respectively. The chemical shifts are reported in δ ppm with tetramethylsilane set to 0 ppm. Infrared spectra (resolution 0.4 cm^–1^) were acquired on samples with an FTIR instrument equipped with an attenuated total reflectance accessory and were processed with SpectraGryph.^44^ GC/MS data were obtained with a capillary gas chromatograph interfaced with a quadrupole, triple-axis mass selective detector operating in the electron impact mode. High resolution mass spectra were obtained on a proton transfer reaction mass spectrometer (PTRMS). High resolution mass spectra were acquired either on a proton-transfer-reaction time-of-flight mass spectrometer (PTR-TOF-MS, Ionicon Analytik, Innsbruck, Austria) or an LC-TOF-MS system. Melting points are uncorrected. Single crystals suitable for X-ray crystallography were either obtained by slow evaporation of the hexanes from column fractions or from vapor diffusion using a binary solvent system (DCM/pentanes).

Photolyses were performed at ambient temperature in benzene in a long-necked quartz cuvette or in benzene-*d6* in a quartz NMR tube and were monitored using GC–MS and NMR. A medium pressure 200 W Hg–Xe arc lamp (Newport Model #6291), equipped with a 280–400 nm dichroic, was used for photolysis with the sample placed approximately 30 cm from the light source. Alternatively, a Rayonet reactor equipped with eight 8 W 254 nm lamps and eight 8 W 300 nm lamps were used for photolyses. Caution should be exercised while working with high powered ultraviolet radiation.

Computational software used in calculations as well as X-ray crystallography equipment and procedures have been recently described.^31^

#### 4.1.1 Exo-1-bromo-1-methyl-1a,9b-dihydrocyclopropa[*l*]phenanthrene **(38a)**

The dibromo derivative **37** (10.66 g, 30.5 mmol) was dissolved in 250 mL of THF in a 500 mL three-necked flask under argon and stirred with a magnetic stir bar. The solution was cooled to −70 °C in a bath of acetone and dry ice. Once cooled, methyllithium (22.5 mL, 1.6 M in diethyl ether, 36 mmol) was slowly added over 60 minutes. After 30 minutes of stirring at this temperature, methyl iodide (5 mL, 80 mmol) was added in excess. The solution was allowed to stir at −70 °C for another hour, after which it was removed from the ice bath and allowed to stir overnight. The reaction was then quenched with H_2_O (60 mL), and the aqueous layer was extracted with CH_2_Cl_2_ (3 × 50 mL). The combined organic layers were washed with H_2_O (2 × 50 mL) and brine (3 × 50 mL). **38a** was isolated as a white solid using flash-column chromatography with hexanes as eluent. The yield was 6.51 g (76%); mp: 109–112 °C. ^1^H NMR (500 MHz, CDCl_3_): δ 7.95 (d, *J* = 7.6 Hz, 2H), 7.47 (d, *J* = 7.2 Hz, 2H), 7.30 (m, 4H), 3.19 (s, 2H), 1.23 (s, 3H). ^13^C{^1^H} NMR (126 MHz, CDCl_3_): δ 131.0, 130.8, 130.1, 128.1, 127.3, 122.9, 34.7, 33.9, 19.1. FTIR: ν 3058, 3024, 2972, 2926, 1585, 1561, 1486, 1445 cm^–1^. HRMS (CI) *m*/*z*: [M + H]^+^ calcd for C_16_H_14_Br, 285.0279; found, 285.0275.

#### 4.1.2 1-Methylene-1a,9b-dihydrocyclopropa[*l*]phenanthrene **(39a)**

A solution of the alkyl halide **38a** (3.02 g, 10.73 mmol) in 75 mL of DMSO was stirred with a magnetic stir bar in a 250 mL three-necked flask under argon at room temperature To this solution was added excess potassium *tert*-butoxide (3.05 g, 27.16 mmol). The solution was stirred for 1 day, after which it was quenched with H_2_O (100 mL). The aqueous layer was extracted with diethyl ether (4 × 50 mL), and the combined organic layers were washed with H_2_O (2 × 100 mL) and brine (2 × 50 mL). **39a** was isolated as a white solid using flash-column chromatography with hexanes as eluent. The yield was 1.77 g (81%); mp: 122–124 °C. ^1^H NMR (500 MHz, CDCl_3_): δ 7.97 (d, *J* = 5.0 Hz, 2H), 7.39 (d, *J* = 4.1 Hz, 2H), 7.26 (dd, *J* = 8.5, 4.3 Hz, 4H), 5.36 (s, 2H), 3.16 (s, 2H). ^13^C{^1^H} NMR (126 MHz, CDCl_3_): δ 134.3, 132.8, 129.0, 128.9, 127.9, 126.3, 123.3, 102.6, 22.3. FTIR: ν 3063, 3030, 2986, 1485, 1439 cm^–1^. HRMS (CI) *m*/*z*: [M + H]^+^ calcd for C_16_H_13_, 205.1017; found, 205.1008.

#### 4.1.3 2,2-Dibromo-1a′,9b′-dihydrospiro[cyclopropane-1,1′-cyclopropa[*l*]phenanthrene] **(40a)**

The alkene **39a** (0.98 g, 4.79 mmol), benzyltriethylammonium chloride (0.074 g, 0.325 mmol), CHBr_3_ (1.1 mL, 3.2 g, 13.4 mmol), and CH_2_Cl_2_ (12 mL) were added to a 50 mL round-bottom flask and stirred with a magnetic stir bar at room temperature. Then, 50% aq NaOH (20 mL) was added over 10 minutes to the reaction mixture. The flask was loosely capped, and the mixture was allowed to stir for 3 days. The mixture was quenched with H_2_O (15 mL), the aqueous layer extracted with CH_2_Cl_2_ (3 × 20 mL), and the combined organic layers washed with H_2_O (2 × 20 mL) and brine (3 × 20 mL). **40a** was isolated as a white solid using flash-column chromatography with hexanes as eluent. The yield was 1.08 g (61%); mp: 186–187 °C. ^1^H NMR (500 MHz, CDCl_3_): δ 8.03 (d, *J* = 7.9 Hz, 2H), 7.40–7.30 (m, 4H), 7.29 (d, *J* = 7.3 Hz, 2H), 3.29 (s, 2H), 1.32 (s, 2H). ^13^C{^1^H} NMR (126 MHz, CDCl_3_): δ 132.1, 130.0, 129.3, 127.9, 127.1, 123.4, 35.2, 30.2, 29.1, 25.8. FTIR: ν 3068, 3011, 1485, 1439 cm^–1^.

#### 4.1.4 1-Vinylidene-1a,9b-dihydro-1*H*-cyclopropa[*l*]phenanthrene **(35a)**

**40a** (186 mg, 0.499 mmol) was dissolved in diethyl ether (60 mL) in a 100 mL three-necked flask and stirred with a magnetic stir bar under argon. The vessel was cooled to and maintained at 0 °C, and methyllithium (0.500 mL, 1.6 M in diethyl ether, 0.800 mmol) was very slowly added over 20 minutes. The mixture was allowed to stir for 30 more minutes, and then quenched with ice water (50 mL). The aqueous layer was extracted with CH_2_Cl_2_ (3 × 25 mL), and the combined organic layers were washed with brine (2 × 25 mL) and dried over sodium sulfate. **35a** was isolated as a white solid using flash-column chromatography with hexanes as eluent. The yield was 59 mg (55%); mp: decomposes at 127 °C. ^1^H NMR (500 MHz, CDCl_3_): δ 8.01 (d, *J* = 7.2 Hz, 2H), 7.45 (d, *J* = 6.3 Hz, 2H), 7.30 (dt, *J* = 11.6, 6.1 Hz, 4H), 4.93–4.77 (m, 1H), 4.75–4.62 (m, 1H), 3.59 (s, 2H). ^13^C{^1^H} NMR (126 MHz, CDCl_3_): δ 190.5, 132.5, 129.5, 129.3, 128.1, 126.9, 123.4, 79.2, 78.5, 26.9. FTIR: ν 3070, 3006, 2921, 2008, 1916, 1487, 1439 cm^–1^. HRMS (CI) *m*/*z*: [M + H]^+^ calcd [M + H]^+^ for C_17_H_13_, 217.1017; found, 217.1011.

#### 4.1.5 1,1-Dibromo-1a,2,3,7b-tetrahydro-1*H*-cyclopropa[*a*]naphthalene **(43)**

Though this compound is reported previously in the literature,^32^ we used our own procedure. Thus, 1,2-dihydronaphthalene (3.50 mL, 3.49 g, 26.8 mmol), bromoform (3.50 mL, 10.1 g, 40.0 mmol), benzyltriethylammonium chloride (150 mg, 0.65 mmol), and CH_2_Cl_2_ (40 mL) were added to a 250 mL Erlenmeyer flask and stirred with a magnetic stir bar under ambient conditions. 50% NaOH (30 mL) was added over the course of 5 minutes, and the reaction was allowed to stir overnight. The reaction was quenched with H_2_O, the aqueous layer extracted with CH_2_Cl_2_ (3 × 50 mL), and the combined organic layers were washed with brine (1 × 50 mL) and dried over sodium sulfate. **43** was isolated as light-yellow oil by flash-column chromatography using hexanes as eluent. The yield was 4.36 g (55%). Characterization data are reported in the literature.^32^

#### 4.1.6 Exo-1-bromo-1-methyl-1a,2,3,7b-tetrahydro-1*H*-cyclopropa[*a*]naphthalene **(44)**

**43** (323 mg, 1.08 mmol) was dissolved in 20 mL THF in a 100 mL three-necked flask and stirred with a magnetic stir bar under argon. The mixture was cooled to −70 °C in a bath of acetone and dry ice. Then, methyllithium (1.20 mL, 1.6 M in diethyl ether, 1.92 mmol) was added over 10 minutes. The bright yellow reaction was allowed to stir for 15 minutes, and an excess of methyl iodide (0.75 mL, 1.71 g, 12.0 mmol) was added. The reaction was allowed to slowly warm to room temperature, quenched with H_2_O, and the aqueous layer extracted with CH_2_Cl_2_ (3 × 15 mL). The combined organic layers were washed with brine (1 × 30 mL) and dried over sodium sulfate. **44** was isolated as a light-yellow oil by flash-column chromatography using hexanes as eluent. The yield was 168 mg (66%). ^1^H NMR (500 MHz, CDCl_3_): δ 7.35 (d, *J* = 7.4 Hz, 1H), 7.21–7.16 (m, 1H), 7.13 (td, *J* = 7.4, 1.5 Hz, 1H), 7.06 (d, *J* = 7.4 Hz, 1H), 2.72 (ddd, *J* = 16.0, 10.2, 6.1 Hz, 1H), 2.53 (d, *J* = 10.1 Hz, 1H), 2.43 (dt, *J* = 15.6, 5.3 Hz, 1H), 2.22 (ddt, *J* = 14.2, 8.6, 5.5 Hz, 1H), 2.15–2.07 (m, 1H), 1.69 (ddq, *J* = 15.3, 10.2, 5.2 Hz, 1H), 1.42 (s, 3H). ^13^C{^1^H} NMR (126 MHz, CDCl_3_): δ 137.2, 132.5, 130.5, 128.4, 126.5, 126.4, 38.1, 27.9, 27.7, 26.4, 21.0 (2 carbon resonances), 19.5. FTIR: ν 3062, 3019, 2935, 2859, 1489, 1455 cm^–1^. HRMS (CI) *m*/*z*: [M + H]^+^ calcd for C_12_H_14_Br, 239.0257; found, 239.0257.

#### 4.1.7 1-Methylene-1a,2,3,7b-tetrahydro-1*H*-cyclopropa[*a*]naphthalene **(45)**

**44** (168 mg, 0.712 mmol) was dissolved in 10 mL of DMSO in a 50 mL round-bottom flask and stirred with a magnetic stir bar under argon at room temperature To this solution was added excess potassium *tert*-butoxide (200 mg, 1.78 mmol). The reaction mixture was allowed to stir for 1.5 hours, and then quenched with H_2_O. The aqueous layer was extracted with diethyl ether (4 × 30 mL), and the combined organic layers were washed with brine (1 × 50 mL) and dried over sodium sulfate. **45** was isolated as light-yellow oil by flash-column chromatography using hexanes as eluent. The yield was 66 mg (59%). ^1^H NMR (500 MHz, CDCl_3_): δ 7.29 (dd, *J* = 7.4, 1.5 Hz, 1H), 7.17 (tt, *J* = 7.4, 1.3 Hz, 1H), 7.12 (td, *J* = 7.4, 1.5 Hz, 1H), 7.04 (d, *J* = 7.2 Hz, 1H), 5.51–5.48 (m, 1H), 5.48–5.42 (m, 1H), 2.67–2.55 (m, 2H), 2.48–2.36 (m, 1H), 2.22 (ddq, *J* = 9.1, 3.8, 1.9 Hz, 1H), 2.13 (ddt, *J* = 12.8, 5.0, 2.3 Hz, 1H), 1.48 (tt, *J* = 13.2, 4.0 Hz, 1H). ^13^C{^1^H} NMR (126 MHz, CDCl_3_): δ 136.7, 134.6, 134.5, 128.3, 128.0, 125.9, 125.0, 105.3, 26.2, 20.1, 18.3, 17.9. FTIR: ν 3064, 3017, 2986, 2923, 2854, 1602, 1491, 1456 cm^–1^. HRMS (CI) *m*/*z*: [M + H]^+^ calcd for C_12_H_13_, 157.1017; found, 157.1032.

#### 4.1.8 2,2-Dibromo-1a′,2′,3′,7b′-tetrahydrospiro[cyclopropane-1,1′-cyclopropa[*a*]naphthalene] **(46)**

**45** (59 mg, 0.378 mmol), bromoform (0.25 mL, 0.57 g, 2.26 mmol), benzyltriethylammonium chloride (40 mg, 0.176 mmol), and CH_2_Cl_2_ (10 mL) were added to a 50 mL Erlenmeyer flask and stirred with a magnetic stir bar under ambient conditions. 50% NaOH (5 mL) was added, and the reaction was allowed to stir overnight. The reaction was quenched with H_2_O, the aqueous layer was extracted with CH_2_Cl_2_ (3 × 15 mL), and the combined organic layers were washed with brine (1 × 15 mL) and dried over sodium sulfate. **46** was isolated as light-yellow oil by flash-column chromatography using hexanes as eluent. The yield was 55 mg (45%). ^1^H NMR (500 MHz, CDCl_3_): δ 7.26–7.22 (m, 1H), 7.19–7.12 (m, 2H), 7.09–7.03 (m, 1H), 2.70–2.60 (m, 2H), 2.48 (ddd, *J* = 15.5, 13.4, 6.2 Hz, 1H), 2.31 (dt, *J* = 8.5, 2.6 Hz, 1H), 2.09 (ddt, *J* = 13.7, 6.3, 2.2 Hz, 1H), 1.92 (d, *J* = 6.7 Hz, 1H), 1.82 (dd, *J* = 6.8, 0.8 Hz, 1H), 1.76 (tdd, *J* = 13.6, 5.6, 3.2 Hz, 1H). ^13^C{^1^H} NMR (126 MHz, CDCl_3_): δ 134.5 (2 carbon resonances), 128.9, 128.6, 126.2, 126.1, 35.1, 28.9, 28.0, 27.8, 26.1, 24.9, 18.7. FTIR: ν 3060, 3018, 2923, 2852, 1580, 1533, 1490, 1452, 1408 cm^–1^. HRMS (CI) *m*/*z*: [M + H]^+^ calcd for C_13_H_13_Br_2_, 328.9364; found, 328.9349.

#### 4.1.9 1-Vinylidene-1a,2,3,7b-tetrahydro-1*H*-cyclopropa[*a*]naphthalene **(42)**

**46** (55 mg, 0.169 mmol) was dissolved in diethyl ether (15 mL) in a 100 mL three-necked flask under argon with stirring. The flask was cooled between 3 and 5 °C, where it was carefully maintained for the rest of the reaction. Then, methyllithium (0.200 mL, 1.6 M in diethyl ether, 0.320 mmol) was very slowly added to the reaction over 10 minutes. The yellow solution was allowed to stir for 5 more minutes, after which it was quenched with addition of ice water (30 mL). The aqueous layer was extracted with diethyl ether, (4 × 10 mL) and the combined organic layers were washed with brine (1 × 30 mL) and dried over sodium sulfate. **42** was isolated as clear oil by flash-column chromatography using hexanes as eluent. The yield was 14 mg (49%). ^1^H NMR (500 MHz, CDCl3): δ 7.29 (dd, *J* = 7.2, 1.6 Hz, 1H), 7.17–7.11 (m, 2H), 7.06 (dt, *J* = 7.4, 1.2 Hz, 1H), 4.84 (dt, *J* = 9.9, 3.6 Hz, 1H), 4.74 (dt, *J* = 9.9, 3.7 Hz, 1H), 3.04 (dt, *J* = 7.6, 3.6 Hz, 1H), 2.65–2.60 (m, 2H), 2.59–2.50 (m, 1H), 2.24 (ddt, *J* = 13.1, 5.0, 2.3 Hz, 1H), 1.60 (tdd, *J* = 13.3, 4.7, 3.2 Hz, 1H). ^13^C{^1^H} NMR (126 MHz, CDCl_3_): δ 194.2, 135.3, 134.8, 128.4, 128.3, 126.1, 125.7, 79.6, 77.5, 26.0, 25.7, 23.1, 19.5. FTIR: ν 3061, 3017, 2998, 2924, 2853, 2009, 1579, 1490, 1453, 1433 cm^–1^. HRMS (CI) *m*/*z*: [M + H]^+^ calcd for C_13_H_13_, 169.1012; found, 169.1018.

#### 4.1.10 2,2-Dibromo-3-phenyl-1a′,9b′-dihydrospiro[cyclopropane-1,1′-cyclopropa[*l*]phenanthrene] **(40b)**

To a 125 mL Erlenmeyer flask under ambient conditions were added **39b**(28) (2.80 g, 10.0 mmol), benzyltriethylammonium chloride (0.126 g, 0.555 mmol), bromoform (5 mL, 14.5 g, 57.2 mmol), dichloromethane (30 mL), and a magnetic stirbar. Then, 15 mL of 50% NaOH was slowly added to the stirred reaction mixture, which turned brown and viscous. The reaction was allowed to continue stirring overnight, after which it was quenched with 100 mL H_2_O and extracted with CH_2_Cl_2_ (3 × 50 mL). The organic layer was washed with H_2_O (1 × 100 mL) and brine (1 × 100 mL), and dried over sodium sulfate. **40b** was isolated as a white solid using flash-column chromatography with hexanes as eluent. The yield was 2.59 g (58%); mp: 156–157 °C. ^1^H NMR (500 MHz, CDCl_3_): δ 7.96–7.90 (m, 1H), 7.87 (d, *J* = 7.9 Hz, 1H), 7.54 (d, *J* = 7.4 Hz, 1H), 7.45–7.40 (m, 1H), 7.34–7.28 (m, 3H), 7.27–7.21 (m, 1H), 7.03 (t, *J* = 7.4 Hz, 1H), 6.94 (t, *J* = 7.6 Hz, 2H), 6.66 (d, *J* = 7.6 Hz, 2H), 3.62 (d, *J* = 8.3 Hz, 1H), 3.25 (d, *J* = 8.3 Hz, 1H), 2.68 (s, 1H). ^13^C{^1^H} NMR (126 MHz, CDCl_3_): δ 134.3, 131.9, 131.8, 130.3, 129.9, 129.3 (2 C), 128.0, 127.9, 127.4, 127.3, 127.1, 126.9, 123.6, 123.5, 40.3, 40.0, 37.2, 31.7, 31.3. FTIR: ν 3060, 3028, 1601, 1491, 1435 cm^–1^.

#### 4.1.11 Exo- and Endo-1-(2-phenylvinylidene)-1a,9b-dihydro-1*H*-cyclopropa[*l*]phenanthrene **(35b)**

**40b** (0.467 g, 1.04 mmol) was dissolved in diethyl ether (50 mL) in a 100 mL three-necked flask under argon with stirring. The flask was cooled to between 3 and 5 °C, where it was carefully maintained for the rest of the reaction. Then, excess methyllithium (1 mL, 1.6 M in diethyl ether, 1.6 mmol) was very slowly added to the reaction over 10 minutes. The reaction was allowed to stir for 30 minutes, after which it was quenched with H_2_O (50 mL). The aqueous layer was then extracted with diethyl ether (2 × 50 mL), and the combined organic layers were washed with H_2_O (2 × 50 mL) and brine (1 × 50 mL), and was dried over sodium sulfate. **35b**, a white solid, was isolated as a mixture of diastereomers in a 0.78:1.0 (endo/exo) ratio by flash-column chromatography using hexanes as eluent. The yield was 0.109 g (36%); mp: decomposes at 111 °C. ^1^H NMR (500 MHz, CDCl_3_): δ 8.07 (d, *J* = 7.9 Hz, 2H), 8.03 (d, *J* = 7.2 Hz, 1.56H), 7.48–7.42 (m, 3.56H), 7.36–7.27 (m, 10.68H), 7.07–6.95 (m, 3.56H), 6.86–6.76 (m, 1.78H), 6.27 (t, *J* = 3.3 Hz, 1H), 6.14 (t, *J* = 3.4 Hz, 0.78H), 3.77 (d, *J* = 3.5 Hz, 1.56H), 3.70 (d, *J* = 3.3 Hz, 2H). ^13^C{^1^H} NMR (126 MHz, CDCl_3_): δ 186.6, 186.1, 135.6, 135.4, 132.9, 132.3, 129.6, 129.5, 129.3 (2 C), 128.7, 128.5, 128.3, 127.1 (2 carbon resonances), 126.7 (2 carbon resonances), 126.5, 126.4 (2 carbon resonances), 123.5 (2 carbon resonances), 98.6, 98.2, 85.2, 83.5, 28.2, 27.7. FTIR: ν 3054, 3028, 2921, 1998, 1598, 1482, 1448 cm^–1^.

#### 4.1.12 (2-(2,2,3,3-Tetramethylcyclopropylidene)vinyl)benzene **(47)**

Isolated from Photolysis Section 4.1.19 (vide infra) as a viscous clear oil. The yield was 13 mg (43%). ^1^H NMR (500 MHz, CDCl_3_): δ 7.26–7.21 (m, 4H), 7.14–7.09 (m, 1H), 6.13 (s, 1H), 1.40 (s, 6H), 1.33 (s, 6H). ^13^C{^1^H} NMR (126 MHz, CDCl_3_): δ 185.9, 136.9, 128.5, 126.6, 125.9, 99.3, 95.7, 31.4, 22.0, 21.2. FTIR: ν 3061, 2989, 2950, 2919, 2866, 1988, 1601, 1491, 1457 cm^–1^. HRMS (CI) *m*/*z*: [M + H]^+^ calcd for C_15_H_19_, 199.1481; found, 199.1495.

#### 4.1.13 1-Bromo-1-ethyl-1a,9b-dihydro-1*H*-cyclopropa[*l*]phenanthrene (**38c**)

The dibromo derivative **37** (10.50 g, 30 mmol) was dissolved in THF (150 mL) in a 250 mL three-necked flask under argon and stirred with a magnetic stir bar. The solution was cooled to −70 °C, and *n*-butyllithium (13 mL, 2.5 M in hexanes, 32.5 mmol) was slowly added over 20 minutes. After 15 more minutes of stirring at this temperature, bromoethane (3.7 mL, 5.4 g, 49.9 mmol) was slowly added. The reaction was stirred at −70 °C for one more hour, and then it was warmed to room temperature and stirred overnight. The reaction was quenched with H_2_O (50 mL), and the aqueous layer was extracted with CH_2_Cl_2_ (3 × 50 mL). The combined organic layers were washed with brine (1 × 50 mL) and dried over sodium sulfate. **38c** was isolated as a waxy white solid using flash-column chromatography with hexanes as eluent. The yield was 5.24 g (58%); mp: 67–70 °C. ^1^H NMR (500 MHz, CDCl_3_): δ 7.95 (dd, *J* = 7.8, 1.4 Hz, 2H), 7.49 (dd, *J* = 7.2, 1.7 Hz, 2H), 7.34–7.27 (m, 4H), 3.22 (s, 2H), 1.31 (q, *J* = 7.1 Hz, 2H), 0.69 (t, *J* = 7.2 Hz, 3H). ^13^C{^1^H} NMR (126 MHz, CDCl_3_): δ 131.1, 130.8, 130.0, 128.0, 127.2, 122.8, 43.4, 34.0, 24.9, 10.8. FTIR: ν 3064, 3023, 2973, 2935, 2914, 2874, 1604, 1584, 1486, 1447, 1442 cm^–1^. HRMS (CI) *m*/*z*: [M + H]^+^ calcd for C_17_H_16_Br, 299.0435; found, 299.0420.

#### 4.1.14 1-Ethylidene-1a,9b-dihydro-1*H*-cyclopropa[*l*]phenanthrene (**39c**)

**38c** (300 mg, 0.993 mmol) was dissolved in THF (20 mL) in a 50 mL round-bottom flask with stirring under argon. The mixture was cooled to between 3 and 5 °C, and an excess of potassium *t*-butoxide (639 mg, 5.23 mmol) was added. The reaction stirred at this temperature for 4 hours, after which it was warmed to room temperature and stirred overnight. The mixture was then quenched with H_2_O, the aqueous layer extracted with CH_2_Cl_2_ (2 × 40 mL), and the combined organic layers washed with brine and dried over sodium sulfate. **39c** was isolated as a white solid using flash-column chromatography with hexanes as eluent. The yield was 158.2 mg (73%); mp: 100–103 °C. ^1^H NMR (500 MHz, CDCl_3_): δ 7.98–7.94 (m, 2H), 7.45–7.41 (m, 1H), 7.39–7.36 (m, 1H), 7.29–7.26 (m, 2H), 7.26–7.24 (m, 2H), 5.74 (tdt, *J* = 6.7, 4.4, 2.2 Hz, 1H), 3.13 (q, *J* = 1.9 Hz, 2H), 1.67 (dt, *J* = 6.6, 1.7 Hz, 3H). ^13^C{^1^H} NMR (126 MHz, CDCl_3_): δ 133.6, 133.4, 129.1 (2 carbon resonances), 128.9, 128.8, 127.8 (2 carbon resonances), 126.1, 126.0, 125.6, 123.3 (2 carbon resonances), 113.4, 22.4, 21.4, 16.1. FTIR: ν 3064, 3029, 2979, 2911, 2854, 1486 cm^–1^. HRMS (ESI) *m*/*z*: [M + H]^+^ calcd for C_17_H_15_, 219.1168; found, 219.1184.

#### 4.1.15 2,2-Dibromo-3-methyl-1a′,9b′-dihydrospiro[cyclopropane-1,1′-cyclopropa[*l*]phenanthrene] (**40c**)

**39c** (158.2 mg, 0.726 mmol), benzyltriethylammonium chloride (20 mg, 0.0878 mmol), excess bromoform (0.50 mL, 1.45 g, 5.74 mmol), and CH_2_Cl_2_ were added to a 50 mL Erlenmeyer flask with stirring under ambient conditions. Then, 50% NaOH (5 mL) was slowly added to the reaction. The mixture was allowed to stir overnight, and then quenched with H_2_O. The aqueous layer was extracted with CH_2_Cl_2_ (3 × 20 mL), and the combined organic layers were washed with brine (2 × 20 mL) and dried over sodium sulfate. **39c** was isolated first as viscous oil which, upon repeated cycles of trituration with CH_3_OH followed by drying, reluctantly formed a waxy white solid. The yield was 136 mg (48%); mp: 84–87 °C. ^1^H NMR (500 MHz, CDCl_3_): δ 8.05–8.00 (m, 2H), 7.40–7.28 (m, 5H), 7.25–7.20 (m, 1H), 3.24 (d, *J* = 8.1 Hz, 1H), 3.19 (d, *J* = 8.1 Hz, 1H), 1.44 (q, *J* = 6.3 Hz, 1H), 0.51 (dd, *J* = 6.3, 0.9 Hz, 3H). ^13^C{^1^H} NMR (126 MHz, CDCl_3_): δ 132.4, 131.6, 129.9 (2 carbon resonances), 129.6, 129.4, 127.9, 127.7, 127.0 (2 carbon resonances), 123.4, 123.3, 39.2, 38.0, 30.6, 30.2, 29.6, 13.1. FTIR: ν 3016, 2964, 2927, 1486 cm^–1^. HRMS (ESI) *m*/*z*: [M – Br]^+^ calcd for C_18_H_14_Br, 309.0279; found, 309.0275.

#### 4.1.16 1-(Prop-1-en-1-ylidene)-1a,9b-dihydro-1*H*-cyclopropa[*l*]phenanthrene (**35c**)

**40c** (288 mg, 0.742 mmol) was dissolved in diethyl ether (25 mL) in a 50 mL three-necked flask under argon with stirring. The solution was cooled to 3–5 °C, and excess methyllithium (1 mL, 1.6 M in diethyl ether, 1.6 mmol) was slowly added over the course of 5 minutes. The solution adopted a light-yellow hue and became cloudy, and it was allowed to stir at ice temperature for 10 more minutes. The reaction was quenched by the addition of H_2_O (20 mL), the aqueous layer was extracted with CH_2_Cl_2_ (3 × 10 mL), and the combined organic layers were washed with brine (1 × 10 mL) and dried over sodium sulfate. **35c** was isolated as a white solid using flash-column chromatography with hexanes as eluent. The yield was 88 mg (51%); mp: decomposed at 109 °C. ^1^H NMR (500 MHz, CDCl_3_): δ 8.02–7.97 (m, 2H), 7.45–7.38 (m, 2H), 7.33–7.26 (m, 4H), 5.27 (qt, *J* = 7.0, 3.4 Hz, 0.39H), 5.10 (qt, *J* = 7.1, 3.6 Hz, 0.60H), 3.52 (d, *J* = 3.6 Hz, 1.2H), 3.48 (d, *J* = 3.3 Hz, 0.78H), 1.75 (d, *J* = 7.1 Hz, 1.8H), 1.48 (d, *J* = 7.1 Hz, 1.2H). ^13^C{^1^H} NMR (126 MHz, CDCl_3_): δ 186.9, 186.8, 133.1, 132.9, 129.5, 129.4, 129.2, 128.0 (2 carbon resonances), 126.7, 126.6, 123.3, 90.5, 90.3, 80.8 (2 carbon resonances), 26.1, 25.9, 15.0 (2 carbon resonances). FTIR: ν 3064, 3029, 2961, 2908, 2849, 2011, 1486, 1434 cm^–1^. HRMS (ESI) *m*/*z*: [M + H]^+^ calcd for C_18_H_15_, 231.1168; found, 231.1178.

#### 4.1.17 (2-(Prop-1-en-1-ylidene)cyclopropane-1,1-diyl)dibenzene (**48**)

Preparation of an authentic sample of this compound was accomplished by adapting the procedure reported by Hori et al.^33^ 3-Butyn-2-ol (1 mL, 12.8 mmol) was dissolved in CH_2_Cl_2_ (10 mL) in a 50 mL two-necked flask under argon with stirring. The solution was cooled to 0 °C, triethylamine (2.7 mL, 19.2 mmol) was added, and methanesulfonyl chloride (1.2 mL, 15.4 mmol) was then added over 15 minutes. The reaction was allowed to stir at ice temperature for 2 hours, and it was quenched with 1 M HCl, extracted with CH_2_Cl_2_ (3 × 50 mL), washed with brine (1 × 50 mL), and dried over sodium sulfate. Solvent was removed by rotary evaporation, and the mesylate was used in the next step without purification. To a 100 mL two-necked flask was added THF (15 mL), potassium *tert*-butoxide (2.00 g, 16.4 mmol), and 1,1-diphenylethylene (3.5 mL, 18.9 mmol), and the solution was cooled to 0 °C. The mesylate dissolved in THF (30 mL) was added very slowly over 15 minutes, turning the reaction a dark brown. The mixture was allowed to stir for 30 minutes, and water (20 mL) was added to quench the reaction. The aqueous layer was extracted with CH_2_Cl_2_ (5 × 20 mL), washed with brine (2 × 20 mL), and dried over sodium sulfate. The crude product was purified by flash-column chromatography using hexanes as eluent to afford **7b** as light yellow oil. The final yield was 236 mg (8% yield over 2 steps). ^1^H NMR (500 MHz, CDCl_3_): δ 7.35–7.26 (m, 8H), 7.23–7.19 (m, 2H), 5.43 (qt, *J* = 7.2, 3.9 Hz, 1H), 2.25 (dd, *J* = 7.0, 3.9 Hz, 1H), 2.19 (dd, *J* = 7.1, 4.1 Hz, 1H), 1.82 (d, *J* = 7.0 Hz, 3H). ^13^C{^1^H} NMR (126 MHz, CDCl_3_): δ 190.3, 143.5, 143.1, 128.3, 128.3, 128.3, 128.1, 126.5, 126.5, 90.4, 86.7, 36.8, 24.4, 15.2. FTIR: ν 3057, 3026, 2911, 2014, 1601, 1493, 1445, 1402 cm^–1^. HRMS (ESI) *m*/*z*: [M + H]^+^ calcd for C_18_H_17_, 233.1325; found, 233.1345.

#### 4.1.18 Photolysis of **35a** in 1,2-Dihydronapthalene and *m*-Xylene

Precursor **35a** (2.2 mg, 0.010 mmol) was dissolved in 0.75 mL benzene-*d6* in a quartz NMR tube. 1,2-Dihydronapthalene (11.3 μL, 11.3 mg, 0.087 mmol) was added, followed by *m*-xylene (2 μL, 1.72 mg, 0.016 mmol) as an internal standard. The tube was placed in the Rayonet Reactor, and the progress of the photolysis was monitored every 30 minutes until all **35a** had photolyzed, or 4.5 hours. Yields of **42** (0.59 mg, 0.0035 mmol, 35%) and phenanthrene (1.5 mg, 0.0085 mmol, 85%) were thus determined. The photolysate was subsequently analyzed via GC–MS and compared to an authentic sample to verify identity.

#### 4.1.19 Photolysis of **35b** in TME

Precursor **35b** (45 mg, 0.154 mmol) was dissolved in 1 mL benzene and 1 mL TME in a long-necked quartz cuvette. The photolysis was performed using the HgXe arc lamp for 20 hours, and the photolysate after this time was assessed via GC–MS and MS to ensure that all **35b** had photolyzed. To isolate the trapped carbene **47**, the mixture was purified by flash column on silica using hexanes, giving **47** as clear oil. The yield was 13 mg (43%).

#### 4.1.20 Photolysis of **35b** in TME and Benzodioxole

Precursor **35b** (6.1 mg, 0.021 mmol) was dissolved in 0.5 mL of C_6_D_6_ in a quartz NMR tube, and 5.0 μL of 1,3-benzodioxole (5.3 mg, 0.043 mmol) was added to the tube as an internal standard. 8.2 μL of TME (5.8 mg, 0.069 mmol) was also added as the trapping agent. Photolysis with the HgXe arc lamp was monitored by NMR until all **35b** had reacted, or for 5 hours. Yields of **47** (1.4 mg, 0.0069 mmol, 33%) and phenanthrene (2.6 mg, 0.017 mmol, 81%) were thus determined.

#### 4.1.21 Photolysis of **35c** in 1,1-Diphenylethylene and *m*-Xylene

Precursor **35c** (3.2 mg, 0.014 mmol) was dissolved in 0.75 mL of C_6_D_6_ in a quartz NMR tube, 2.5 μL of *m*-xylene (2.2 mg, 0.021 mmol) was added as an internal standard, and 16 μL 1,1-diphenylethylene (16 mg, 0.087 mmol) was added as a trapping agent. Photolysis was performed using the HgXe arc lamp and was monitored every 30 minutes until all **35c** had reacted, or for 1 hour. A yield of **48** (2.1 mg, 0.0092 mmol, 66%) was thus determined.

## Data Availability

The data underlying this study are available in the published article and its Supporting Information.

## Supporting Information Available

Spectral data, X-ray data, Cartesian coordinates, and total energies of the relevant optimized structures (PDF)
