# Supplementary material for: Photochemical Generation of Allenylidenes from Cyclopropanated Phenanthrenes: An Experimental and Computational Study
Source: J Org Chem. 2024 May 29;89(11):7503–12. doi: 10.1021/acs.joc.4c00147 (PMC11165590; doi:10.1021/acs.joc.4c00147)
Supplement: Supplementary file 1 — jo4c00147_si_001.pdf [file jo4c00147_si_001.pdf]

ELECTRONIC SUPPORTING INFORMATION

Photochemical Generation of Allenylidenes from Cyclopropanated Phenanthrenes: An Experimental and Computational Study

*Alexander D. Roth, David R. Ramgren, Yuewei Wen, Megan S. Michie, and Dasan M. Thamattoor**

Department of Chemistry, Colby College, Waterville, ME 04901

dmthamat@colby.edu

Table of Contents

(1) Characterization data for *1-bromo-1-methyl-1a,9b-dihydro-1H-cyclopropa[l]phenanthrene* **(38a)**
- (a) GC-MS data……………………………………………………………....S5
- (b) ^{1}H NMR spectrum……………………………………………………..S6
- (c) ^{13}C NMR spectrum…………………………………………………......S7
- (d) FTIR spectrum…………………………………………………………....S8
- (e) Crystal structure and data.………………………………………………..S9

(2) Characterization data for *1-methylene-1a,9b-dihydro-1H-cyclopropa[l]phenanthrene* **(39a)**
- (a) GC-MS data……………………………………………………………....S10
- (b) ^{1}H NMR spectrum……………………………………………………..S11
- (c) ^{13}C NMR spectrum…………………………………………………......S12
- (d) FTIR spectrum…………………………………………………………… S13
- (e) Crystal structure and data.…………………………………………..……S14

(3) Characterization data for *2,2-dibromo-1a',9b'-dihydrospiro[cyclopropane-1,1'-cyclopropa[l]phenanthrene* **(40a)**
- (a) GC-MS data……………………………………………………………...S15
- (b) ^{1}H NMR spectrum………………………………………………………S16
- (c) ^{13}C NMR spectrum……………………………………………………....S17
- (d) FTIR spectrum…………………………………………………………..S18
- (e) Crystal structure and data….……………………………………………S19

(4) Characterization data for *1-vinylidene-1a,9b-dihydro-1H-cyclopropa[l]phenanthrene* **(35a)**
- (a) GC-MS data……………………………………………………………...S20
- (b) ^{1}H NMR spectrum………………………………………………………S21
- (c) ^{13}C NMR spectrum……………………………………………………....S22
- (d) FTIR spectrum…………………………………………………………..S23
- (e) Crystal structure…………………………………………………………S24

(5) Characterization data for *1-bromo-1-methyl-1a,2,3,7b-tetrahydro-1H-cyclopropa[a]naphthalene* **(44)**
- (a) GC-MS data……………………………………………………………...S25
- (b) ^{1}H NMR spectrum………………………………………………………S26
- (c) ^{13}C NMR spectrum……………………………………………………....S27

(d) FTIR spectrum…………………………………………………………………..S28
(6) Characterization data for *1-methylene-1a,2,3,7b-tetrahydro-1H-cyclopropa[a]naphthalene* **(45)**
(a) GC-MS data…………………………………………………………………...S29
(b) ^{1}H NMR spectrum…………………………………………………………S30
(c) ^{13}C NMR spectrum………………………………………………………....S31
(d) FTIR spectrum…………………………………………………………………..S32
(7) Characterization data for *2,2-dibromo-1a',2',3',7b'-tetrahydrospiro[cyclopropane-1,1'-cyclopropa[a]naphthalene]* **(46)**
(a) GC-MS data…………………………………………………………………...S33
(b) ^{1}H NMR spectrum…………………………………………………………S34
(c) ^{13}C NMR spectrum………………………………………………………....S35
(d) FTIR spectrum…………………………………………………………………..S36
(8) Characterization data for *1-vinylidene-1a,2,3,7b-tetrahydro-1H-cyclopropa[a]naphthalene* **(42)**
(a) GC-MS data…………………………………………………………………...S37
(b) ^{1}H NMR spectrum…………………………………………………………S38
(c) ^{1}H NMR spectrum in C_6D_6……………………………………………S39
(d) ^{13}C NMR spectrum………………………………………………………....S40
(e) FTIR spectrum…………………………………………………………………..S41
(9) Characterization data for *2,2-dibromo-3-phenyl-1a',9b'-dihydrospiro[cyclopropane-1,1'-cyclopropa[l]phenanthrene]* **(40b)**
(a) ^{1}H NMR spectrum…………………………………………………………S42
(b) ^{13}C NMR spectrum………………………………………………………....S43
(c) FTIR spectrum…………………………………………………………………..S44
(d) Crystal structure and data………………………………………………………S45
(10) Characterization data for *1-(2-phenylvinylidene)-1a,9b-dihydro-1H-cyclopropa[l]phenanthrene* **(35b)**
(a) ^{1}H NMR spectrum…………………………………………………………S46
(b) ^{13}C NMR spectrum………………………………………………………....S47
(c) FTIR spectrum…………………………………………………………………..S48
(d) Crystal structure and data………………………………………………………S49
(11) Characterization data for *(2-(2,2,3,3-tetramethylcyclopropylidene)vinyl)benzene* **(47)**
(a) GC-MS data…………………………………………………………………...S50
(b) ^{1}H NMR spectrum…………………………………………………………S51
(c) ^{1}H NMR spectrum in C_6D_6……………………………………………S52
(d) ^{13}C NMR spectrum………………………………………………………....S53
(e) FTIR spectrum…………………………………………………………………..S54
(12) Characterization data for *1-bromo-1-ethyl-1a,9b-dihydro-1H-cyclopropa[l]phenanthrene* ***(38c)***
(a) GC-MS data…………………………………………………………………...S55
(b) ^{1}H NMR spectrum…………………………………………………………S56
(c) ^{13}C NMR spectrum………………………………………………………....S57
(d) FTIR spectrum…………………………………………………………………..S58
(13) Characterization data for *1-ethylidene-1a,9b-dihydro-1H-cyclopropa[l]phenanthrene* ***(39c)***
(a) GC-MS data…………………………………………………………………...S59
(b) ^{1}H NMR spectrum…………………………………………………………S60

(c) ^{13}C NMR spectrum…………………………………………………………....S61
(d) FTIR spectrum………………………………………………………………...S62
(14) Characterization data for *2,2-dibromo-3-methyl-1a',9b'-dihydrospiro[cyclopropane-1,1'-cyclopropa[l]phenanthrene] (**40c**)*
(a) GC-MS data…………………………………………………………………...S63
(b) ^{1}H NMR spectrum……………………………………………………………S64
(c) ^{13}C NMR spectrum…………………………………………………………....S65
(d) FTIR spectrum………………………………………………………………...S66
(15) Characterization data for *1-(prop-1-en-1-ylidene)-1a,9b-dihydro-1H-cyclopropa[l]phenanthrene (**35c**)*
(a) ^{1}H NMR spectrum……………………………………………………………S67
(b) ^{13}C NMR spectrum…………………………………………………………....S68
(c) FTIR spectrum………………………………………………………………...S69
(16) Characterization data for *(2-(prop-1-en-1-ylidene)cyclopropane-1,1-diyl)dibenzene (**48**)*
(a) GC-MS data…………………………………………………………………...S70
(b) ^{1}H NMR spectrum……………………………………………………………S71
(c) ^{13}C NMR spectrum…………………………………………………………....S72
(d) FTIR spectrum………………………………………………………………...S73
(17) Photolysis data
(a) Photolysis A, ^{1}H NMR, t = 0 hours……………………………………………....S74
(b) Photolysis A, ^{1}H NMR, t = 4.5 hours……………………………………………S75
(c) Photolysis A, GC-MS, t = 4.5 hours……………………………………………...S76
(c) Photolysis B, GC-MS, t = 27 hours……………………………………………S77
(d) Photolysis C, ^{1}H NMR, t = 0 hours……………………………………………S78
(e) Photolysis C, ^{1}H NMR, t = 5 hours……………………………………………S79
(f) Photolysis D, ^{1}H NMR, t = 0 hours……………………………………………S80
(g) Photolysis D, ^{1}H NMR, t = 1 hour……………………………………………....S81
(13) Computational data
(a) Parent allenylidene **(1)**
(i) Optimized energies, coordinates, stability analyses, and frequencies for B2PLYP/def2-TZVP calculations..……………..………………………......S82-84
(ii) Optimized energies, coordinates, stability analyses, and frequencies for B3LYP/def2-TZVP calculations…….…………………………………..S89-91
(iii) Optimized energies, coordinates, stability analyses, and frequencies for PBE0/def2-TZVP calculations...…….………………………………………..S96-98
(iv) Optimized energies, coordinates, stability analyses, and frequencies for ωB97x-D3BJ/def2-TZVP calculations…………………………………..S103-106
(v) Single point energies and T1 diagnostics for DLPNO-CCSD(T)/def2-TZVP//B2PLYP/def2-TZVP calculations..………………………….……….….…S110-111
(vi) Single point energies and T1 diagnostics for DLPNO-CCSD(T)/def2-TZVP//B3LYP/def2-TZVP calculations…………………………..…….….……S111-112
(vii) Single point energies and T1 diagnostics for DLPNO-CCSD(T)/def2-TZVP//PBE0/def2-TZVP calculations..……………………….…….…….…..……S112
(viii) Single point energies and T1 diagnostics for DLPNO-CCSD(T)/def2-TZVP//ωB79x-D3BJ/def2-TZVP calculations……………………….…………S113
(b) Phenyl-substituted allenylidene **(36a)**

(i) Optimized energies, coordinates, stability analyses, and frequencies for B2PLYP/def2-TZVP calculations..…………….………………………..…S84-87
(ii) Optimized energies, coordinates, stability analyses, and frequencies for B3LYP/def2-TZVP calculations…….……………………………………..S91-94
(iii) Optimized energies, coordinates, stability analyses, and frequencies for PBE0/def2-TZVP calculations...……..……………………………………S98-101
(iv) Optimized energies, coordinates, stability analyses, and frequencies for ωB97x-D3BJ/def2-TZVP calculations………………………………...S106-108
(v) Single point energies and T1 diagnostics for DLPNO-CCSD(T)/def2-TZVP// B2PLYP/def2-TZVP calculations..…………………….…….….………S111
(vi) Single point energies and T1 diagnostics for DLPNO-CCSD(T)/def2-TZVP// B3LYP/def2-TZVP calculations…………………………..…..….………S112
(vii) Single point energies and T1 diagnostics for DLPNO-CCSD(T)/def2-TZVP// PBE0/def2-TZVP calculations..……………………….……….……...S112-113
(viii) Single point energies and T1 diagnostics for DLPNO-CCSD(T)/def2-TZVP//ωB79x-D3BJ/def2-TZVP calculations…………………….…………S113
(c) Methyl-substituted allenylidene **(36b)**
(i) Optimized energies, coordinates, stability analyses, and frequencies for B2PLYP/def2-TZVP calculations..…………….………………………..…S87-89
(ii) Optimized energies, coordinates, stability analyses, and frequencies for B3LYP/def2-TZVP calculations…….……………………………………..S94-96
(iii) Optimized energies, coordinates, stability analyses, and frequencies for PBE0/def2-TZVP calculations...……..………………………………...S101-103
(iv) Optimized energies, coordinates, stability analyses, and frequencies for ωB97x-D3BJ/def2-TZVP calculations………………………………...S108-110
(v) Single point energies and T1 diagnostics for DLPNO-CCSD(T)/def2-TZVP// B2PLYP/def2-TZVP calculations..…………………….…….….………S111
(vi) Single point energies and T1 diagnostics for DLPNO-CCSD(T)/def2-TZVP// B3LYP/def2-TZVP calculations…………………………..…..….………S112
(vii) Single point energies and T1 diagnostics for DLPNO-CCSD(T)/def2-TZVP// PBE0/def2-TZVP calculations..……………………….……….….........S113
(viii) Single point energies and T1 diagnostics for DLPNO-CCSD(T)/def2-TZVP//ωB79x-D3BJ/def2-TZVP calculations…………………….…………S114

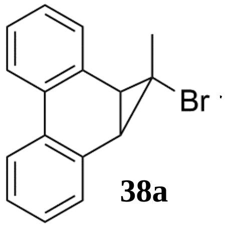
Br

38a

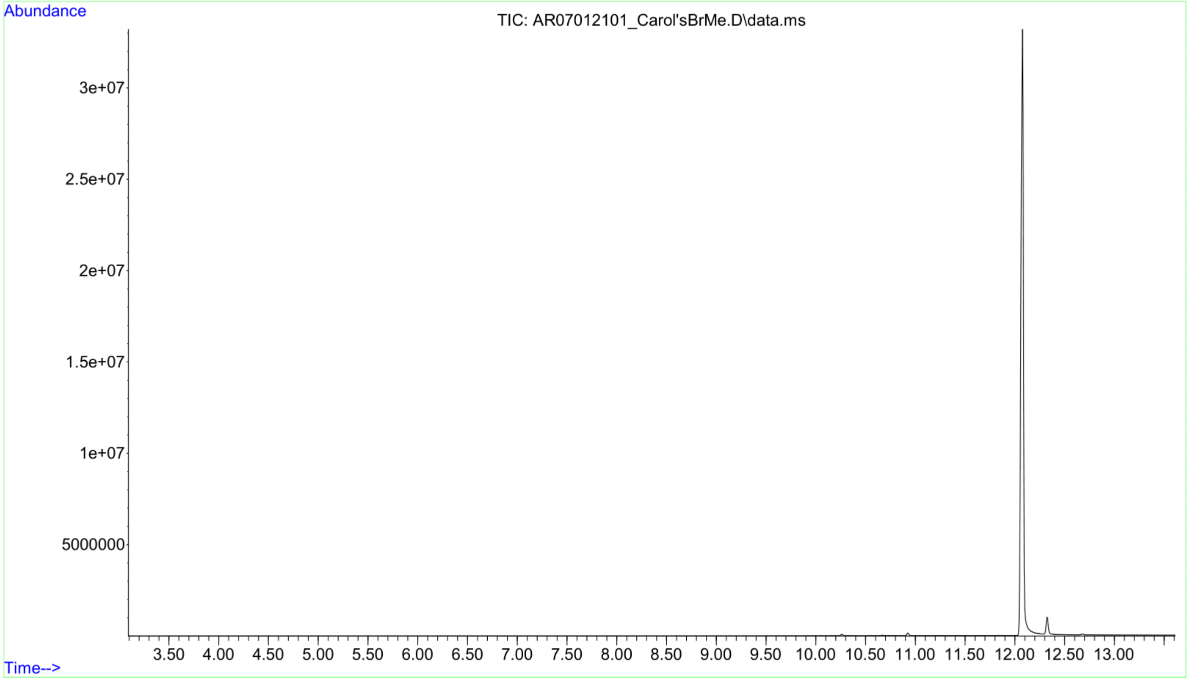
Abundance
TIC: AR07012101_Carol'sBrMe.D\data.ms
3e+07
2.5e+07
2e+07
1.5e+07
1e+07
5000000
3.50 4.00 4.50 5.00 5.50 6.00 6.50 7.00 7.50 8.00 8.50 9.00 9.50 10.00 10.50 11.00 11.50 12.00 12.50 13.00
Time-->

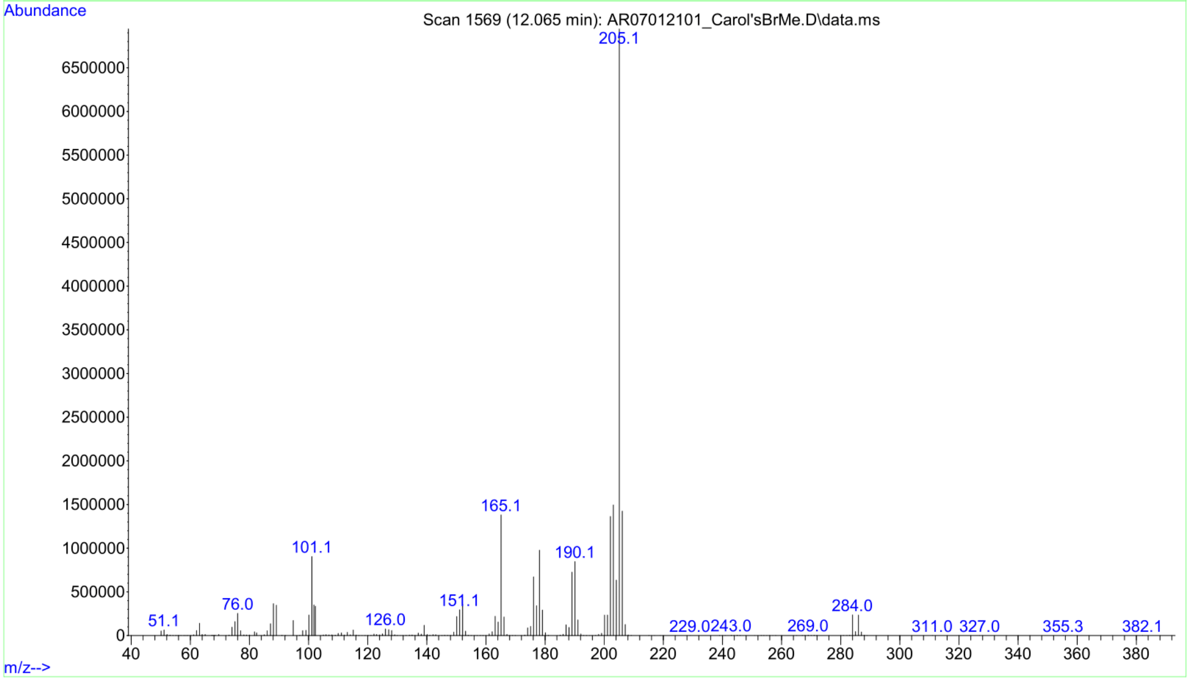
Abundance
Scan 1569 (12.065 min): AR07012101_Carol'sBrMe.D\data.ms
205.1
165.1
101.1
190.1
151.1
76.0
51.1
126.0
229.0
243.0
269.0
284.0
311.0
327.0
355.3
382.1
6500000
6000000
5500000
5000000
4500000
4000000
3500000
3000000
2500000
2000000
1500000
1000000
500000
0
40 60 80 100 120 140 160 180 200 220 240 260 280 300 320 340 360 380
m/z-->

^1H, 500MHz, $CDCl_3$

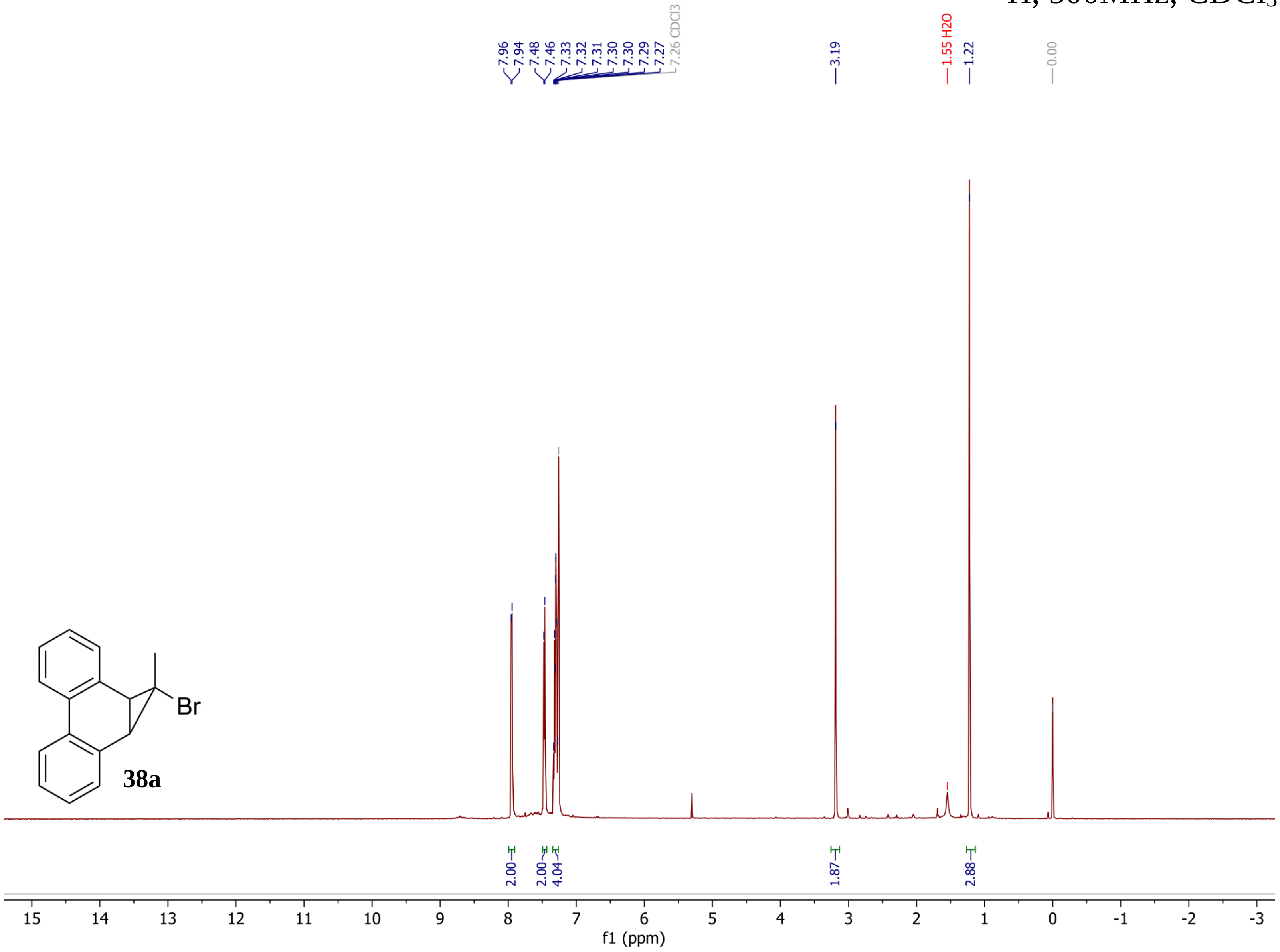

$^{13}C\{^{1}H\}$, 126 MHz, $CDCl_3$

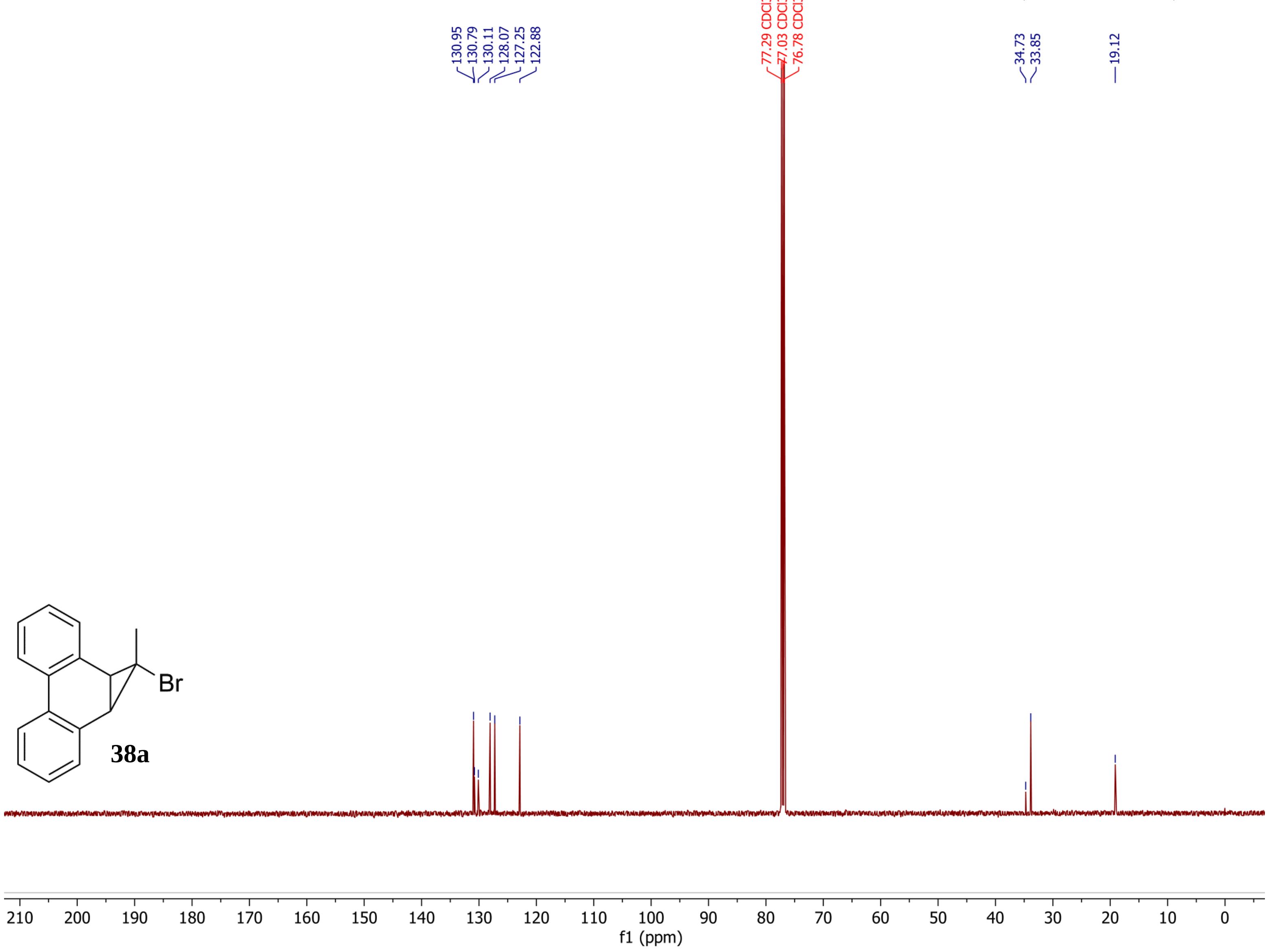

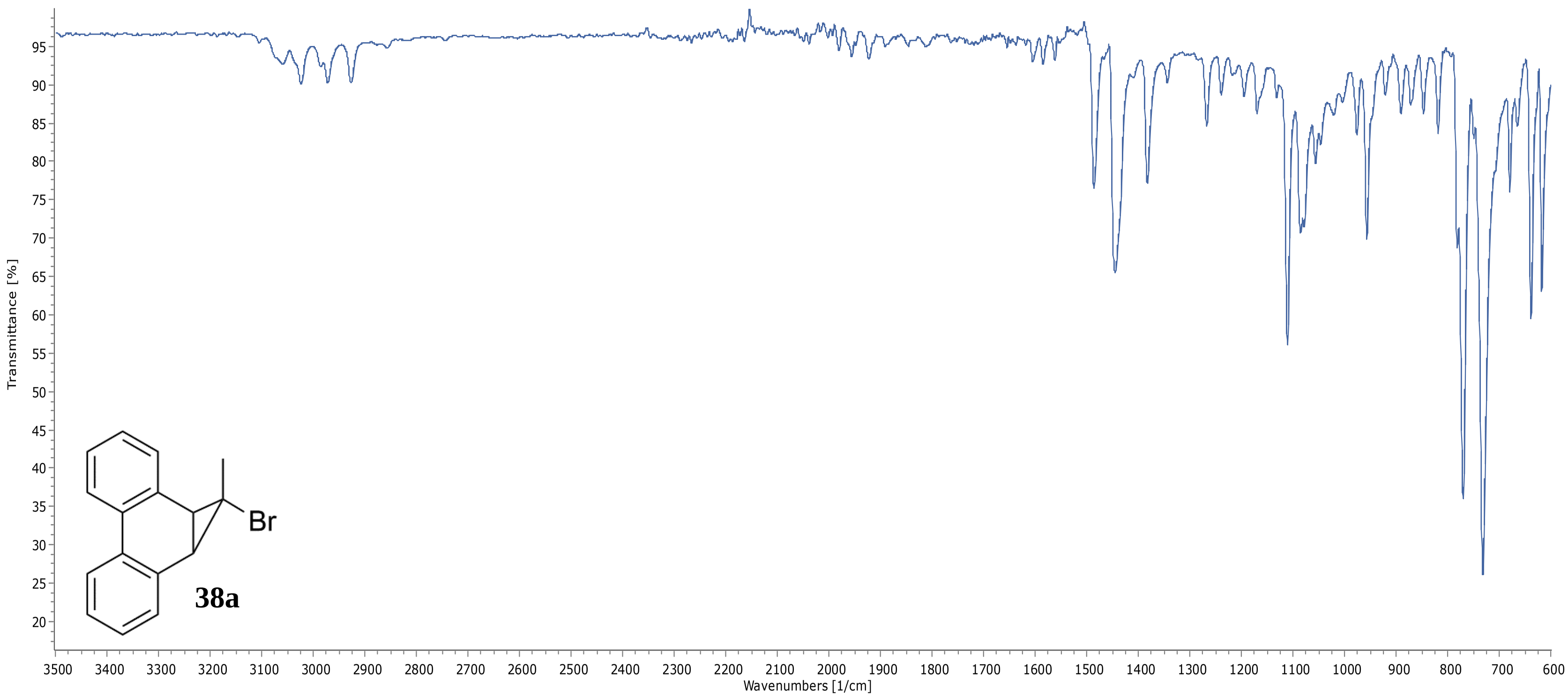
Transmittance [%]
Wavenumbers [1/cm]
Br
38a

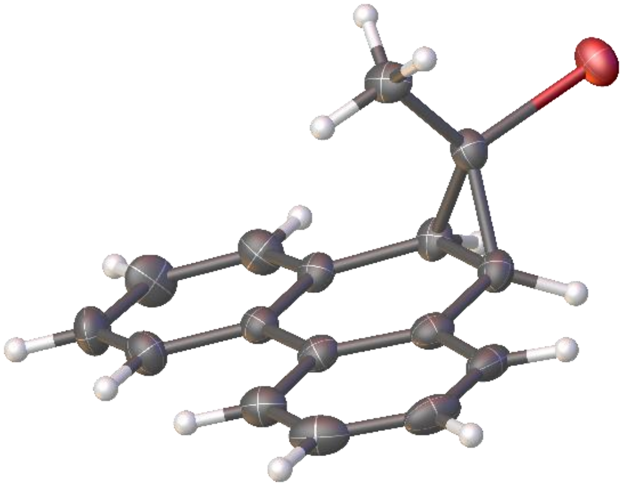

Empirical formula	$C_{16}H_{13}Br$
Formula weight	285.17
Temperature/K	173.00
Crystal system	Monoclinic
Space group	P21/c
a/Å	5.8594(3)
b/Å	16.2374(8)
c/Å	12.9987(7)
α/°	90
β/°	96.893(3)
γ/°	90
Volume/Å^3	1227.78(11)
Z	4
ρ_{calc}g/cm^3	1.543
μ/mm^{-1}	3.321
F(000)	576.0
Crystal size/mm^3	0.324 × 0.21 × 0.123
Radiation	Mo Kα (λ = 0.71073)
2Θ range for data collection/°	5.018 to 55.33
Index ranges	-7 ≤ h ≤ 7, -21 ≤ k ≤ 21, -16 ≤ l ≤ 16
Reflections collected	20854
Independent reflections	2843 [Rint = 0.0545, Rsigma = 0.0353]
Data/restraints/parameters	2843/0/155
Goodness-of-fit on F^2	1.150
Final R indexes [I>=2σ (I)]	R1 = 0.0567, wR2 = 0.1336
Final R indexes [all data]	R1 = 0.0678, wR2 = 0.1391
Largest diff. peak/hole / e Å^{-3}	1.52/-0.54
CCDC Number	2312796

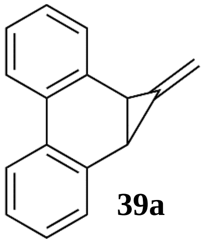

39a

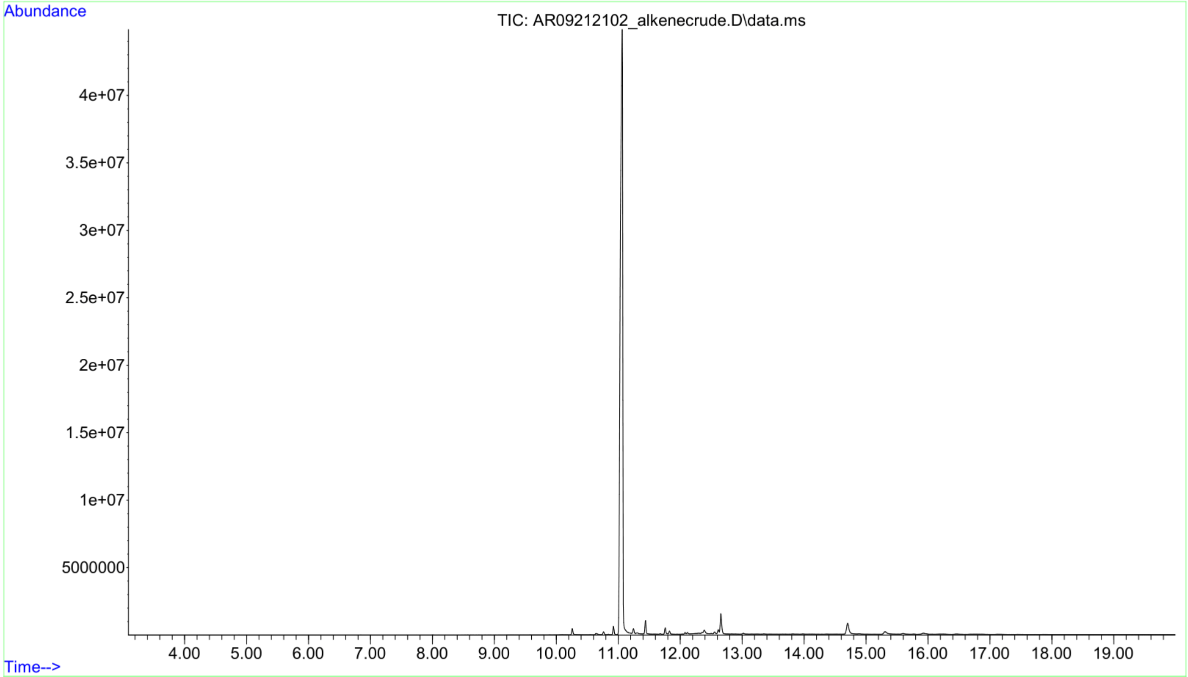

Abundance
TIC: AR09212102_alkenecrude.D\data.ms
Time-->

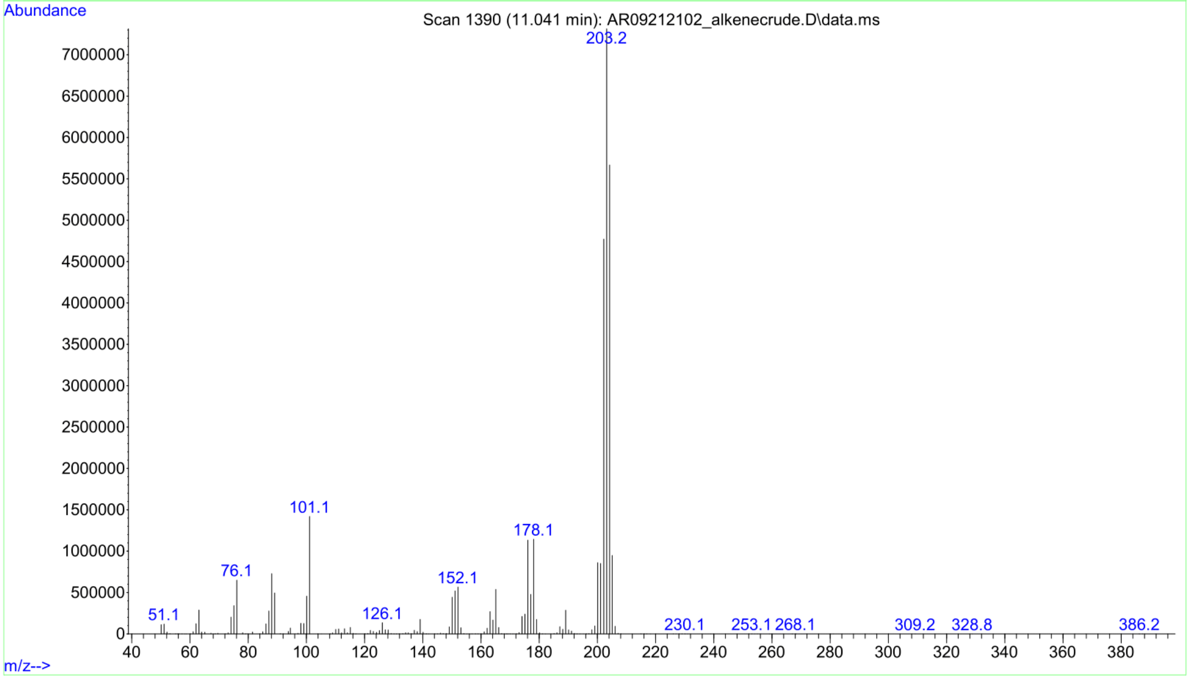

Abundance
Scan 1390 (11.041 min): AR09212102_alkenecrude.D\data.ms
203.2
101.1
178.1
76.1
152.1
51.1
126.1
230.1
253.1
268.1
309.2
328.8
386.2
m/z-->

^1H, 500MHz, $CDCl_3$

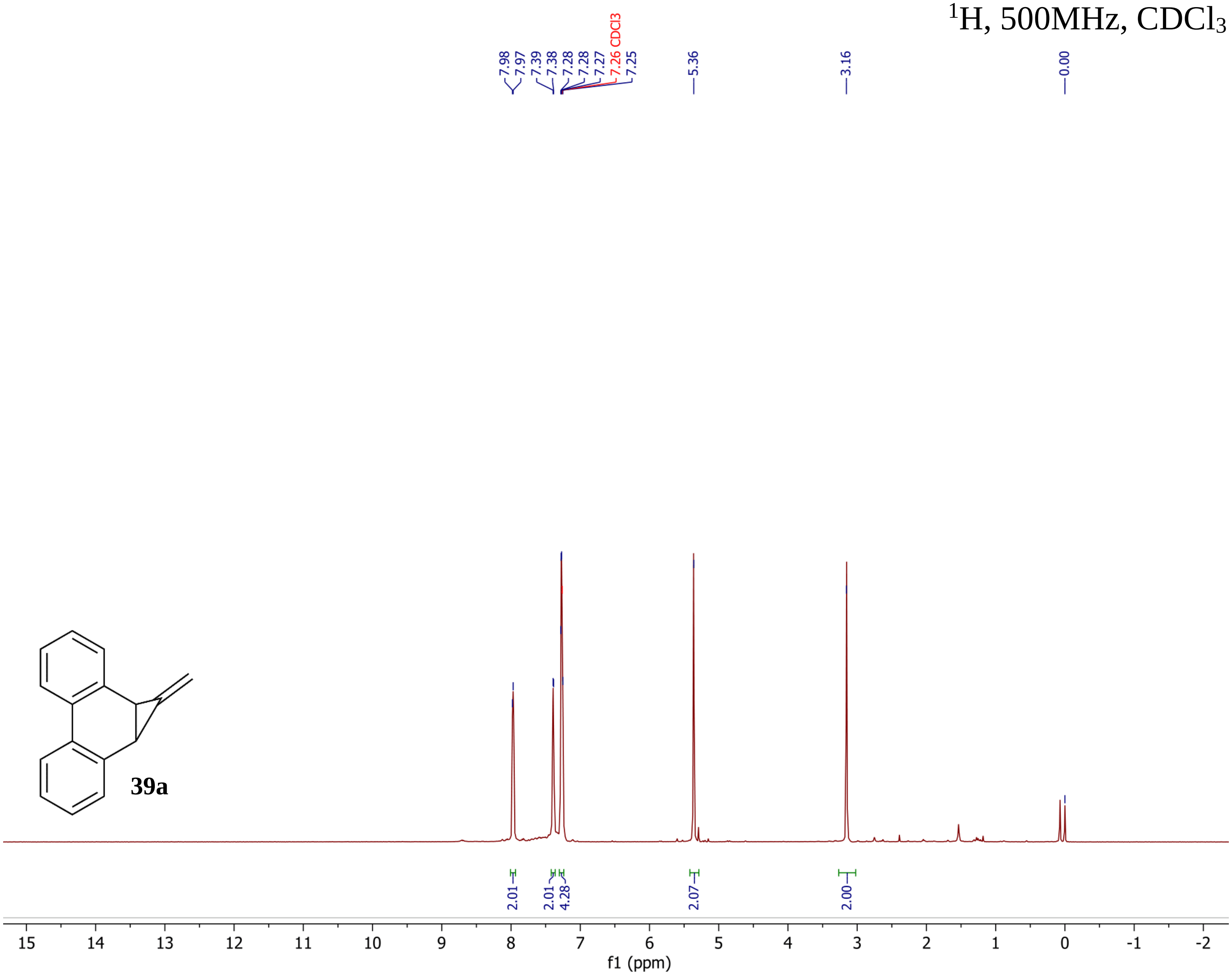

39a

$^{13}C\{^{1}H\}$, 126 MHz, $CDCl_3$

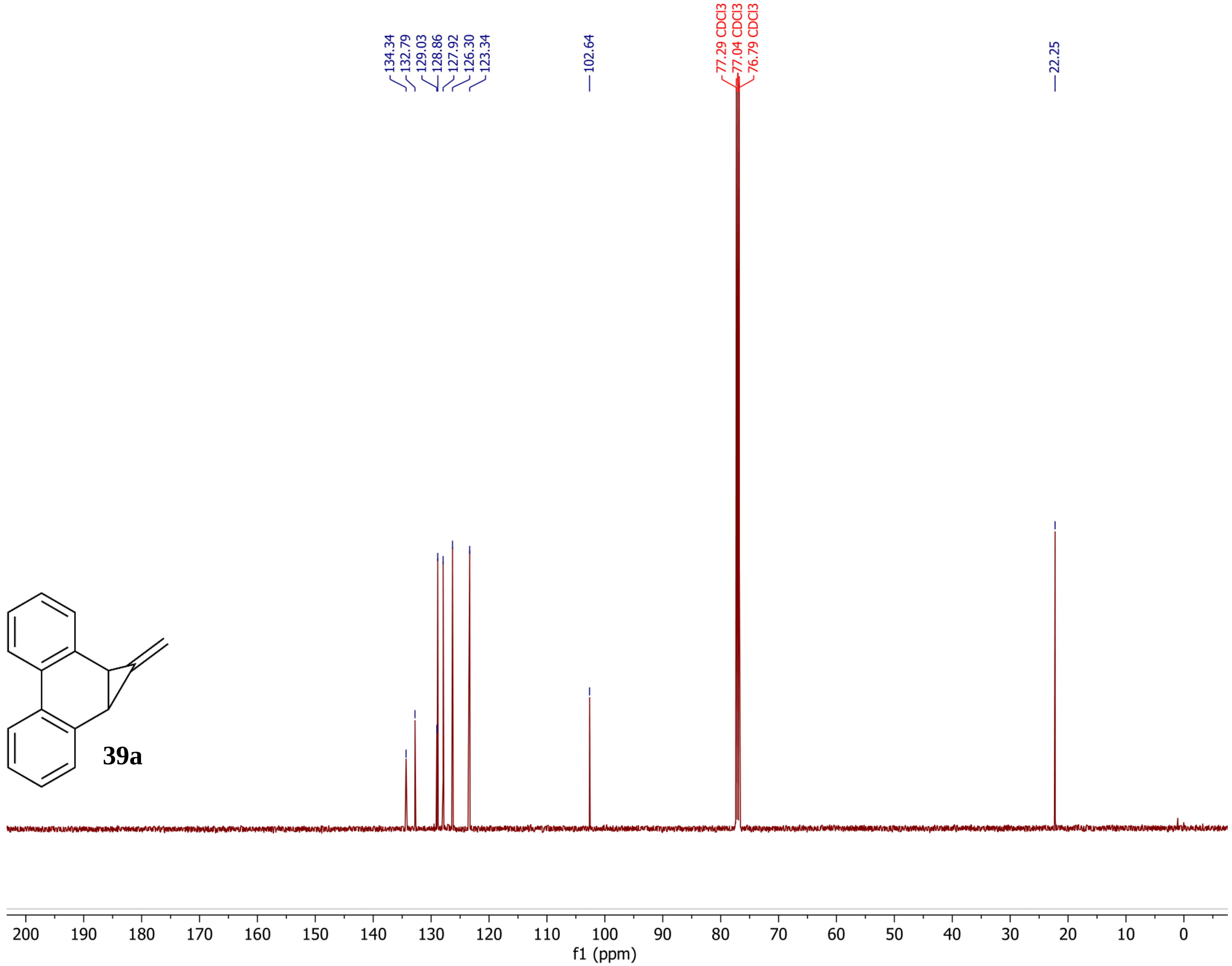

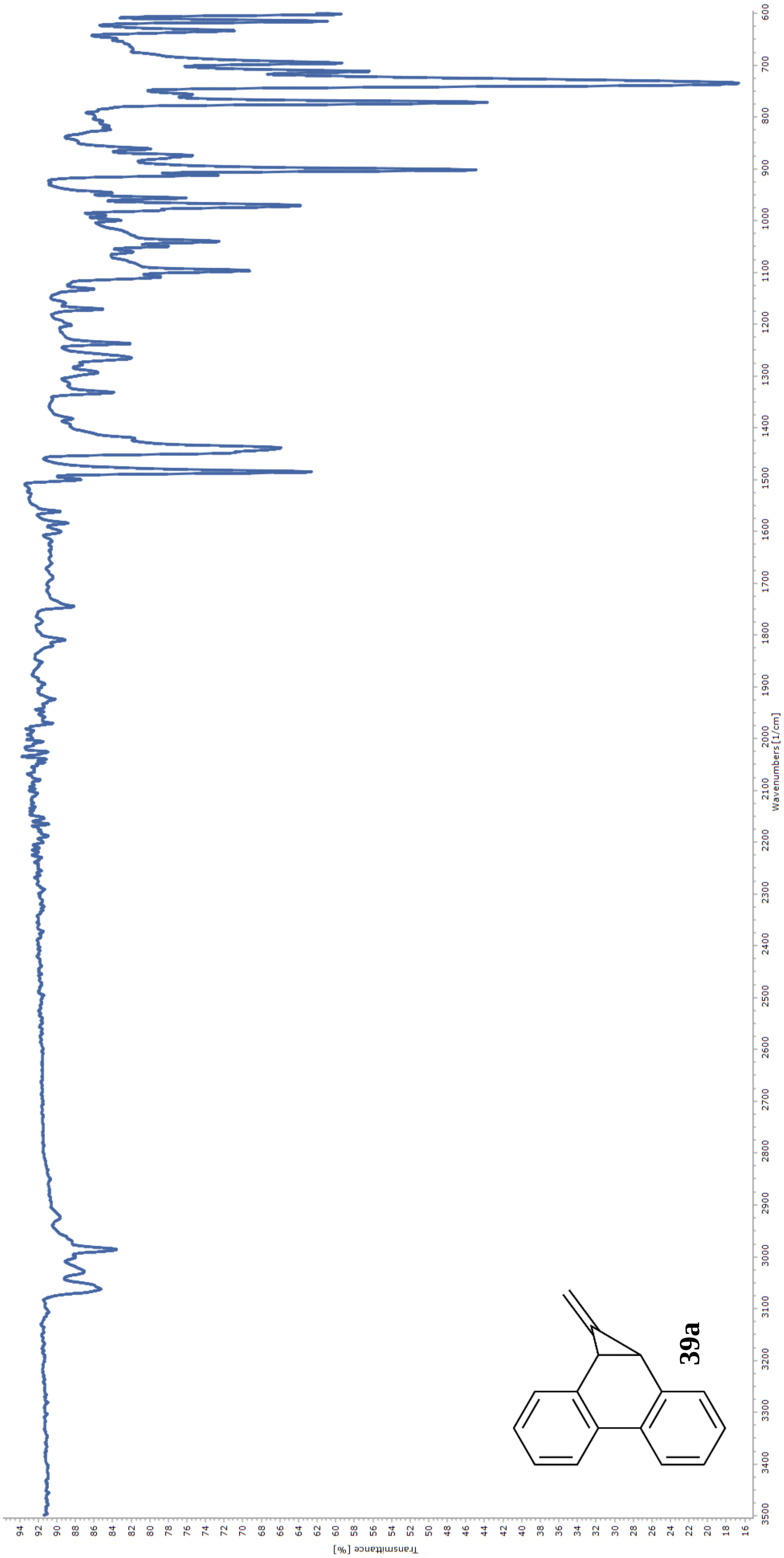
Wavenumbers [1/cm]
Transmittance [%]
39a

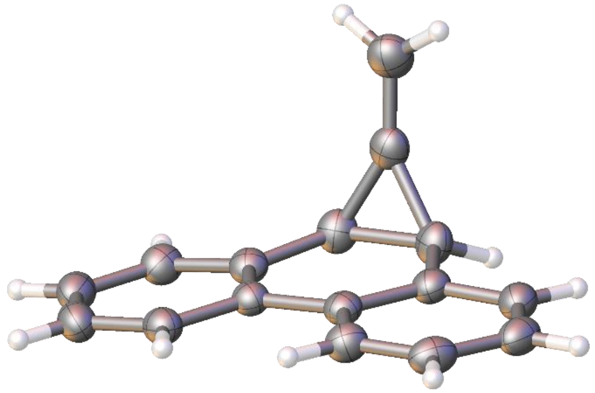

Empirical formula	$C_{16}H_{12}$
Formula weight	204.26
Temperature/K	173.00
Crystal system	Orthorhombic
Space group	Pbca
a/Å	11.7309(3)
b/Å	7.5417(2)
c/Å	24.2285(6)
α/°	90
β/°	90
γ/°	90
Volume/Å^3	2143.52(10)
Z	8
ρ_{calc}g/cm^3	1.266
μ/mm^{-1}	0.071
F(000)	864.0
Crystal size/mm^3	0.416 × 0.331 × 0.165
Radiation	MoKα (λ = 0.71073)
2Θ range for data collection/°	6.64 to 54.984
Index ranges	-15 ≤ h ≤ 15, -9 ≤ k ≤ 9, -31 ≤ l ≤ 31
Reflections collected	44666
Independent reflections	2444 [Rint = 0.0354, Rsigma = 0.0127]
Data/restraints/parameters	2444/0/147
Goodness-of-fit on F^2	1.053
Final R indexes [I>=2σ (I)]	R1 = 0.0534, wR2 = 0.0871
Final R indexes [all data]	R1 = 0.0740, wR2 = 0.1026
Largest diff. peak/hole / e Å^{-3}	0.25/-0.20
CCDC Number	2261489

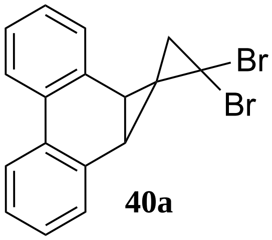

Br
Br

40a

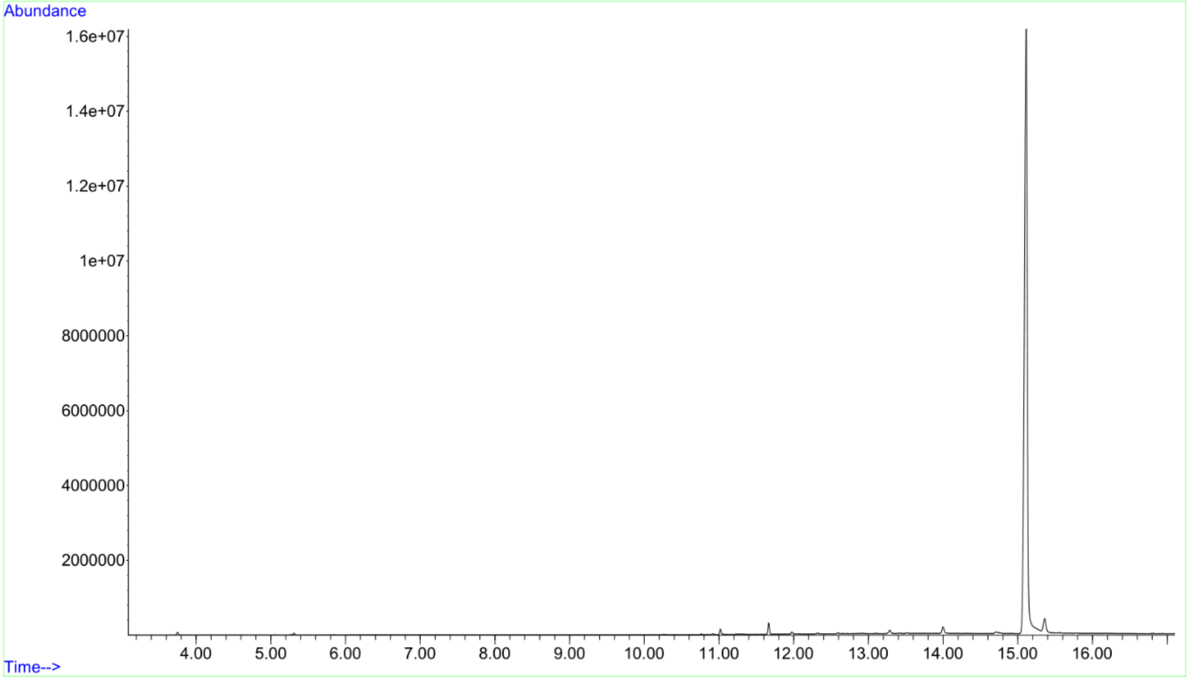

Abundance
1.6e+07
1.4e+07
1.2e+07
1e+07
8000000
6000000
4000000
2000000
4.00
5.00
6.00
7.00
8.00
9.00
10.00
11.00
12.00
13.00
14.00
15.00
16.00
Time-->

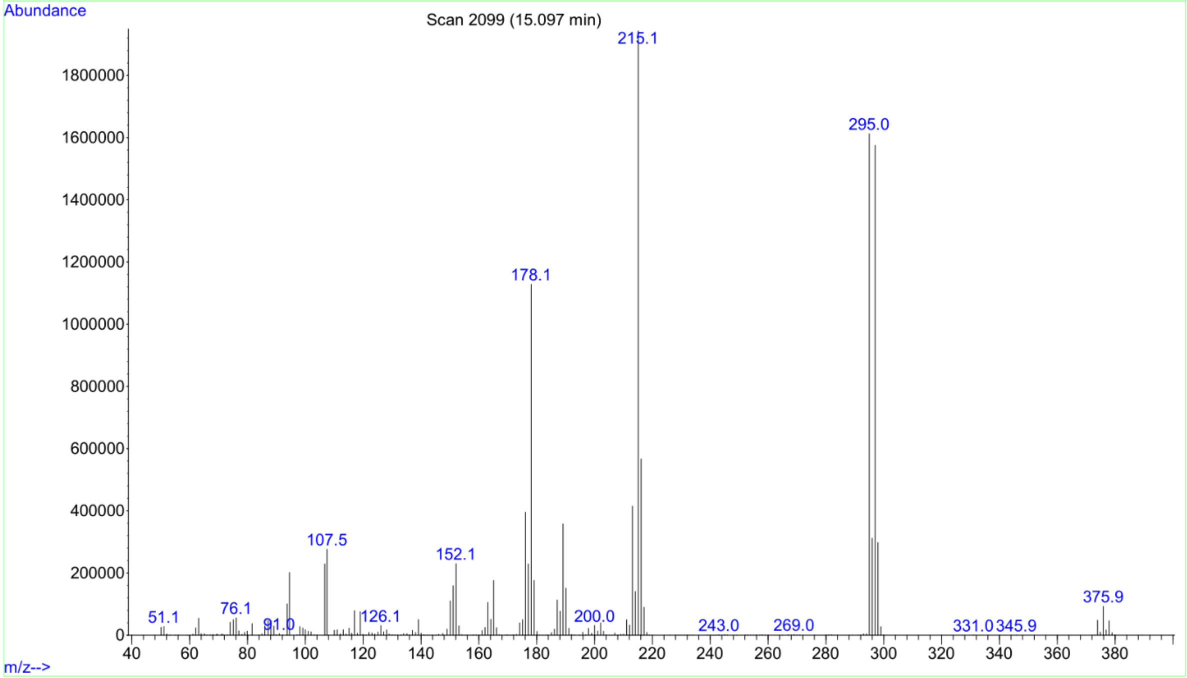

Abundance
Scan 2099 (15.097 min)
215.1
295.0
178.1
107.5
152.1
51.1
76.1
91.0
126.1
200.0
243.0
269.0
331.0
345.9
375.9
m/z-->

^{1}H, 500MHz, $CDCl_3$

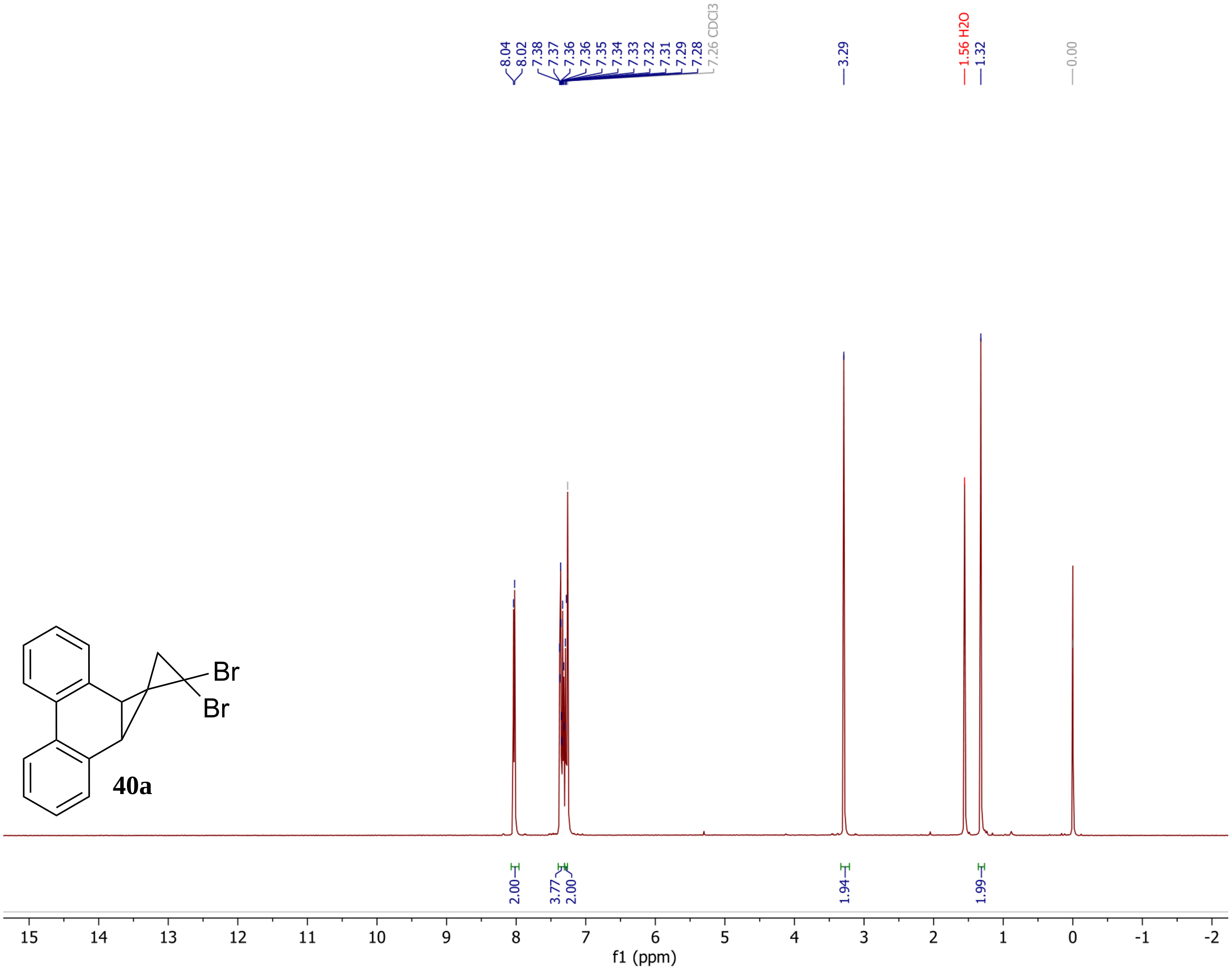

40a

$^{13}C\{^{1}H\}$, 126 MHz, $CDCl_3$

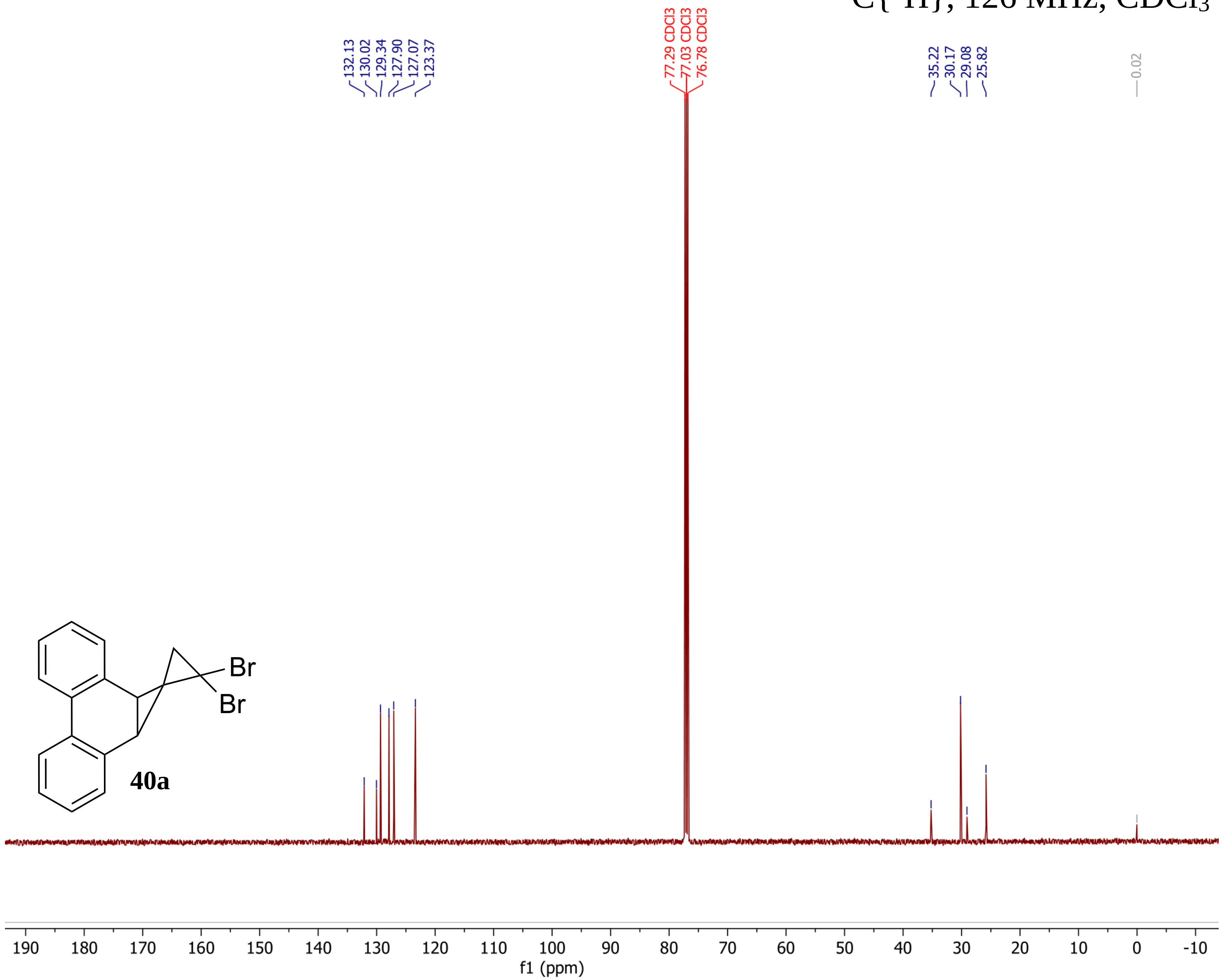

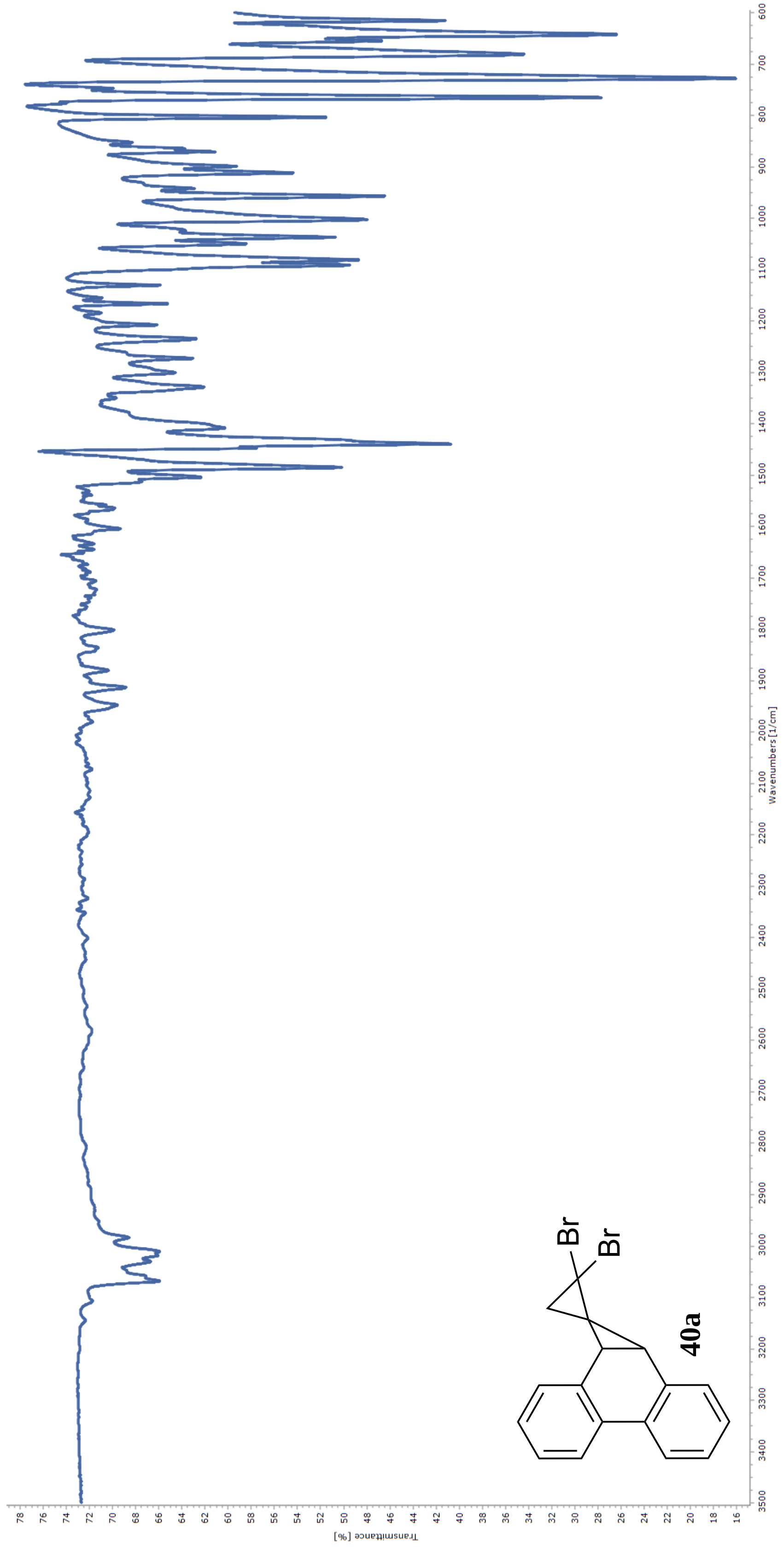
Transmittance [%]
Wavenumbers [1/cm]
Br
Br
40a

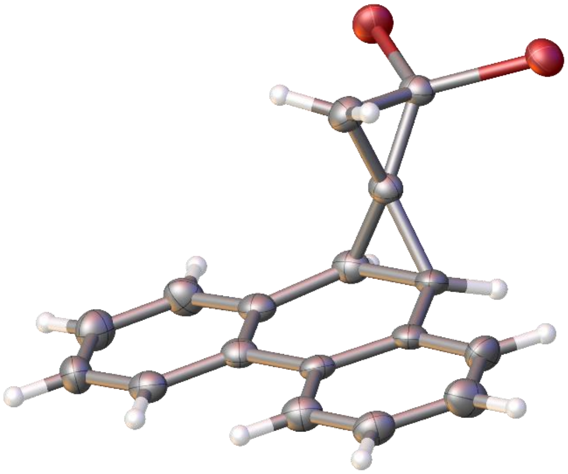

Empirical formula	$C_{17}H_{12}Br_2$
Formula weight	376.09
Temperature/K	173.00
Crystal system	Monoclinic
Space group	P21/n
a/Å	15.8340(6)
b/Å	5.4029(2)
c/Å	16.5725(6)
α/°	90
β/°	96.946(2)
γ/°	90
Volume/Å^3	1407.36(9)
Z	4
ρ_{calc}g/cm^3	1.775
μ/mm^{-1}	5.742
F(000)	736.0
Crystal size/mm^3	0.328 × 0.242 × 0.054
Radiation	MoKα (λ = 0.71073)
2Θ range for data collection/°	6.724 to 54.994
Index ranges	-20 ≤ h ≤ 20, -7 ≤ k ≤ 7, -21 ≤ l ≤ 21
Reflections collected	29962
Independent reflections	3189 [Rint = 0.0406, Rsigma = 0.0211]
Data/restraints/parameters	3189/0/172
Goodness-of-fit on F^2	1.179
Final R indexes [I>=2σ (I)]	R1 = 0.0483, wR2 = 0.1239
Final R indexes [all data]	R1 = 0.0607, wR2 = 0.1308
Largest diff. peak/hole / e Å^{-3}	1.33/-0.85
CCDC Number	2267018

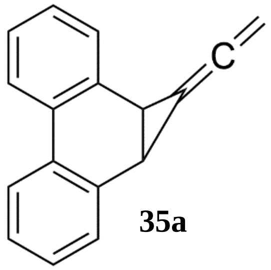

35a

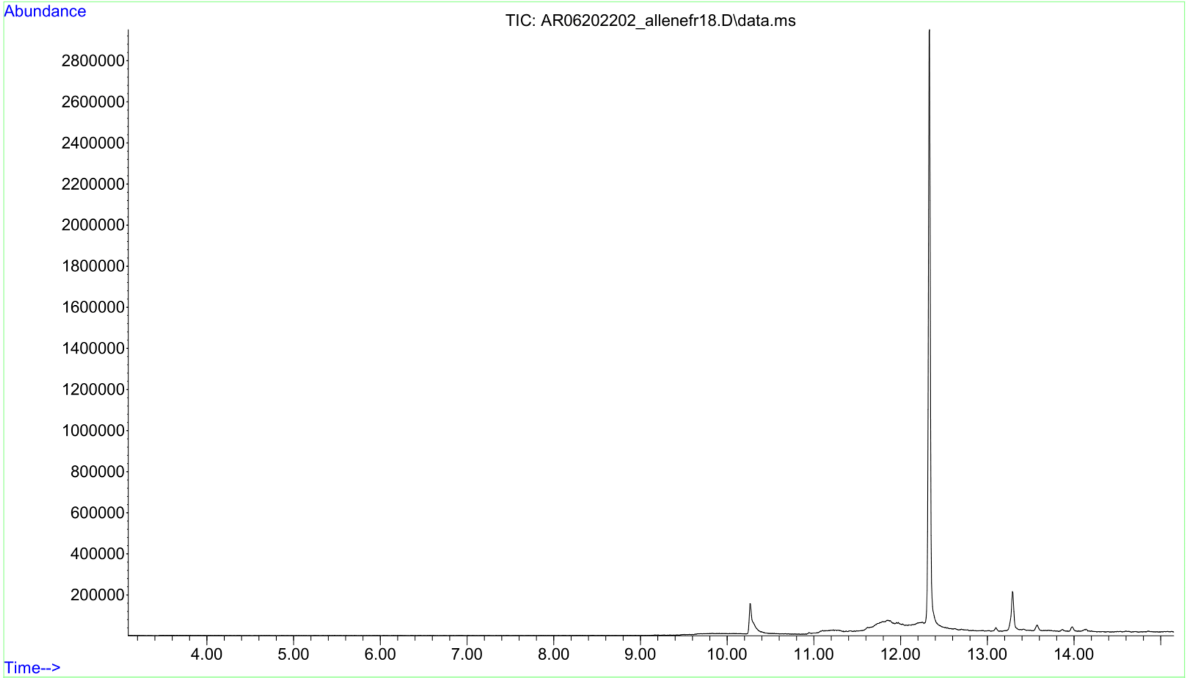
Abundance
TIC: AR06202202_allenefr18.D\data.ms
2800000
2600000
2400000
2200000
2000000
1800000
1600000
1400000
1200000
1000000
800000
600000
400000
200000
4.00
5.00
6.00
7.00
8.00
9.00
10.00
11.00
12.00
13.00
14.00
Time-->

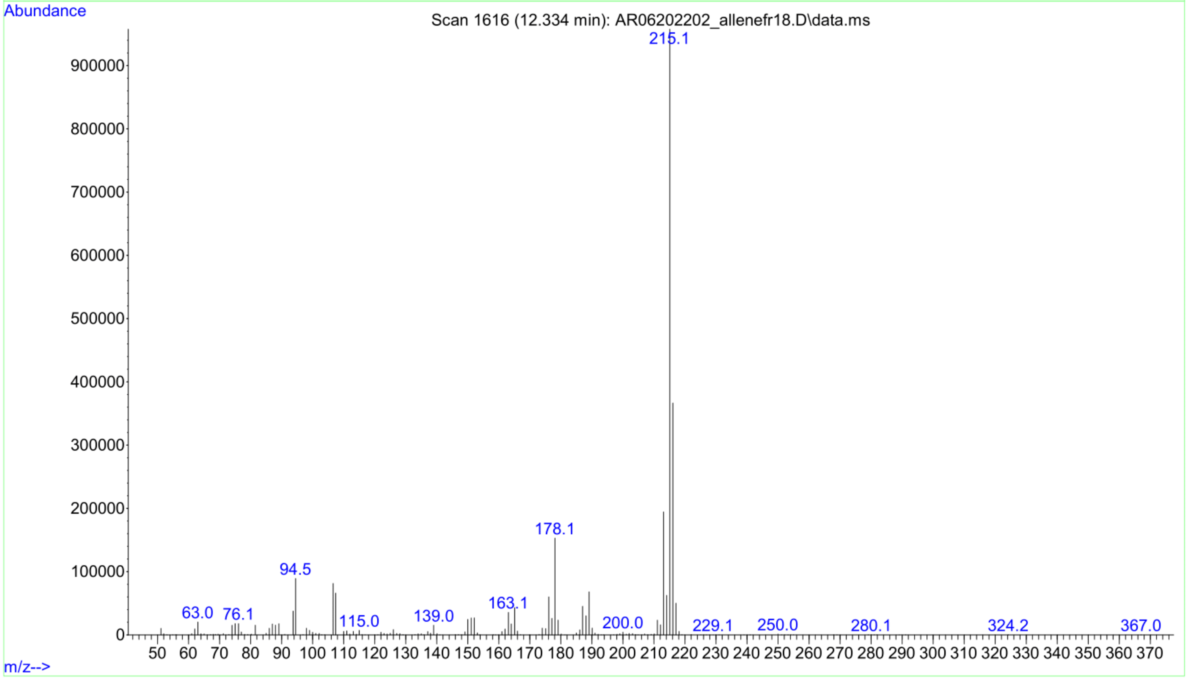
Abundance
Scan 1616 (12.334 min): AR06202202_allenefr18.D\data.ms
215.1
900000
800000
700000
600000
500000
400000
300000
200000
100000
0
178.1
94.5
63.0
76.1
115.0
139.0
163.1
200.0
229.1
250.0
280.1
324.2
367.0
50 60 70 80 90 100 110 120 130 140 150 160 170 180 190 200 210 220 230 240 250 260 270 280 290 300 310 320 330 340 350 360 370
m/z-->

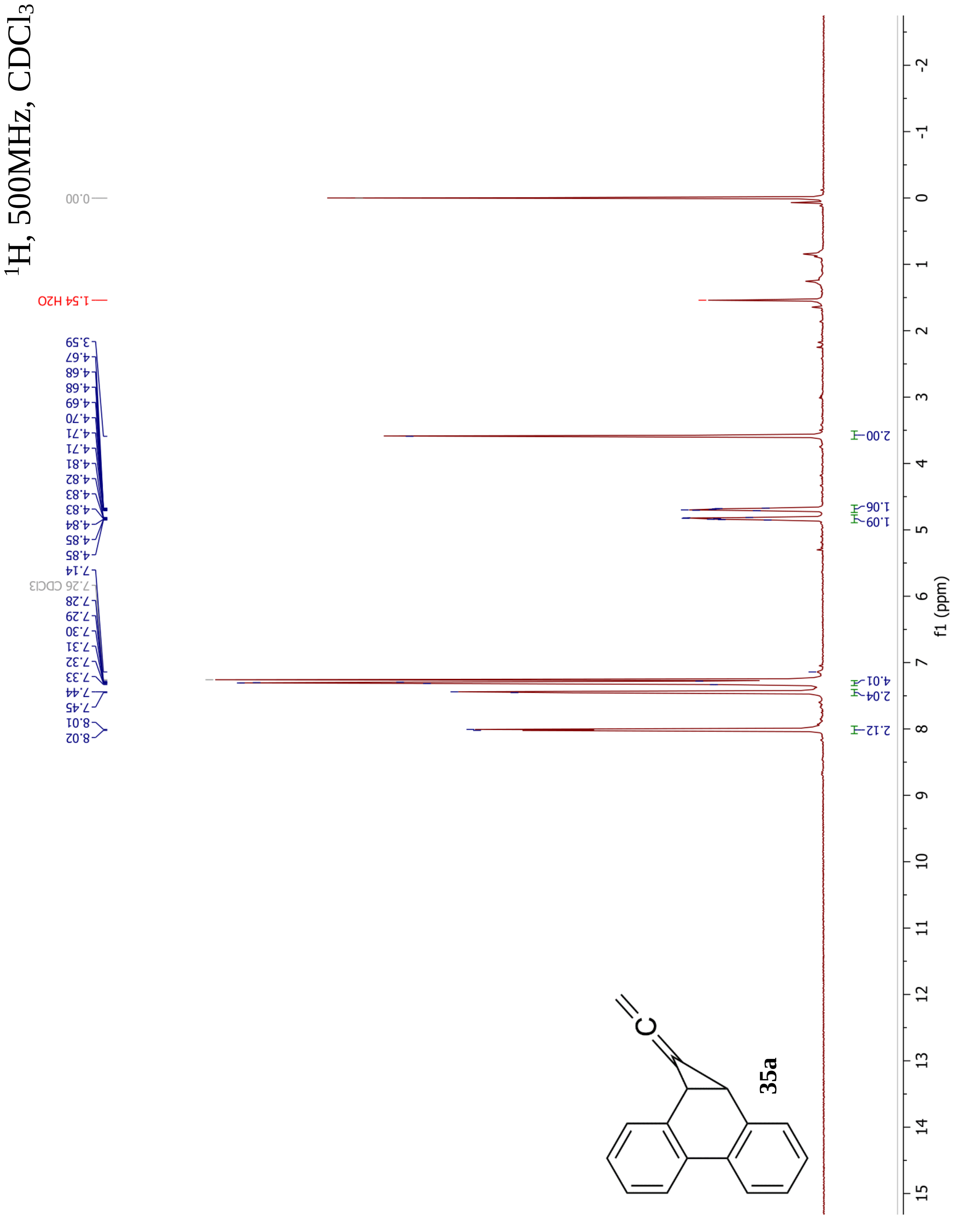

1H, 500MHz, CDCl3
0.00
1.54 H2O
3.59
4.67
4.68
4.68
4.69
4.70
4.71
4.71
4.81
4.82
4.83
4.83
4.84
4.85
4.85
7.14
7.26 CDCl3
7.28
7.29
7.30
7.31
7.32
7.33
7.44
7.45
8.01
8.02
2.00
1.06
1.09
4.01
2.04
2.12
f1 (ppm)
35a
C

$^{13}C\{^{1}H\}$, 126 MHz, $CDCl_3$

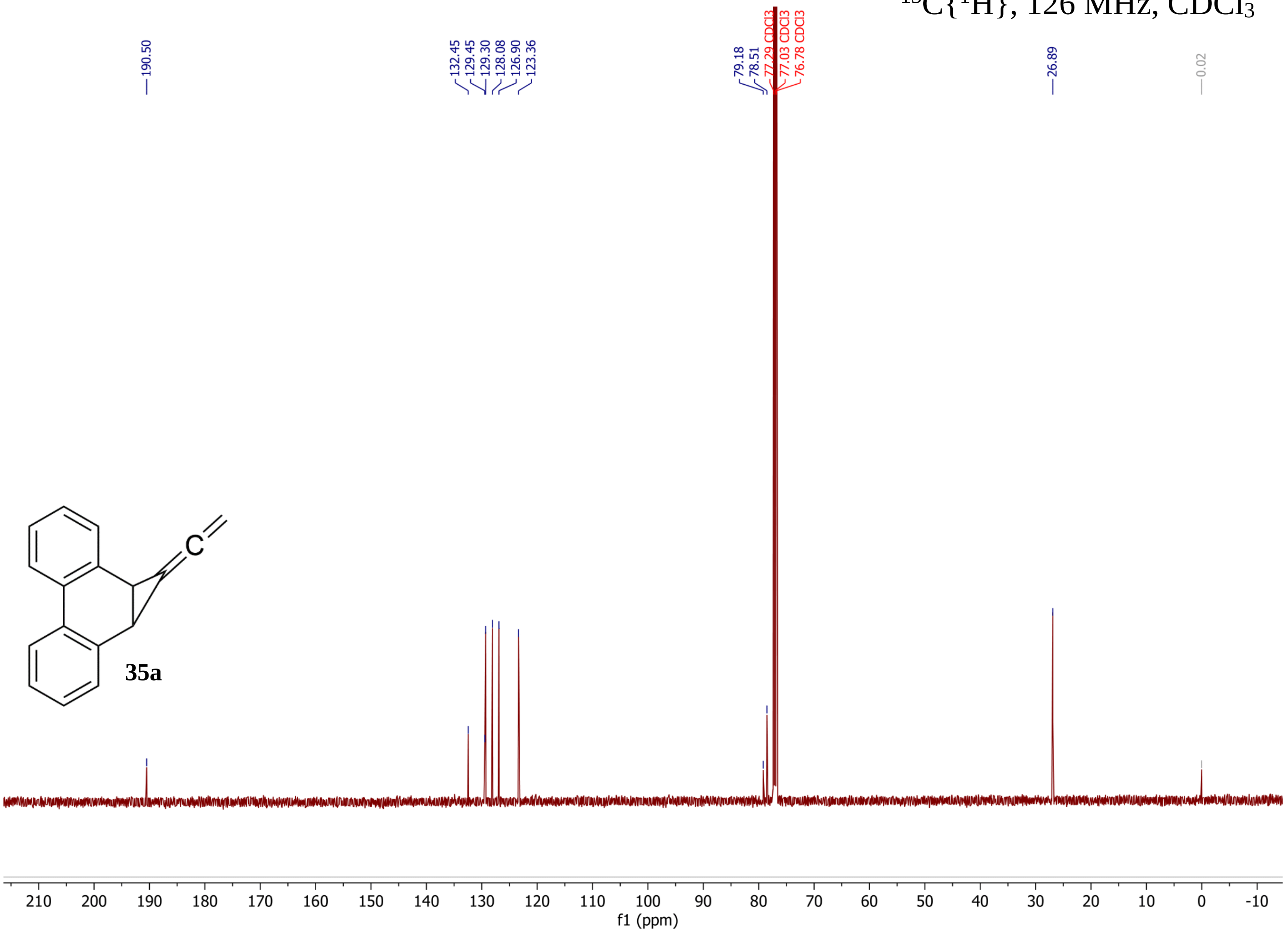

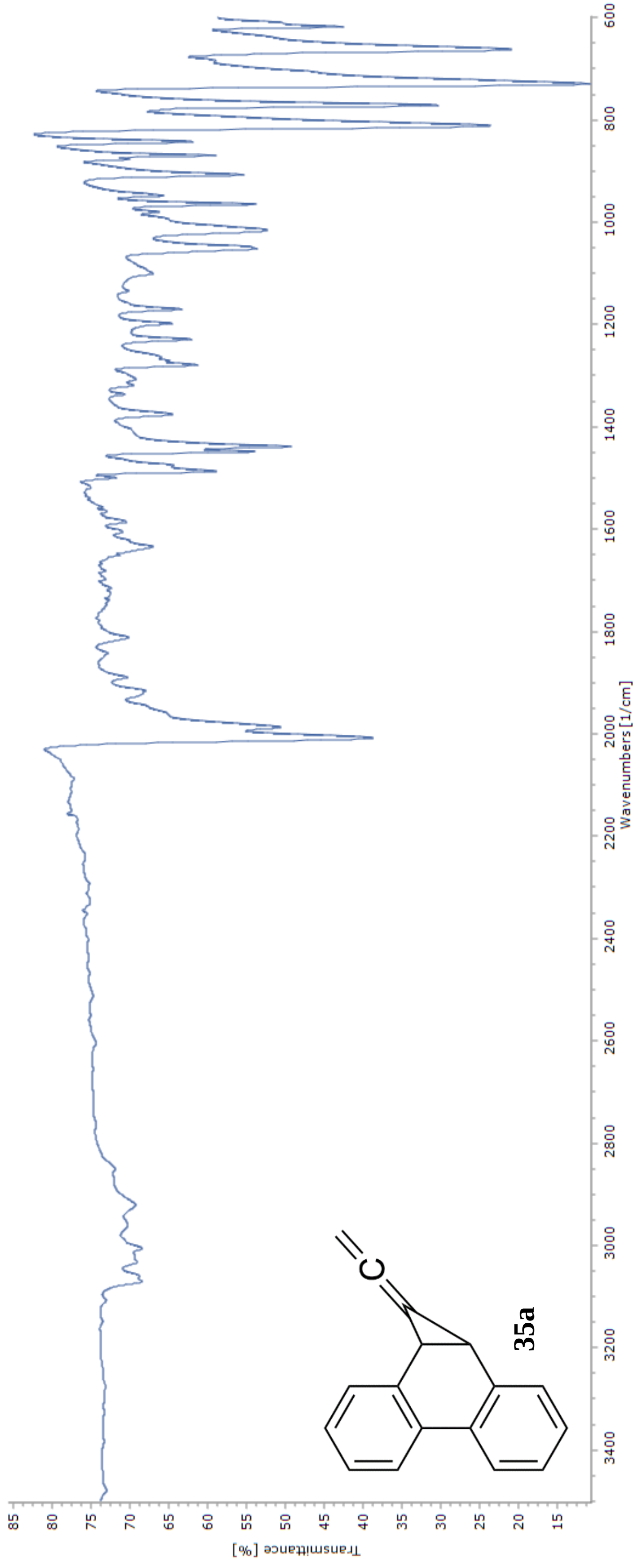

Transmittance [%]
Wavenumbers [1/cm]
35a

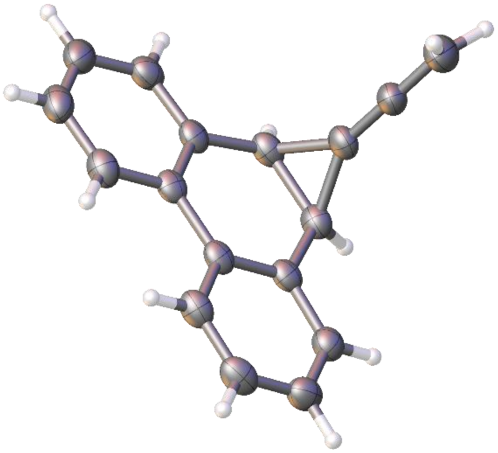

Empirical formula	$C_{17}H_{12}$
Formula weight	216.27
Temperature/K	173.11
Crystal system	Orthorhombic
Space group	Pna21
a/Å	17.3695(9)
b/Å	13.4559(7)
c/Å	4.9150(3)
α/°	90
β/°	90
γ/°	90
Volume/Å^3	1148.74(11)
Z	4
ρ_{calc}g/cm^3	1.250
μ/mm^{-1}	0.071
F(000)	456.0
Crystal size/mm^3	0.32 × 0.13 × 0.07
Radiation	MoKα (λ = 0.71073)
2Θ range for data collection/°	5.582 to 52.81
Index ranges	-19 ≤ h ≤ 20, -16 ≤ k ≤ 16, -5 ≤ l ≤ 6
Reflections collected	8908
Independent reflections	2248 [Rint = 0.0230, Rsigma = 0.0210]
Data/restraints/parameters	2248/1/162
Goodness-of-fit on F^2	1.030
Final R indexes [I>=2σ (I)]	R1 = 0.0326, wR2 = 0.0877
Final R indexes [all data]	R1 = 0.0440, wR2 = 0.0959
Largest diff. peak/hole / e Å^{-3}	0.14/-0.16
Flack parameter	4.3(10)
CCDC Number	2267948

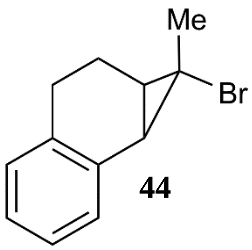
Me
Br

44

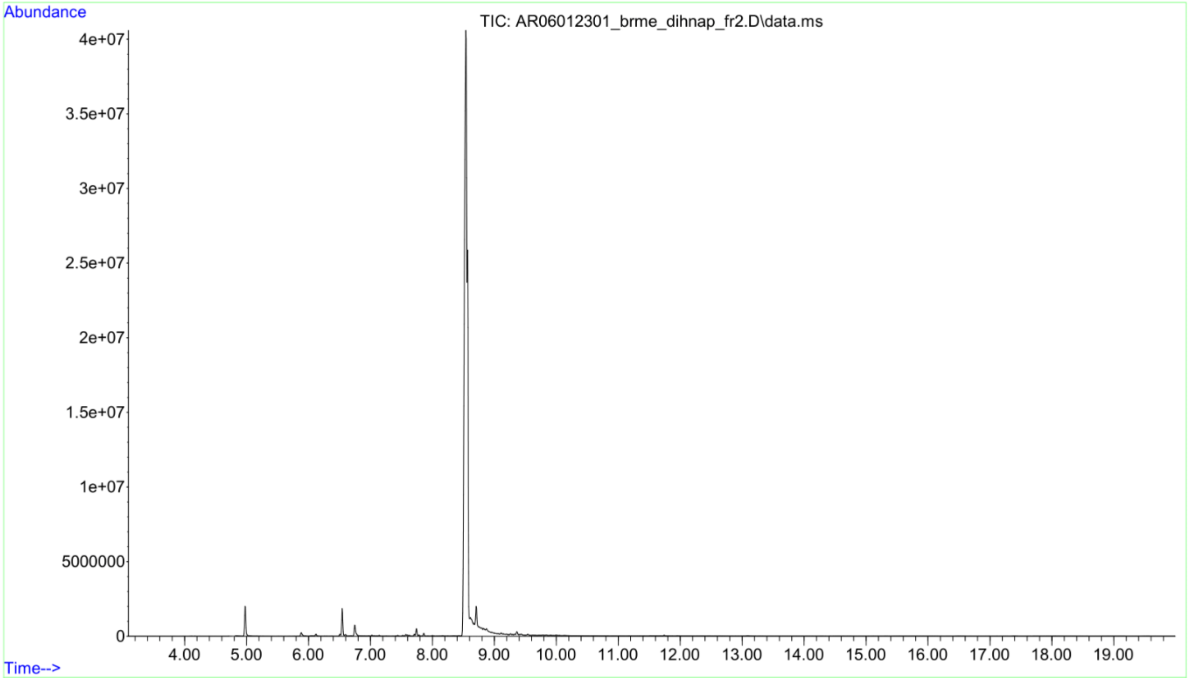
Abundance
TIC: AR06012301_brme_dihnap_fr2.D\data.ms
4e+07
3.5e+07
3e+07
2.5e+07
2e+07
1.5e+07
1e+07
5000000
0
4.00 5.00 6.00 7.00 8.00 9.00 10.00 11.00 12.00 13.00 14.00 15.00 16.00 17.00 18.00 19.00
Time-->

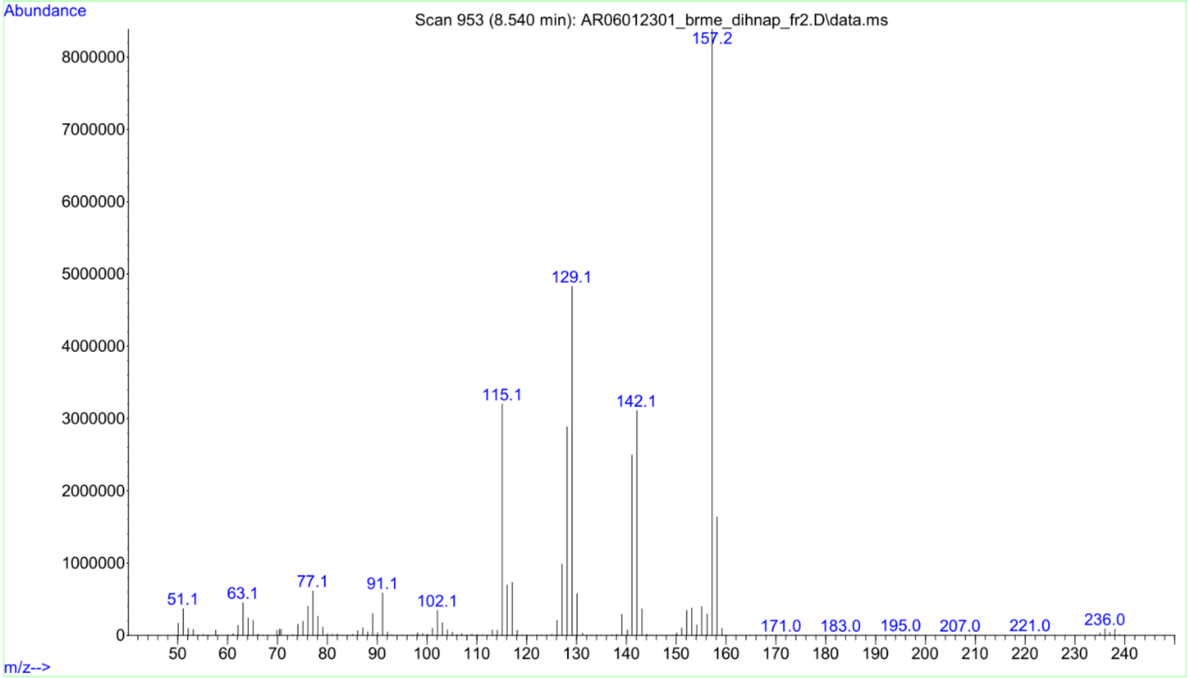
Abundance
Scan 953 (8.540 min): AR06012301_brme_dihnap_fr2.D\data.ms
157.2
129.1
115.1
142.1
51.1
63.1
77.1
91.1
102.1
171.0
183.0
195.0
207.0
221.0
236.0
8000000
7000000
6000000
5000000
4000000
3000000
2000000
1000000
0
50 60 70 80 90 100 110 120 130 140 150 160 170 180 190 200 210 220 230 240
m/z-->

^1H, 500MHz, $CDCl_3$

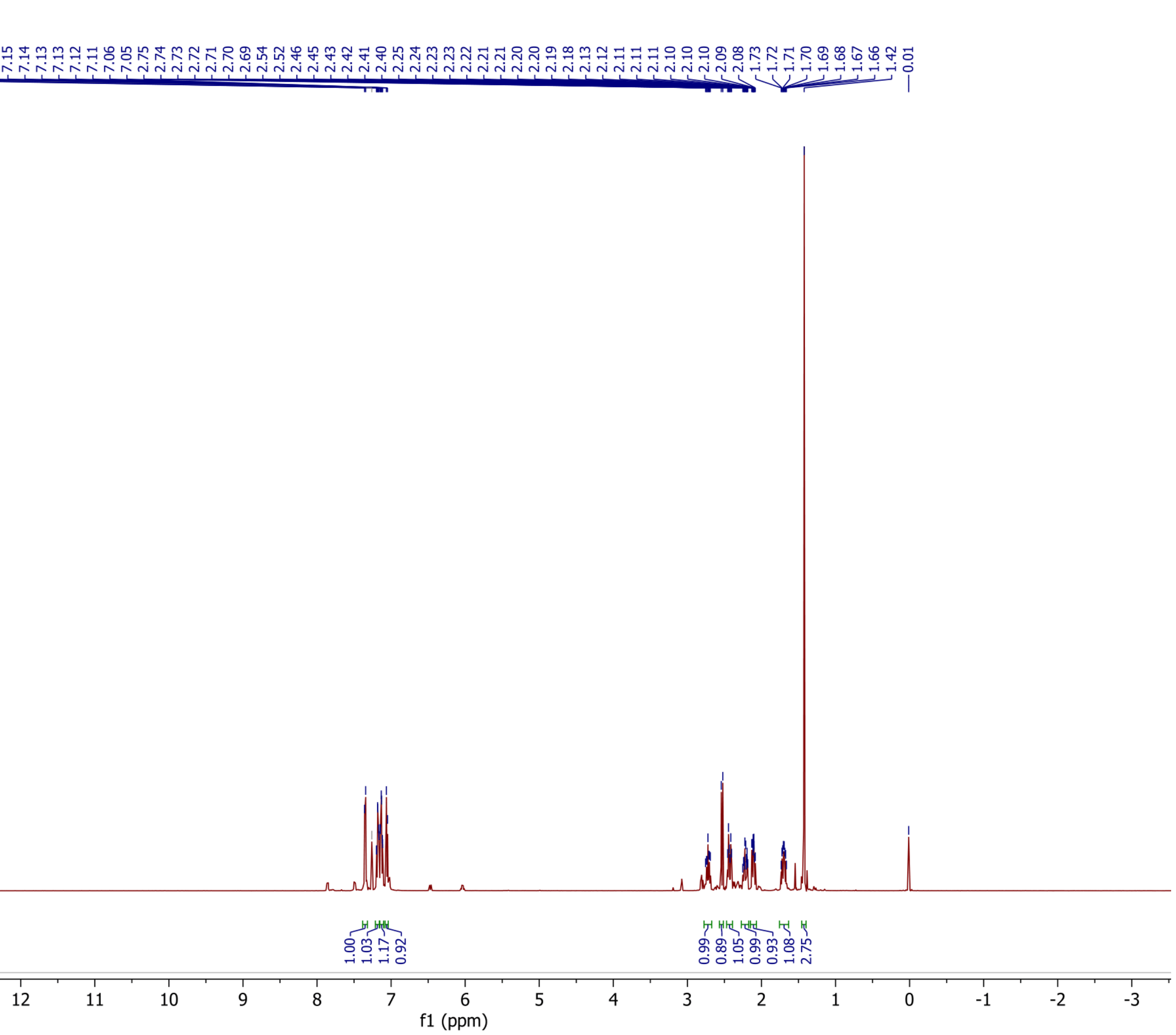

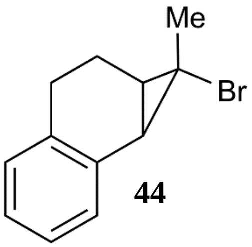

44

$^{13}C\{^{1}H\}$, 126 MHz, $CDCl_3$

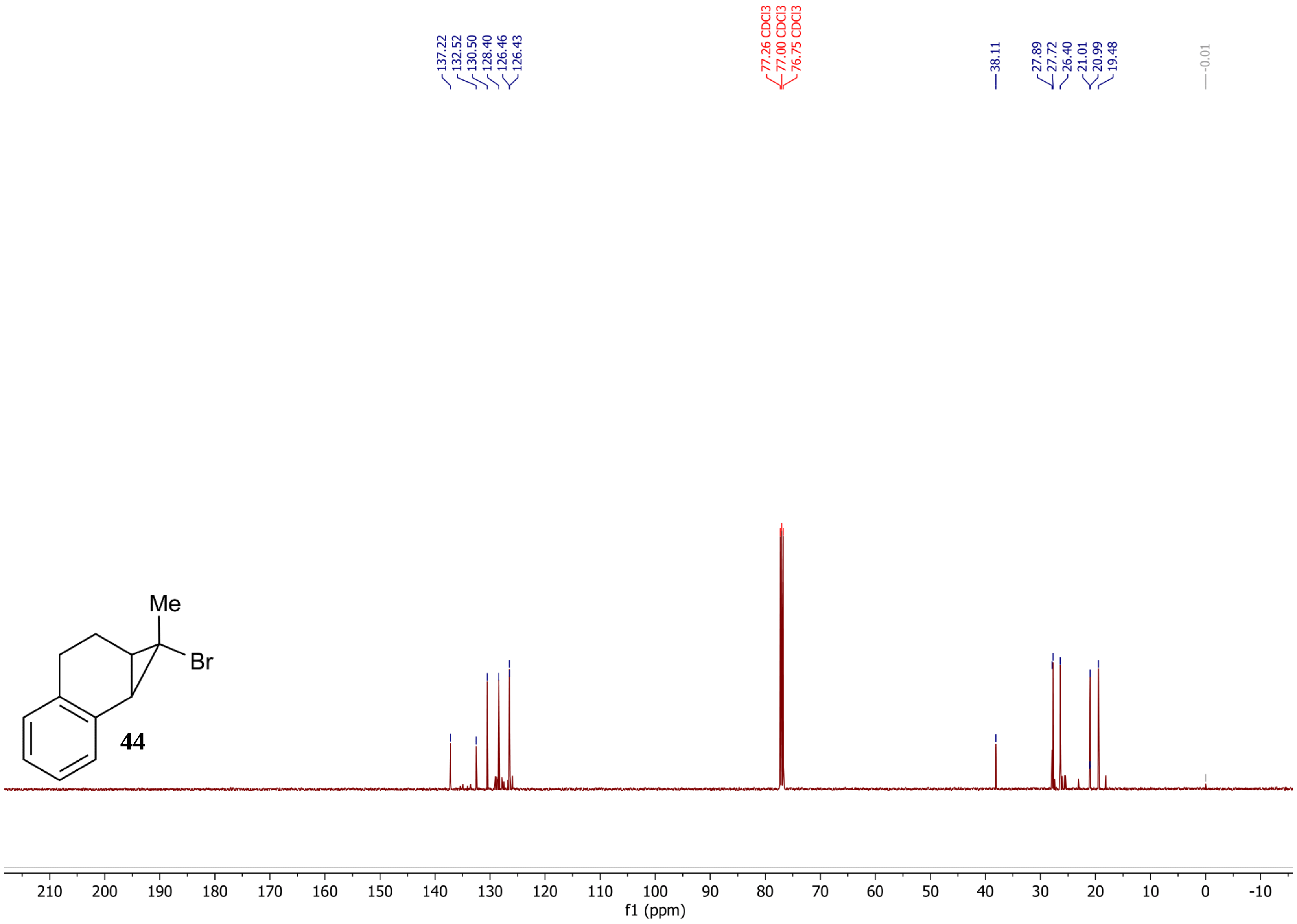

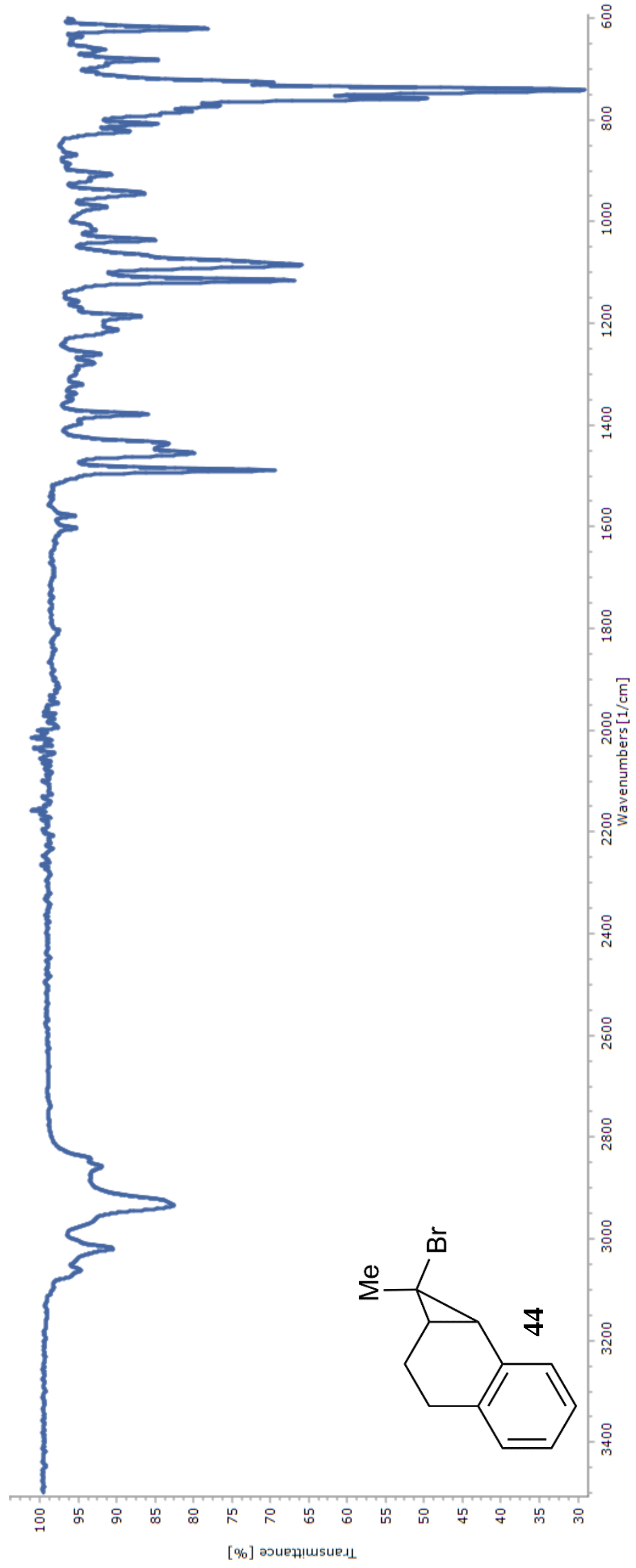
Transmittance [%]
Wavenumbers [1/cm]
Me
Br
44

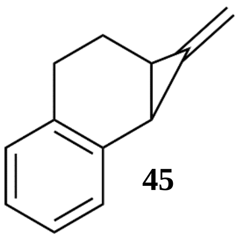

45

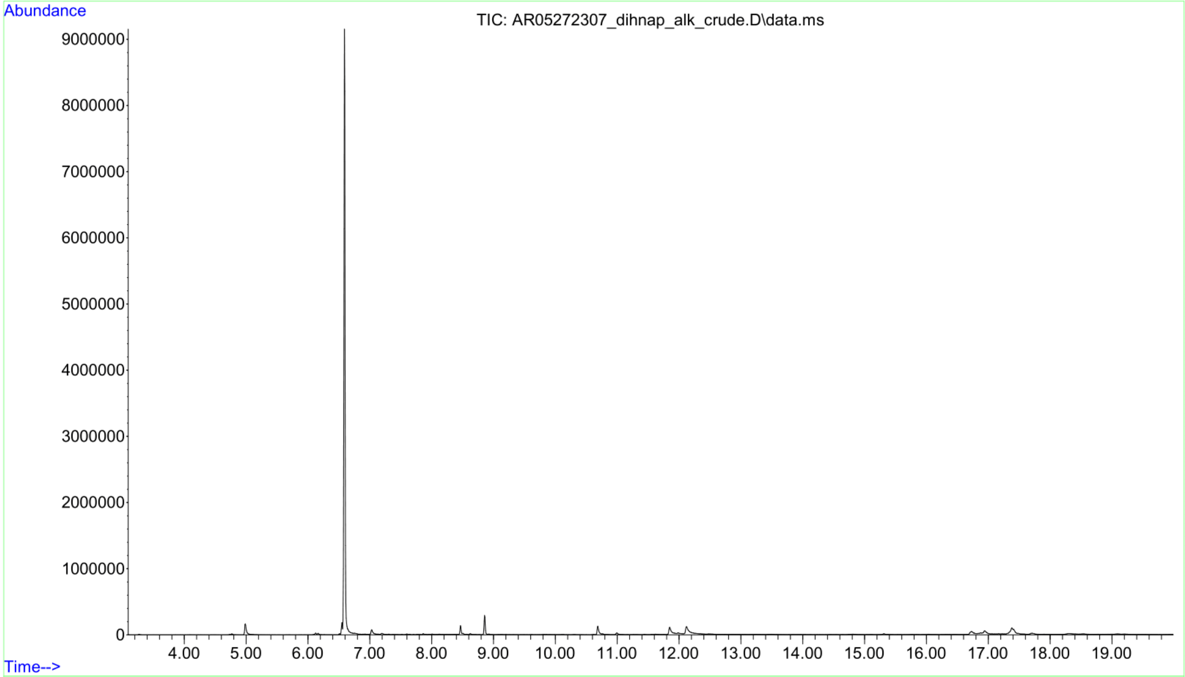

Abundance
TIC: AR05272307_dihnap_alk_crude.D\data.ms
9000000
8000000
7000000
6000000
5000000
4000000
3000000
2000000
1000000
0
4.00
5.00
6.00
7.00
8.00
9.00
10.00
11.00
12.00
13.00
14.00
15.00
16.00
17.00
18.00
19.00
Time-->

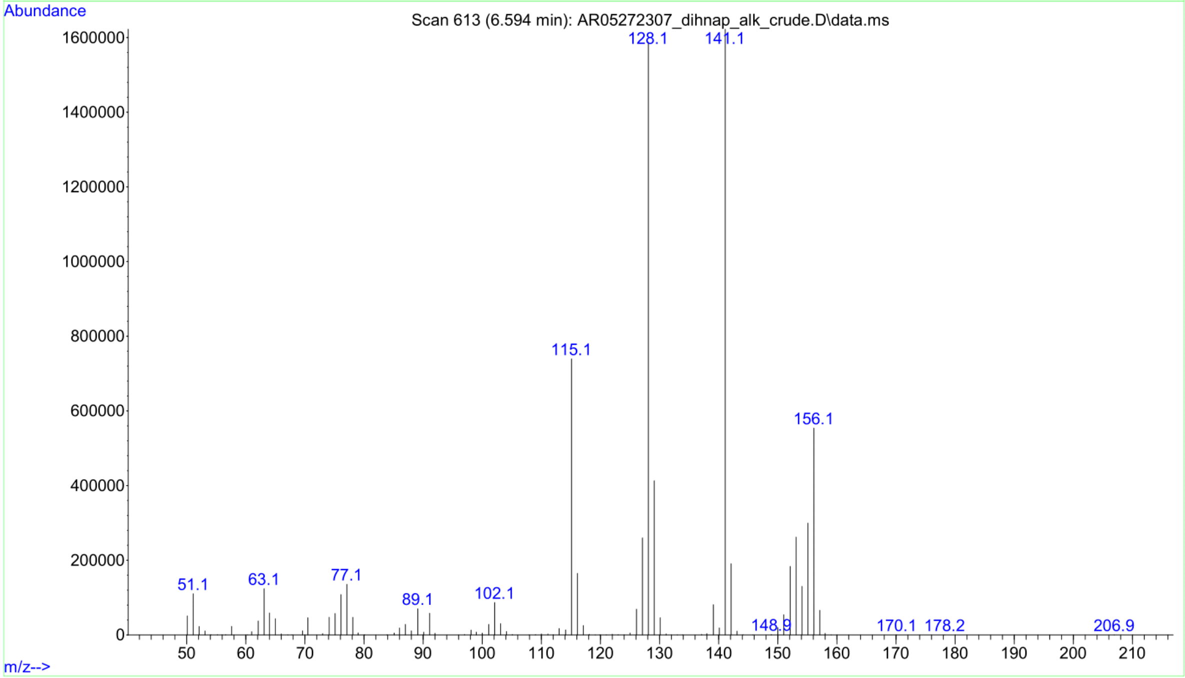

Abundance
Scan 613 (6.594 min): AR05272307_dihnap_alk_crude.D\data.ms
1600000
1400000
1200000
1000000
800000
600000
400000
200000
0
128.1
141.1
115.1
156.1
51.1
63.1
77.1
89.1
102.1
148.9
170.1
178.2
206.9
50
60
70
80
90
100
110
120
130
140
150
160
170
180
190
200
210
m/z-->

^{1}H, 500MHz, $CDCl_3$

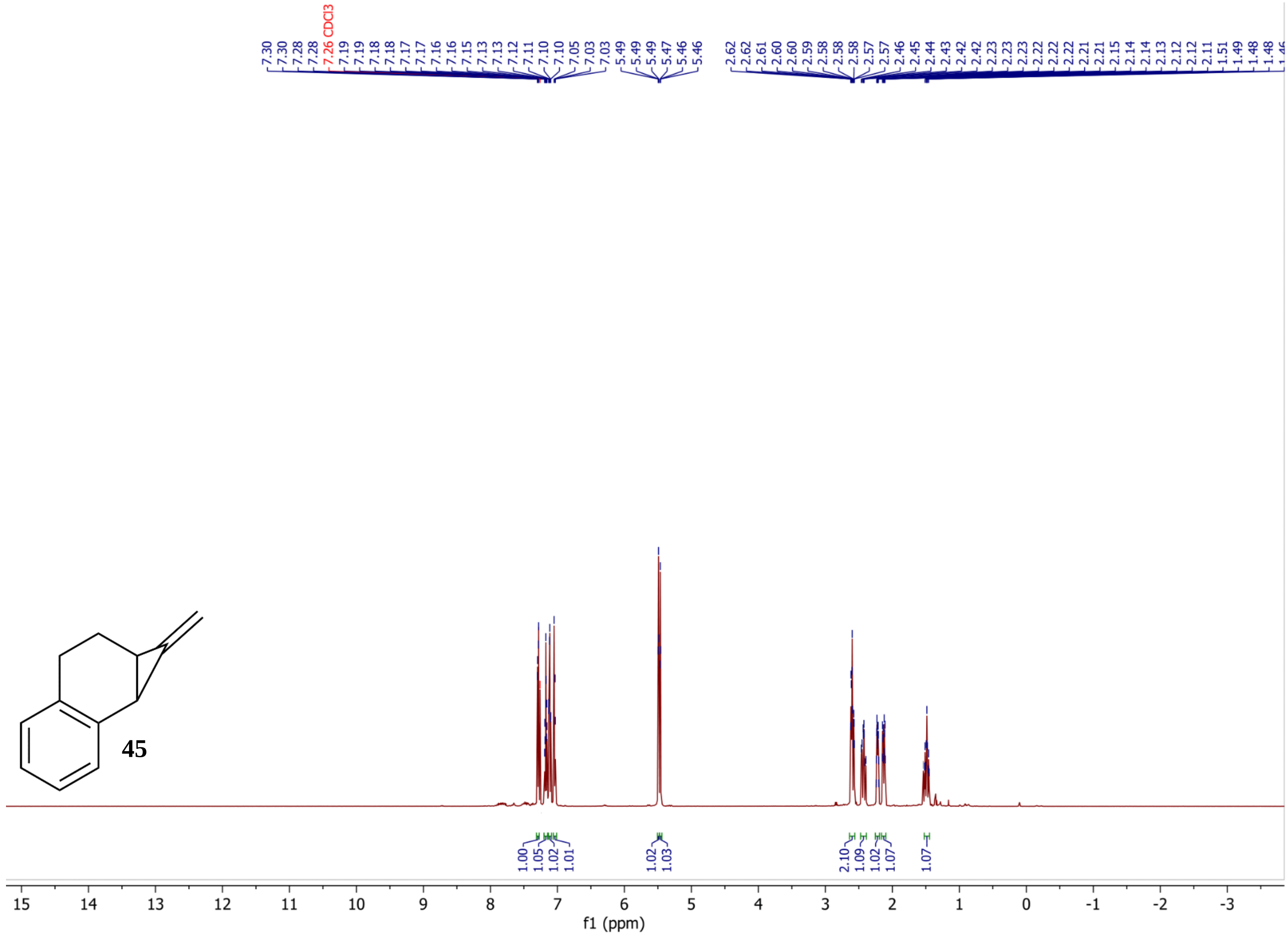

$^{13}C\{^{1}H\}$, 126 MHz, $CDCl_3$

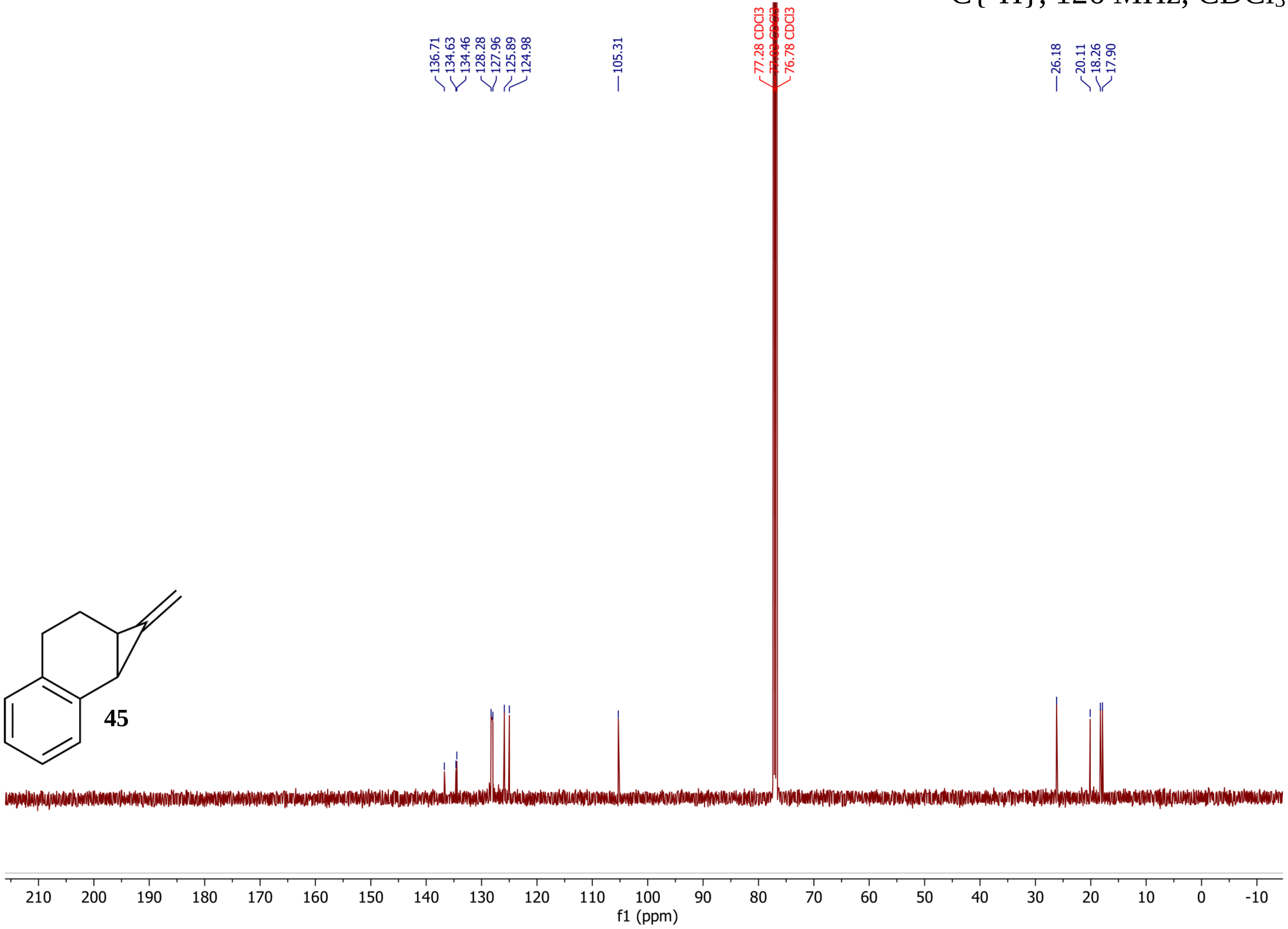

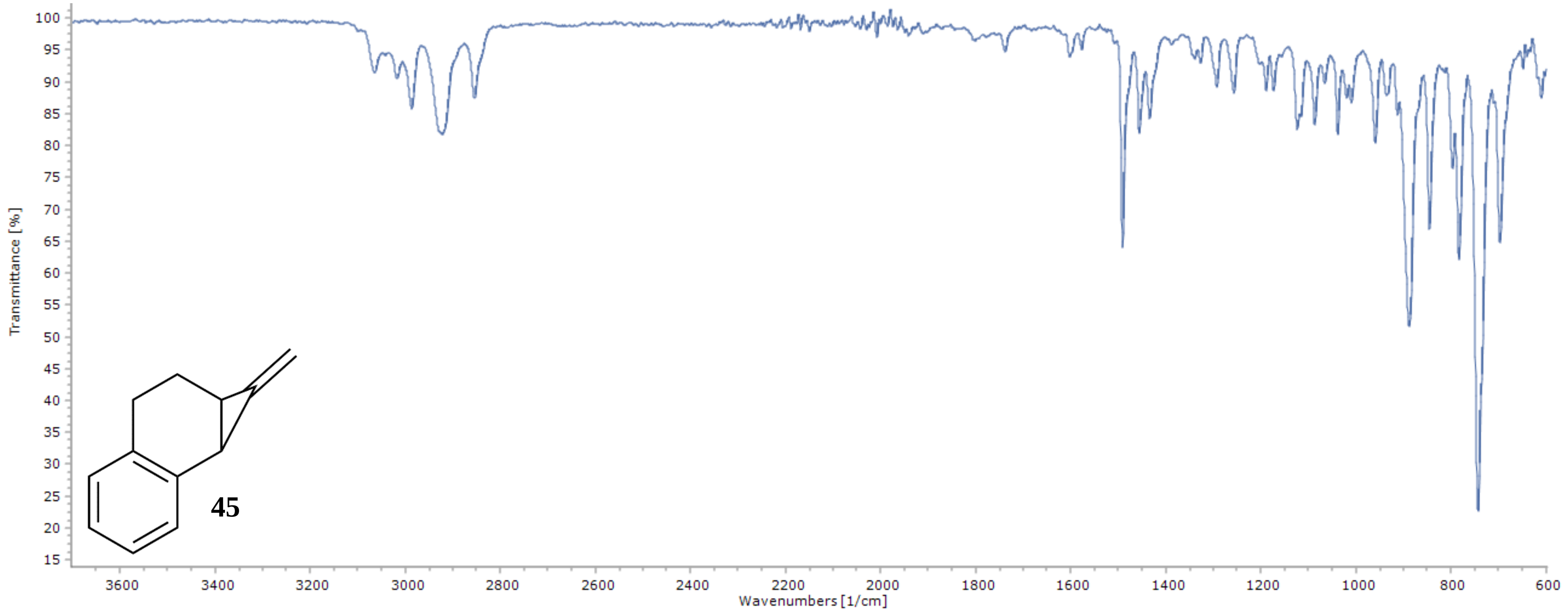

Transmittance [%]
Wavenumbers [1/cm]
45

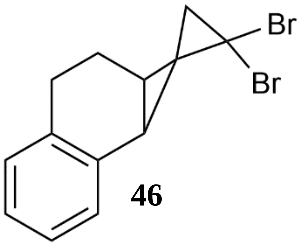

Br
Br

46

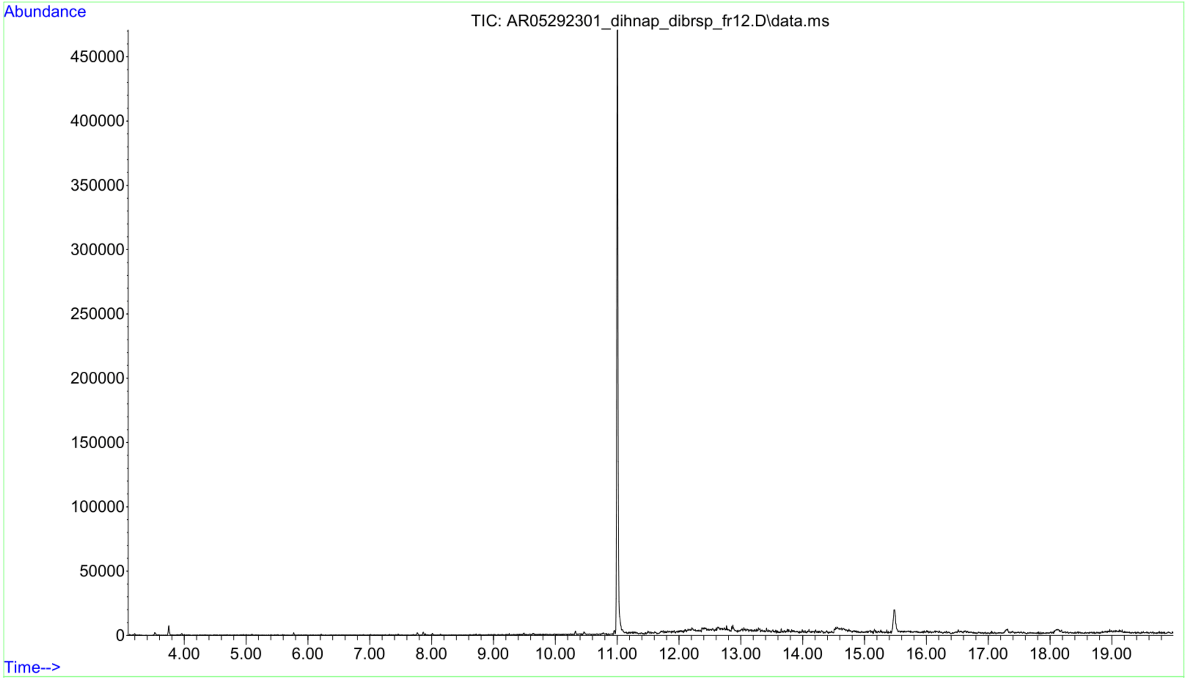

Abundance
TIC: AR05292301_dihnap_dibrsp_fr12.D\data.ms
450000
400000
350000
300000
250000
200000
150000
100000
50000
0
4.00 5.00 6.00 7.00 8.00 9.00 10.00 11.00 12.00 13.00 14.00 15.00 16.00 17.00 18.00 19.00
Time-->

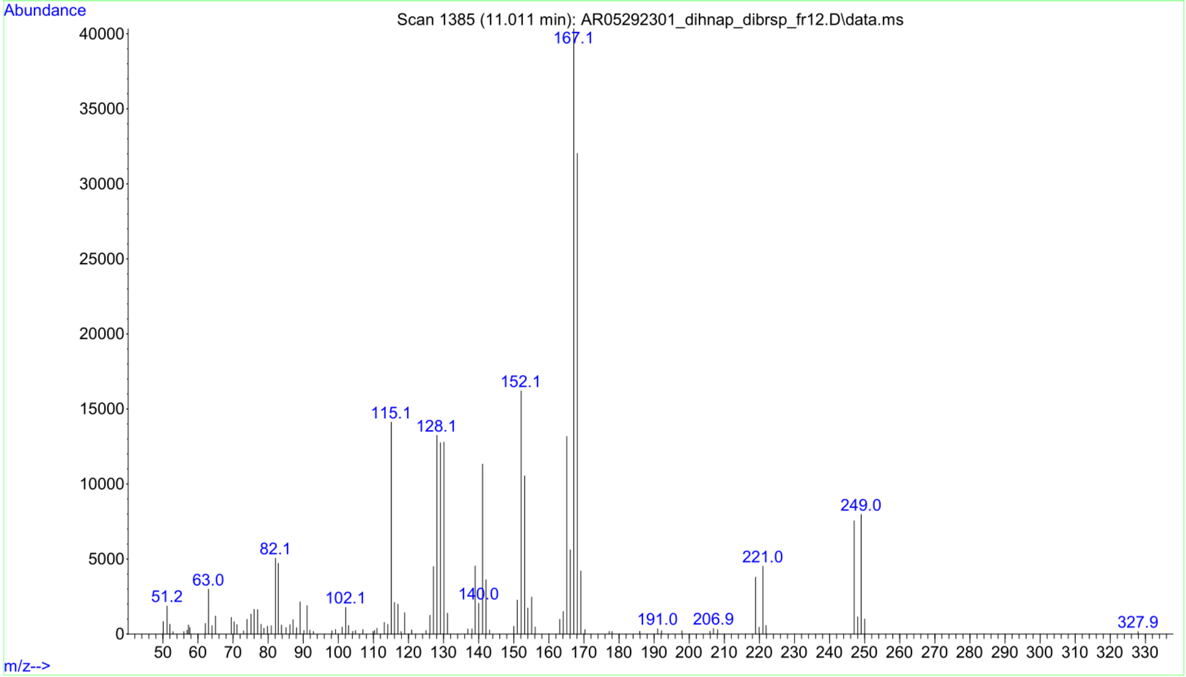

Abundance
Scan 1385 (11.011 min): AR05292301_dihnap_dibrsp_fr12.D\data.ms
40000
35000
30000
25000
20000
15000
10000
5000
0
167.1
152.1
115.1
128.1
249.0
82.1
221.0
63.0
51.2
102.1
140.0
191.0
206.9
327.9
50 60 70 80 90 100 110 120 130 140 150 160 170 180 190 200 210 220 230 240 250 260 270 280 290 300 310 320 330
m/z-->

^{1}H, 500MHz, $CDCl_3$

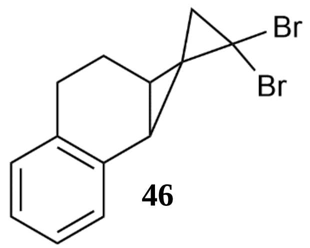

46

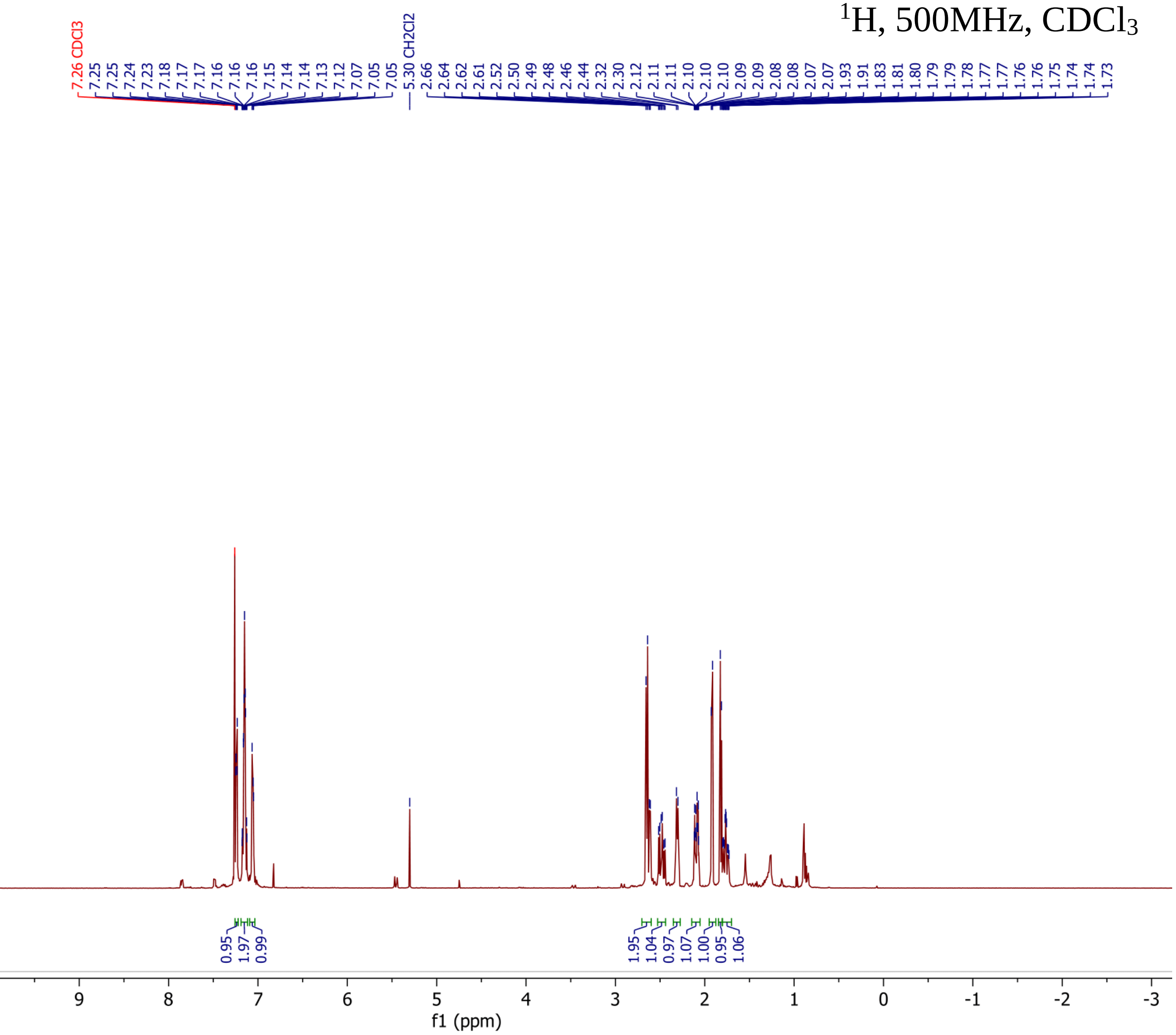

$^{13}C\{^{1}H\}$, 126 MHz, $CDCl_3$

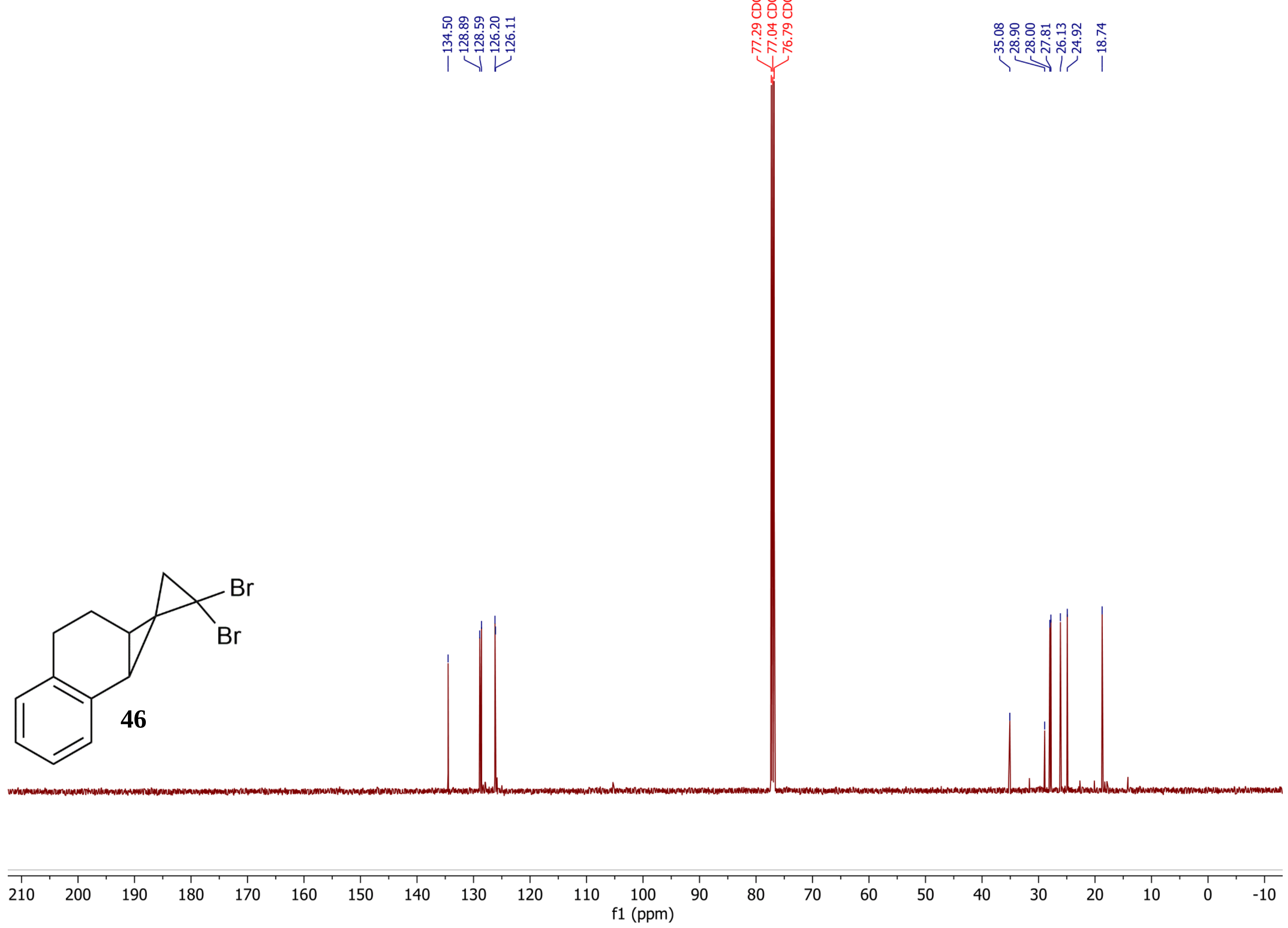

46

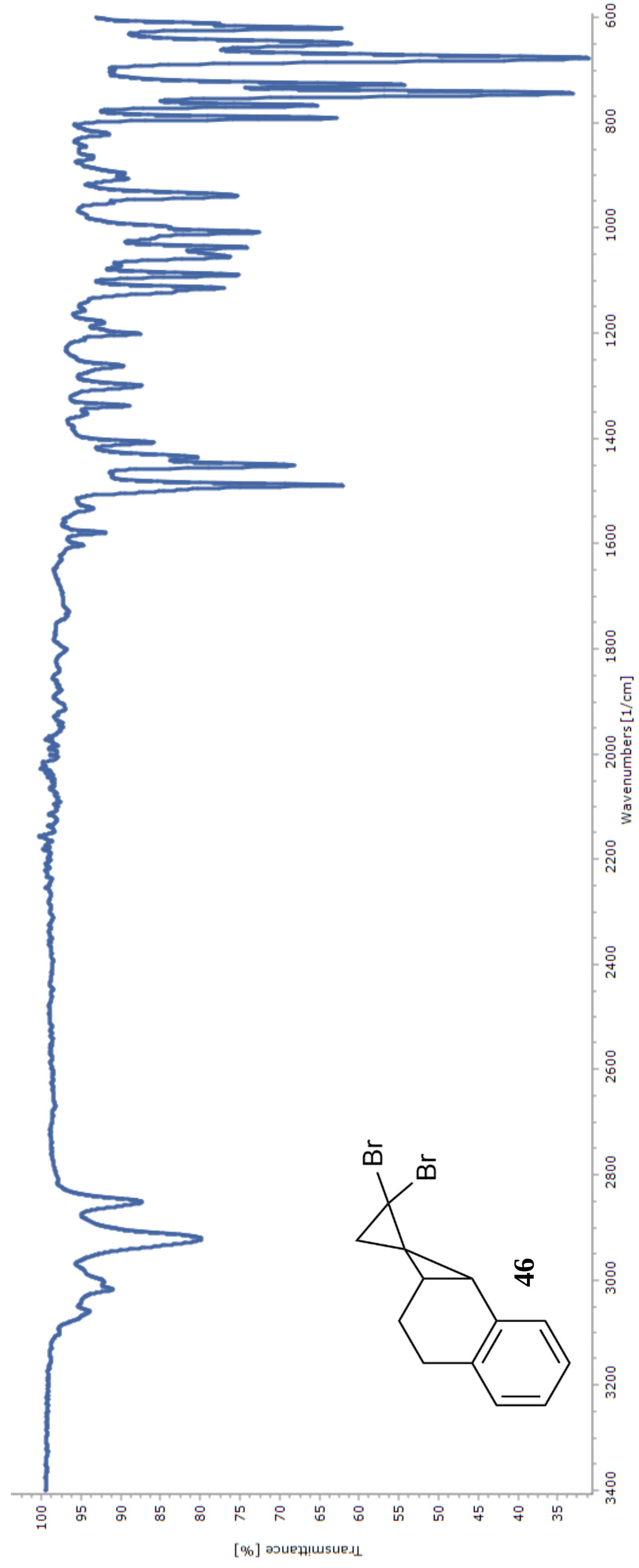

Transmittance [%]
Wavenumbers [1/cm]
46
Br
Br

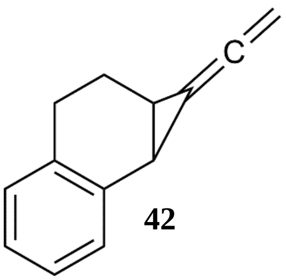

42

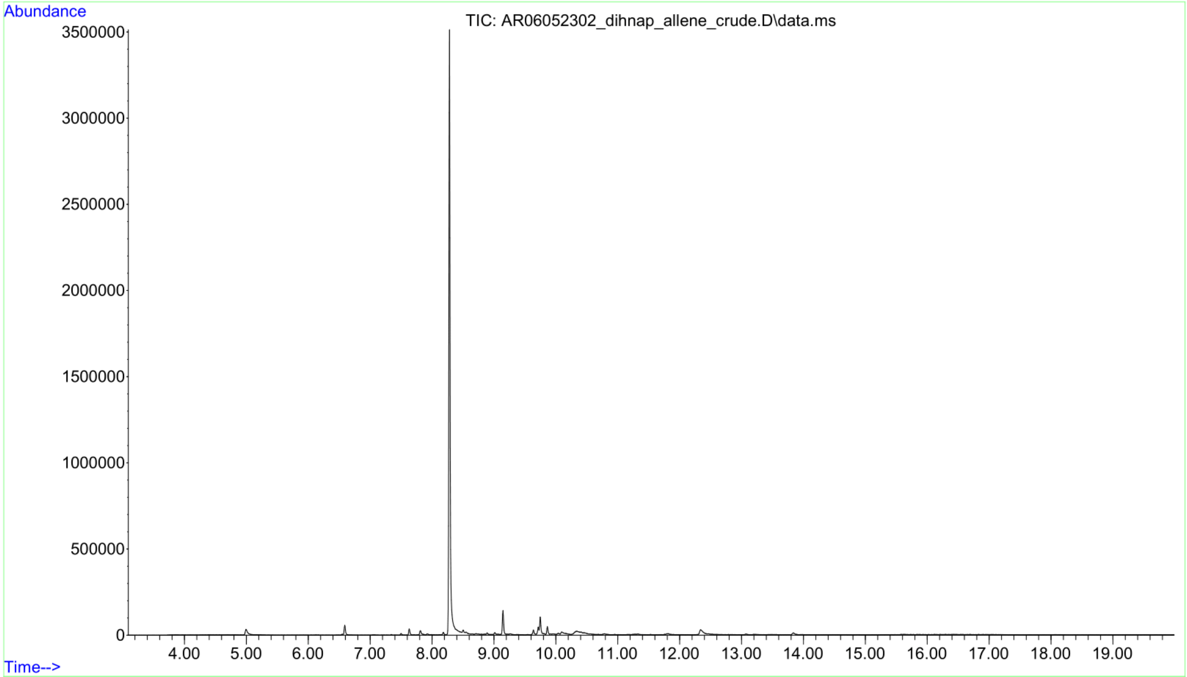

Abundance
TIC: AR06052302_dihnap_allene_crude.D\data.ms
3500000
3000000
2500000
2000000
1500000
1000000
500000
0
4.00 5.00 6.00 7.00 8.00 9.00 10.00 11.00 12.00 13.00 14.00 15.00 16.00 17.00 18.00 19.00
Time-->

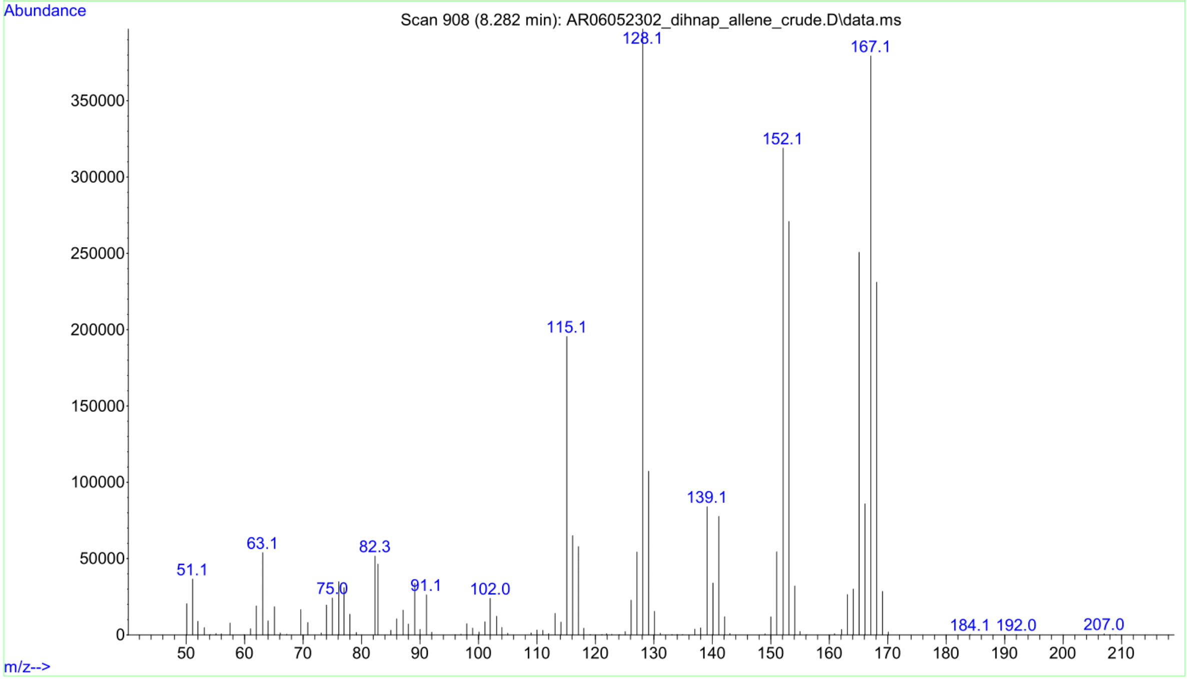

Abundance
Scan 908 (8.282 min): AR06052302_dihnap_allene_crude.D\data.ms
128.1
167.1
152.1
115.1
139.1
51.1
63.1
75.0
82.3
91.1
102.0
184.1
192.0
207.0
350000
300000
250000
200000
150000
100000
50000
0
50 60 70 80 90 100 110 120 130 140 150 160 170 180 190 200 210
m/z-->

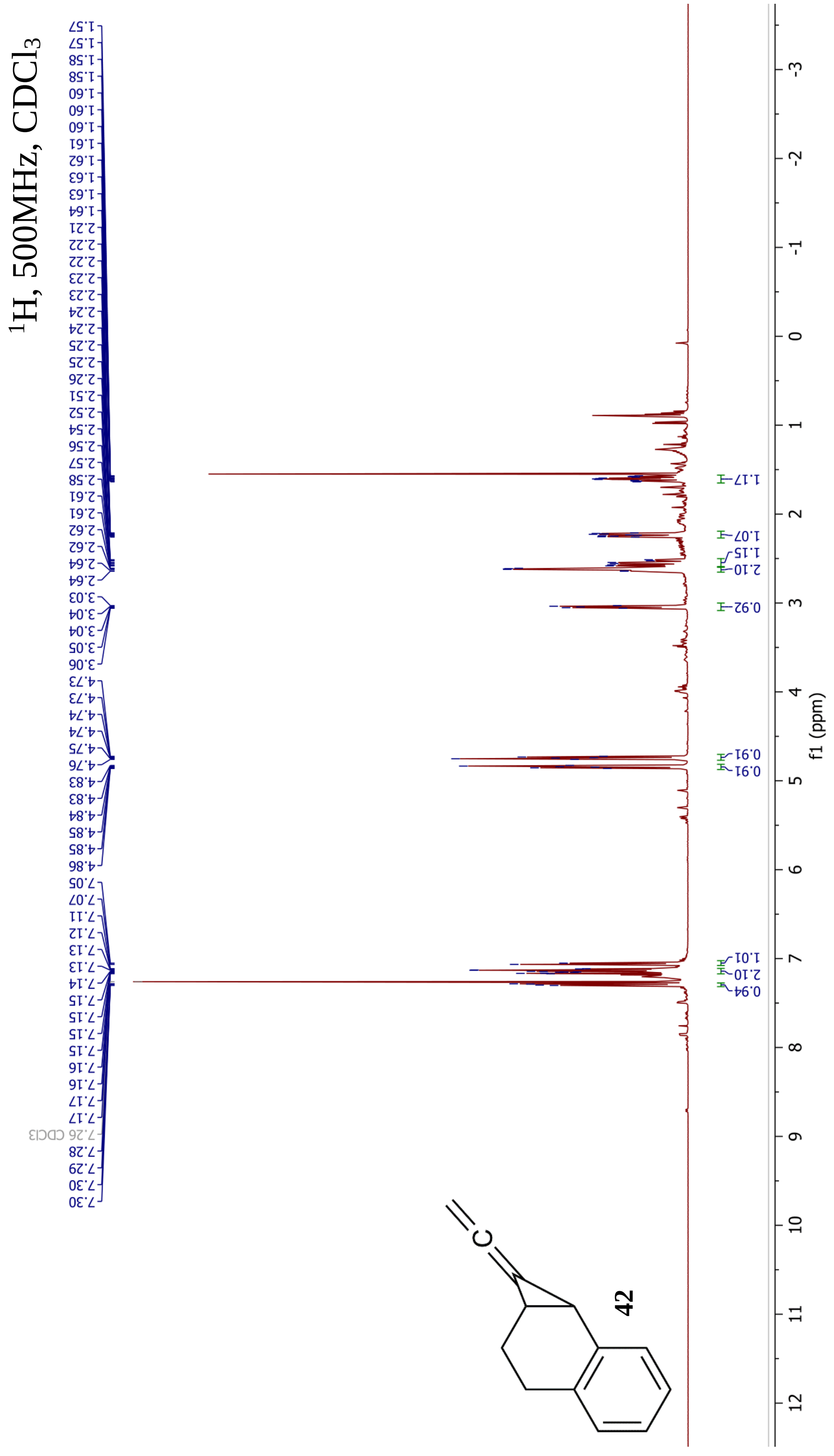
^{1}H, 500MHz, $CDCl_3$
42
C
1.57
1.57
1.58
1.58
1.60
1.60
1.60
1.61
1.62
1.63
1.63
1.64
2.21
2.22
2.22
2.23
2.23
2.24
2.24
2.25
2.25
2.26
2.51
2.52
2.54
2.56
2.57
2.58
2.61
2.61
2.62
2.62
2.64
2.64
3.03
3.04
3.04
3.05
3.06
4.73
4.73
4.74
4.74
4.75
4.76
4.83
4.83
4.84
4.85
4.85
4.86
7.05
7.07
7.11
7.12
7.13
7.13
7.14
7.15
7.15
7.15
7.15
7.16
7.16
7.17
7.17
7.26 CDCl3
7.28
7.29
7.30
7.30
1.17
1.07
1.15
2.10
0.92
0.91
0.91
1.01
2.10
0.94
12
11
10
9
8
7
6
5
4
3
2
1
0
-1
-2
-3
f1 (ppm)

^1H, 500MHz, C_6D_6

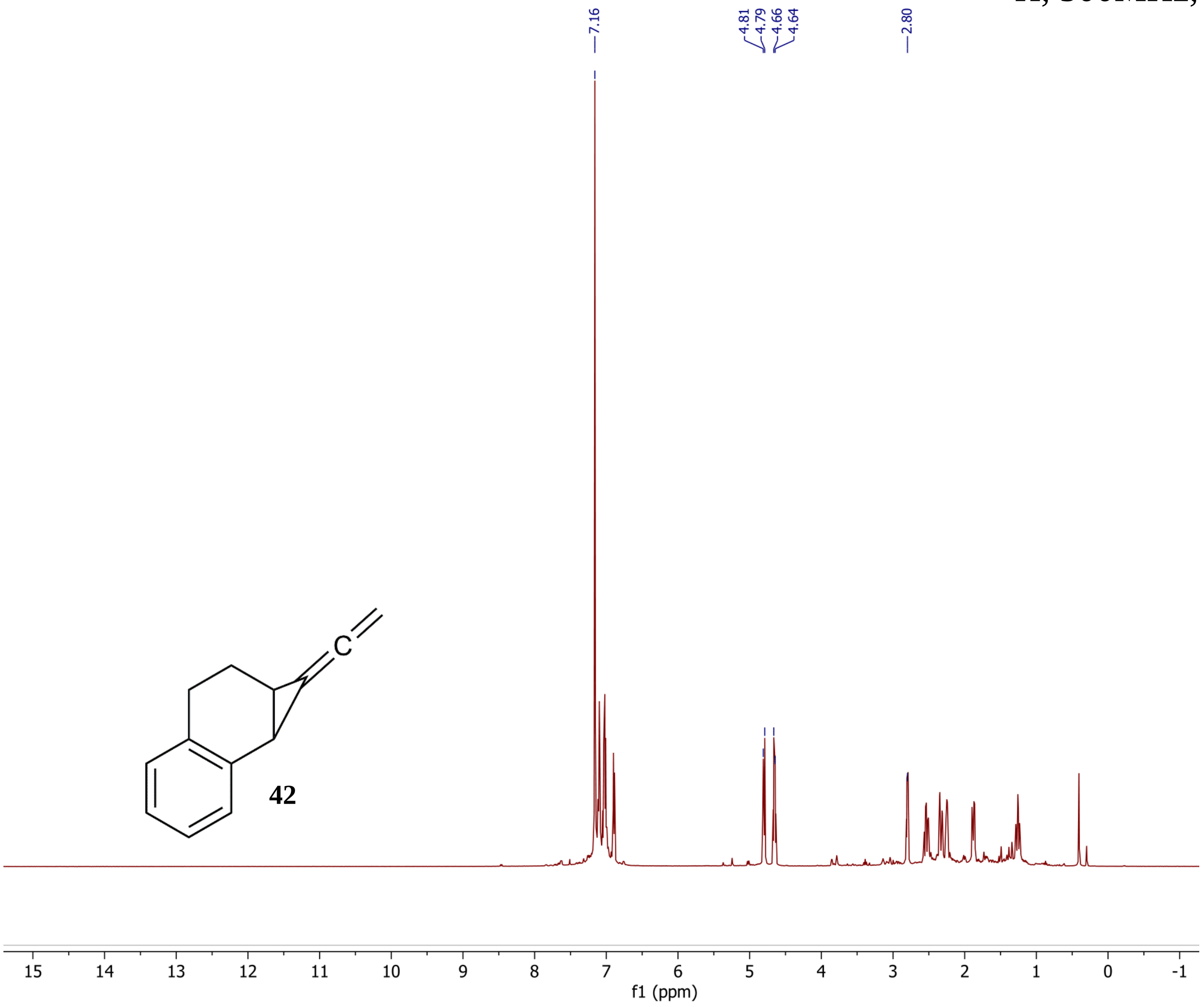

42

$^{13}C\{^1H\}$, 126 MHz, $CDCl_3$

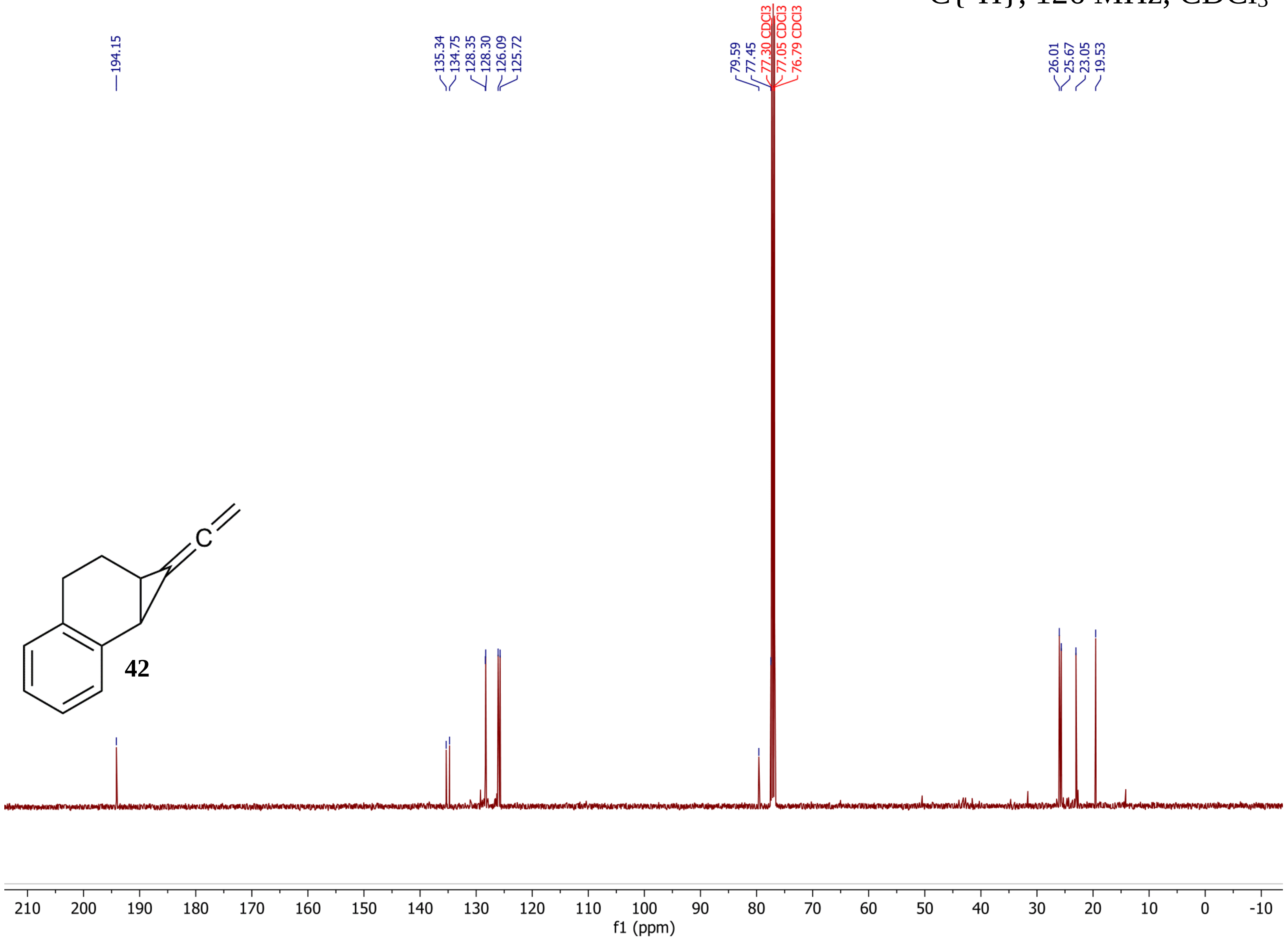

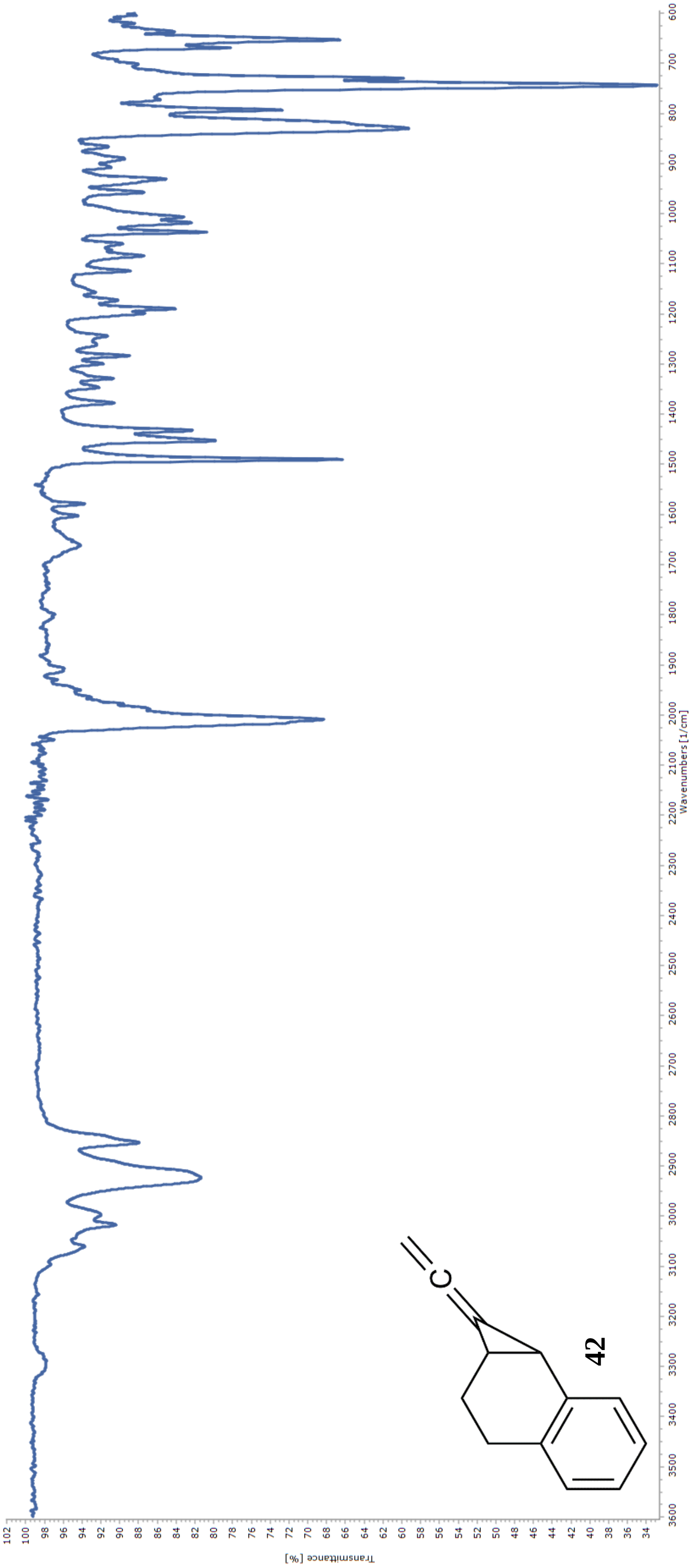

Transmittance [%]
Wavenumbers [1/cm]
C
42

^{1}H, 500MHz, CDCl$_3$

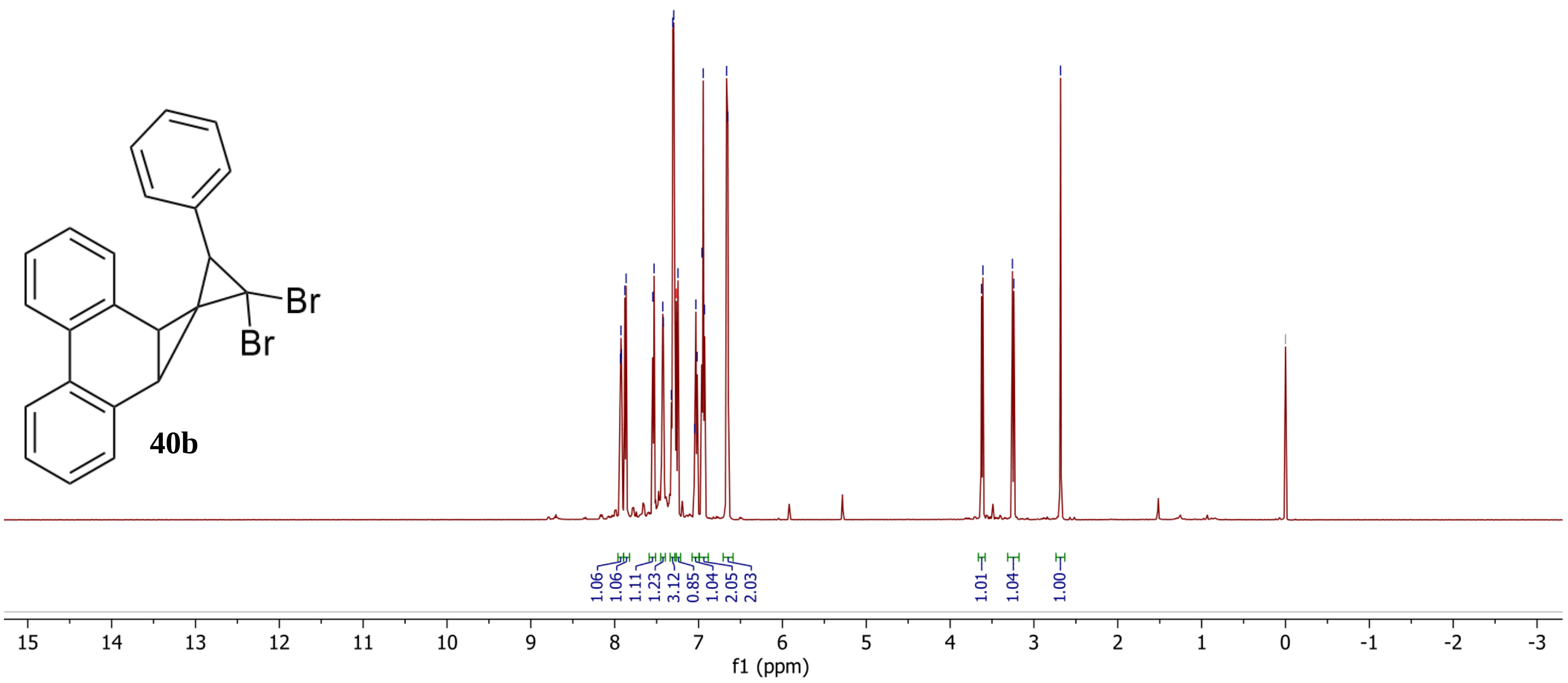

40b

$^{13}C\{^{1}H\}$, 126 MHz, $CDCl_3$

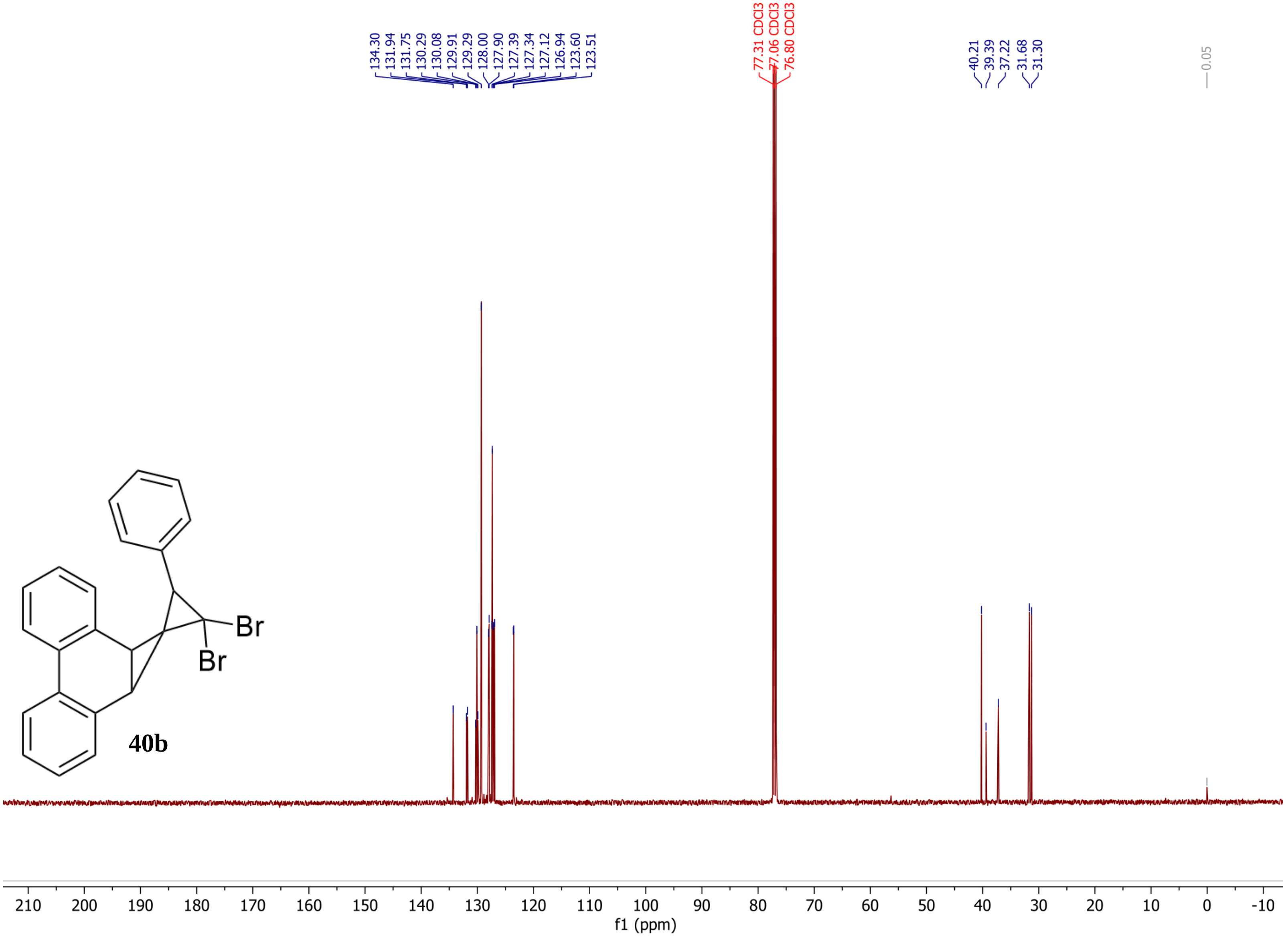

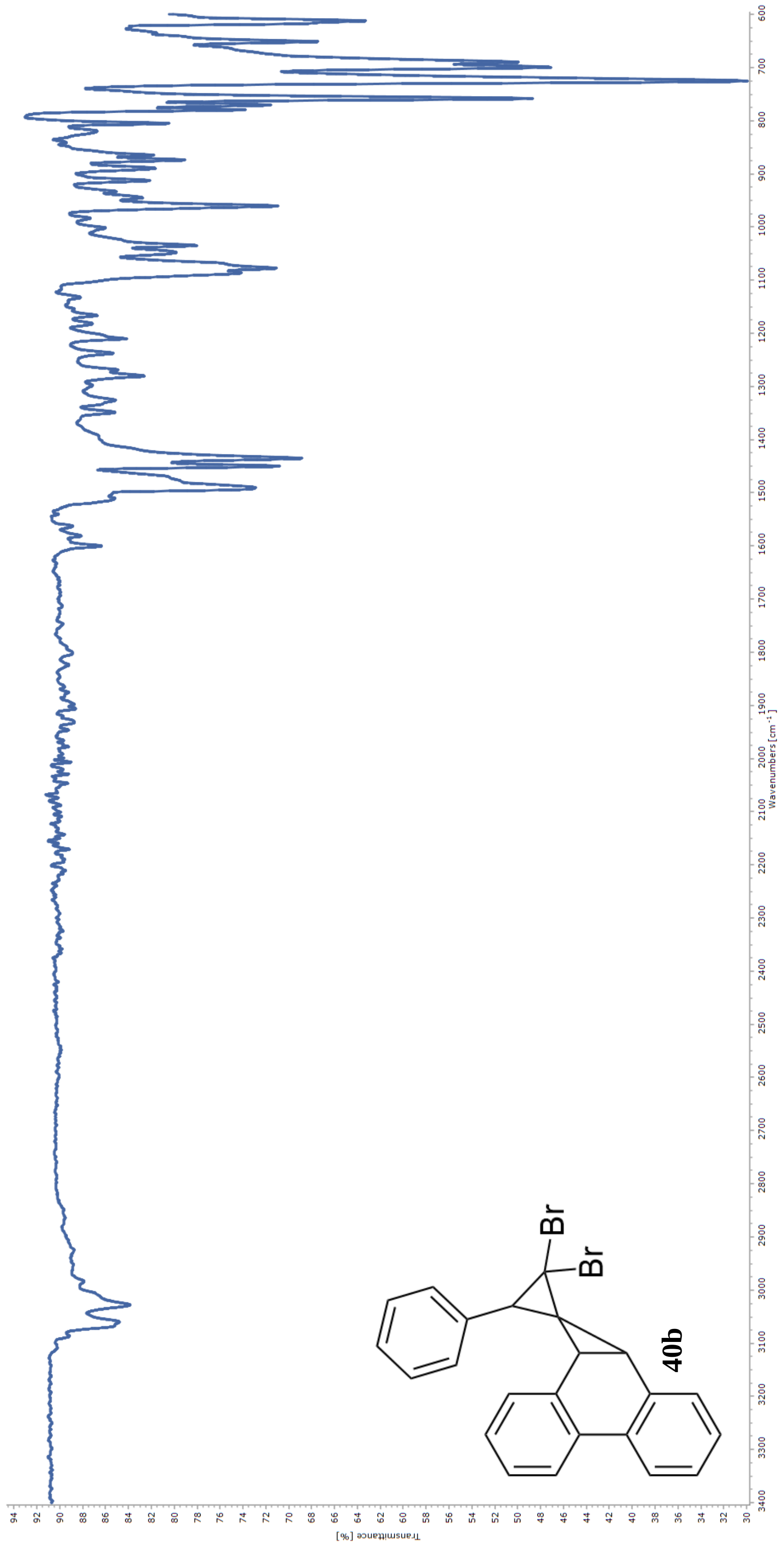
40b
Br
Br
Transmittance [%]
Wavenumbers [cm-1]

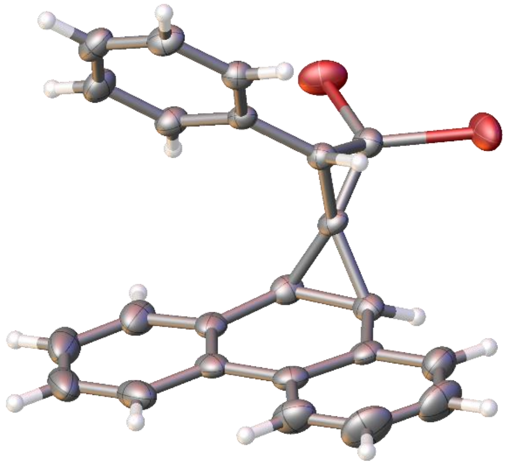

Empirical formula	$C_{46}H_{32}Br_4$
Formula weight	904.381
Temperature/K	173
Crystal system	Orthorhombic
Space group	P212121
a/Å	9.9462(7)
b/Å	16.2497(16)
c/Å	22.644(2)
α/°	90
β/°	90
γ/°	90
Volume/$Å^3$	3659.8(6)
Z	4
$\rho_{calc}g/cm^3$	1.641
μ/mm^{-1}	4.431
F(000)	1789.0
Crystal size/mm^3	0.28 × 0.15 × 0.12
Radiation	MoKα (λ = 0.71073)
2Θ range for data collection/°	5.96 to 52.8
Index ranges	$-12 \leq h \leq 12$, $-20 \leq k \leq 20$, $-28 \leq l \leq 27$
Reflections collected	46409
Independent reflections	7474 [Rint = 0.0429, Rsigma = 0.0379]
Data/restraints/parameters	7474/0/451
Goodness-of-fit on F^2	1.021
Final R indexes [I>=2σ (I)]	R1 = 0.0272, wR2 = 0.0474
Final R indexes [all data]	R1 = 0.0368, wR2 = 0.0499
Largest diff. peak/hole / e $Å^{-3}$	0.57/-0.40
Flack parameter	0.023(4)
CCDC Number	2267367

^{1}H, 500MHz, $CDCl_3$

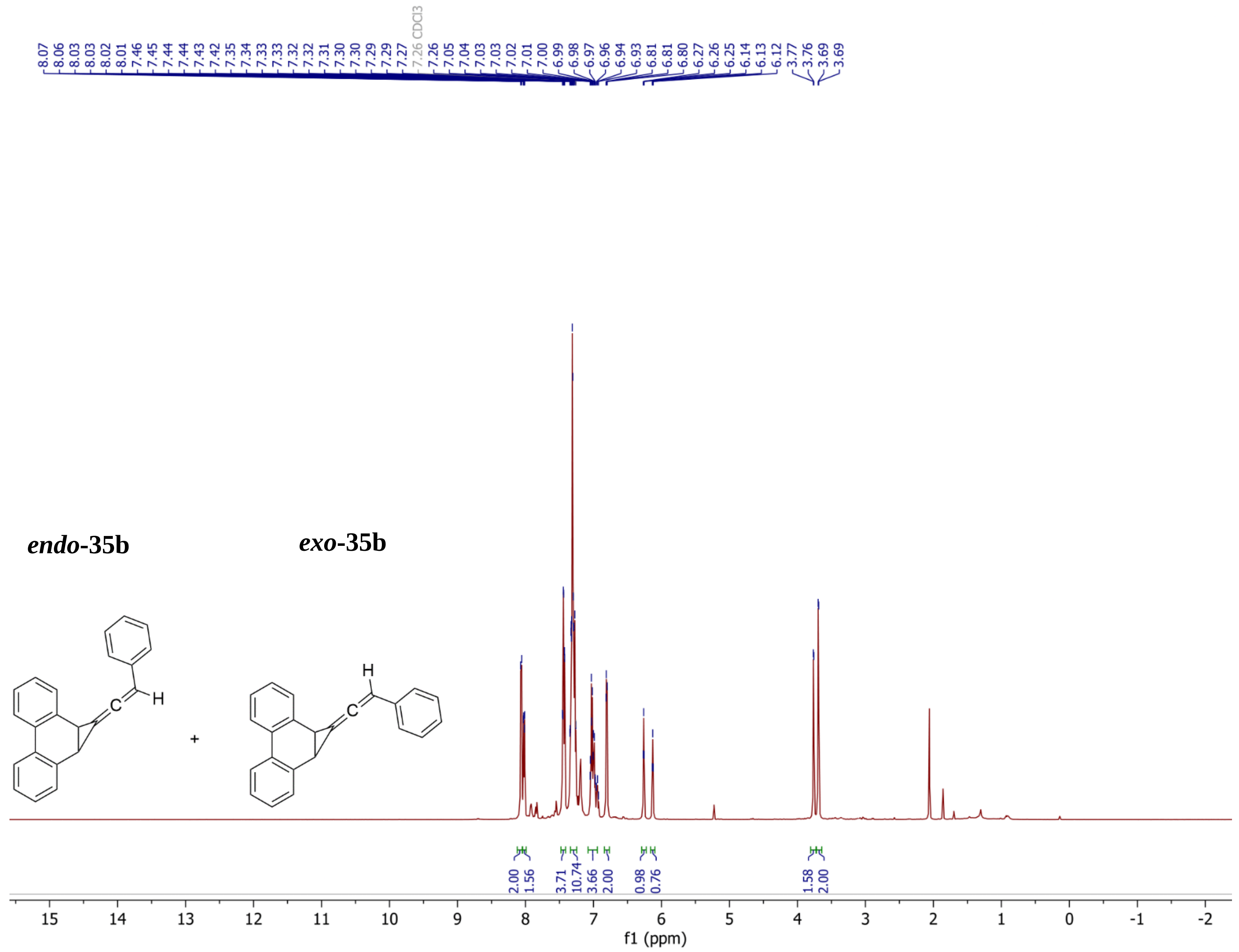

$^{13}C\{^{1}H\}$, 126 MHz, $CDCl_3$

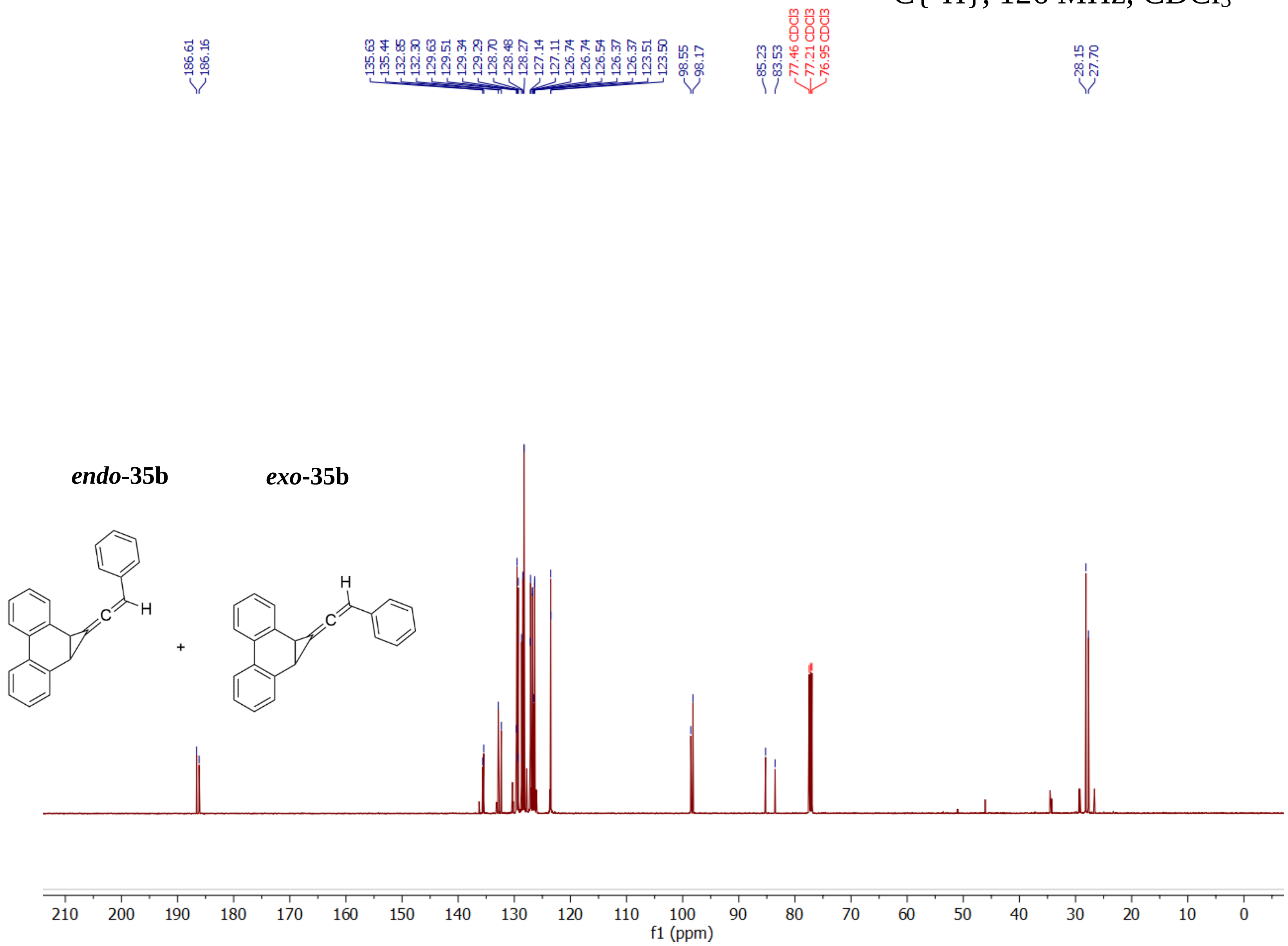

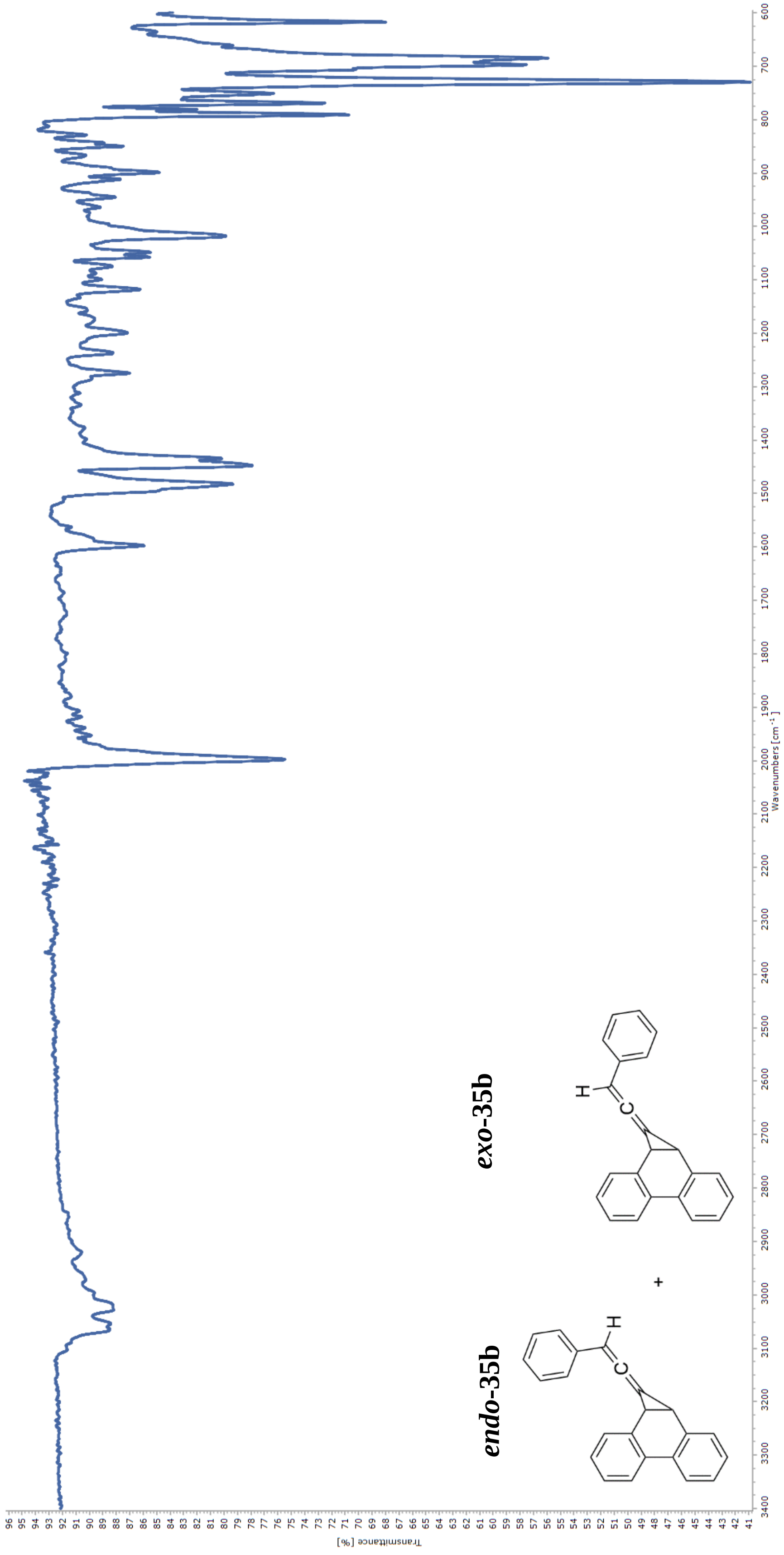
endo-35b
exo-35b
+
Transmittance [%]
Wavenumbers [cm-1]

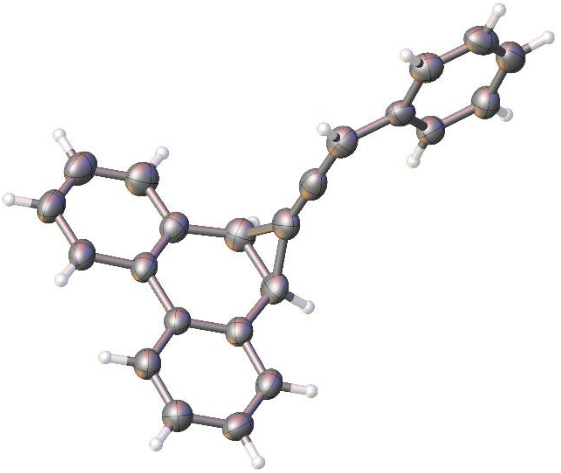

Empirical formula	$C_{23}H_{16}$
Formula weight	292.36
Temperature/K	173.00
Crystal system	Monoclinic
Space group	P21
a/Å	5.26840(10)
b/Å	13.0725(4)
c/Å	11.6144(3)
α/°	90
β/°	102.527(2)
γ/°	90
Volume/Å^3	780.85(4)
Z	2
ρ_{calc}g/cm^3	1.243
μ/mm^{-1}	0.070
F(000)	308.0
Crystal size/mm^3	0.2 × 0.088 × 0.085
Radiation	MoKα (λ = 0.71073)
2Θ range for data collection/°	6.234 to 55.076
Index ranges	-6 ≤ h ≤ 6, -17 ≤ k ≤ 16, -15 ≤ l ≤ 15
Reflections collected	17718
Independent reflections	3560 [Rint = 0.0580, Rsigma = 0.0545]
Data/restraints/parameters	3560/1/208
Goodness-of-fit on F^2	1.091
Final R indexes [I>=2σ (I)]	R1 = 0.0578, wR2 = 0.1291
Final R indexes [all data]	R1 = 0.1272, wR2 = 0.1739
Largest diff. peak/hole / e Å^{-3}	0.22/-0.25
Flack parameter	0.4(10)
CCDC Number	2261481

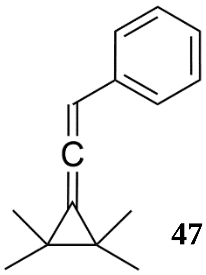

47

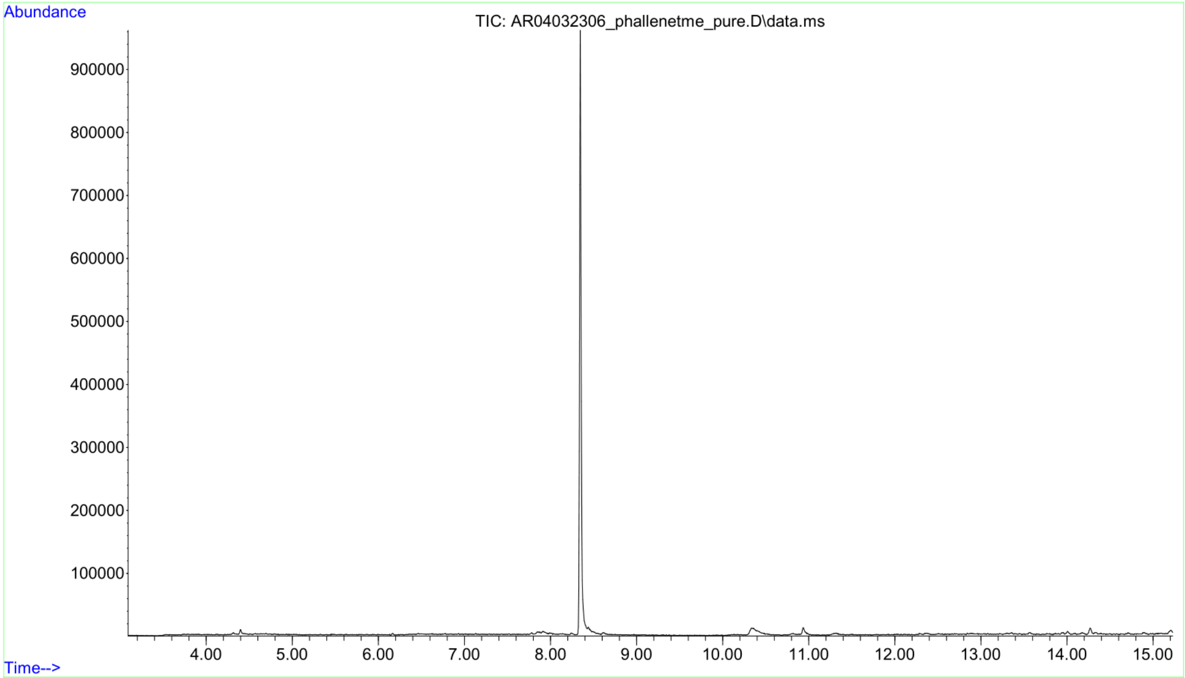

Abundance
TIC: AR04032306_phallenetme_pure.D\data.ms
900000
800000
700000
600000
500000
400000
300000
200000
100000
Time-->
4.00
5.00
6.00
7.00
8.00
9.00
10.00
11.00
12.00
13.00
14.00
15.00

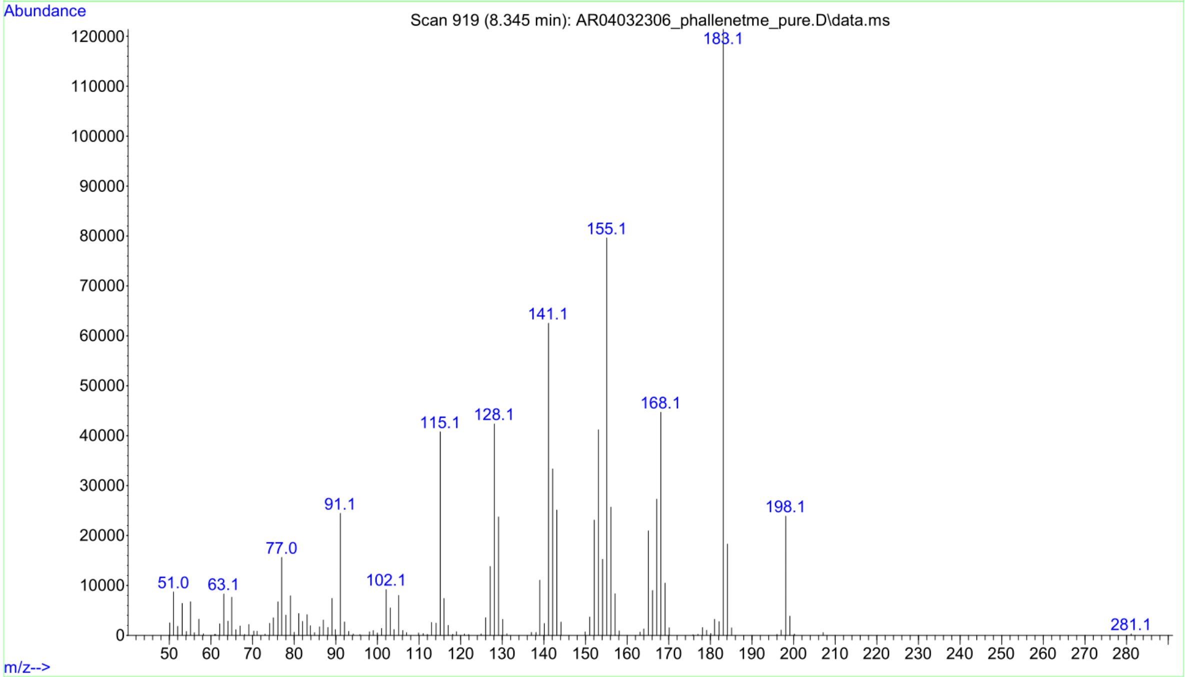

Abundance
Scan 919 (8.345 min): AR04032306_phallenetme_pure.D\data.ms
183.1
155.1
141.1
168.1
115.1
128.1
91.1
198.1
77.0
51.0
63.1
102.1
281.1
m/z-->

^1H, 500MHz, $CDCl_3$

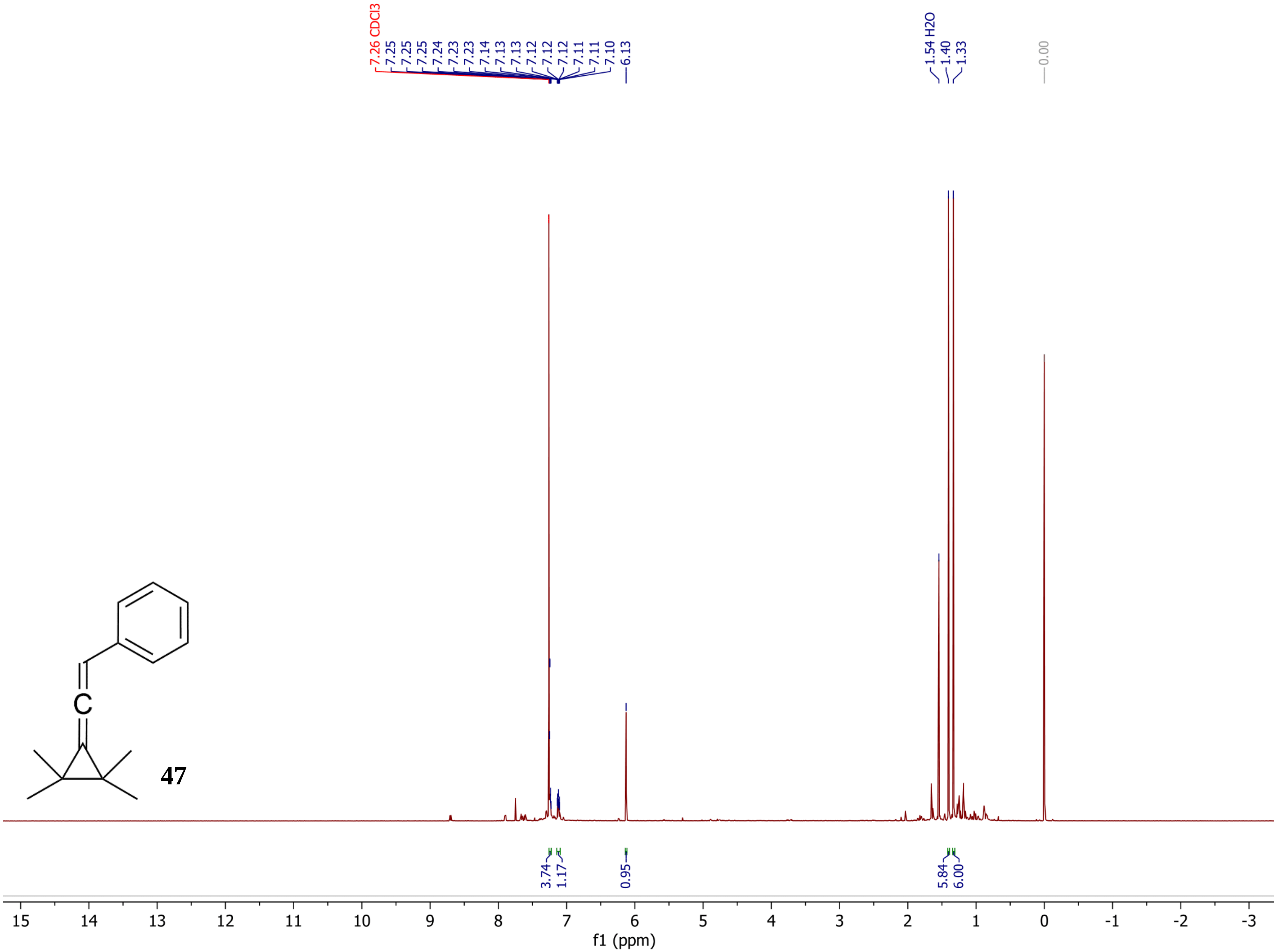

^{1}H, 500MHz, C_6D_6

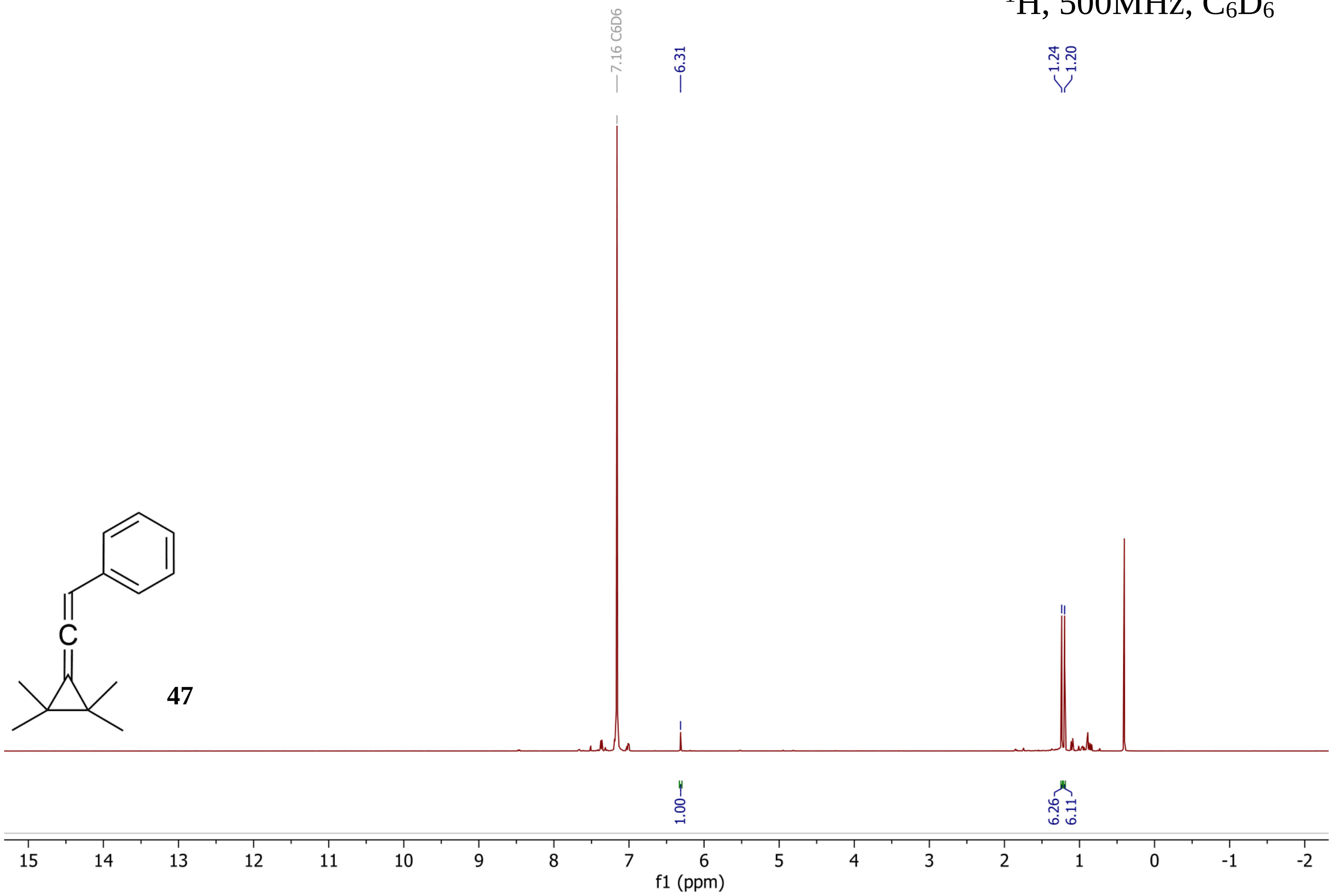

$^{13}C\{^{1}H\}$, 126 MHz, $CDCl_3$

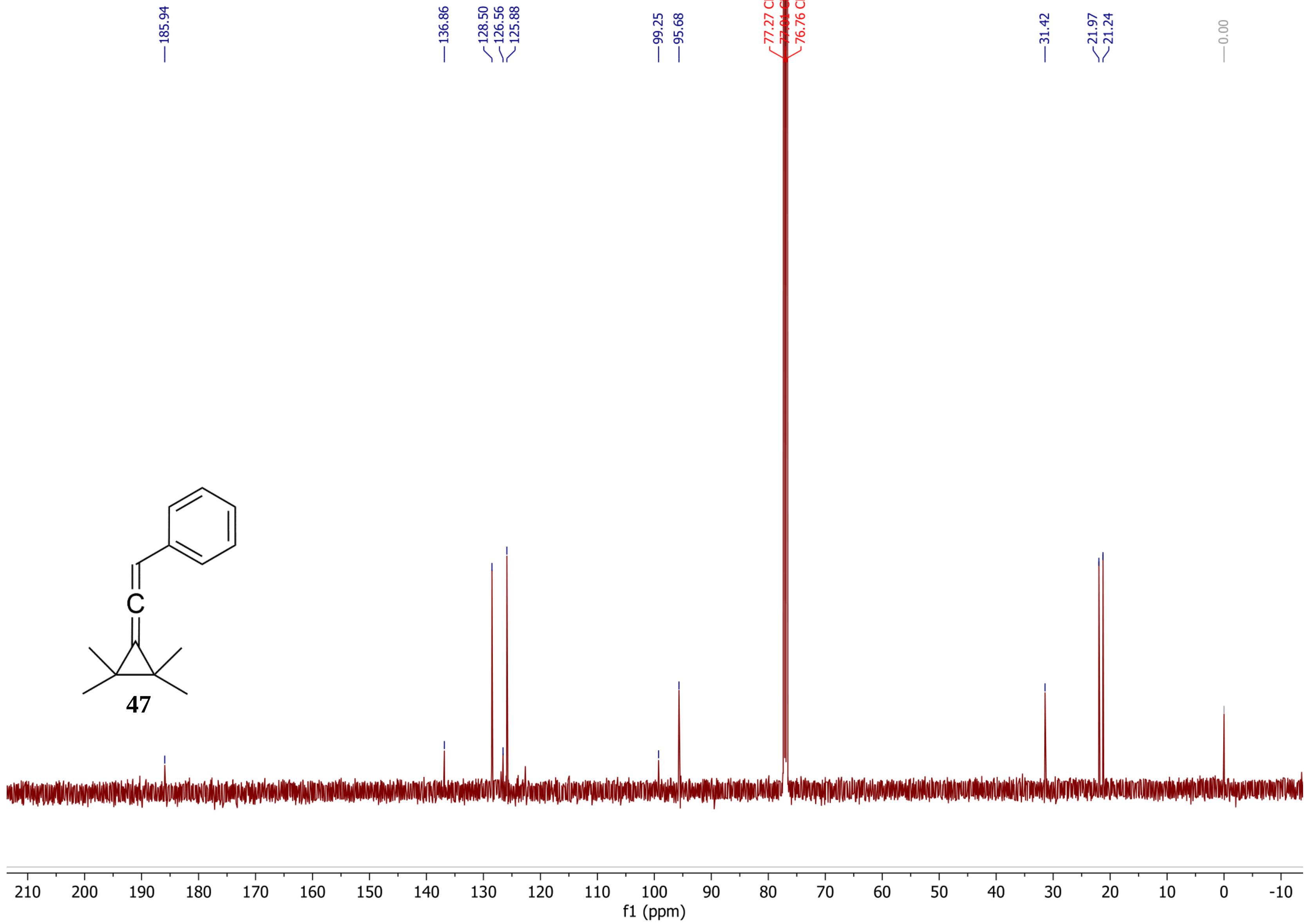

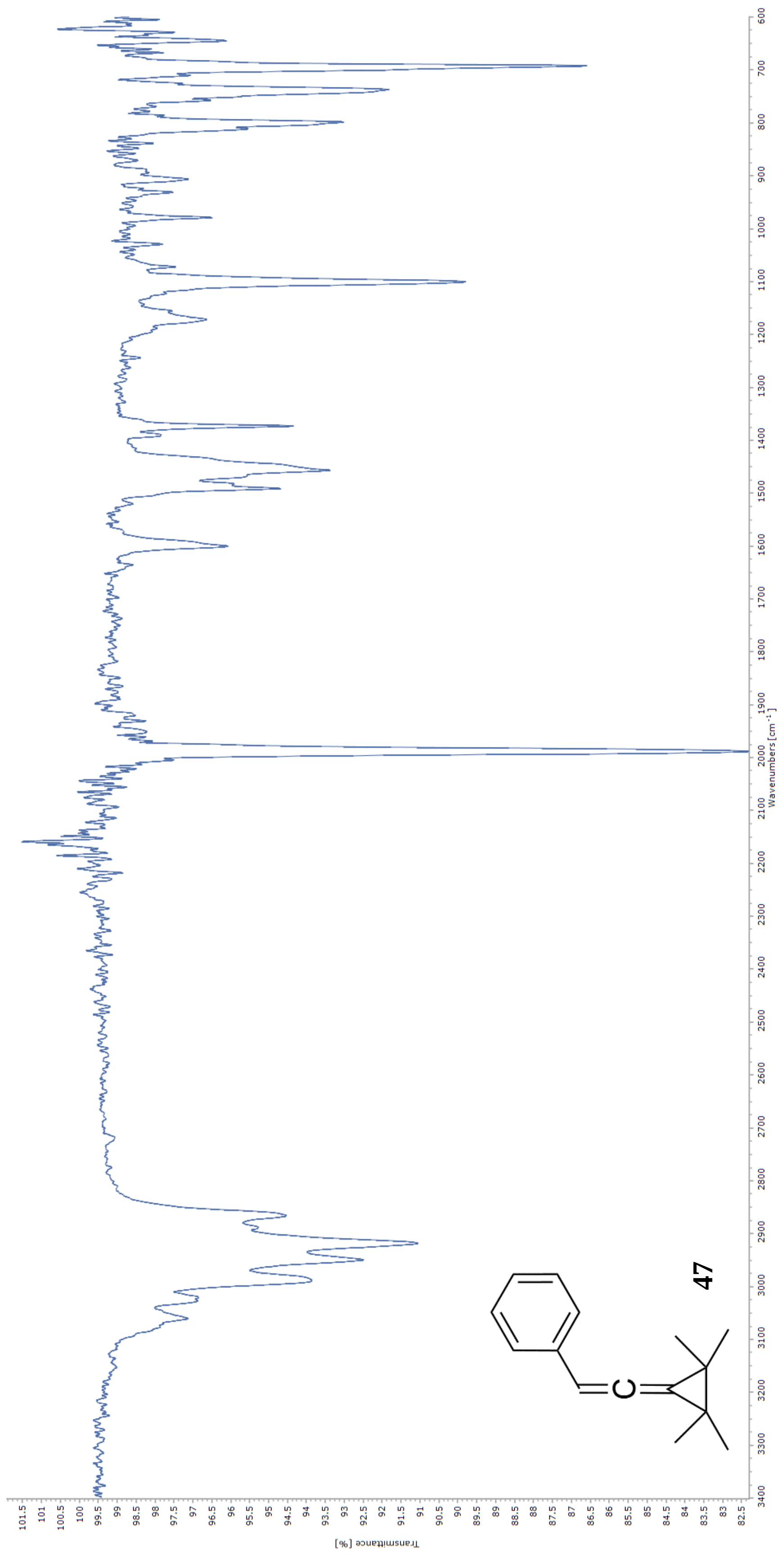

Transmittance [%]
Wavenumbers [cm-1]
47

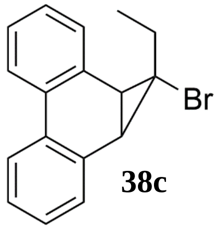
Br

38c

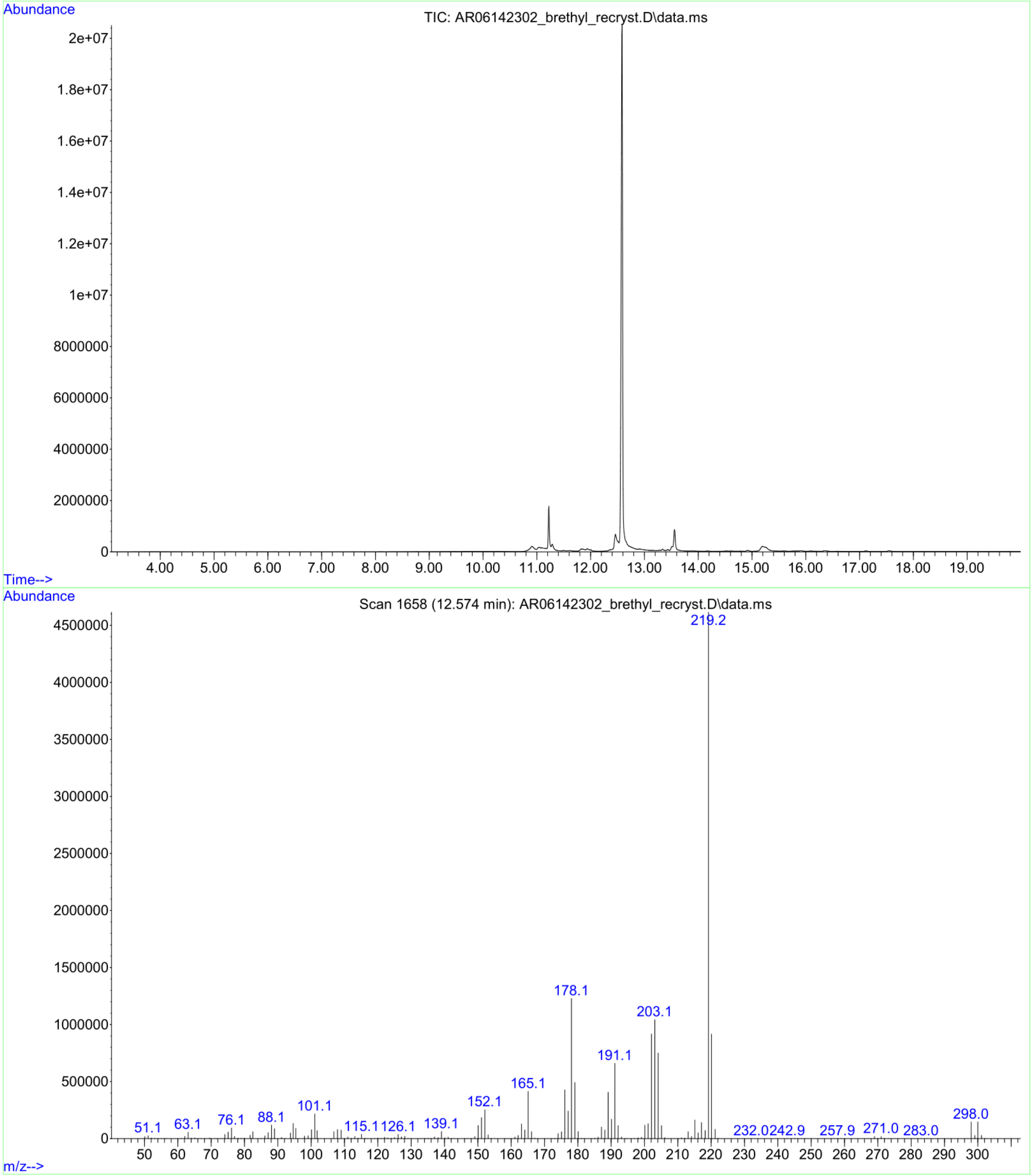
Abundance
TIC: AR06142302_brethyl_recryst.D\data.ms
Time-->
Abundance
Scan 1658 (12.574 min): AR06142302_brethyl_recryst.D\data.ms
219.2
178.1
203.1
191.1
165.1
152.1
101.1
298.0
m/z-->

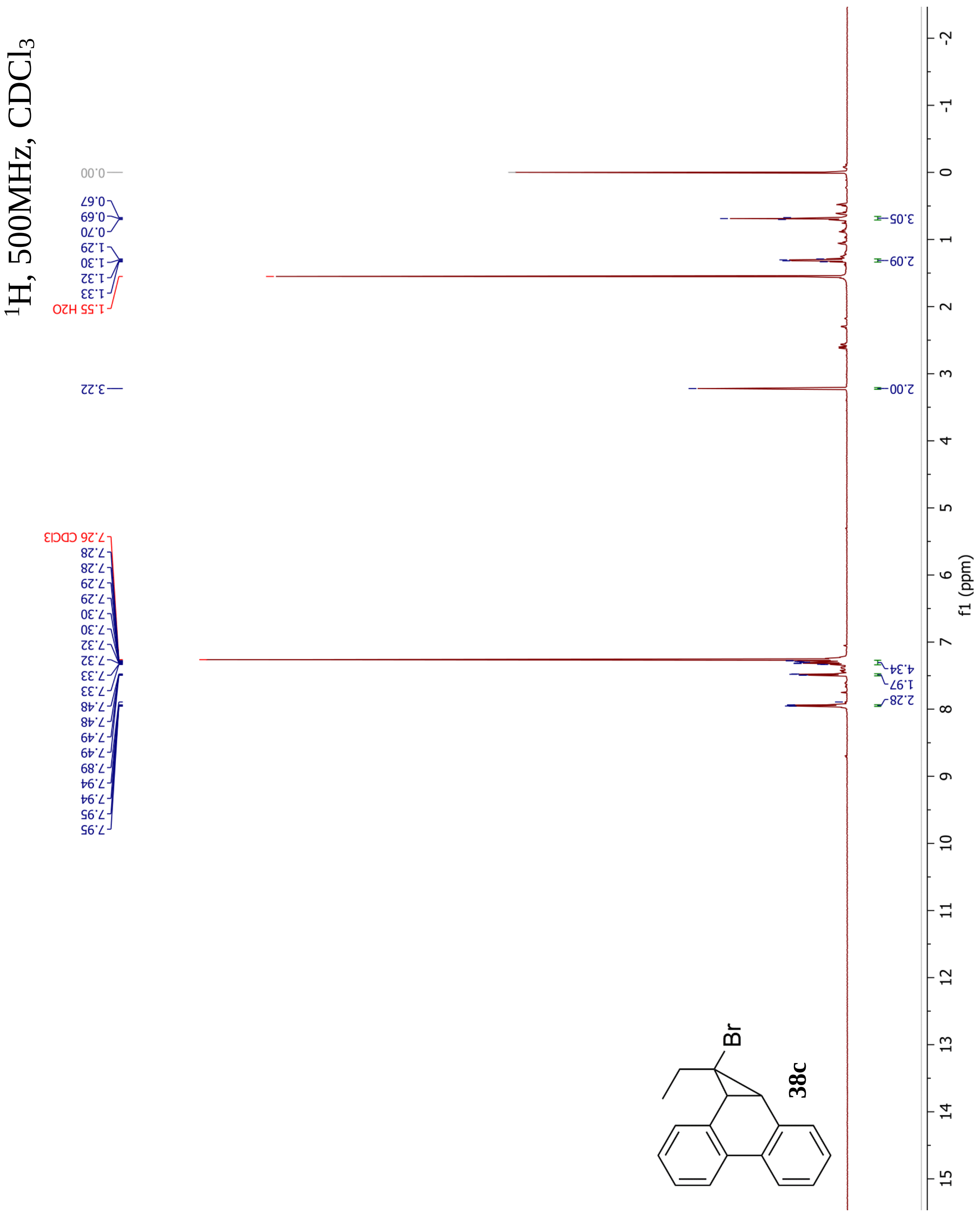
1H, 500MHz, CDCl3
0.00
0.67
0.69
0.70
1.29
1.30
1.32
1.33
1.55 H2O
3.22
7.26 CDCl3
7.28
7.28
7.29
7.29
7.30
7.30
7.32
7.32
7.33
7.33
7.48
7.48
7.49
7.49
7.89
7.94
7.94
7.95
7.95
3.05
2.09
2.00
4.34
1.97
2.28
f1 (ppm)
Br
38c

$^{13}C\{^1H\}$, 126 MHz, $CDCl_3$

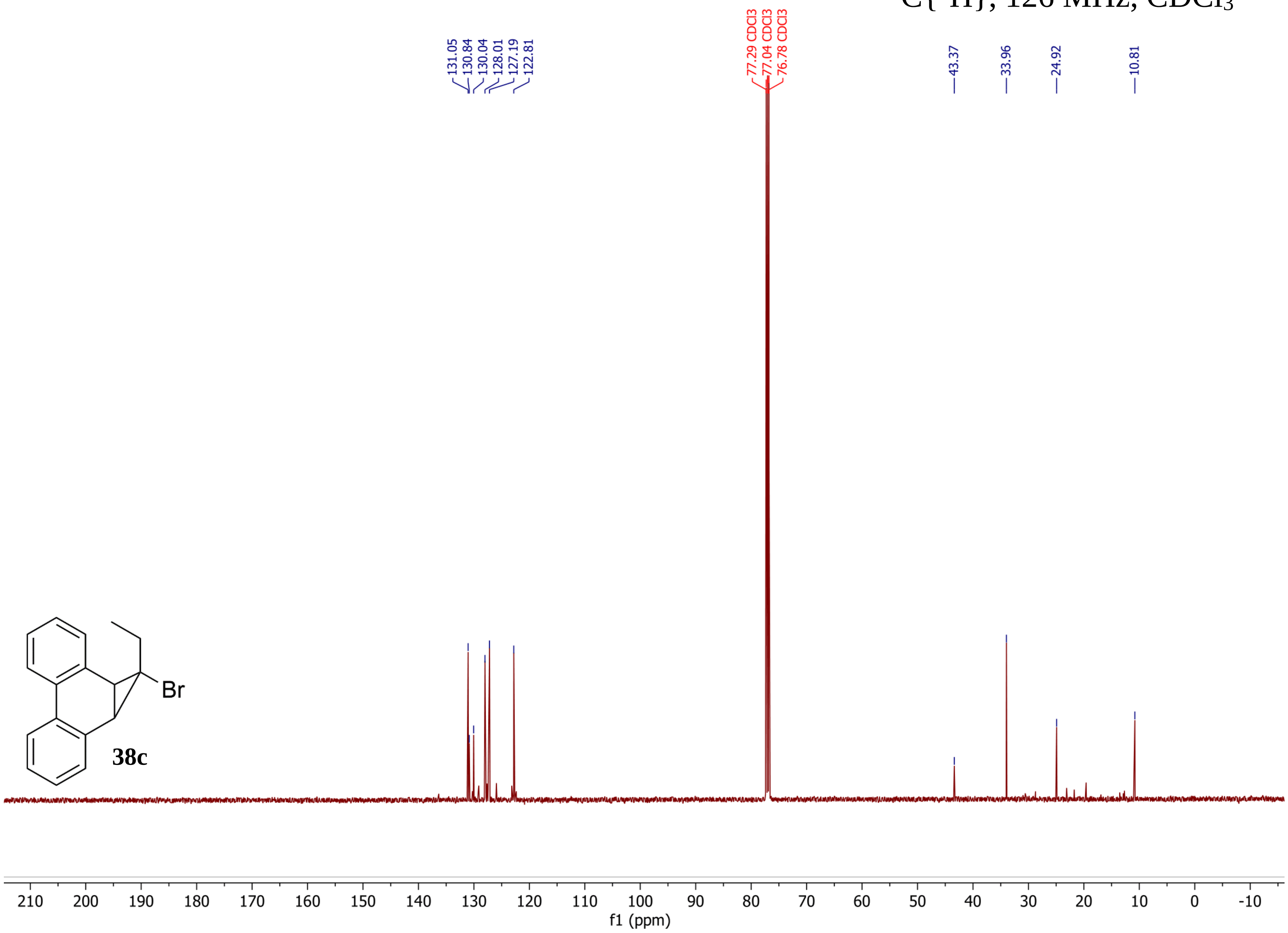

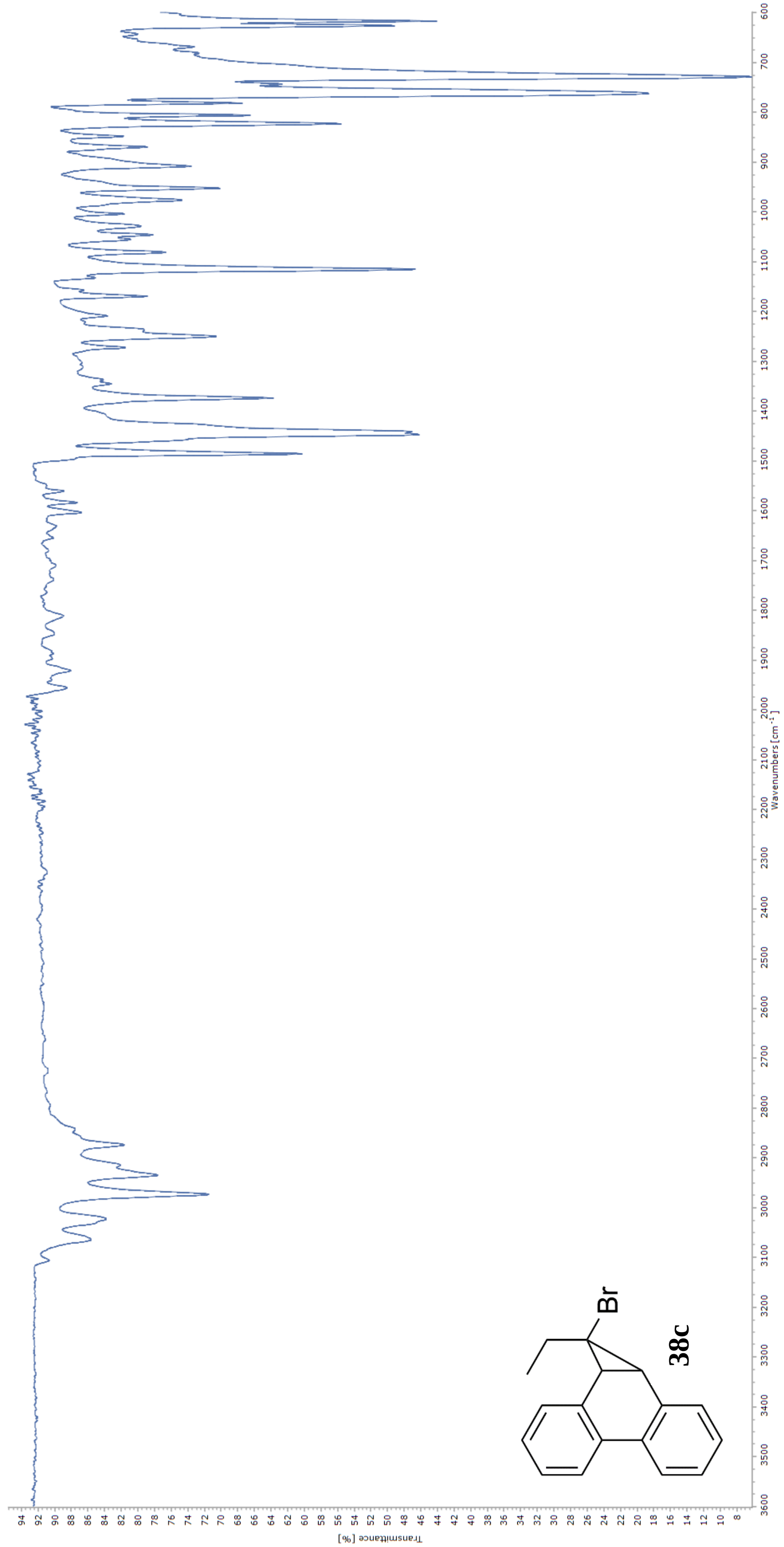
Br
38c
Wavenumbers [cm-1]
Transmittance [%]

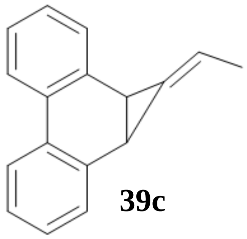

39c

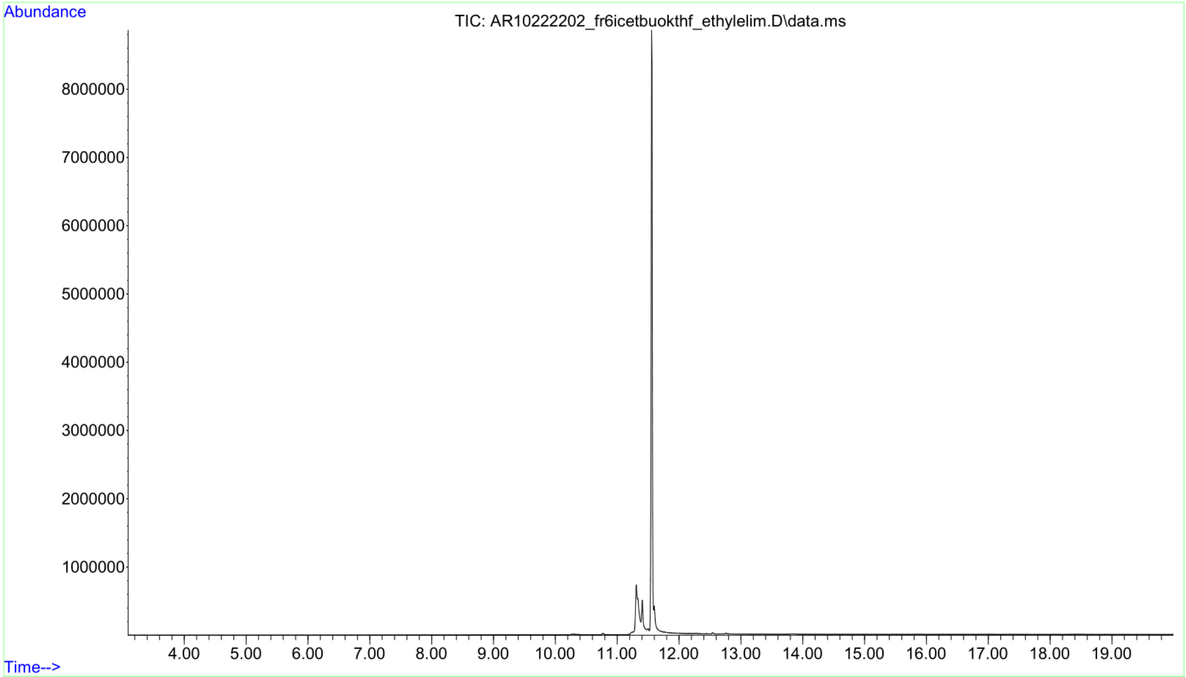

Abundance
TIC: AR10222202_fr6icetbuokthf_ethylelim.D\data.ms
Time-->

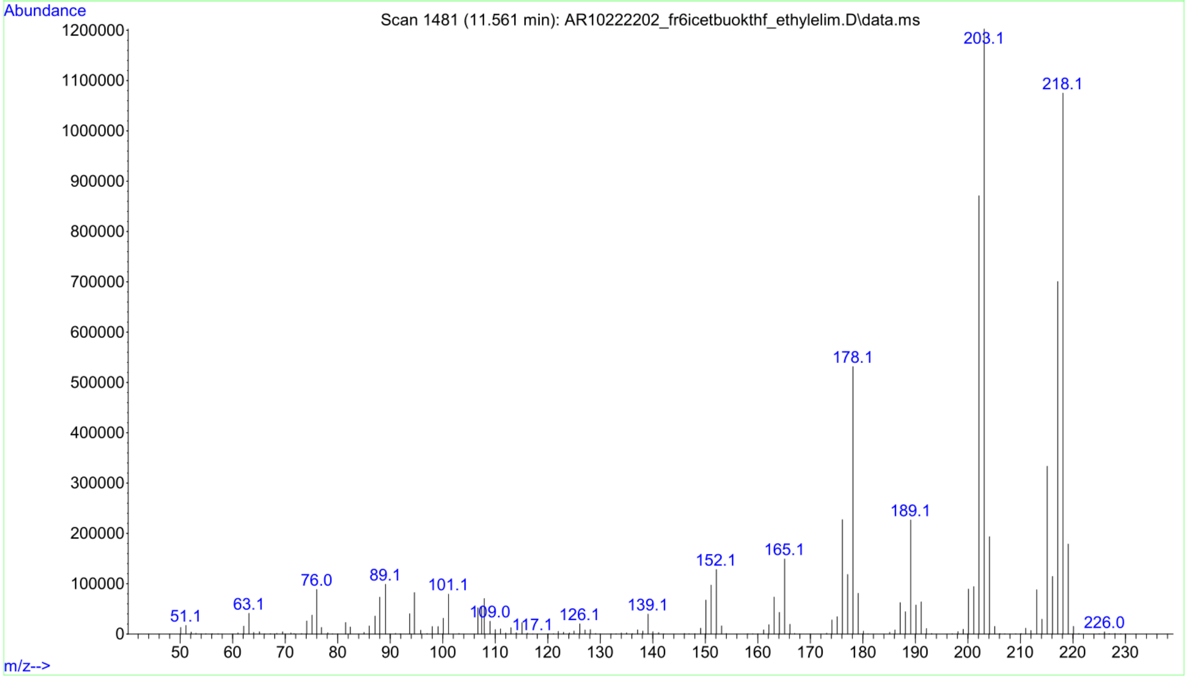

Abundance
Scan 1481 (11.561 min): AR10222202_fr6icetbuokthf_ethylelim.D\data.ms
203.1
218.1
178.1
189.1
165.1
152.1
139.1
126.1
117.1
109.0
101.1
89.1
76.0
63.1
51.1
226.0
m/z-->

^{1}H, 500MHz, $CDCl_3$

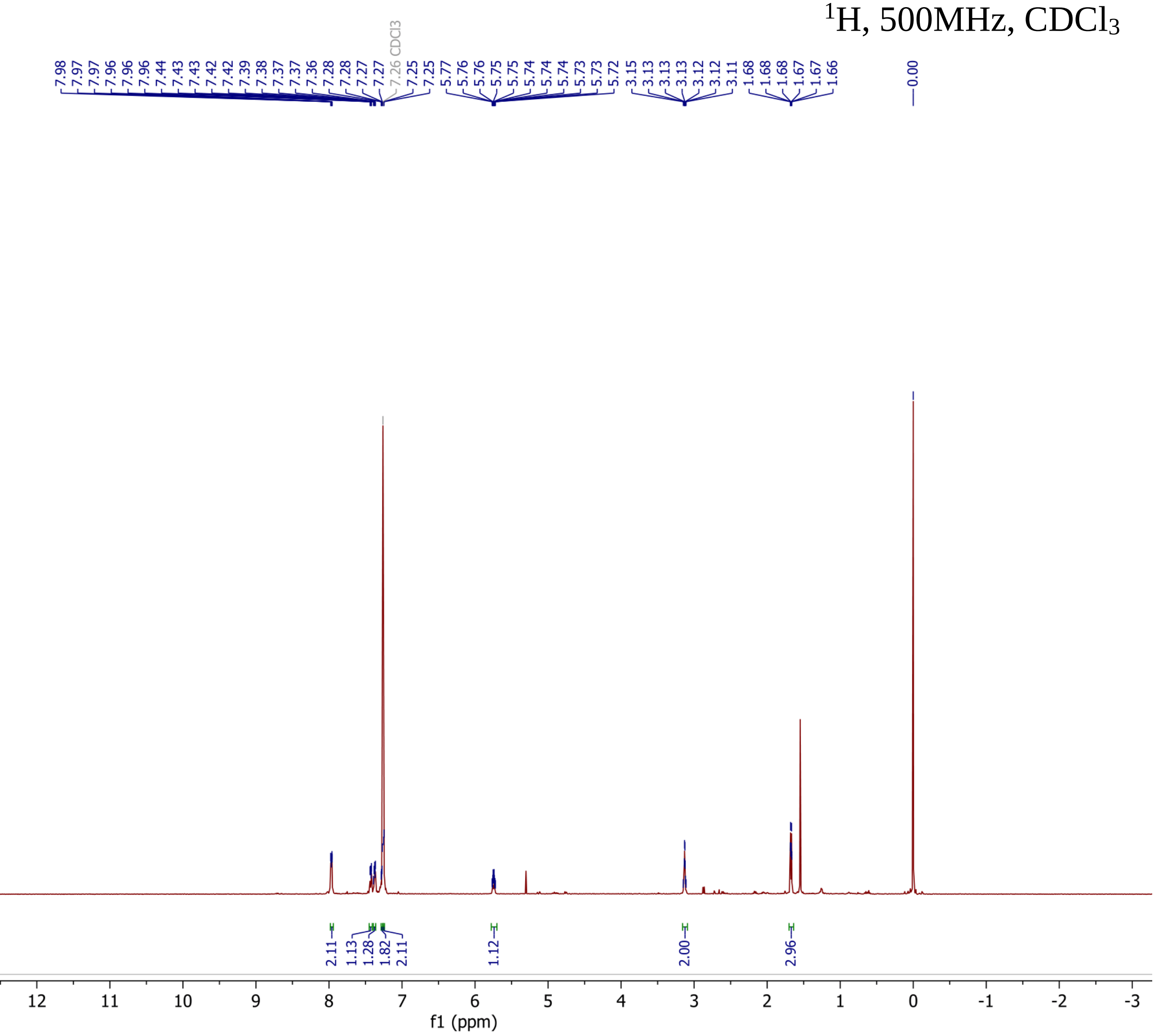

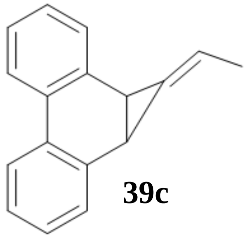

39c

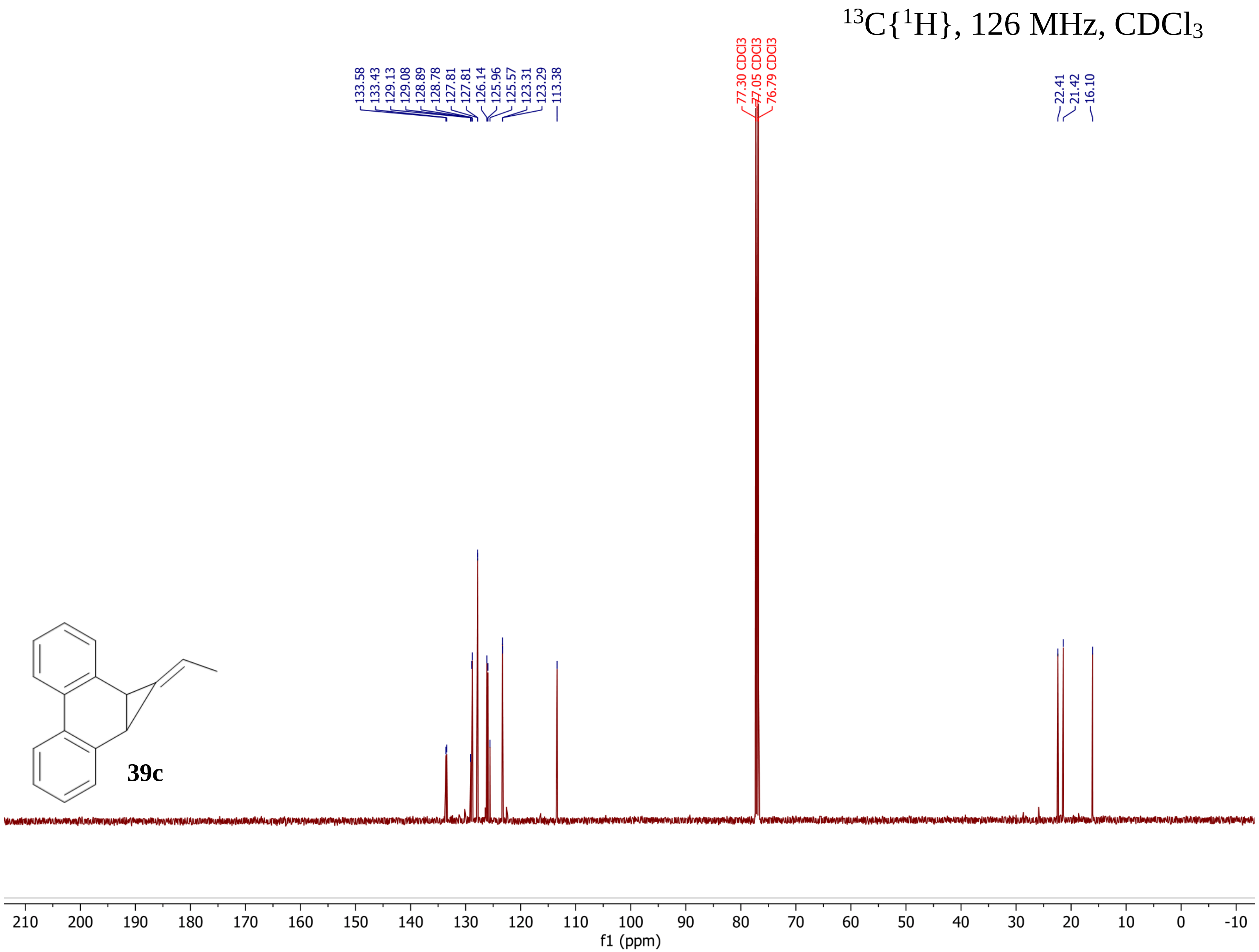
13C{1H}, 126 MHz, CDCl3
133.58
133.43
129.13
129.08
128.89
128.78
127.81
127.81
126.14
125.96
125.57
123.31
123.29
113.38
77.30 CDCl3
77.05 CDCl3
76.79 CDCl3
22.41
21.42
16.10
39c
210 200 190 180 170 160 150 140 130 120 110 100 90 80 70 60 50 40 30 20 10 0 -10
f1 (ppm)

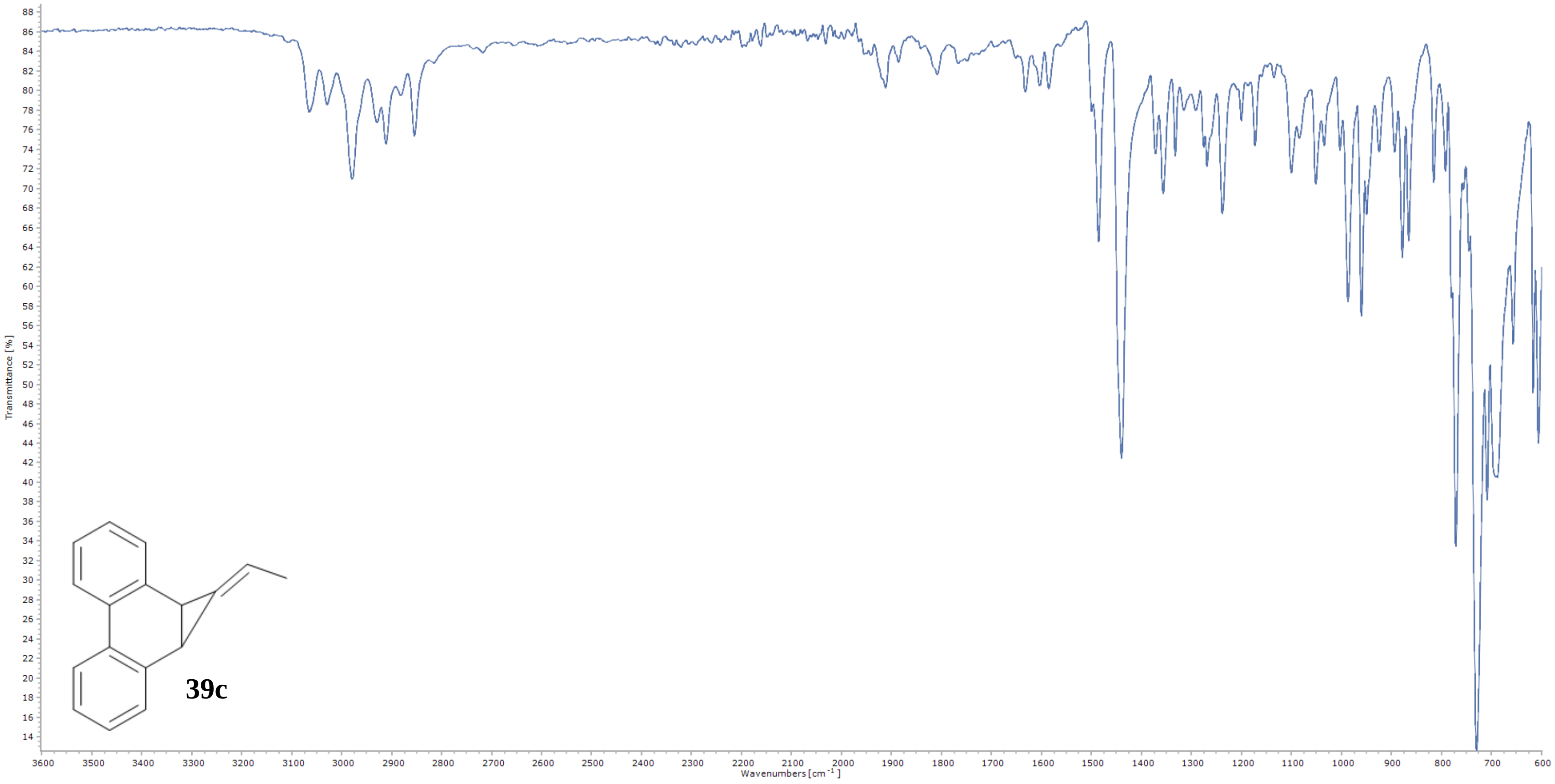
Transmittance [%]
Wavenumbers [cm⁻¹]
3600 3500 3400 3300 3200 3100 3000 2900 2800 2700 2600 2500 2400 2300 2200 2100 2000 1900 1800 1700 1600 1500 1400 1300 1200 1100 1000 900 800 700 600
88 86 84 82 80 78 76 74 72 70 68 66 64 62 60 58 56 54 52 50 48 46 44 42 40 38 36 34 32 30 28 26 24 22 20 18 16 14

39c

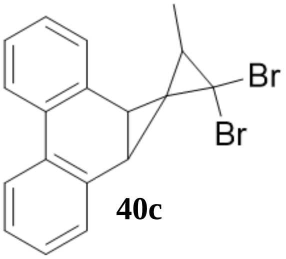
Br
Br

40c

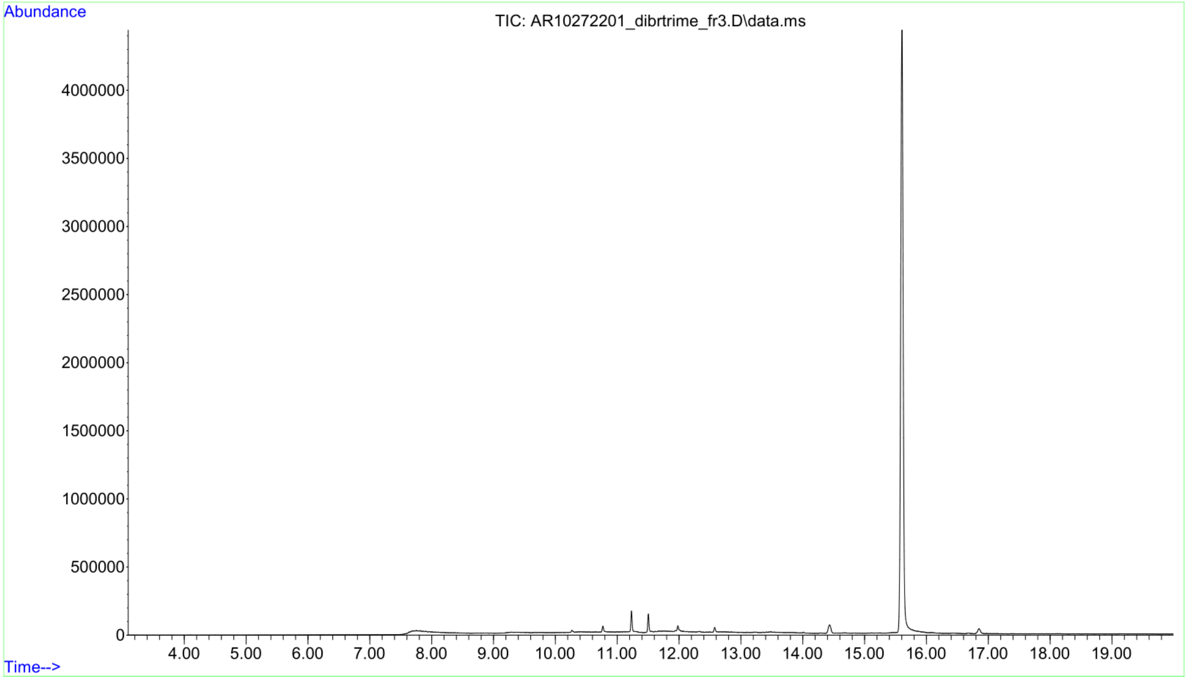
Abundance
TIC: AR10272201_dibrtrime_fr3.D\data.ms
4000000
3500000
3000000
2500000
2000000
1500000
1000000
500000
0
4.00
5.00
6.00
7.00
8.00
9.00
10.00
11.00
12.00
13.00
14.00
15.00
16.00
17.00
18.00
19.00
Time-->

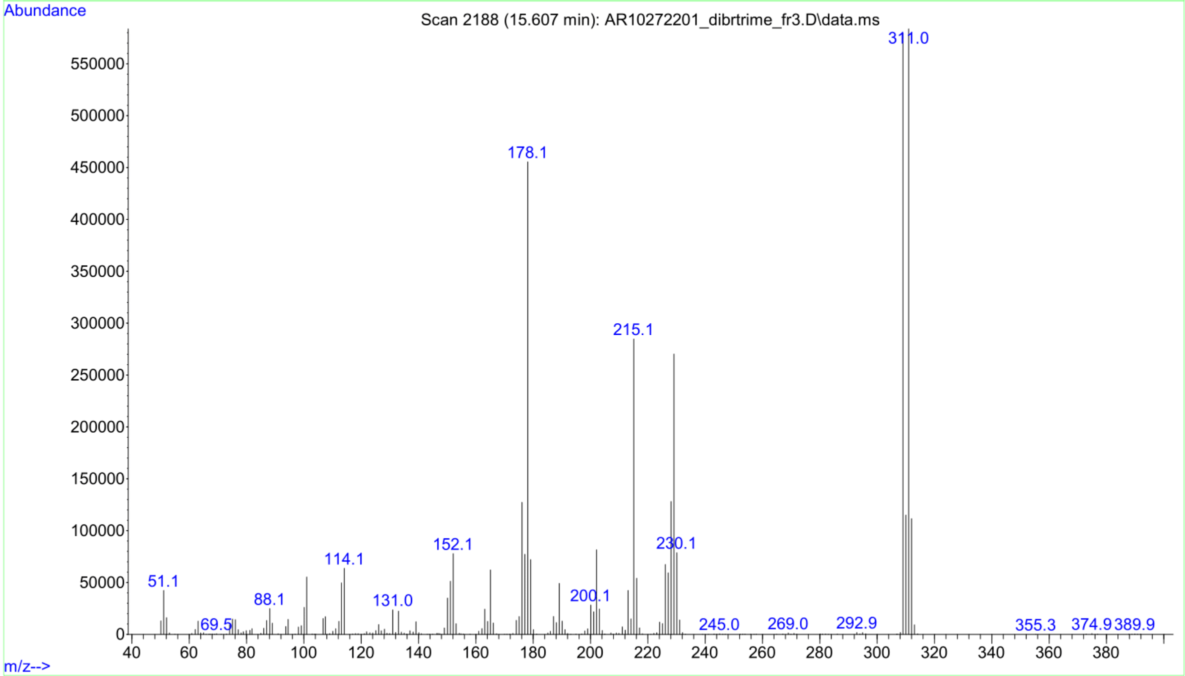
Abundance
Scan 2188 (15.607 min): AR10272201_dibrtrime_fr3.D\data.ms
311.0
178.1
215.1
230.1
152.1
114.1
51.1
88.1
131.0
200.1
69.5
245.0
269.0
292.9
355.3
374.9
389.9
550000
500000
450000
400000
350000
300000
250000
200000
150000
100000
50000
0
40
60
80
100
120
140
160
180
200
220
240
260
280
300
320
340
360
380
m/z-->

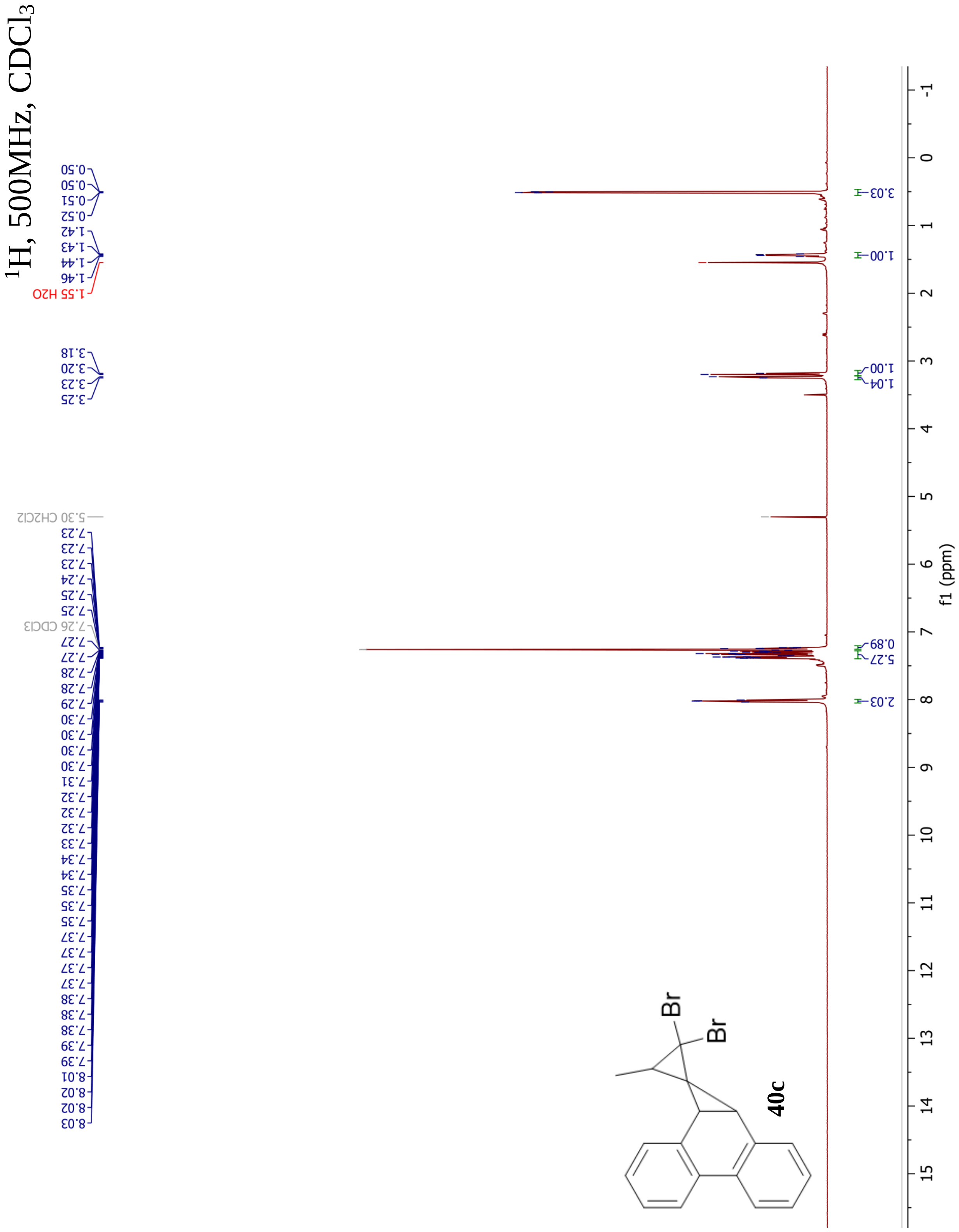

1H, 500MHz, CDCl3
0.50
0.50
0.51
0.52
1.42
1.43
1.44
1.46
1.55 H2O
3.18
3.20
3.23
3.25
5.30 CH2Cl2
7.23
7.23
7.23
7.24
7.25
7.25
7.26 CDCl3
7.27
7.27
7.28
7.28
7.29
7.30
7.30
7.30
7.30
7.31
7.32
7.32
7.32
7.33
7.34
7.34
7.35
7.35
7.35
7.37
7.37
7.37
7.37
7.38
7.38
7.38
7.39
7.39
8.01
8.02
8.02
8.03
3.03
1.00
1.00
1.04
0.89
5.27
2.03
f1 (ppm)
Br
Br
40c

$^{13}C\{^{1}H\}$, 126 MHz, $CDCl_3$

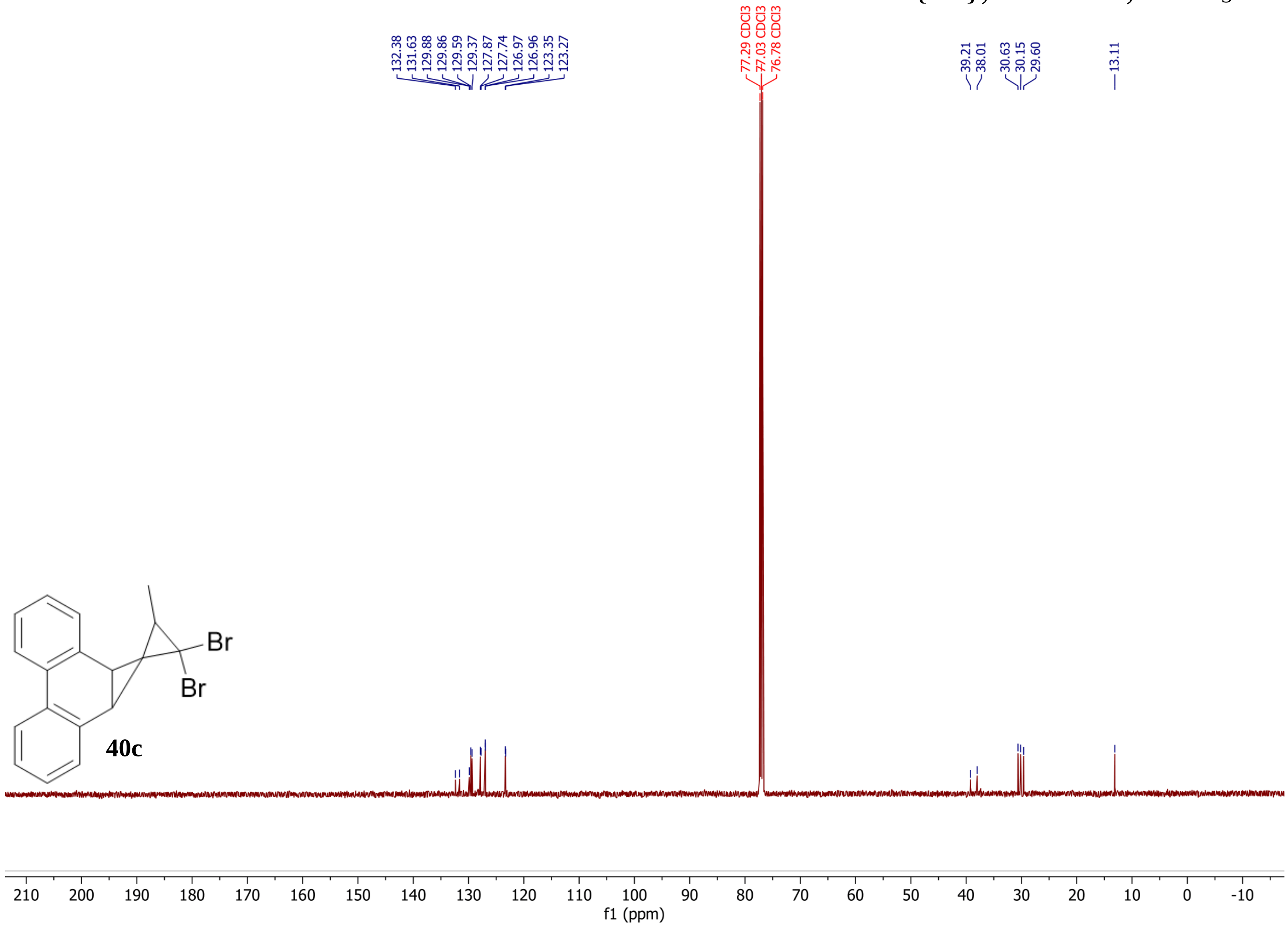

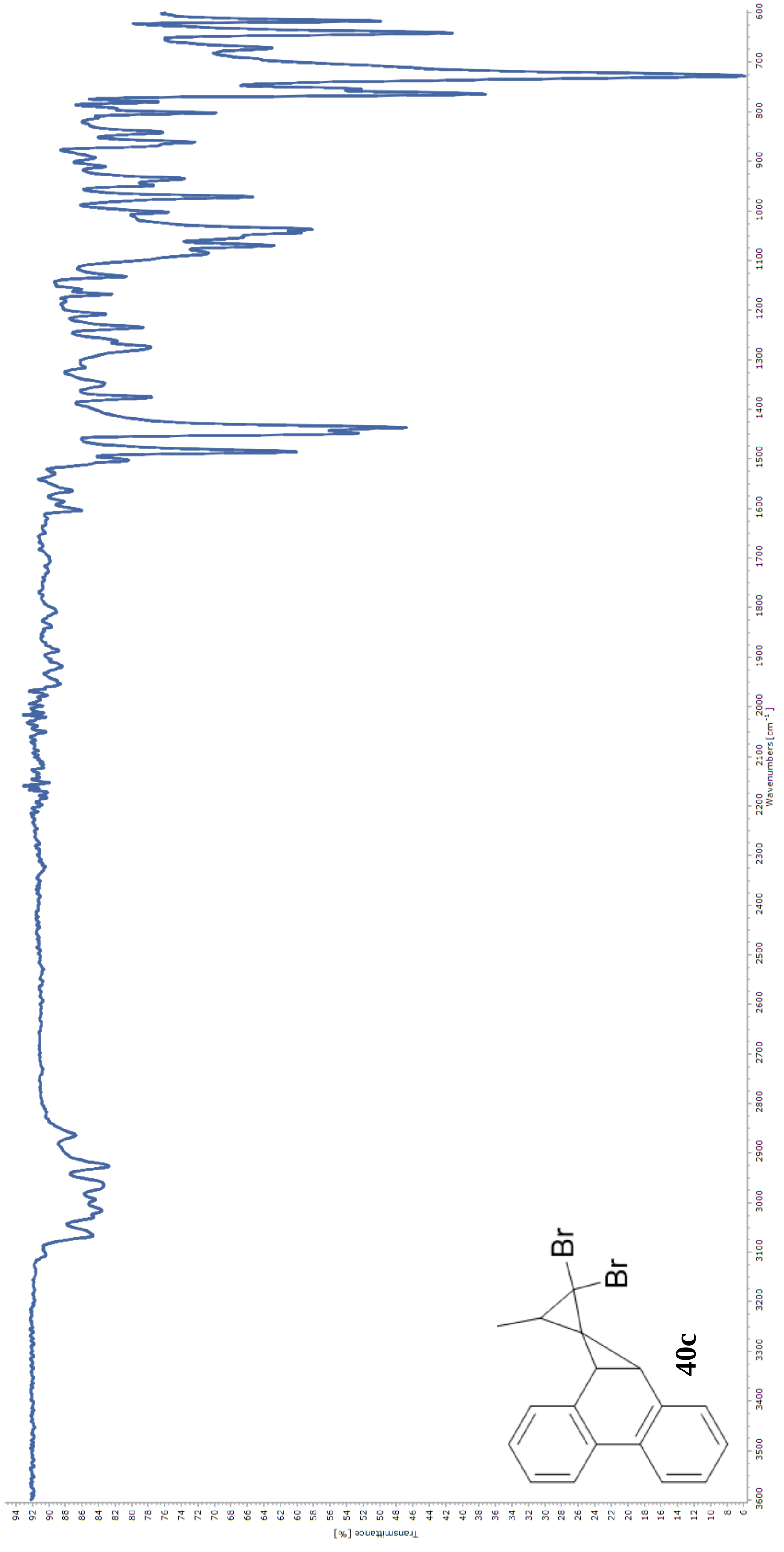
Transmittance [%]
Wavenumbers [cm-1]
Br
Br
40c

^{1}H, 500MHz, $CDCl_3$

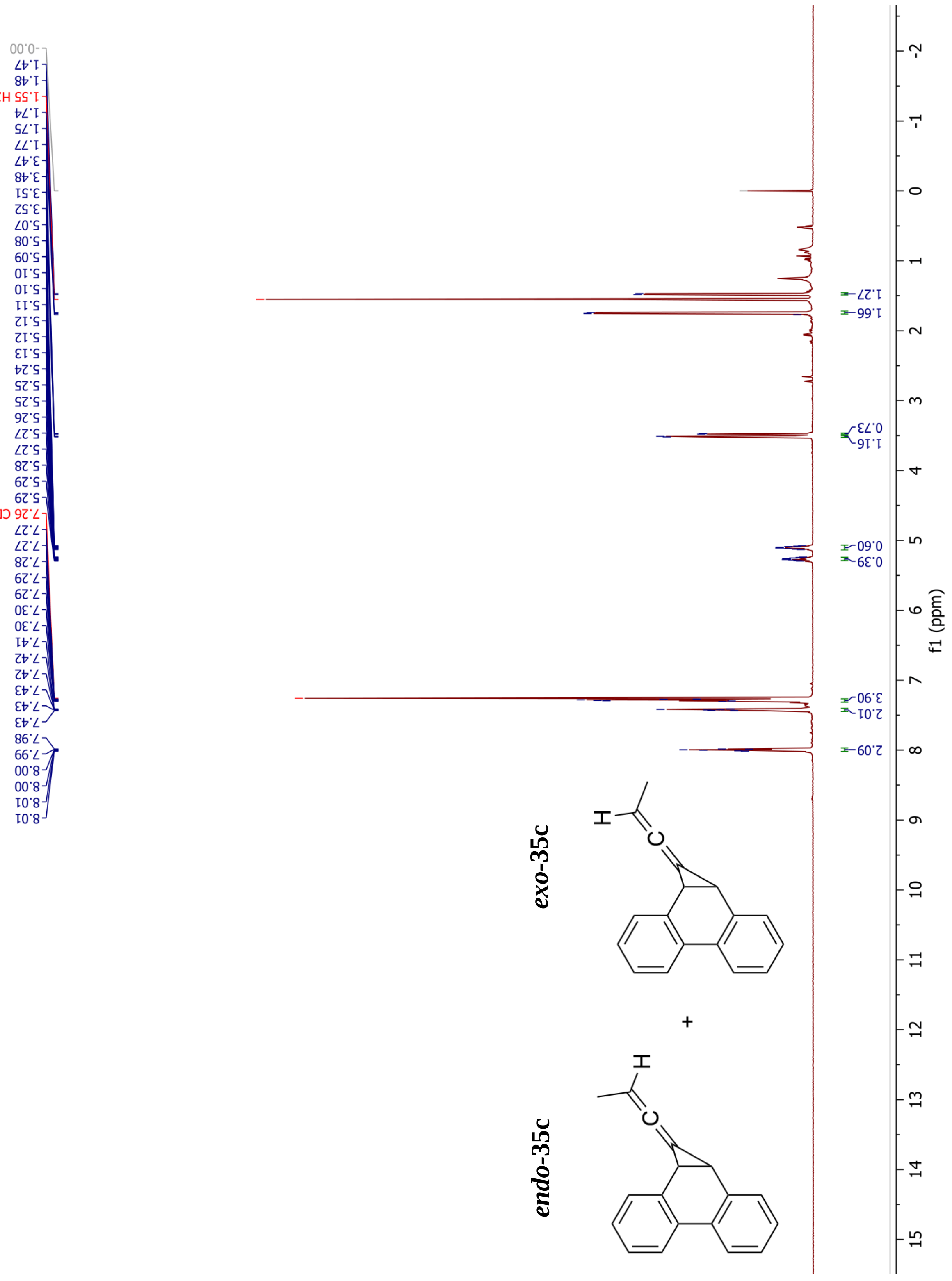

$^{13}C\{^{1}H\}$, 126 MHz, $CDCl_3$

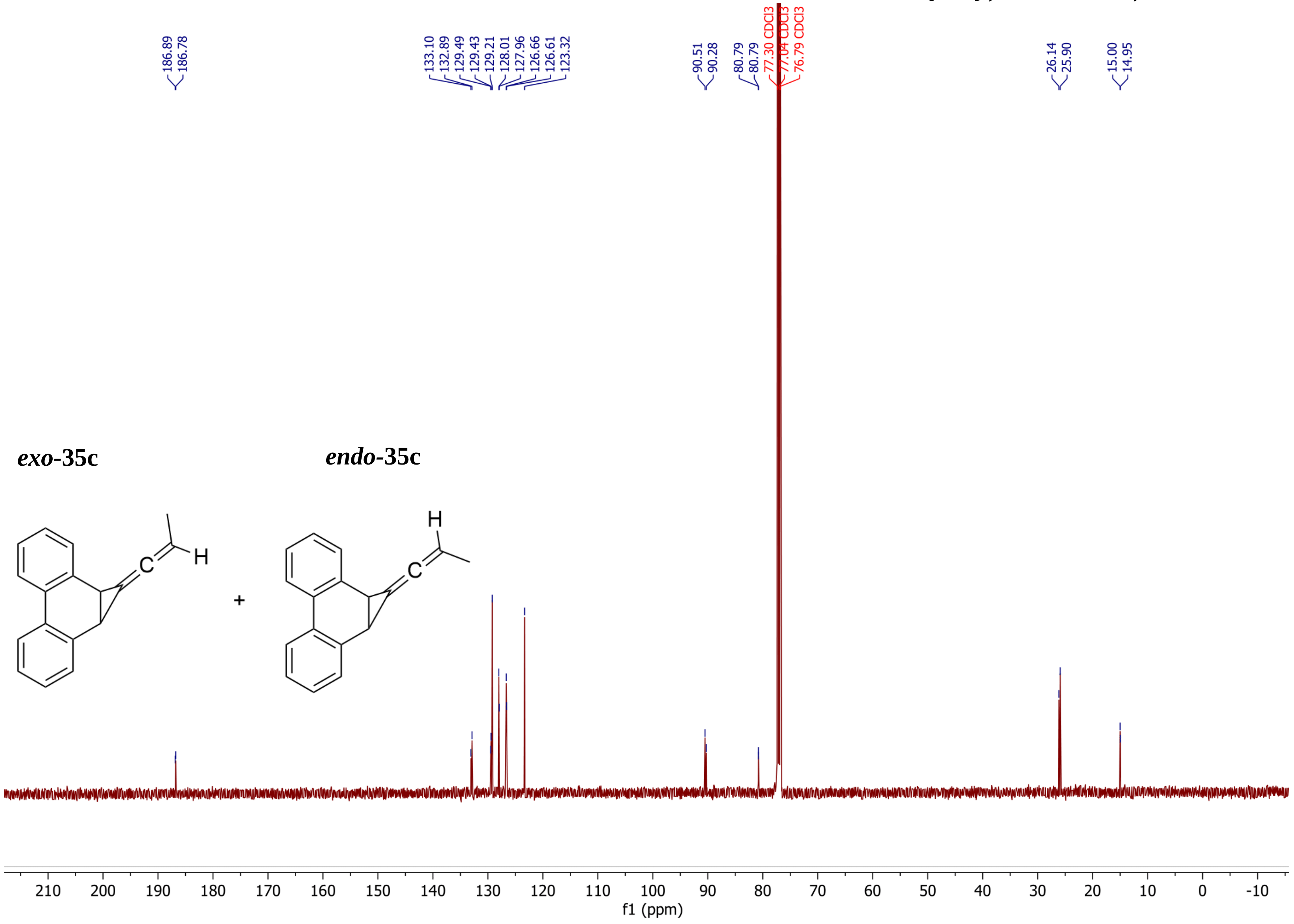

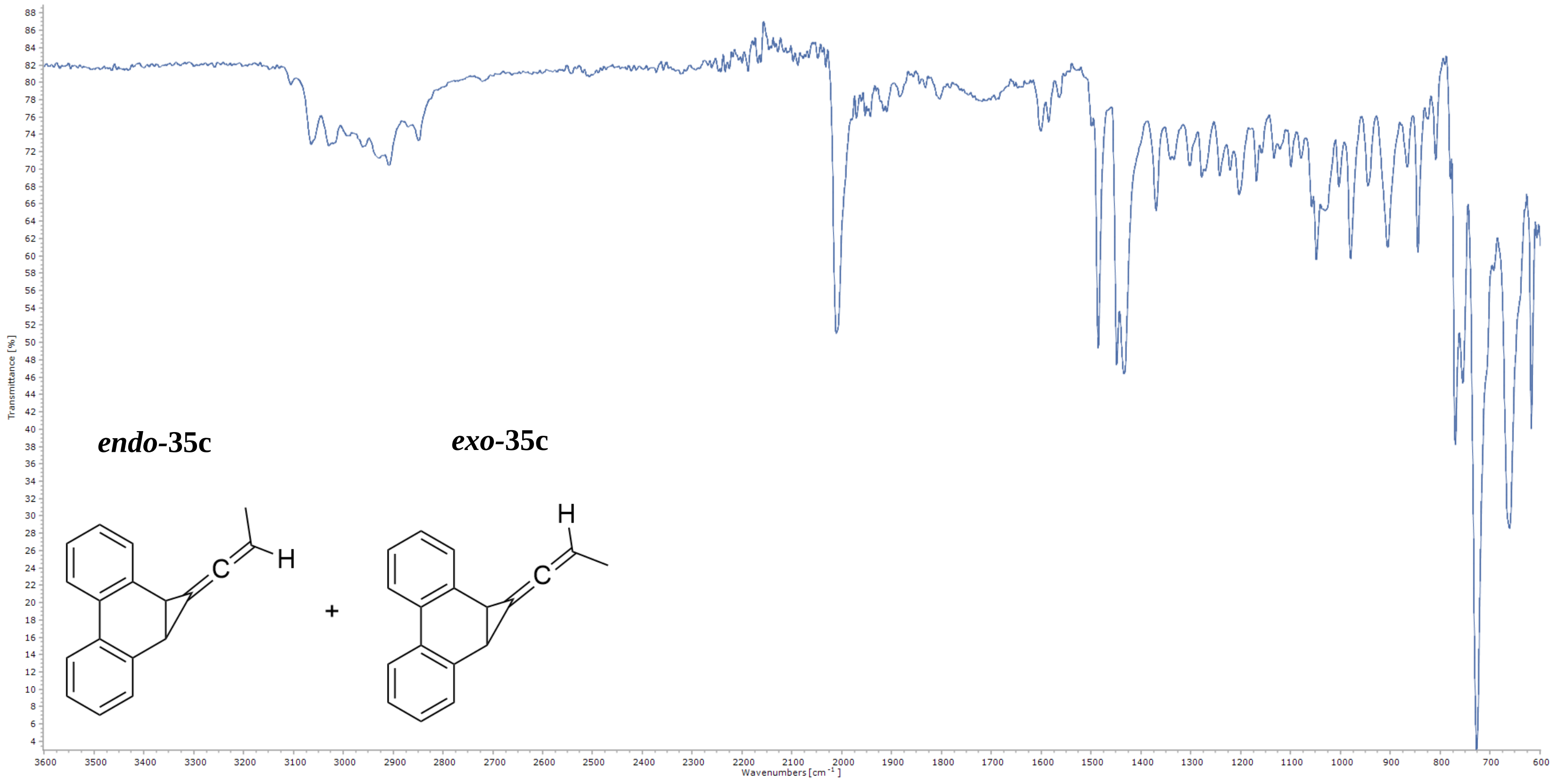
Transmittance [%]
Wavenumbers [cm -1]
endo-35c
exo-35c
C
H
+

^{1}H, 500MHz, $CDCl_3$

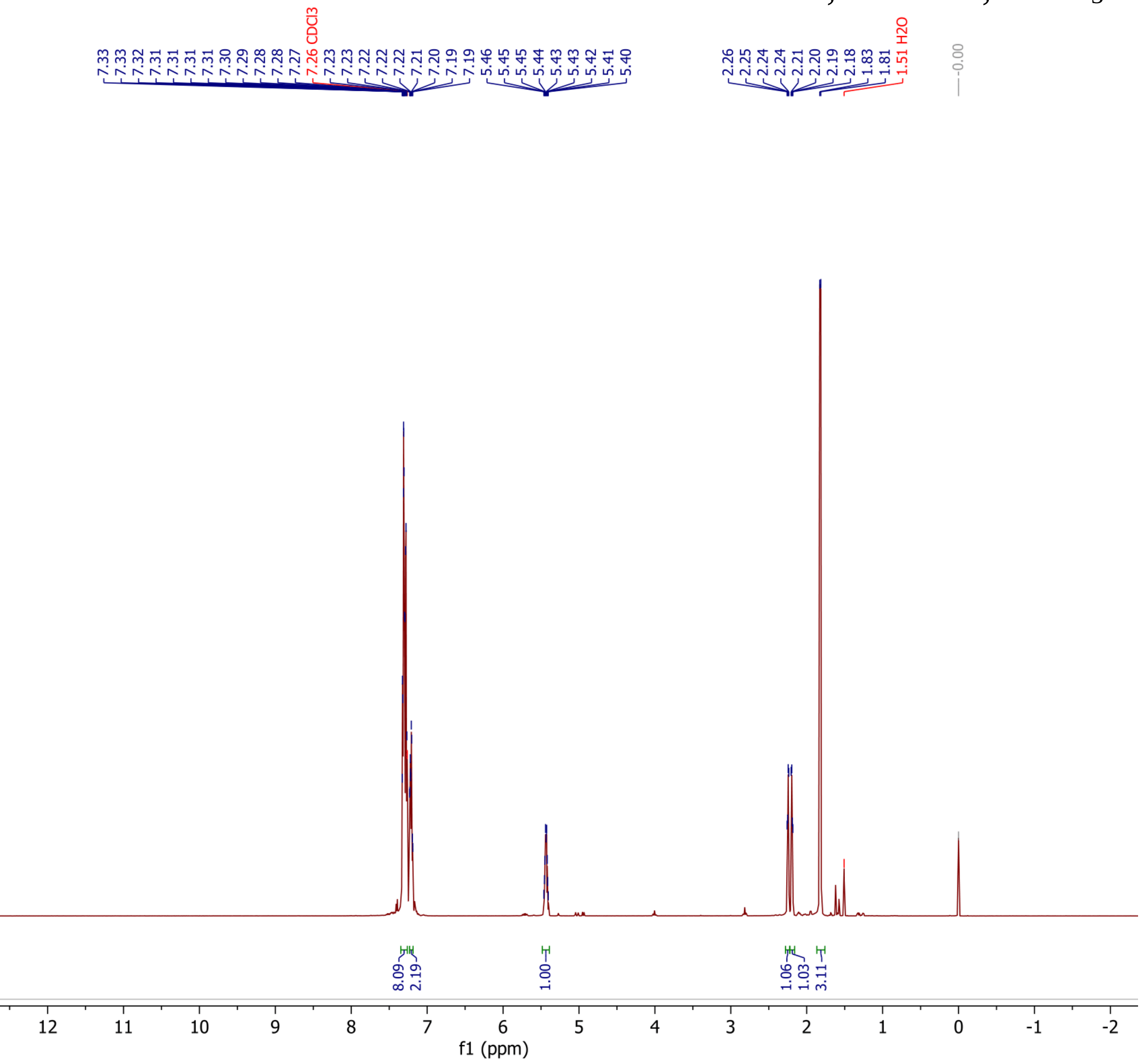

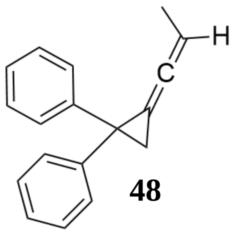

48

$^{13}C\{^1H\}$, 126 MHz, $CDCl_3$

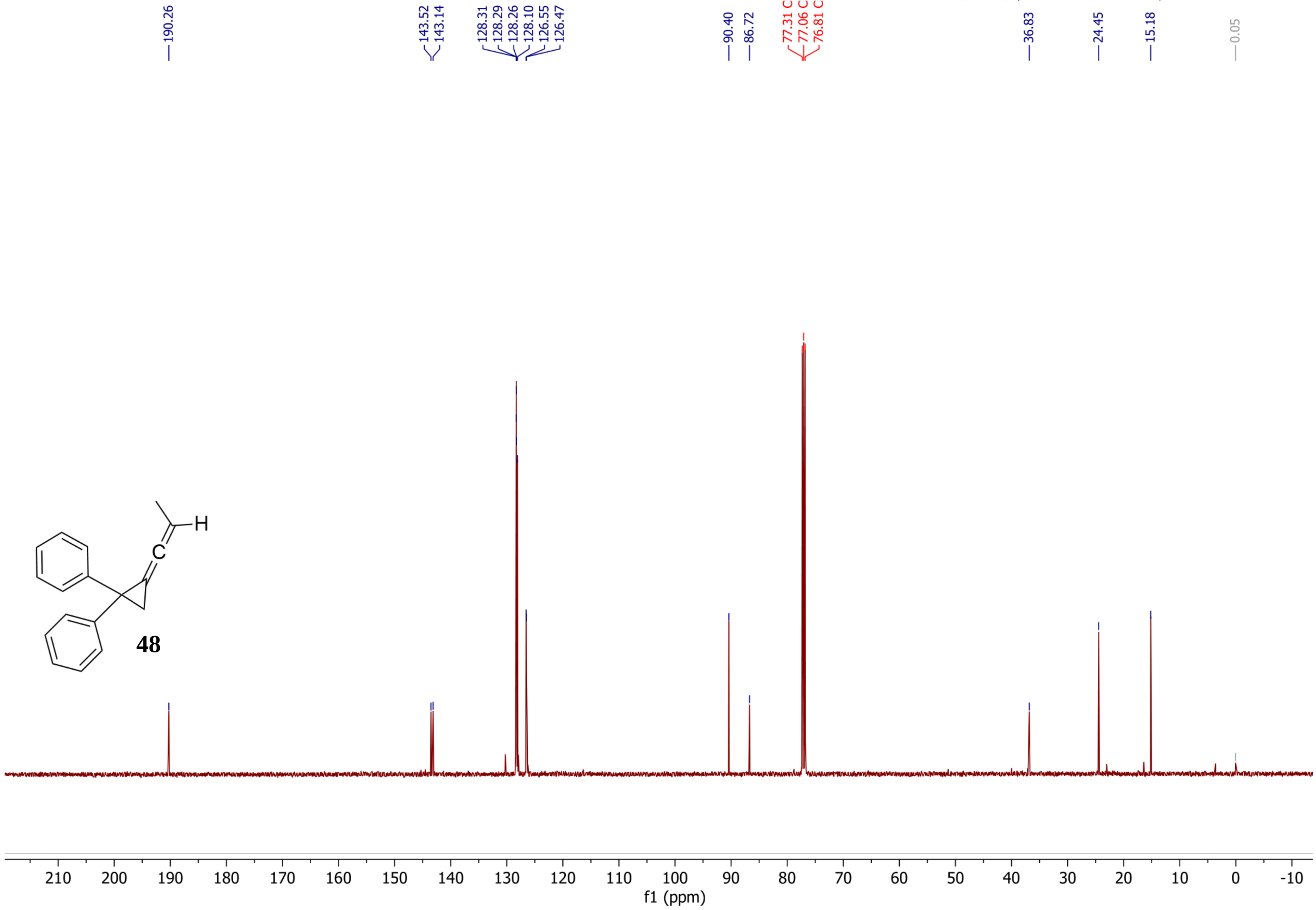

^1H, 500MHz, C_6D_6

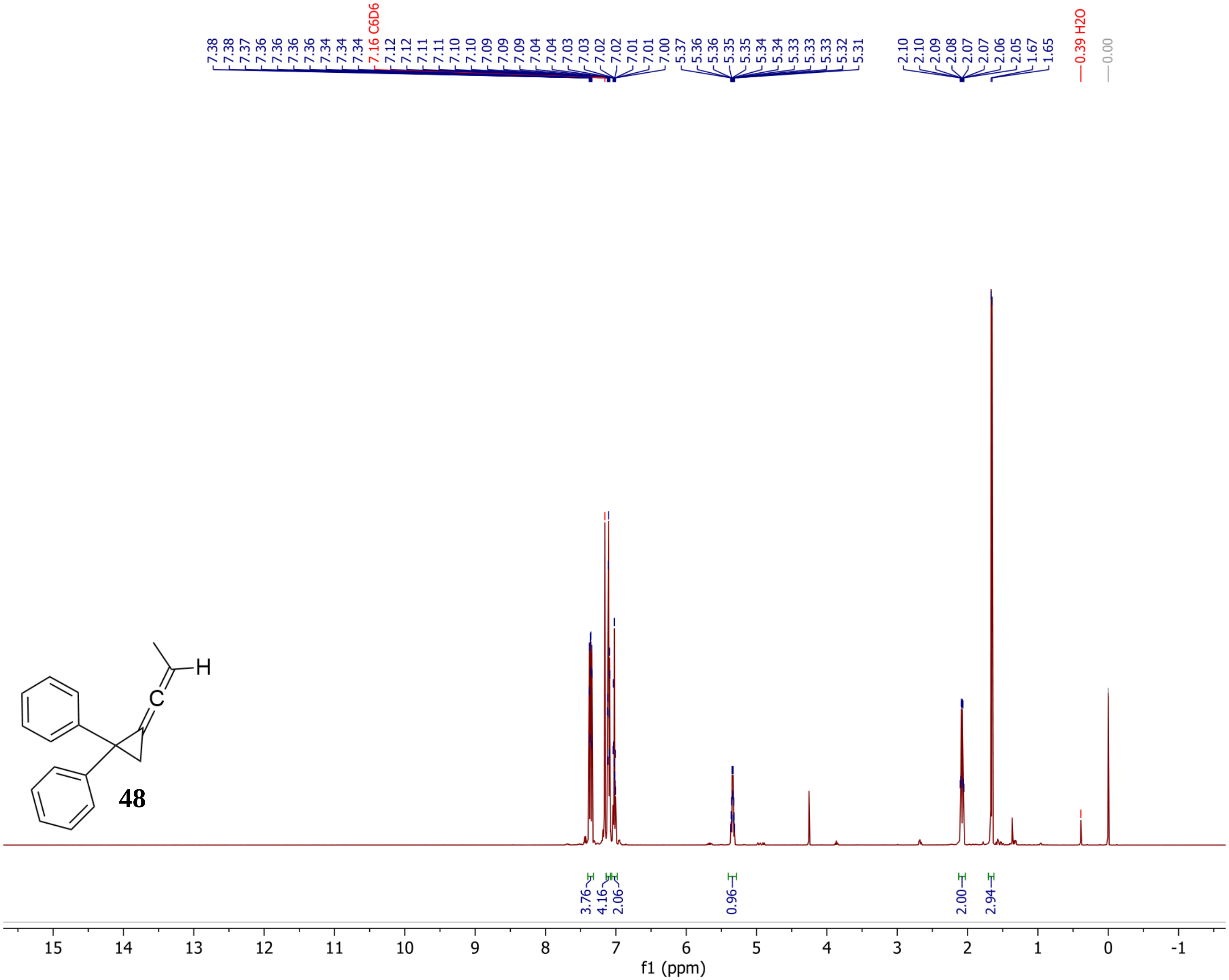

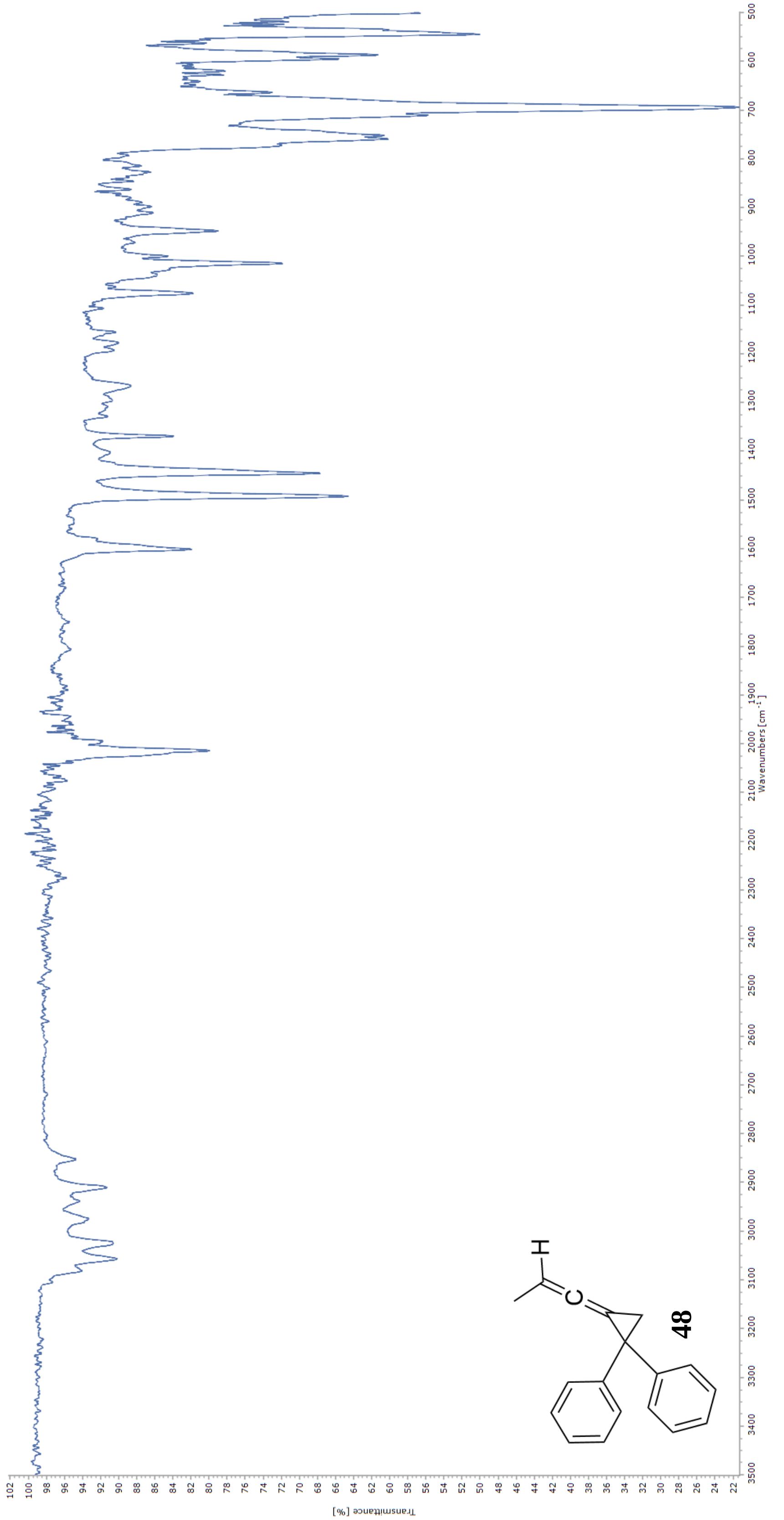
Transmittance [%]
Wavenumbers [cm⁻¹]
48

^{1}H, 500MHz, C_6D_6

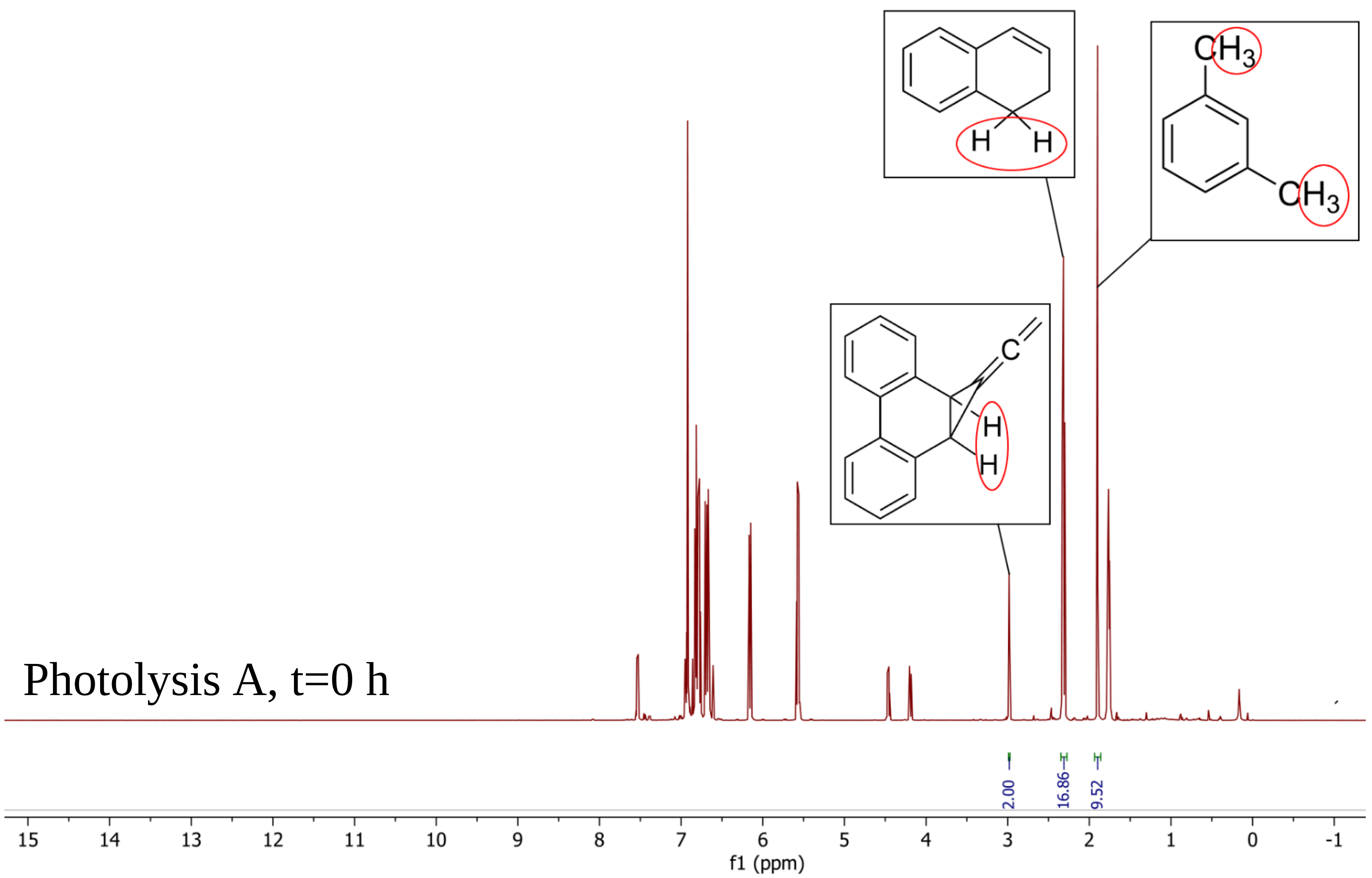

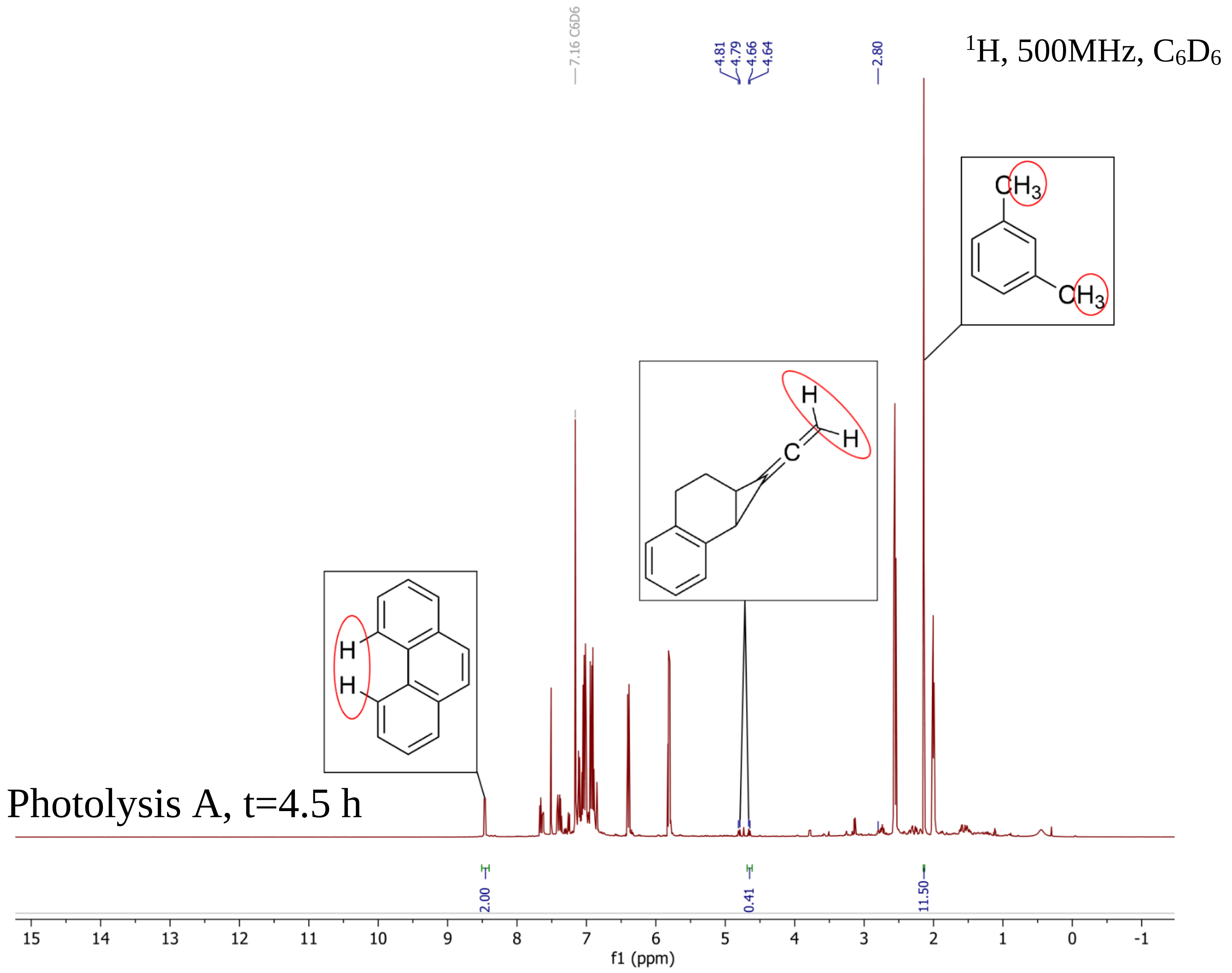

1H, 500MHz, C6D6
Photolysis A, t=4.5 h
7.16 C6D6
4.81
4.79
4.66
4.64
2.80
CH3
CH3
H
H
C
H
H
2.00
0.41
11.50
15
14
13
12
11
10
9
8
7
6
5
4
3
2
1
0
-1
f1 (ppm)

Photolysis A, t=4.5 h

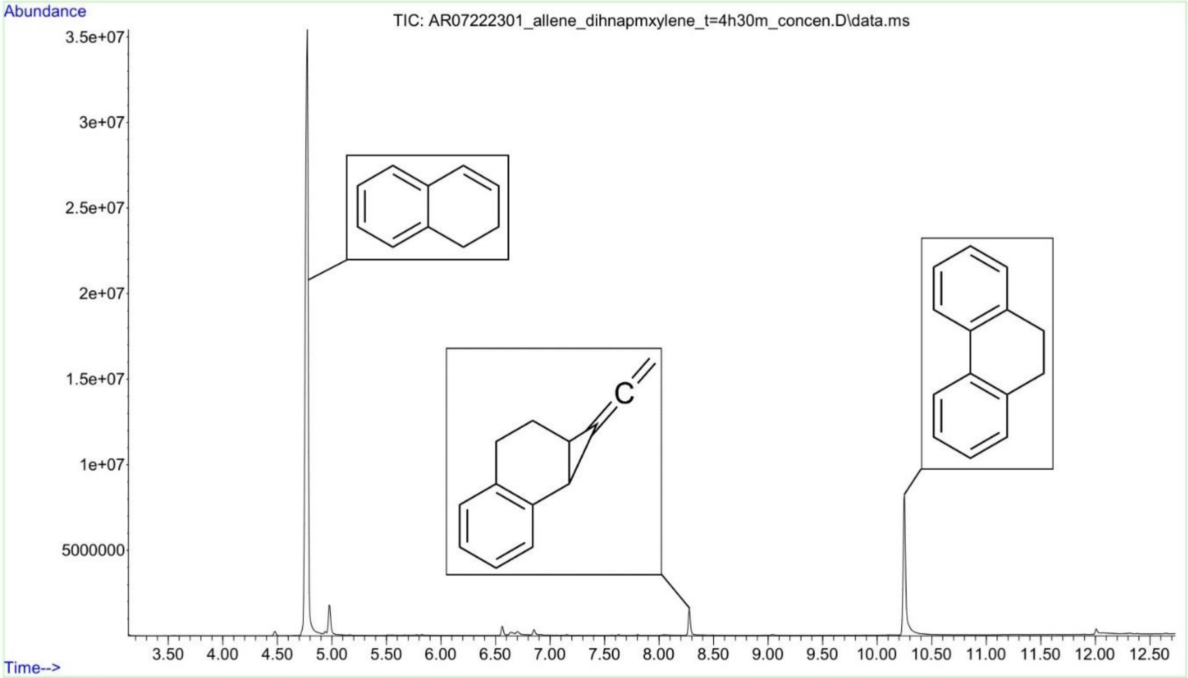

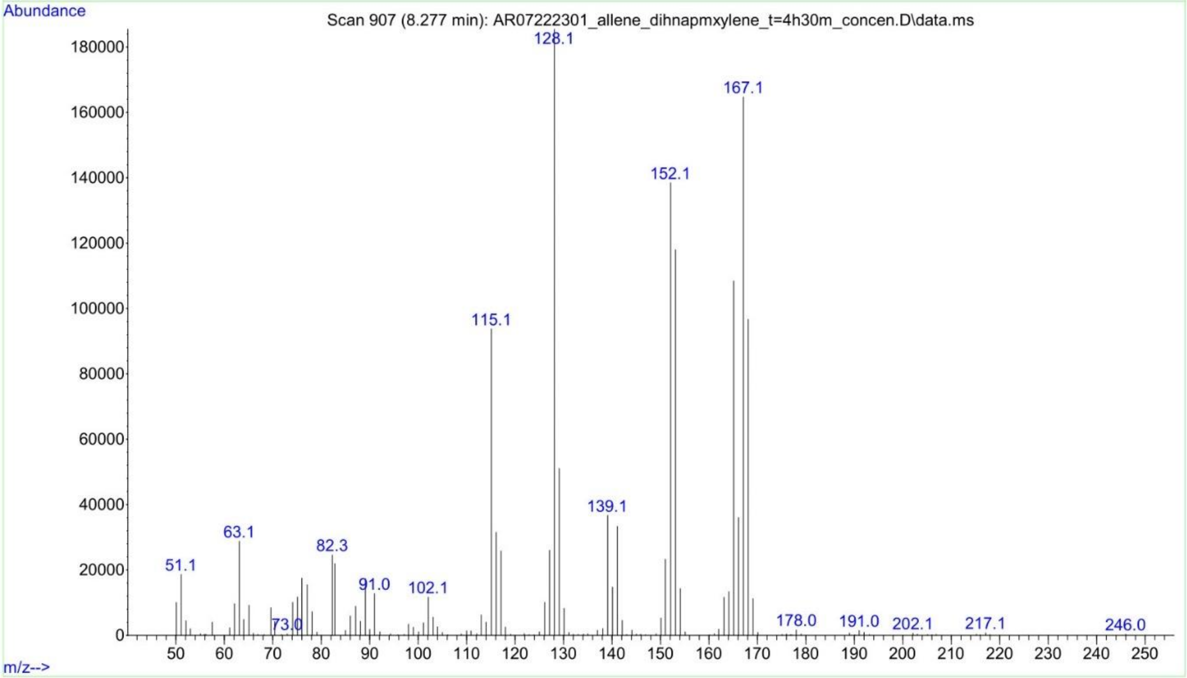

Photolysis B, t = 27 hrs

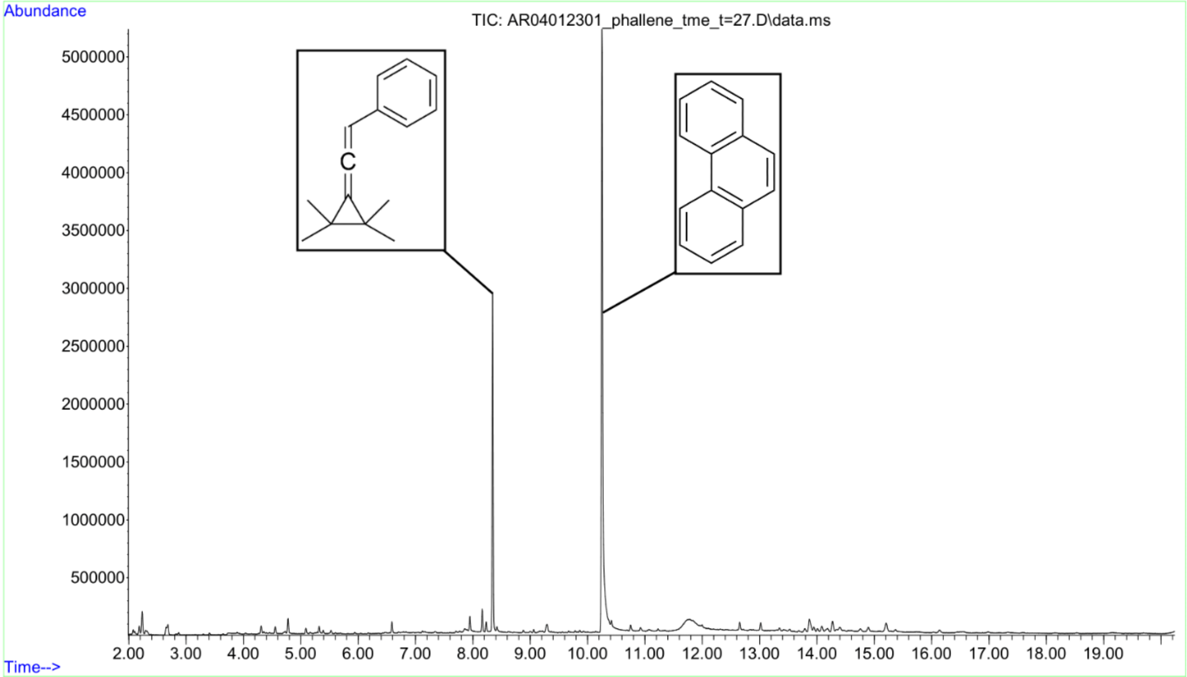

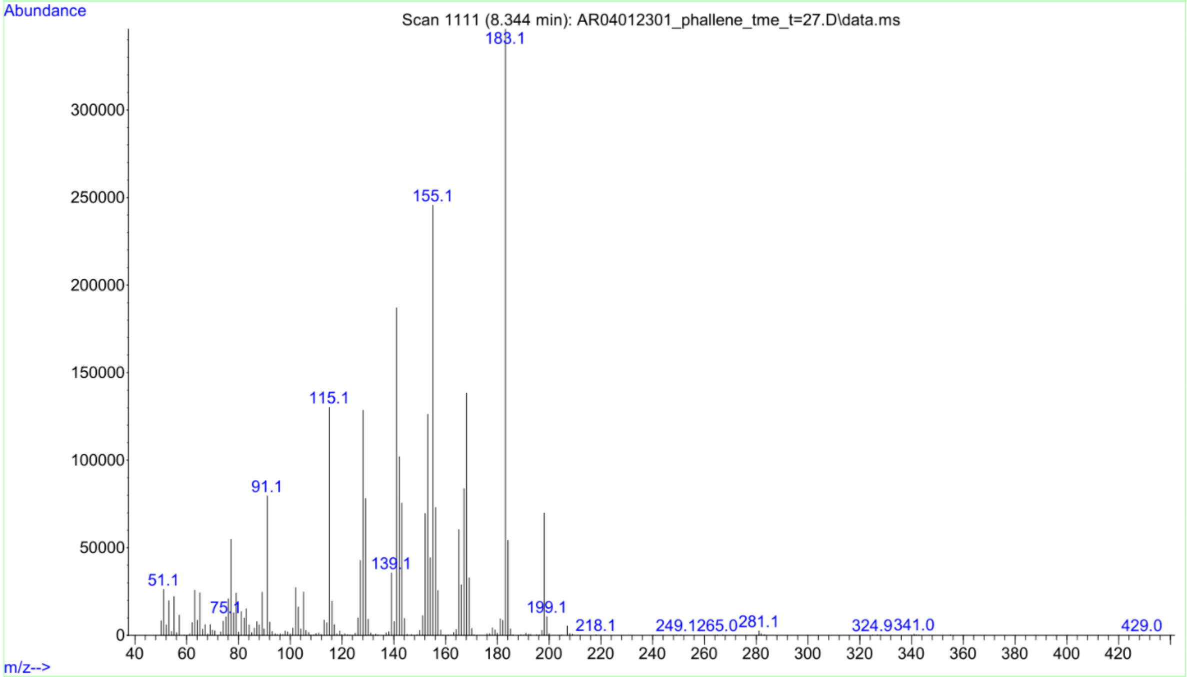

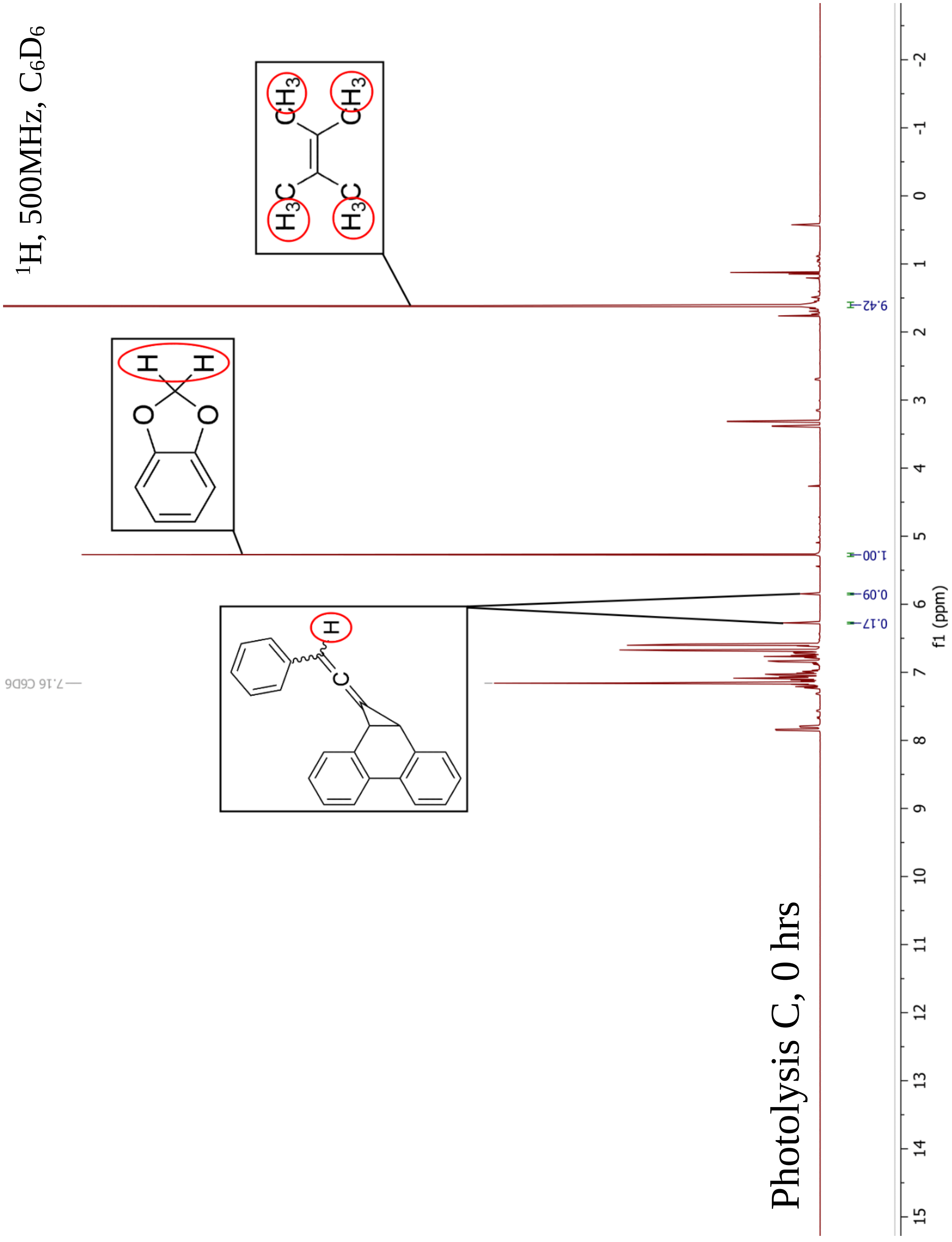

1H, 500MHz, C6D6
Photolysis C, 0 hrs
7.16 C6D6
9.42
1.00
0.09
0.17
f1 (ppm)

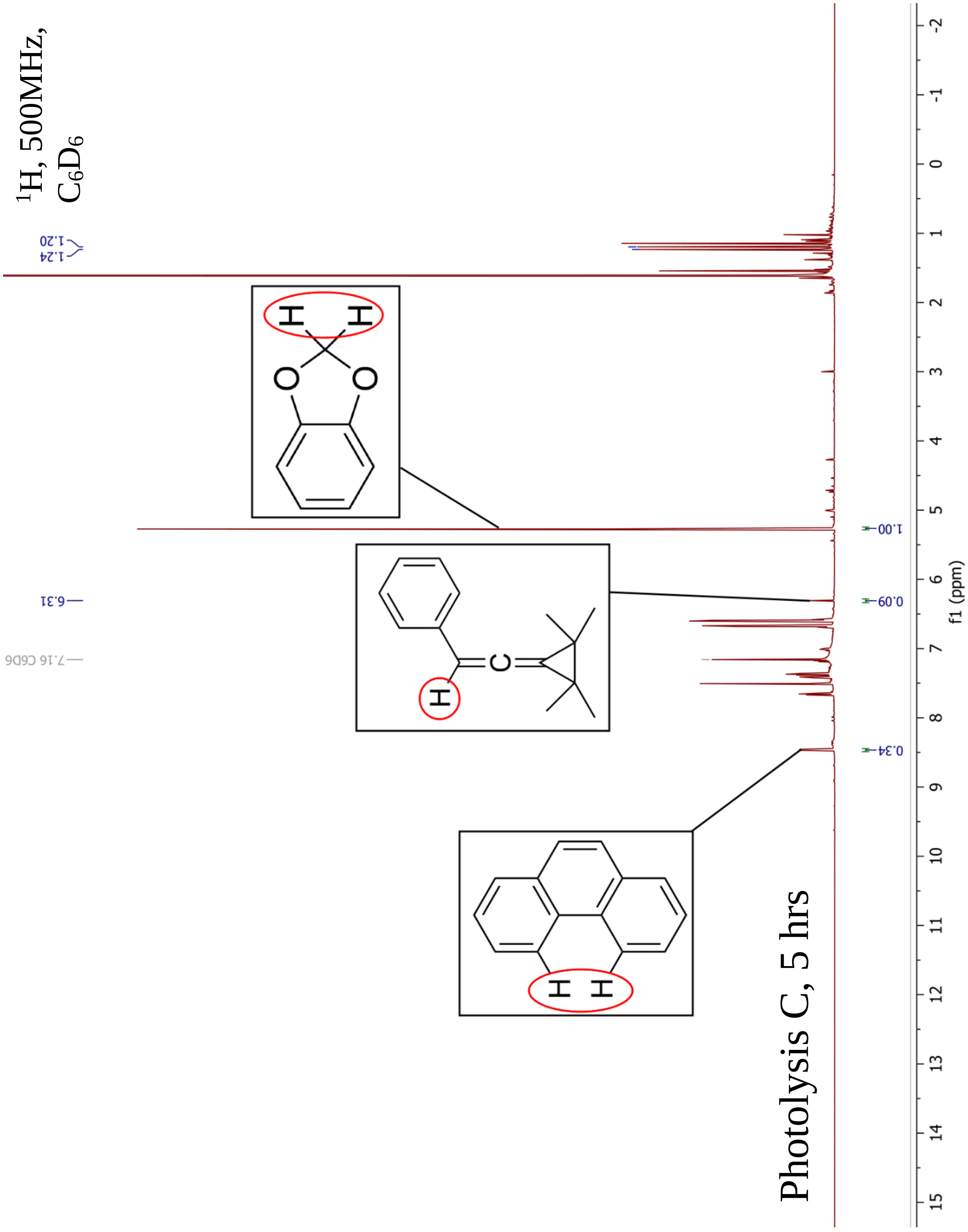
Photolysis C, 5 hrs
1H, 500MHz, C6D6
1.24
1.20
6.31
7.16 C6D6
1.00
0.09
0.34
f1 (ppm)

^{1}H, 500MHz, C_6D_6

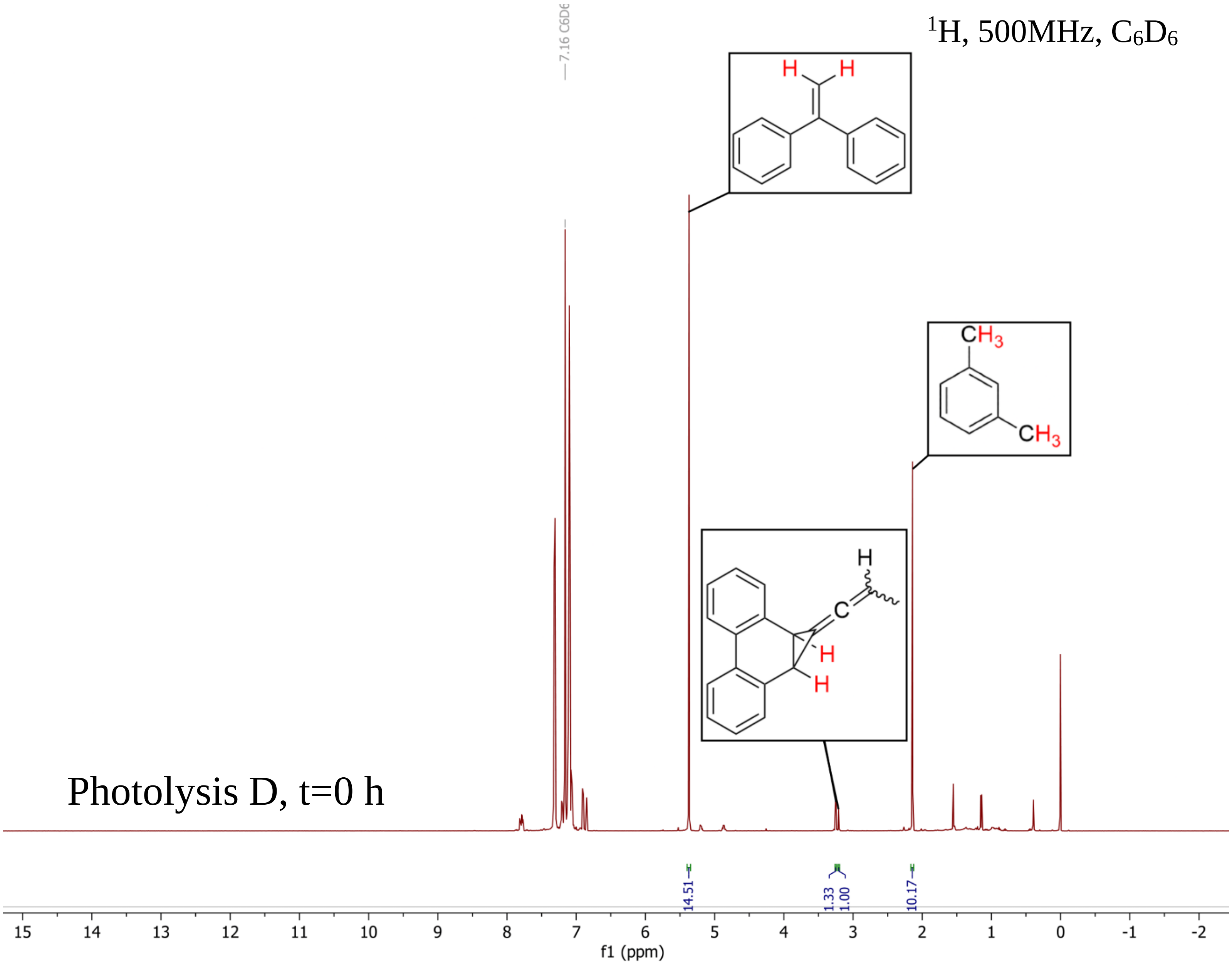

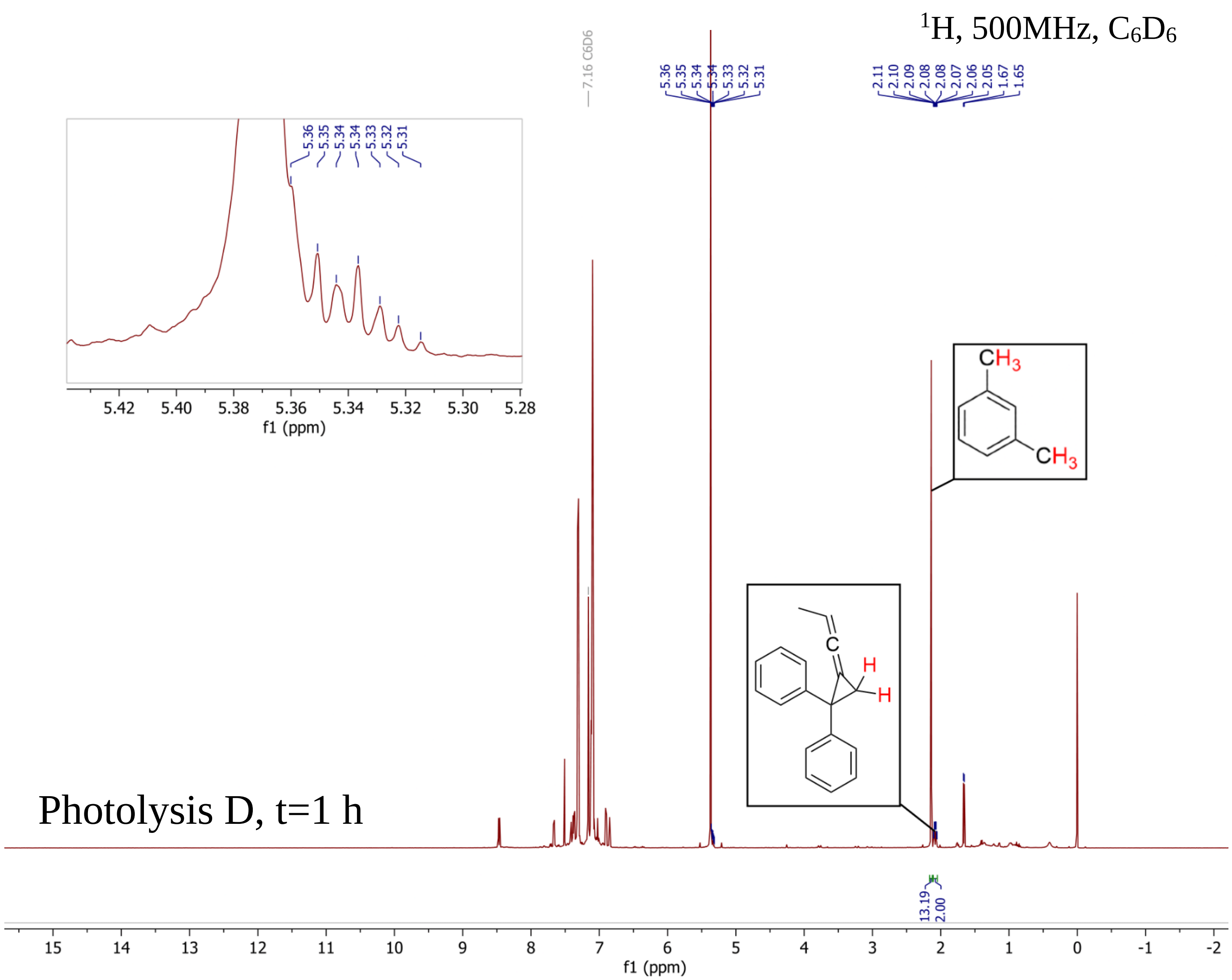

^1H, 500MHz, C_6D_6
7.16 C6D6
5.36 5.35 5.34 5.34 5.33 5.32 5.31
2.11 2.10 2.09 2.08 2.08 2.07 2.06 2.05 1.67 1.65
5.42 5.40 5.38 5.36 5.34 5.32 5.30 5.28
f1 (ppm)
CH3
CH3
C
H
H
Photolysis D, t=1 h
13.19
2.00
15 14 13 12 11 10 9 8 7 6 5 4 3 2 1 0 -1 -2
f1 (ppm)

Optimized energies, coordinates, and frequencies for B2PLYP/def2-TZVP calculations:

1 (singlet)

```
------------
INNER ENERGY
------------

The inner energy is: U= E(el) + E(ZPE) + E(vib) + E(rot) + E(trans)
   E(el)   - is the total energy from the electronic structure calculation
           = E(kin-el) + E(nuc-el) + E(el-el) + E(nuc-nuc)
   E(ZPE)  - the the zero temperature vibrational energy from the frequency calculation
   E(vib)  - the the finite temperature correction to E(ZPE) due to population
           of excited vibrational states
   E(rot)  - is the rotational thermal energy
   E(trans)- is the translational thermal energy

Summary of contributions to the inner energy U:
Electronic energy                ...   -115.27152766 Eh
Zero point energy                ...      0.03105177 Eh      19.49 kcal/mol
Thermal vibrational correction   ...      0.00103266 Eh       0.65 kcal/mol
Thermal rotational correction    ...      0.00141627 Eh       0.89 kcal/mol
Thermal translational correction ...      0.00141627 Eh       0.89 kcal/mol
-----------------------------------------------------------------------
Total thermal energy                   -115.23661069 Eh


Summary of corrections to the electronic energy:
(perhaps to be used in another calculation)
Total thermal correction                  0.00386520 Eh       2.43 kcal/mol
Non-thermal (ZPE) correction              0.03105177 Eh      19.49 kcal/mol
-----------------------------------------------------------------------
Total correction                          0.03491697 Eh      21.91 kcal/mol

---------------------------------
CARTESIAN COORDINATES (ANGSTROEM)
---------------------------------
  C     -0.597094    0.000005   -3.805400
  C     -0.853328   -0.000004   -2.548329
  C     -1.117847   -0.000013   -1.249912
  H     -2.137726    0.000006   -0.877075
  H     -0.324429    0.000006   -0.508644
```

No imaginary frequency.

```
-----------------------------
SCF STABILITY ANALYSIS RESULT
```

RHF/RKS->UHF/UKS - triplet - external

Root	Eigenvalue (au)
0	0.008608
1	0.017820
2	0.029274

Stability Analysis indicates a stable HF/KS wave function.

1 (triplet)

INNER ENERGY

The inner energy is: U= E(el) + E(ZPE) + E(vib) + E(rot) + E(trans)
E(el) - is the total energy from the electronic structure calculation
= E(kin-el) + E(nuc-el) + E(el-el) + E(nuc-nuc)
E(ZPE) - the the zero temperature vibrational energy from the frequency calculation
E(vib) - the the finite temperature correction to E(ZPE) due to population
of excited vibrational states
E(rot) - is the rotational thermal energy
E(trans)- is the translational thermal energy

Summary of contributions to the inner energy U:
Electronic energy ... -115.22231137 Eh
Zero point energy ... 0.03052828 Eh 19.16 kcal/mol
Thermal vibrational correction ... 0.00099520 Eh 0.62 kcal/mol
Thermal rotational correction ... 0.00141627 Eh 0.89 kcal/mol
Thermal translational correction ... 0.00141627 Eh 0.89 kcal/mol
----
Total thermal energy -115.18795534 Eh

Summary of corrections to the electronic energy:
(perhaps to be used in another calculation)
Total thermal correction 0.00382774 Eh 2.40 kcal/mol
Non-thermal (ZPE) correction 0.03052828 Eh 19.16 kcal/mol

Total correction 0.03435603 Eh 21.56 kcal/mol

CARTESIAN COORDINATES (ANGSTROEM)

C -0.608647 -0.000002 -3.777395

C	-0.851141	0.000003	-2.573978
C	-1.118442	-0.000001	-1.238111
H	-2.137909	0.000000	-0.882310
H	-0.314284	-0.000001	-0.517565

No imaginary frequency.

SCF STABILITY ANALYSIS RESULT

UHF/UKS->UHF/UKS - singlet - internal

Root	Eigenvalue (au)
0	0.032626
1	0.091922
2	0.126983

Stability Analysis indicates a stable HF/KS wave function.

<u>36a (singlet)</u>

INNER ENERGY

The inner energy is: U= E(el) + E(ZPE) + E(vib) + E(rot) + E(trans)
E(el) - is the total energy from the electronic structure calculation
= E(kin-el) + E(nuc-el) + E(el-el) + E(nuc-nuc)
E(ZPE) - the the zero temperature vibrational energy from the frequency calculation
E(vib) - the the finite temperature correction to E(ZPE) due to population
of excited vibrational states
E(rot) - is the rotational thermal energy
E(trans)- is the translational thermal energy

Summary of contributions to the inner energy U:
Electronic energy ... -346.23185183 Eh
Zero point energy ... 0.11422747 Eh 71.68 kcal/mol
Thermal vibrational correction ... 0.00468176 Eh 2.94 kcal/mol
Thermal rotational correction ... 0.00141627 Eh 0.89 kcal/mol
Thermal translational correction ... 0.00141627 Eh 0.89 kcal/mol
----
Total thermal energy -346.11011005 Eh

Summary of corrections to the electronic energy:
(perhaps to be used in another calculation)

Total thermal correction 0.00751430 Eh 4.72 kcal/mol
Non-thermal (ZPE) correction 0.11422747 Eh 71.68 kcal/mol

Total correction 0.12174177 Eh 76.39 kcal/mol

CARTESIAN COORDINATES (ANGSTROEM)

C	-0.511889	-0.280015	-4.123601
C	-0.827982	-0.128469	-2.898974
C	-1.160331	0.032820	-1.607327
H	-2.158202	0.397135	-1.364878
C	-0.302073	-0.239248	-0.479445
C	-0.788500	-0.022969	0.819613
C	0.018806	-0.270535	1.918832
C	1.315324	-0.742546	1.730146
C	1.807414	-0.965244	0.442227
C	1.008717	-0.714416	-0.658211
H	-1.798952	0.342691	0.952701
H	-0.356199	-0.100554	2.918629
H	1.945268	-0.937545	2.587804
H	2.815152	-1.332620	0.305849
H	1.371483	-0.875488	-1.664980

No imaginary frequency.

SCF STABILITY ANALYSIS RESULT

RHF/RKS->UHF/UKS - triplet - external

Root	Eigenvalue (au)
0	0.011036
1	0.023436
2	0.042201

Stability Analysis indicates a stable HF/KS wave function.

36a (triplet)

INNER ENERGY

The inner energy is: U= E(el) + E(ZPE) + E(vib) + E(rot) + E(trans)
E(el) - is the total energy from the electronic structure calculation

= E(kin-el) + E(nuc-el) + E(el-el) + E(nuc-nuc)
E(ZPE) - the the zero temperature vibrational energy from the frequency calculation
E(vib) - the the finite temperature correction to E(ZPE) due to population
of excited vibrational states
E(rot) - is the rotational thermal energy
E(trans)- is the translational thermal energy

Summary of contributions to the inner energy U:
Electronic energy ... -346.17461064 Eh
Zero point energy ... 0.11378914 Eh 71.40 kcal/mol
Thermal vibrational correction ... 0.00464924 Eh 2.92 kcal/mol
Thermal rotational correction ... 0.00141627 Eh 0.89 kcal/mol
Thermal translational correction ... 0.00141627 Eh 0.89 kcal/mol
----
Total thermal energy -346.05333971 Eh

Summary of corrections to the electronic energy:
(perhaps to be used in another calculation)
Total thermal correction 0.00748178 Eh 4.69 kcal/mol
Non-thermal (ZPE) correction 0.11378914 Eh 71.40 kcal/mol

Total correction 0.12127093 Eh 76.10 kcal/mol

CARTESIAN COORDINATES (ANGSTROEM)

C	-0.474695	-0.285251	-4.082038
C	-0.823371	-0.127648	-2.921105
C	-1.180019	0.041326	-1.599348
H	-2.178493	0.407009	-1.394399
C	-0.315570	-0.237091	-0.484220
C	-0.783840	-0.023037	0.823013
C	0.027734	-0.271687	1.915777
C	1.323208	-0.744472	1.731154
C	1.799025	-0.965345	0.440637
C	0.994653	-0.715695	-0.655669
H	-1.791701	0.345139	0.968508
H	-0.348335	-0.098549	2.915001
H	1.957521	-0.939700	2.585001
H	2.805069	-1.334648	0.292742
H	1.366848	-0.887354	-1.656671

No imaginary frequency.

SCF STABILITY ANALYSIS RESULT

UHF/UKS->UHF/UKS - singlet - internal

Root	Eigenvalue (au)
0	0.034660
1	0.064157
2	0.143694

Stability Analysis indicates a stable HF/KS wave function.

36b (singlet)

INNER ENERGY

The inner energy is: U= E(el) + E(ZPE) + E(vib) + E(rot) + E(trans)
E(el) - is the total energy from the electronic structure calculation
= E(kin-el) + E(nuc-el) + E(el-el) + E(nuc-nuc)
E(ZPE) - the the zero temperature vibrational energy from the frequency calculation
E(vib) - the the finite temperature correction to E(ZPE) due to population
of excited vibrational states
E(rot) - is the rotational thermal energy
E(trans)- is the translational thermal energy

Summary of contributions to the inner energy U:
Electronic energy ... -154.57288048 Eh
Zero point energy ... 0.06013856 Eh 37.74 kcal/mol
Thermal vibrational correction ... 0.00218026 Eh 1.37 kcal/mol
Thermal rotational correction ... 0.00141627 Eh 0.89 kcal/mol
Thermal translational correction ... 0.00141627 Eh 0.89 kcal/mol
----
Total thermal energy -154.50772912 Eh

Summary of corrections to the electronic energy:
(perhaps to be used in another calculation)
Total thermal correction 0.00501280 Eh 3.15 kcal/mol
Non-thermal (ZPE) correction 0.06013856 Eh 37.74 kcal/mol

Total correction 0.06515136 Eh 40.88 kcal/mol

CARTESIAN COORDINATES (ANGSTROEM)

C	-3.476646	2.041106	0.432848
C	-2.267564	2.335811	0.143140
C	-1.006666	2.641213	-0.159806
C	-0.387916	3.980542	0.013694
H	-0.022102	4.338256	-0.952952
H	0.490243	3.895093	0.659598
H	-1.087045	4.698214	0.431898
H	-0.363752	1.862254	-0.568410

No imaginary frequency.

36b (triplet)

INNER ENERGY

The inner energy is: U= E(el) + E(ZPE) + E(vib) + E(rot) + E(trans)
E(el) - is the total energy from the electronic structure calculation
= E(kin-el) + E(nuc-el) + E(el-el) + E(nuc-nuc)
E(ZPE) - the the zero temperature vibrational energy from the frequency calculation
E(vib) - the the finite temperature correction to E(ZPE) due to population
of excited vibrational states
E(rot) - is the rotational thermal energy
E(trans)- is the translational thermal energy

Summary of contributions to the inner energy U:
Electronic energy ... -154.51592407 Eh
Zero point energy ... 0.05962885 Eh 37.42 kcal/mol
Thermal vibrational correction ... 0.00222986 Eh 1.40 kcal/mol
Thermal rotational correction ... 0.00141627 Eh 0.89 kcal/mol
Thermal translational correction ... 0.00141627 Eh 0.89 kcal/mol
----
Total thermal energy -154.45123282 Eh

Summary of corrections to the electronic energy:
(perhaps to be used in another calculation)
Total thermal correction 0.00506240 Eh 3.18 kcal/mol
Non-thermal (ZPE) correction 0.05962885 Eh 37.42 kcal/mol

Total correction 0.06469125 Eh 40.59 kcal/mol

CARTESIAN COORDINATES (ANGSTROEM)

C	-3.448247	2.087632	0.444008

C	-2.279867	2.319282	0.144979
C	-0.985981	2.618210	-0.175281
C	-0.384920	3.972694	0.009448
H	-0.022512	4.369185	-0.942834
H	0.477328	3.926238	0.680094
H	-1.106844	4.673099	0.422891
H	-0.370408	1.826148	-0.583296

No imaginary frequency.

Optimized energies, coordinates, and frequencies for B3LYP/def2-TZVP calculations:

1 (singlet)

```
------------
INNER ENERGY
------------

The inner energy is: U= E(el) + E(ZPE) + E(vib) + E(rot) + E(trans)
    E(el)   - is the total energy from the electronic structure calculation
              = E(kin-el) + E(nuc-el) + E(el-el) + E(nuc-nuc)
    E(ZPE)  - the the zero temperature vibrational energy from the frequency calculation
    E(vib)  - the the finite temperature correction to E(ZPE) due to population
              of excited vibrational states
    E(rot)  - is the rotational thermal energy
    E(trans)- is the translational thermal energy

Summary of contributions to the inner energy U:
Electronic energy                ...   -115.31152696 Eh
Zero point energy                ...      0.03096711 Eh      19.43 kcal/mol
Thermal vibrational correction   ...      0.00100863 Eh       0.63 kcal/mol
Thermal rotational correction    ...      0.00141627 Eh       0.89 kcal/mol
Thermal translational correction ...      0.00141627 Eh       0.89 kcal/mol
-----------------------------------------------------------------------
Total thermal energy                   -115.27671868 Eh


Summary of corrections to the electronic energy:
(perhaps to be used in another calculation)
Total thermal correction                  0.00384117 Eh       2.41 kcal/mol
Non-thermal (ZPE) correction              0.03096711 Eh      19.43 kcal/mol
-----------------------------------------------------------------------
Total correction                          0.03480828 Eh      21.84 kcal/mol

--------------------------------
CARTESIAN COORDINATES (ANGSTROEM)
--------------------------------
```

C	-0.597498	0.000002	-3.801727
C	-0.853330	-0.000002	-2.547010
C	-1.117307	-0.000007	-1.253055
H	-2.137994	0.000003	-0.878319
H	-0.324294	0.000003	-0.509247

No imaginary frequency.

```
----------------------------
SCF STABILITY ANALYSIS RESULT
----------------------------

RHF/RKS->UHF/UKS - triplet - external

   Root    Eigenvalue (au)
     0      0.014350
     1      0.035828
     2      0.055351

Stability Analysis indicates a stable HF/KS wave function.
```

1 (triplet)

```
------------
INNER ENERGY
------------

The inner energy is: U= E(el) + E(ZPE) + E(vib) + E(rot) + E(trans)
   E(el)   - is the total energy from the electronic structure calculation
           = E(kin-el) + E(nuc-el) + E(el-el) + E(nuc-nuc)
   E(ZPE)  - the the zero temperature vibrational energy from the frequency calculation
   E(vib)  - the the finite temperature correction to E(ZPE) due to population
           of excited vibrational states
   E(rot)  - is the rotational thermal energy
   E(trans)- is the translational thermal energy

Summary of contributions to the inner energy U:
Electronic energy                ...   -115.22231031 Eh
Zero point energy                ...      0.03050313 Eh     19.14 kcal/mol
Thermal vibrational correction   ...      0.00098869 Eh      0.62 kcal/mol
Thermal rotational correction    ...      0.00141627 Eh      0.89 kcal/mol
Thermal translational correction ...      0.00141627 Eh      0.89 kcal/mol
-----------------------------------------------------------------------
Total thermal energy                   -115.18798595 Eh


Summary of corrections to the electronic energy:
```

(perhaps to be used in another calculation)
Total thermal correction 0.00382123 Eh 2.40 kcal/mol
Non-thermal (ZPE) correction 0.03050313 Eh 19.14 kcal/mol

Total correction 0.03432436 Eh 21.54 kcal/mol

CARTESIAN COORDINATES (ANGSTROEM)

C	-0.608509	-0.000002	-3.777548
C	-0.851222	0.000003	-2.574200
C	-1.118418	-0.000001	-1.238343
H	-2.137617	-0.000000	-0.881894
H	-0.314656	-0.000001	-0.517373

No imaginary frequency.

SCF STABILITY ANALYSIS RESULT

UHF/UKS->UHF/UKS - singlet - internal

Root	Eigenvalue (au)
0	0.008342
1	0.058810
2	0.113789

Stability Analysis indicates a stable HF/KS wave function.

36a (singlet)

INNER ENERGY

The inner energy is: U= E(el) + E(ZPE) + E(vib) + E(rot) + E(trans)
E(el) - is the total energy from the electronic structure calculation
= E(kin-el) + E(nuc-el) + E(el-el) + E(nuc-nuc)
E(ZPE) - the the zero temperature vibrational energy from the frequency calculation
E(vib) - the the finite temperature correction to E(ZPE) due to population
of excited vibrational states
E(rot) - is the rotational thermal energy
E(trans)- is the translational thermal energy

Summary of contributions to the inner energy U:
Electronic energy ... -346.34507521 Eh

Zero point energy ... 0.11414871 Eh 71.63 kcal/mol
Thermal vibrational correction ... 0.00464900 Eh 2.92 kcal/mol
Thermal rotational correction ... 0.00141627 Eh 0.89 kcal/mol
Thermal translational correction ... 0.00141627 Eh 0.89 kcal/mol
----
Total thermal energy -346.22344495 Eh

Summary of corrections to the electronic energy:
(perhaps to be used in another calculation)
Total thermal correction 0.00748154 Eh 4.69 kcal/mol
Non-thermal (ZPE) correction 0.11414871 Eh 71.63 kcal/mol

Total correction 0.12163025 Eh 76.32 kcal/mol

CARTESIAN COORDINATES (ANGSTROEM)

C	-0.526649	-0.282638	-4.122626
C	-0.833333	-0.130320	-2.900179
C	-1.157114	0.032088	-1.610114
H	-2.154216	0.398726	-1.365448
C	-0.299467	-0.236933	-0.482160
C	-0.784929	-0.020392	0.817423
C	0.017730	-0.272947	1.917222
C	1.313938	-0.744304	1.731131
C	1.808695	-0.962515	0.444349
C	1.012049	-0.712295	-0.655809
H	-1.795513	0.346059	0.950840
H	-0.360764	-0.104641	2.916687
H	1.942645	-0.943014	2.589725
H	2.817955	-1.328311	0.308922
H	1.377008	-0.875564	-1.661577

No imaginary frequency.

SCF STABILITY ANALYSIS RESULT

RHF/RKS->UHF/UKS - triplet - external

Root	Eigenvalue (au)
0	0.025276
1	0.044945
2	0.046689

Stability Analysis indicates a stable HF/KS wave function.

36a (triplet)

INNER ENERGY

The inner energy is: U= E(el) + E(ZPE) + E(vib) + E(rot) + E(trans)
E(el) - is the total energy from the electronic structure calculation
= E(kin-el) + E(nuc-el) + E(el-el) + E(nuc-nuc)
E(ZPE) - the the zero temperature vibrational energy from the frequency calculation
E(vib) - the the finite temperature correction to E(ZPE) due to population
of excited vibrational states
E(rot) - is the rotational thermal energy
E(trans)- is the translational thermal energy

Summary of contributions to the inner energy U:
Electronic energy ... -346.17461064 Eh
Zero point energy ... 0.11378914 Eh 71.40 kcal/mol
Thermal vibrational correction ... 0.00464924 Eh 2.92 kcal/mol
Thermal rotational correction ... 0.00141627 Eh 0.89 kcal/mol
Thermal translational correction ... 0.00141627 Eh 0.89 kcal/mol
----
Total thermal energy -346.05333971 Eh

Summary of corrections to the electronic energy:
(perhaps to be used in another calculation)
Total thermal correction 0.00748178 Eh 4.69 kcal/mol
Non-thermal (ZPE) correction 0.11378914 Eh 71.40 kcal/mol

Total correction 0.12127093 Eh 76.10 kcal/mol

CARTESIAN COORDINATES (ANGSTROEM)

C	-0.474695	-0.285251	-4.082038
C	-0.823371	-0.127648	-2.921105
C	-1.180019	0.041326	-1.599348
H	-2.178493	0.407009	-1.394399
C	-0.315570	-0.237091	-0.484220
C	-0.783840	-0.023037	0.823013
C	0.027734	-0.271687	1.915777
C	1.323208	-0.744472	1.731154
C	1.799025	-0.965345	0.440637

C	0.994653	-0.715695	-0.655669
H	-1.791701	0.345139	0.968508
H	-0.348335	-0.098549	2.915001
H	1.957521	-0.939700	2.585001
H	2.805069	-1.334648	0.292742
H	1.366848	-0.887354	-1.656671

No imaginary frequency.

SCF STABILITY ANALYSIS RESULT

UHF/UKS->UHF/UKS - singlet - internal

Root	Eigenvalue (au)
0	0.010470
1	0.025316
2	0.055789

Stability Analysis indicates a stable HF/KS wave function.

36b (singlet)

INNER ENERGY

The inner energy is: U= E(el) + E(ZPE) + E(vib) + E(rot) + E(trans)
E(el) - is the total energy from the electronic structure calculation
= E(kin-el) + E(nuc-el) + E(el-el) + E(nuc-nuc)
E(ZPE) - the the zero temperature vibrational energy from the frequency calculation
E(vib) - the the finite temperature correction to E(ZPE) due to population
of excited vibrational states
E(rot) - is the rotational thermal energy
E(trans)- is the translational thermal energy

Summary of contributions to the inner energy U:
Electronic energy ... -154.62823541 Eh
Zero point energy ... 0.05977667 Eh 37.51 kcal/mol
Thermal vibrational correction ... 0.00217357 Eh 1.36 kcal/mol
Thermal rotational correction ... 0.00141627 Eh 0.89 kcal/mol
Thermal translational correction ... 0.00141627 Eh 0.89 kcal/mol
----
Total thermal energy -154.56345262 Eh

Summary of corrections to the electronic energy:
(perhaps to be used in another calculation)
Total thermal correction 0.00500612 Eh 3.14 kcal/mol
Non-thermal (ZPE) correction 0.05977667 Eh 37.51 kcal/mol

Total correction 0.06478279 Eh 40.65 kcal/mol

CARTESIAN COORDINATES (ANGSTROEM)

C	-3.471223	2.039683	0.434952
C	-2.266615	2.334922	0.143230
C	-1.011007	2.642326	-0.161172
C	-0.389910	3.979607	0.013346
H	-0.016786	4.338000	-0.952399
H	0.488440	3.893548	0.662350
H	-1.087659	4.701586	0.429730
H	-0.366687	1.862817	-0.570026

No imaginary frequency.

36b (triplet)

INNER ENERGY

The inner energy is: U= E(el) + E(ZPE) + E(vib) + E(rot) + E(trans)
E(el) - is the total energy from the electronic structure calculation
= E(kin-el) + E(nuc-el) + E(el-el) + E(nuc-nuc)
E(ZPE) - the the zero temperature vibrational energy from the frequency calculation
E(vib) - the the finite temperature correction to E(ZPE) due to population
of excited vibrational states
E(rot) - is the rotational thermal energy
E(trans)- is the translational thermal energy

Summary of contributions to the inner energy U:
Electronic energy ... -154.51592539 Eh
Zero point energy ... 0.05961756 Eh 37.41 kcal/mol
Thermal vibrational correction ... 0.00223838 Eh 1.40 kcal/mol
Thermal rotational correction ... 0.00141627 Eh 0.89 kcal/mol
Thermal translational correction ... 0.00141627 Eh 0.89 kcal/mol
----
Total thermal energy -154.45123691 Eh

Summary of corrections to the electronic energy:

(perhaps to be used in another calculation)
Total thermal correction 0.00507092 Eh 3.18 kcal/mol
Non-thermal (ZPE) correction 0.05961756 Eh 37.41 kcal/mol

Total correction 0.06468848 Eh 40.59 kcal/mol

CARTESIAN COORDINATES (ANGSTROEM)

C	-3.448248	2.087701	0.443926
C	-2.279852	2.319271	0.144931
C	-0.986034	2.618112	-0.175254
C	-0.384926	3.972613	0.009421
H	-0.022676	4.369271	-0.942844
H	0.477349	3.926324	0.680040
H	-1.106707	4.673100	0.422987
H	-0.370358	1.826096	-0.583198

No imaginary frequency.

Optimized energies, coordinates, and frequencies for PBE0/def2-TZVP calculations:

<u>1 (singlet)</u>

INNER ENERGY

The inner energy is: U= E(el) + E(ZPE) + E(vib) + E(rot) + E(trans)
E(el) - is the total energy from the electronic structure calculation
= E(kin-el) + E(nuc-el) + E(el-el) + E(nuc-nuc)
E(ZPE) - the the zero temperature vibrational energy from the frequency calculation
E(vib) - the the finite temperature correction to E(ZPE) due to population
of excited vibrational states
E(rot) - is the rotational thermal energy
E(trans)- is the translational thermal energy

Summary of contributions to the inner energy U:
Electronic energy ... -115.22344943 Eh
Zero point energy ... 0.03108226 Eh 19.50 kcal/mol
Thermal vibrational correction ... 0.00101793 Eh 0.64 kcal/mol
Thermal rotational correction ... 0.00141627 Eh 0.89 kcal/mol
Thermal translational correction ... 0.00141627 Eh 0.89 kcal/mol
----
Total thermal energy -115.18851671 Eh

Summary of corrections to the electronic energy:
(perhaps to be used in another calculation)
Total thermal correction 0.00385047 Eh 2.42 kcal/mol
Non-thermal (ZPE) correction 0.03108226 Eh 19.50 kcal/mol

Total correction 0.03493273 Eh 21.92 kcal/mol

CARTESIAN COORDINATES (ANGSTROEM)

C	-0.597200	0.000003	-3.801062
C	-0.853627	-0.000002	-2.545346
C	-1.117479	-0.000008	-1.252945
H	-2.139880	0.000004	-0.879955
H	-0.322237	0.000004	-0.510050

No imaginary frequency.

SCF STABILITY ANALYSIS RESULT

RHF/RKS->UHF/UKS - triplet - external

Root	Eigenvalue (au)
0	0.006319
1	0.030462
2	0.043034

Stability Analysis indicates a stable HF/KS wave function.

1 (triplet)

INNER ENERGY

The inner energy is: U= E(el) + E(ZPE) + E(vib) + E(rot) + E(trans)
E(el) - is the total energy from the electronic structure calculation
= E(kin-el) + E(nuc-el) + E(el-el) + E(nuc-nuc)
E(ZPE) - the the zero temperature vibrational energy from the frequency calculation
E(vib) - the the finite temperature correction to E(ZPE) due to population
of excited vibrational states
E(rot) - is the rotational thermal energy
E(trans)- is the translational thermal energy

Summary of contributions to the inner energy U:

Electronic energy ... -115.18539772 Eh
Zero point energy ... 0.03039899 Eh 19.08 kcal/mol
Thermal vibrational correction ... 0.00096738 Eh 0.61 kcal/mol
Thermal rotational correction ... 0.00141627 Eh 0.89 kcal/mol
Thermal translational correction ... 0.00141627 Eh 0.89 kcal/mol
----
Total thermal energy -115.15119880 Eh

Summary of corrections to the electronic energy:
(perhaps to be used in another calculation)
Total thermal correction 0.00379993 Eh 2.38 kcal/mol
Non-thermal (ZPE) correction 0.03039899 Eh 19.08 kcal/mol

Total correction 0.03419892 Eh 21.46 kcal/mol

CARTESIAN COORDINATES (ANGSTROEM)

C	-0.604688	0.000000	-3.775760
C	-0.848989	-0.000000	-2.569753
C	-1.119056	-0.000000	-1.241615
H	-2.141847	0.000000	-0.886727
H	-0.315843	0.000000	-0.515504

No imaginary frequency.

SCF STABILITY ANALYSIS RESULT

UHF/UKS->UHF/UKS - singlet - internal

Root	Eigenvalue (au)
0	0.019543
1	0.076985
2	0.117494

Stability Analysis indicates a stable HF/KS wave function.

36a (singlet)

INNER ENERGY

The inner energy is: U= E(el) + E(ZPE) + E(vib) + E(rot) + E(trans)

E(el)   - is the total energy from the electronic structure calculation
= E(kin-el) + E(nuc-el) + E(el-el) + E(nuc-nuc)
E(ZPE) - the the zero temperature vibrational energy from the frequency calculation
E(vib) - the the finite temperature correction to E(ZPE) due to population
of excited vibrational states
E(rot) - is the rotational thermal energy
E(trans)- is the translational thermal energy

Summary of contributions to the inner energy U:
Electronic energy ... -346.11459932 Eh
Zero point energy ... 0.11468036 Eh 71.96 kcal/mol
Thermal vibrational correction ... 0.00465950 Eh 2.92 kcal/mol
Thermal rotational correction ... 0.00141627 Eh 0.89 kcal/mol
Thermal translational correction ... 0.00141627 Eh 0.89 kcal/mol
----
Total thermal energy -345.99242693 Eh

Summary of corrections to the electronic energy:
(perhaps to be used in another calculation)
Total thermal correction 0.00749204 Eh 4.70 kcal/mol
Non-thermal (ZPE) correction 0.11468036 Eh 71.96 kcal/mol

Total correction 0.12217240 Eh 76.66 kcal/mol

CARTESIAN COORDINATES (ANGSTROEM)

C	-0.516854	-0.285749	-4.114733
C	-0.825163	-0.131964	-2.891510
C	-1.153799	0.032184	-1.604522
H	-2.153190	0.399292	-1.363758
C	-0.299342	-0.238061	-0.478398
C	-0.784974	-0.023276	0.816940
C	0.018384	-0.271652	1.913328
C	1.311205	-0.742856	1.725419
C	1.803811	-0.962952	0.441616
C	1.008254	-0.711345	-0.655393
H	-1.797371	0.343066	0.949142
H	-0.357753	-0.102182	2.914888
H	1.941137	-0.940629	2.585042
H	2.813582	-1.330444	0.305308
H	1.370107	-0.870434	-1.664986

No imaginary frequency.

SCF STABILITY ANALYSIS RESULT

RHF/RKS->UHF/UKS - triplet - external

Root	Eigenvalue (au)
0	0.018911
1	0.035826
2	0.042850

Stability Analysis indicates a stable HF/KS wave function.

36a (triplet)

INNER ENERGY

The inner energy is: U= E(el) + E(ZPE) + E(vib) + E(rot) + E(trans)
E(el) - is the total energy from the electronic structure calculation
= E(kin-el) + E(nuc-el) + E(el-el) + E(nuc-nuc)
E(ZPE) - the the zero temperature vibrational energy from the frequency calculation
E(vib) - the the finite temperature correction to E(ZPE) due to population
of excited vibrational states
E(rot) - is the rotational thermal energy
E(trans)- is the translational thermal energy

Summary of contributions to the inner energy U:
Electronic energy ... -346.06983822 Eh
Zero point energy ... 0.11369082 Eh 71.34 kcal/mol
Thermal vibrational correction ... 0.00463458 Eh 2.91 kcal/mol
Thermal rotational correction ... 0.00141627 Eh 0.89 kcal/mol
Thermal translational correction ... 0.00141627 Eh 0.89 kcal/mol
----
Total thermal energy -345.94868028 Eh

Summary of corrections to the electronic energy:
(perhaps to be used in another calculation)
Total thermal correction 0.00746713 Eh 4.69 kcal/mol
Non-thermal (ZPE) correction 0.11369082 Eh 71.34 kcal/mol

Total correction 0.12115794 Eh 76.03 kcal/mol

CARTESIAN COORDINATES (ANGSTROEM)

C	-0.490810	-0.292035	-4.082204
C	-0.821074	-0.135471	-2.914707
C	-1.171632	0.037220	-1.597148
H	-2.172616	0.404487	-1.392236
C	-0.313125	-0.233884	-0.486080
C	-0.783170	-0.022993	0.821376
C	0.024943	-0.273524	1.912295
C	1.320054	-0.744782	1.729398
C	1.799406	-0.960762	0.440853
C	0.999123	-0.709485	-0.654219
H	-1.793884	0.343865	0.964842
H	-0.353079	-0.102932	2.913579
H	1.952840	-0.943109	2.586254
H	2.808554	-1.328161	0.294422
H	1.372504	-0.875436	-1.658041

No imaginary frequency.

SCF STABILITY ANALYSIS RESULT

UHF/UKS->UHF/UKS - singlet - internal

Root	Eigenvalue (au)
0	0.022313
1	0.043634
2	0.084090

Stability Analysis indicates a stable HF/KS wave function.

36b (singlet)

INNER ENERGY

The inner energy is: U= E(el) + E(ZPE) + E(vib) + E(rot) + E(trans)
E(el) - is the total energy from the electronic structure calculation
= E(kin-el) + E(nuc-el) + E(el-el) + E(nuc-nuc)
E(ZPE) - the the zero temperature vibrational energy from the frequency calculation
E(vib) - the the finite temperature correction to E(ZPE) due to population
of excited vibrational states
E(rot) - is the rotational thermal energy
E(trans)- is the translational thermal energy

Summary of contributions to the inner energy U:
Electronic energy ... -154.51765773 Eh
Zero point energy ... 0.06000494 Eh 37.65 kcal/mol
Thermal vibrational correction ... 0.00217658 Eh 1.37 kcal/mol
Thermal rotational correction ... 0.00141627 Eh 0.89 kcal/mol
Thermal translational correction ... 0.00141627 Eh 0.89 kcal/mol
----
Total thermal energy -154.45264366 Eh

Summary of corrections to the electronic energy:
(perhaps to be used in another calculation)
Total thermal correction 0.00500912 Eh 3.14 kcal/mol
Non-thermal (ZPE) correction 0.06000494 Eh 37.65 kcal/mol

Total correction 0.06501406 Eh 40.80 kcal/mol

CARTESIAN COORDINATES (ANGSTROEM)

C	-3.469306	2.040998	0.435557
C	-2.263733	2.337825	0.143874
C	-1.008743	2.645814	-0.160243
C	-0.392456	3.977510	0.013537
H	-0.016240	4.334254	-0.952055
H	0.486310	3.891112	0.662674
H	-1.090927	4.700174	0.428872
H	-0.366353	1.864803	-0.572206

No imaginary frequency.

36b (triplet)

INNER ENERGY

The inner energy is: U= E(el) + E(ZPE) + E(vib) + E(rot) + E(trans)
E(el) - is the total energy from the electronic structure calculation
= E(kin-el) + E(nuc-el) + E(el-el) + E(nuc-nuc)
E(ZPE) - the the zero temperature vibrational energy from the frequency calculation
E(vib) - the the finite temperature correction to E(ZPE) due to population
of excited vibrational states
E(rot) - is the rotational thermal energy
E(trans)- is the translational thermal energy

Summary of contributions to the inner energy U:

Electronic energy ... -154.47155740 Eh
Zero point energy ... 0.05946273 Eh 37.31 kcal/mol
Thermal vibrational correction ... 0.00218343 Eh 1.37 kcal/mol
Thermal rotational correction ... 0.00141627 Eh 0.89 kcal/mol
Thermal translational correction ... 0.00141627 Eh 0.89 kcal/mol
----
Total thermal energy -154.40707870 Eh

Summary of corrections to the electronic energy:
(perhaps to be used in another calculation)
Total thermal correction 0.00501597 Eh 3.15 kcal/mol
Non-thermal (ZPE) correction 0.05946273 Eh 37.31 kcal/mol

Total correction 0.06447870 Eh 40.46 kcal/mol

CARTESIAN COORDINATES (ANGSTROEM)

C	-3.445083	2.085379	0.449228
C	-2.276632	2.323713	0.146607
C	-0.990102	2.622688	-0.176513
C	-0.386115	3.969978	0.008810
H	-0.014459	4.364991	-0.942715
H	0.473998	3.921418	0.685479
H	-1.107545	4.676207	0.418205
H	-0.375509	1.828116	-0.589091

No imaginary frequency.

Optimized energies, coordinates, and frequencies for ωB97x-D3BJ/def2-TZVP calculations:

<u>1 (singlet)</u>

INNER ENERGY

The inner energy is: U= E(el) + E(ZPE) + E(vib) + E(rot) + E(trans)
E(el) - is the total energy from the electronic structure calculation
= E(kin-el) + E(nuc-el) + E(el-el) + E(nuc-nuc)
E(ZPE) - the the zero temperature vibrational energy from the frequency calculation
E(vib) - the the finite temperature correction to E(ZPE) due to population
of excited vibrational states
E(rot) - is the rotational thermal energy
E(trans)- is the translational thermal energy

Summary of contributions to the inner energy U:
Electronic energy ... -115.41885603 Eh
Zero point energy ... 0.03136847 Eh 19.68 kcal/mol
Thermal vibrational correction ... 0.00099991 Eh 0.63 kcal/mol
Thermal rotational correction ... 0.00141627 Eh 0.89 kcal/mol
Thermal translational correction ... 0.00141627 Eh 0.89 kcal/mol
----
Total thermal energy -115.38365511 Eh

Summary of corrections to the electronic energy:
(perhaps to be used in another calculation)
Total thermal correction 0.00383245 Eh 2.40 kcal/mol
Non-thermal (ZPE) correction 0.03136847 Eh 19.68 kcal/mol

Total correction 0.03520092 Eh 22.09 kcal/mol

CARTESIAN COORDINATES (ANGSTROEM)

C	-0.597422	-0.000006	-3.799447
C	-0.853710	0.000005	-2.544167
C	-1.117790	0.000017	-1.251652
H	-2.140904	-0.000008	-0.882364
H	-0.320597	-0.000008	-0.511728

No imaginary frequency.

SCF STABILITY ANALYSIS RESULT

RHF/RKS->UHF/UKS - triplet - external

Root	Eigenvalue (au)
0	0.031082
1	0.037304
2	0.046138

Stability Analysis indicates a stable HF/KS wave function.

<u>1 (triplet)</u>

INNER ENERGY

The inner energy is: U= E(el) + E(ZPE) + E(vib) + E(rot) + E(trans)
E(el) - is the total energy from the electronic structure calculation
= E(kin-el) + E(nuc-el) + E(el-el) + E(nuc-nuc)
E(ZPE) - the the zero temperature vibrational energy from the frequency calculation
E(vib) - the the finite temperature correction to E(ZPE) due to population
of excited vibrational states
E(rot) - is the rotational thermal energy
E(trans)- is the translational thermal energy

Summary of contributions to the inner energy U:
Electronic energy ... -115.36927989 Eh
Zero point energy ... 0.03046194 Eh 19.12 kcal/mol
Thermal vibrational correction ... 0.00098993 Eh 0.62 kcal/mol
Thermal rotational correction ... 0.00141627 Eh 0.89 kcal/mol
Thermal translational correction ... 0.00141627 Eh 0.89 kcal/mol
----
Total thermal energy -115.33499548 Eh

Summary of corrections to the electronic energy:
(perhaps to be used in another calculation)
Total thermal correction 0.00382247 Eh 2.40 kcal/mol
Non-thermal (ZPE) correction 0.03046194 Eh 19.12 kcal/mol

Total correction 0.03428441 Eh 21.51 kcal/mol

CARTESIAN COORDINATES (ANGSTROEM)

C	-0.608606	-0.000002	-3.778486
C	-0.850782	0.000003	-2.574598
C	-1.118687	-0.000001	-1.236957
H	-2.141693	0.000000	-0.882837
H	-0.310654	-0.000001	-0.516482

No imaginary frequency.

SCF STABILITY ANALYSIS RESULT

UHF/UKS->UHF/UKS - singlet - internal

Root	Eigenvalue (au)
0	0.016878

1	0.073984
2	0.117111

Stability Analysis indicates a stable HF/KS wave function.

36a (singlet)

INNER ENERGY

The inner energy is: U= E(el) + E(ZPE) + E(vib) + E(rot) + E(trans)
E(el) - is the total energy from the electronic structure calculation
= E(kin-el) + E(nuc-el) + E(el-el) + E(nuc-nuc)
E(ZPE) - the the zero temperature vibrational energy from the frequency calculation
E(vib) - the the finite temperature correction to E(ZPE) due to population
of excited vibrational states
E(rot) - is the rotational thermal energy
E(trans)- is the translational thermal energy

Summary of contributions to the inner energy U:
Electronic energy ... -346.68095852 Eh
Zero point energy ... 0.11537079 Eh 72.40 kcal/mol
Thermal vibrational correction ... 0.00460066 Eh 2.89 kcal/mol
Thermal rotational correction ... 0.00141627 Eh 0.89 kcal/mol
Thermal translational correction ... 0.00141627 Eh 0.89 kcal/mol
----
Total thermal energy -346.55815452 Eh

Summary of corrections to the electronic energy:
(perhaps to be used in another calculation)
Total thermal correction 0.00743320 Eh 4.66 kcal/mol
Non-thermal (ZPE) correction 0.11537079 Eh 72.40 kcal/mol

Total correction 0.12280400 Eh 77.06 kcal/mol

CARTESIAN COORDINATES (ANGSTROEM)

C	-0.505554	-0.293003	-4.111919
C	-0.829860	-0.132307	-2.894592
C	-1.163453	0.034926	-1.611923
H	-2.162939	0.402454	-1.376406
C	-0.300143	-0.237623	-0.474980
C	-0.785962	-0.022933	0.818812
C	0.020714	-0.272111	1.917450

C	1.314636	-0.744132	1.727749
C	1.806227	-0.962484	0.442065
C	1.006087	-0.709315	-0.656390
H	-1.798894	0.342658	0.954382
H	-0.355251	-0.102779	2.919837
H	1.945718	-0.943380	2.587095
H	2.816792	-1.328918	0.302798
H	1.369917	-0.868055	-1.665594

No imaginary frequency.

SCF STABILITY ANALYSIS RESULT

RHF/RKS->UHF/UKS - triplet - external

Root	Eigenvalue (au)
0	0.030102
1	0.042958
2	0.048939

Stability Analysis indicates a stable HF/KS wave function.

36a (triplet)

INNER ENERGY

The inner energy is: U= E(el) + E(ZPE) + E(vib) + E(rot) + E(trans)
E(el) - is the total energy from the electronic structure calculation
= E(kin-el) + E(nuc-el) + E(el-el) + E(nuc-nuc)
E(ZPE) - the the zero temperature vibrational energy from the frequency calculation
E(vib) - the the finite temperature correction to E(ZPE) due to population
of excited vibrational states
E(rot) - is the rotational thermal energy
E(trans)- is the translational thermal energy

Summary of contributions to the inner energy U:
Electronic energy ... -346.62815841 Eh
Zero point energy ... 0.11417386 Eh 71.65 kcal/mol
Thermal vibrational correction ... 0.00459961 Eh 2.89 kcal/mol
Thermal rotational correction ... 0.00141627 Eh 0.89 kcal/mol
Thermal translational correction ... 0.00141627 Eh 0.89 kcal/mol

----
Total thermal energy -346.50655240 Eh

Summary of corrections to the electronic energy:
(perhaps to be used in another calculation)
Total thermal correction 0.00743215 Eh 4.66 kcal/mol
Non-thermal (ZPE) correction 0.11417386 Eh 71.65 kcal/mol

Total correction 0.12160601 Eh 76.31 kcal/mol

CARTESIAN COORDINATES (ANGSTROEM)

C	-0.474625	-0.302083	-4.082085
C	-0.816226	-0.139530	-2.922431
C	-1.180343	0.040330	-1.597428
H	-2.182326	0.406427	-1.399769
C	-0.316757	-0.230390	-0.483432
C	-0.784975	-0.018567	0.826139
C	0.025923	-0.274453	1.917468
C	1.321950	-0.747684	1.730317
C	1.800620	-0.959833	0.438746
C	0.996108	-0.706204	-0.656606
H	-1.795788	0.348520	0.972901
H	-0.350604	-0.105820	2.920429
H	1.956530	-0.950240	2.585870
H	2.810598	-1.325923	0.289805
H	1.367950	-0.871551	-1.661540

No imaginary frequency.

SCF STABILITY ANALYSIS RESULT

UHF/UKS->UHF/UKS - singlet - internal

Root	Eigenvalue (au)
0	0.057306
1	0.077617
2	0.100603

Stability Analysis indicates a stable HF/KS wave function.

36b (singlet)

INNER ENERGY

The inner energy is: U= E(el) + E(ZPE) + E(vib) + E(rot) + E(trans)
E(el) - is the total energy from the electronic structure calculation
= E(kin-el) + E(nuc-el) + E(el-el) + E(nuc-nuc)
E(ZPE) - the the zero temperature vibrational energy from the frequency calculation
E(vib) - the the finite temperature correction to E(ZPE) due to population
of excited vibrational states
E(rot) - is the rotational thermal energy
E(trans)- is the translational thermal energy

Summary of contributions to the inner energy U:
Electronic energy ... -154.78236967 Eh
Zero point energy ... 0.06054124 Eh 37.99 kcal/mol
Thermal vibrational correction ... 0.00216528 Eh 1.36 kcal/mol
Thermal rotational correction ... 0.00141627 Eh 0.89 kcal/mol
Thermal translational correction ... 0.00141627 Eh 0.89 kcal/mol
----
Total thermal energy -154.71683060 Eh

Summary of corrections to the electronic energy:
(perhaps to be used in another calculation)
Total thermal correction 0.00499783 Eh 3.14 kcal/mol
Non-thermal (ZPE) correction 0.06054124 Eh 37.99 kcal/mol

Total correction 0.06553907 Eh 41.13 kcal/mol

CARTESIAN COORDINATES (ANGSTROEM)

C	-3.468651	2.046754	0.436254
C	-2.262378	2.336647	0.142909
C	-1.007219	2.638760	-0.162358
C	-0.387952	3.981381	0.013105
H	-0.019791	4.340404	-0.953691
H	0.486073	3.895097	0.667161
H	-1.093123	4.697862	0.429685
H	-0.368407	1.855583	-0.573054

No imaginary frequency.

36b (triplet)

INNER ENERGY

The inner energy is: U= E(el) + E(ZPE) + E(vib) + E(rot) + E(trans)
E(el) - is the total energy from the electronic structure calculation
= E(kin-el) + E(nuc-el) + E(el-el) + E(nuc-nuc)
E(ZPE) - the the zero temperature vibrational energy from the frequency calculation
E(vib) - the the finite temperature correction to E(ZPE) due to population
of excited vibrational states
E(rot) - is the rotational thermal energy
E(trans)- is the translational thermal energy

Summary of contributions to the inner energy U:
Electronic energy ... -154.72590355 Eh
Zero point energy ... 0.05974324 Eh 37.49 kcal/mol
Thermal vibrational correction ... 0.00219588 Eh 1.38 kcal/mol
Thermal rotational correction ... 0.00141627 Eh 0.89 kcal/mol
Thermal translational correction ... 0.00141627 Eh 0.89 kcal/mol
----
Total thermal energy -154.66113189 Eh

Summary of corrections to the electronic energy:
(perhaps to be used in another calculation)
Total thermal correction 0.00502843 Eh 3.16 kcal/mol
Non-thermal (ZPE) correction 0.05974324 Eh 37.49 kcal/mol

Total correction 0.06477166 Eh 40.64 kcal/mol

CARTESIAN COORDINATES (ANGSTROEM)

C	-3.449620	2.082363	0.450124
C	-2.283526	2.318993	0.147554
C	-0.989003	2.620198	-0.177729
C	-0.382314	3.974222	0.008672
H	-0.012280	4.368301	-0.943928
H	0.476405	3.923583	0.687106
H	-1.105677	4.679533	0.418233
H	-0.375431	1.825297	-0.590021

No imaginary frequency.

Single point energies and T1 diagnostics for DLPNO-CCSD(T)/def2-TZVP//B2PLYP/def2-TZVP calculations:

1 (singlet)

------------------------- --------------------

FINAL SINGLE POINT ENERGY -115.108988038479
------------------------- --------------------
T1 diagnostic … 0.018198424

1 (triplet)
------------------------- --------------------
FINAL SINGLE POINT ENERGY -115.061902371308
------------------------- --------------------
T1 diagnostic ... 0.034251038

36a (singlet)
------------------------- --------------------
FINAL SINGLE POINT ENERGY -345.730086988464
------------------------- --------------------
T1 diagnostic ... 0.013455663

36a (triplet)
------------------------- --------------------
FINAL SINGLE POINT ENERGY -345.681285964734
------------------------- --------------------
T1 diagnostic ... 0.023118288

36b (singlet)
------------------------- --------------------
FINAL SINGLE POINT ENERGY -154.353211297816
------------------------- --------------------
T1 diagnostic ... 0.016060927

36b (triplet)
------------------------- --------------------
FINAL SINGLE POINT ENERGY -154.299943828744
------------------------- --------------------
T1 diagnostic ... 0.029050369

Single point energies and T1 diagnostics for DLPNO-CCSD(T)/def2-TZVP//B3LYP/def2-TZVP calculations:

1 (singlet)
------------------------- --------------------
FINAL SINGLE POINT ENERGY -115.108811972956
------------------------- --------------------
T1 diagnostic ... 0.018112492

1 (triplet)
------------------------- --------------------
FINAL SINGLE POINT ENERGY -115.061900427165

------------------------ -------------------

T1 diagnostic ... 0.034250541

36a (singlet)

------------------------ -------------------

FINAL SINGLE POINT ENERGY -345.729837605154

------------------------ -------------------

T1 diagnostic ... 0.013398368

36a (triplet)

------------------------ -------------------

FINAL SINGLE POINT ENERGY -345.681285964734

------------------------ -------------------

T1 diagnostic ... 0.023118288

36b (singlet)

------------------------ -------------------

FINAL SINGLE POINT ENERGY -154.353049572243

------------------------ -------------------

T1 diagnostic ... 0.016023814

36b (triplet)

------------------------ -------------------

FINAL SINGLE POINT ENERGY -154.299942266552

------------------------ -------------------

T1 diagnostic ... 0.029049272

Single point energies and T1 diagnostics for DLPNO-CCSD(T)/def2-TZVP//PBE0/def2-TZVP calculations:

1 (singlet)

------------------------ -------------------

FINAL SINGLE POINT ENERGY -115.108806596152

------------------------ -------------------

T1 diagnostic ... 0.018130007

1 (triplet)

------------------------ -------------------

FINAL SINGLE POINT ENERGY -115.061875598772

------------------------ -------------------

T1 diagnostic ... 0.034321223

36a (singlet)

------------------------ -------------------

FINAL SINGLE POINT ENERGY -345.729590739183

------------------------ -------------------

T1 diagnostic ... 0.013376277

36a (triplet)

------------------------- --------------------
FINAL SINGLE POINT ENERGY -345.681044812385
------------------------- --------------------
T1 diagnostic ... 0.023115630

36b (singlet)

------------------------- --------------------
FINAL SINGLE POINT ENERGY -154.353007447171
------------------------- --------------------
T1 diagnostic ... 0.016105693

36b (triplet)

------------------------- --------------------
FINAL SINGLE POINT ENERGY -154.299878434473
------------------------- --------------------
T1 diagnostic ... 0.029070881

Single point energies and T1 diagnostics for DLPNO-CCSD(T)/def2-TZVP//ωB97x-D3BJ/def2-TZVP calculations:

1 (singlet)

------------------------- --------------------
FINAL SINGLE POINT ENERGY -115.108793133114
------------------------- --------------------
T1 diagnostic ... 0.018116231

1 (triplet)

------------------------- --------------------
FINAL SINGLE POINT ENERGY -115.061961985820
------------------------- --------------------
T1 diagnostic ... 0.034293389

36a (singlet)

------------------------- --------------------
FINAL SINGLE POINT ENERGY -345.729874736565
------------------------- --------------------
T1 diagnostic ... 0.013370113

36a (triplet)

------------------------- --------------------
FINAL SINGLE POINT ENERGY -345.681405711120
------------------------- --------------------
T1 diagnostic ... 0.023077255

36b (singlet)

-------------------------   --------------------

FINAL SINGLE POINT ENERGY   -154.353065545429

-------------------------   --------------------

T1 diagnostic   ...   0.015975522

36b (triplet)

-------------------------   --------------------

FINAL SINGLE POINT ENERGY   -154.300001050939

-------------------------   --------------------

T1 diagnostic   ...   0.029050641
